# Macrophage depletion blocks congenital SARM1-dependent neuropathy

**DOI:** 10.1172/JCI159800

**Published:** 2022-12-01

**Authors:** Caitlin B. Dingwall, Amy Strickland, Sabrina W. Yum, Aldrin K.Y. Yim, Jian Zhu, Peter L. Wang, Yurie Yamada, Robert E. Schmidt, Yo Sasaki, A. Joseph Bloom, Aaron DiAntonio, Jeffrey Milbrandt

**Affiliations:** 1 Department of Genetics, Washington University School of Medicine, St. Louis, Missouri, USA.; 2 Division of Neurology, Children’s Hospital of Philadelphia, Department of Neurology, Perelman School of Medicine, Philadelphia, Pennsylvania, USA.; 3 Department of Pathology and Immunology, Washington University School of Medicine, St. Louis, Missouri, USA.; 4 Needleman Center for Neurometabolism and Axonal Therapeutics, St. Louis, Missouri, USA.; 5 Department of Developmental Biology, Washington University School of Medicine, St. Louis, Missouri, USA.

**Keywords:** Neuroscience, Macrophages, Mouse models, Neurodegeneration

## Abstract

Axon loss contributes to many common neurodegenerative disorders. In healthy axons, the axon survival factor NMNAT2 inhibits SARM1, the central executioner of programmed axon degeneration. We identified 2 rare *NMNAT2* missense variants in 2 brothers afflicted with a progressive neuropathy syndrome. The polymorphisms resulted in amino acid substitutions V98M and R232Q, which reduced NMNAT2 NAD^+^-synthetase activity. We generated a mouse model to mirror the human syndrome and found that *Nmnat2^V98M/R232Q^* compound-heterozygous CRISPR mice survived to adulthood but developed progressive motor dysfunction, peripheral axon loss, and macrophage infiltration. These disease phenotypes were all SARM1-dependent. Remarkably, macrophage depletion therapy blocked and reversed neuropathic phenotypes in Nmnat2^V98M/R232Q^ mice, identifying a SARM1-dependent neuroimmune mechanism as a key driver of disease pathogenesis. These findings demonstrate that SARM1 induced inflammatory neuropathy and highlight the potential of immune therapy as a treatment for this rare syndrome and other neurodegenerative conditions associated with NMNAT2 loss and SARM1 activation.

## Graphical abstract

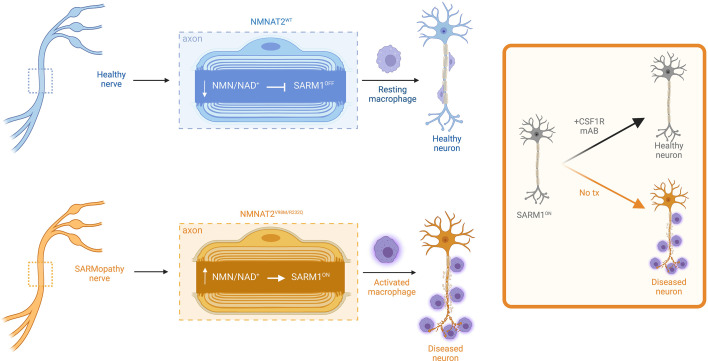

## Introduction

Axon loss is one of the earliest pathological hallmarks — and likely the initiating event — in many common neurodegenerative disorders (1–3). Programmed axon degeneration is an active, genetically encoded, subcellular, self-destruction pathway executed by the prodegenerative NADase enzyme sterile α and Toll/interleukin-1 receptor motif-containing (SARM1) (4, 5). In a healthy axon, SARM1 activity is restrained by the NAD^+^ biosynthetic enzyme nicotinamide mononucleotide adenylyltransferase 2 (NMNAT2), which converts NMN and ATP into NAD^+^ (6). NMNAT2 is a highly labile protein produced in the soma and trafficked into the axon (7). Nerve injury blocks axonal transport and leads to rapid depletion of axonal NMNAT2 (8), causing NMN buildup and NAD^+^ loss. Recent breakthroughs led to the discovery that SARM1 is activated by an increase in the ratio of NMN to NAD^+^ (9). NMN and NAD^+^ can both bind at an allosteric site in the enzyme’s N-terminus to differentially regulate its conformation, and hence, the activation state of SARM1 (9–12). The ratio of NMN/NAD^+^ rises after NMNAT2 loss, favoring SARM1 activation and subsequent axon degeneration (9). Genetic deletion of NMNAT2 alone is perinatal lethal; however, when combined with SARM1 deletion, mice are viable and resistant to injury-induced axon degeneration, suggesting that an essential role of NMNAT2 is the inhibition of SARM1 (13).

SARM1 is the central executioner of a cell-autonomous axon-degeneration program. Upon activation, SARM1, an NAD^+^ hydrolase, depletes axonal NAD^+^ levels, culminating in metabolic crisis and axon fragmentation. Acute injury is the best-understood trigger of pathological axon degeneration, inducing distal axon loss (5, 14–19). Loss of SARM1 is protective in models of chemotherapy-induced peripheral neuropathy (CIPN) (15, 17) and traumatic brain injury (TBI) (16). However, programmed axon degeneration is also common in chronic neurodegenerative disease models that do not include acute axonal injury (3), suggesting a role for subacute SARM1 activation in the pathogenesis of a wide range of chronic neurodegenerative conditions.

Evidence for the involvement of the NMNAT2/SARM1 axon degeneration pathway in chronic disease has emerged from studying rare human mutations in patients. Indeed, in a model of Leber congenital amaurosis type 9, SARM1 depletion rescues photoreceptor cell death caused by loss of the nuclear NMNAT isoform, *NMNAT1* (20). Furthermore, *NMNAT2* mutations were identified in a still-born fetus with fetal akinesia deformation sequence and in 2 sisters suffering from a mild polyneuropathy (21, 22). The first direct evidence of SARM1 mutations in human disease emerged with the discovery of rare hypermorphic *SARM1* alleles in a subset of patients with ALS (23, 24). However, we believe that until the present study, the field lacked a mechanistically defined disease model of a SARM1-dependent, chronic axonopathy (Herein referred to as SARMopathy).

Here we examine 2 brothers that presented in early childhood with recurring Guillain-Barré–like episodes requiring mechanical ventilation combined with severe, progressive peripheral neurodegeneration. Whole exome sequencing revealed that they are both compound heterozygotes for 2 rare missense variants in the *NMNAT2* gene, each inherited from 1 of their parents. We created a mouse that harbors both mutant *NMNAT2* alleles and found that this model recapitulates key features of the human syndrome. NMNAT2 is the endogenous inhibitor of SARM1 in axons; thus, defects in NMNAT2 can trigger aberrant activation of SARM1. Indeed, we find that these disease phenotypes are all SARM1-dependent. While SARM1 is best understood as the executioner of a cell-autonomous, axo-destructive program, here we make the surprising discovery that SARM1 also promotes axon degeneration via induction of non-cell-autonomous macrophage activation.

Macrophages play complex roles as both prodegenerative and prorestorative immune cells. Indeed, neuroinflammation has been referred to as a double-edged sword as it can have both beneficial and deleterious effects on the nervous system (25). On one hand, in the peripheral nervous system, macrophages play a necessary role in facilitating axon regeneration through the clearance of myelin and axonal debris after nerve injury (26–28). Indeed, macrophage depletion hampers axon regeneration after peripheral nerve injury (29–32). However, macrophages and their CNS counterparts, microglia, are also drivers of disease in several common neurodegenerative diseases, including multiple sclerosis (MS) (33), CIPN (34), and Alzheimer’s disease (AD) (35). In these disease models, depletion of macrophages and microglia can mitigate disease phenotypes, suggesting a conserved prodegenerative role for phagocytes in human neurological disease.

Collectively, our data establish *NMNAT2* variants as the genetic basis of a human neuropathy and demonstrate an unexpected role for SARM1 as a driver of neuroinflammation in the peripheral nervous system. In this model, we demonstrate that AAV-mediated delivery of a SARM1 dominant negative (SARM1-DN) gene therapeutic inhibited the development of motor neuropathy. In addition, we found that macrophage depletion early in the disease course could block the development of neuropathy and that, remarkably, treatment after symptom onset could reverse neuropathic phenotypes. Our study provides what we believe to be the first mouse model of a chronic, injury-independent SARMopathy with which to test axon degeneration-blocking treatments. This will be of substantial benefit and high clinical relevance as the field uncovers an ever-growing list of chronic neurodegenerative diseases that involve SARM1 activation. Importantly, our work uncovered a SARM1-dependent non-cell-autonomous mechanism of axon loss and highlights macrophage depletion as a potent axoprotective therapeutic strategy.

## Results

### Rare missense variants in NMNAT2 cause hereditary neuropathy.

Patients 1 and 2 are brothers from nonconsanguineous, healthy parents of African American ancestry. No other members of the extended family are known to be affected. Patient 1 was born following an uneventful pregnancy. Development was normal and the patient acquired the ability to walk before the onset of illness. At age 13 months, he experienced an acute episode of hypotonia, weakness, and respiratory failure that required hospitalization and mechanical ventilation. Electrophysiology testing (nerve conduction studies and electromyography) at the time of symptom onset showed features of multifocal, sensory, and motor neuropathy thought to be consistent with Guillain-Barré syndrome. After treatment with intravenous immune globulin and steroids, he regained some motor function and was taken off ventilatory support but exhibited residual weakness.

In subsequent years he developed a unique sensorimotor syndrome comprising both chronic and episodic features. Episodic attacks are frequently contemporaneous with infection and include severe neuropathic pain, worsening erythromelalgia, flaccid quadriparesis, and respiratory failure requiring mechanical ventilation. During these episodes, electrophysiological testing showed a complete absence of sensory and motor responses. In between episodes, the patient experienced a chronic, progressive motor-predominant peripheral neuropathy. Currently 25-years-old, patient 1 is cognitively normal and attends college. Electrophysiology testing indicated a predominantly motor axonal neuropathy. He is wheelchair-dependent, exhibits progressive scoliosis, poor weight gain, and has severe combined proximal and distal muscle atrophy with predominantly distal muscle weakness. Muscle ultrasound revealed fatty, fibrotic tissue replacement of muscle, consistent with chronic neuropathy (36). The patient also experiences recurring neuropathic pain, erythromelalgia, bilateral optic atrophy, and tongue fasciculation. Cranial and spinal MRI are normal except for mildly prominent extra-axial spaces. At age 15, a head CT showed mild diffuse parenchymal atrophy or pseudoatrophy.

Patient 2 was born 3 years after patient 1 following an uneventful pregnancy. His first episode of severe weakness requiring mechanical ventilation occurred at 11 months. Patient 2’s clinical course has been virtually identical to his brother’s with very similar symptoms and degree of impairment.

**Figure 1:**
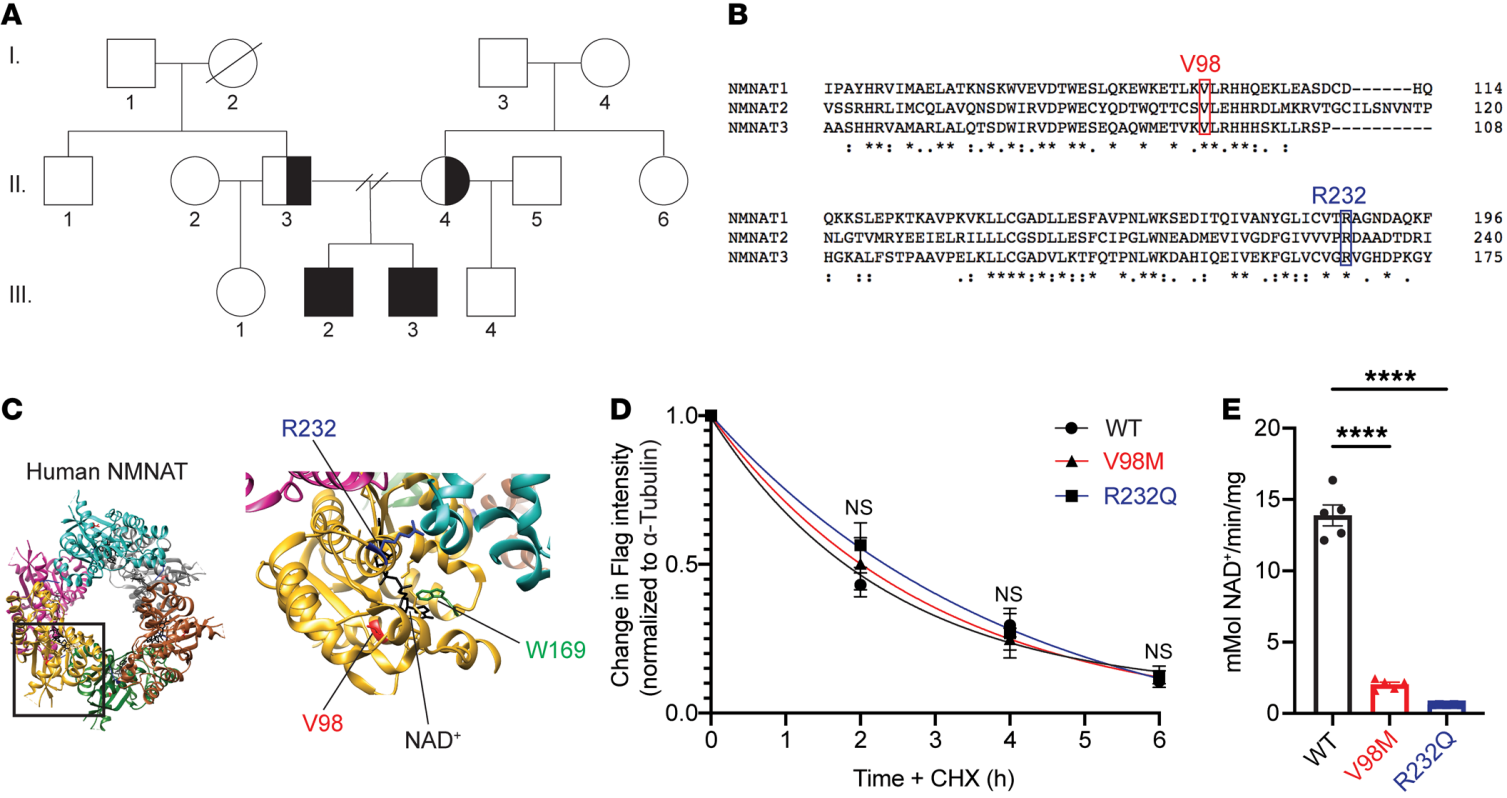
Identification of compound heterozygous *NMNAT2* variants in 2 brothers with relapsing-remitting neuropathy. (**A**) Both brothers carry 2 extremely rare missense mutations in the *NMNAT2* gene (c.292G>A. c.695G>A), each inherited from 1 of their parents. Half-shaded represents heterozygous, unaffected. Fully-shaded represents compound heterozygous, affected. (**B**) V98 and R232 residues are conserved in all 3 human *NMNAT* isoforms. (**C**) Schematic of NMNAT structure. Patients’ missense variants noted in red (V98) and blue (R232). W169 (green) is the catalytic residue. (**D**) Relative turnover rates for Flag-NMNAT2 (WT, V98M, R232Q) after cycloheximide (CHX) addition. 1-phase decay curves were fitted to the data using nonlinear regression. (**E**) NMNAT activity assay. NAD^+^ production at steady state (10 minutes) was used to calculate the NMNAT activity. All data are presented as mean ± SEM from *n* = 5 independent experiments. Statistical significance determined by 2-way ANOVA with multiple comparisons. ****P < 0.0001.

Whole-exome sequencing was performed on the brothers and their parents to identify candidate variants that may have caused the patients’ disease. Both affected patients share rare, compound heterozygous variants (c. 695G>A [p.Arg232Gln] and c.292G>A [p.Val98Met]; R232Q and V98M, respectively) in *NMNAT2*. Each parent was found to be heterozygous for 1 of the 2 variants identified in the patients (Figure 1A). R232Q was previously identified as a loss-of-function variant associated with fetal akinesia deformation sequence and occurs in a region of NMNAT2 involved in substrate binding (22). V98M appeared to be a novel NMNAT2 variant of unknown significance. Both variants occurred at residues that are conserved in all 3 human NMNAT isoforms (Figure 1, B and C).

### V98M reduces NMNAT2 NAD^+^ synthetase activity but not protein stability.

We sought to elucidate the functional consequences of the NMNAT2 variant alleles. To investigate whether NMNAT2^V98M^ altered protein stability, we used immunoblotting to compare its relative half life to NMNAT2^R232Q^ and NMNAT2^WT^ in transfected HEK cells. In line with prior studies of NMNAT2 half life (8), protein synthesis blockade led to a rapid drop in NMNAT2 protein levels. Turnover rates of NMNAT2^V98M^ and NMNAT2^R232Q^ were not significantly different from that of control NMNAT2 (Figure 1D).

NMNAT2 is an NAD^+^ synthesizing enzyme, and this activity is required for its function as an axon survival factor. To investigate whether NMNAT2^V98M^ has impaired enzymatic activity, we purified recombinant Strep-tagged NMNAT2 proteins using affinity chromatography (37). In agreement with previous findings (22), NMNAT2^V98M^ had 14.6% of the NAD^+^ synthesis activity of NMNAT2^WT^ at 37°C, whereas NMNAT2^R232Q^ was 4.4% as active as the NMNAT2^WT^ enzyme (Figure 1E). Collectively, these data demonstrate that these *NMNAT2* variants disrupt enzymatic function, which may underly their pathogenicity.

### Nmnat2^V98M/R232Q^ mice develop progressive motor neuropathy.

**Figure 2:**
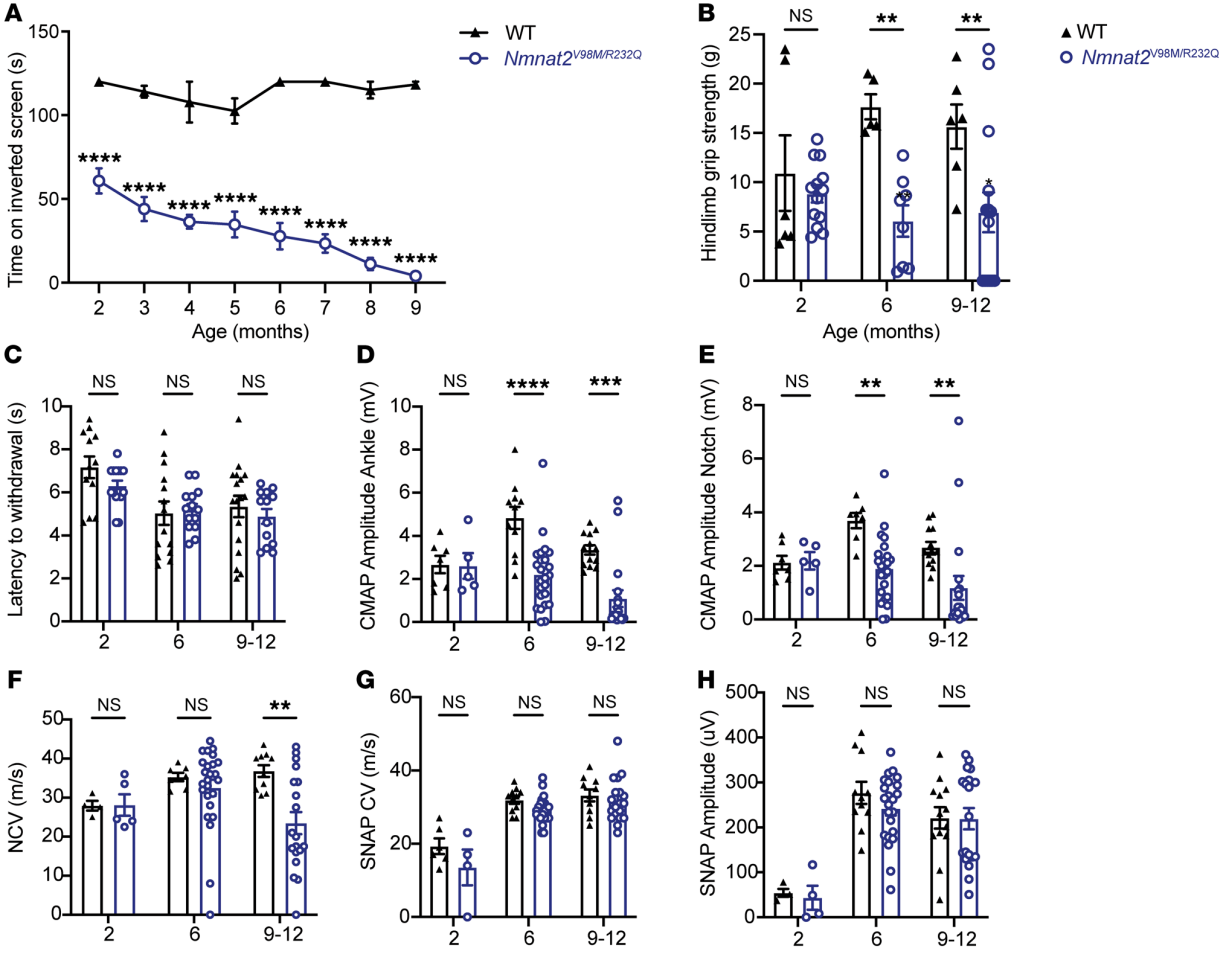
*Nmnat2^V98M/R232Q^* mice have behavioral and electrophysiologic features consistent with a motor neuropathy. (**A**) Average time suspended from an inverted screen (max. 120 seconds) for WT (*n* = 3–7) or *Nmnat2*^V98M/R232Q^ (*n* = 3–21) mice. (**B**) Hindlimb grip strength for WT (*n* = 5–6) or *Nmnat2^V98M/R232Q^* (*n* = 8–15) male mice at 2, 6, and 9–12 months. (**C**) Average time it takes for WT (*n* = 12–17) or *Nmnat2^V98M/R232Q^* (*n* = 13–15) mice to remove their tails from a 55°C hot water bath at 2, 6, and 9–12 months. (**D** and **E**) CMAP amplitude of WT (*n* = 7–13) and *Nmnat2^V98M/R232Q^* (*n* = 5–25) mice at the ankle (**D**) and sciatic notch (**E**) at 2, 6, and 9–12 months. (**F**) NCV (m/s) of sciatic nerves of WT (*n* = 4–10) or *Nmnat2^V98M/R232Q^* (*n* = 5–25) mice at 2, 6, and 9–12 months (**G**) SNAP CV (m/s) of sciatic nerves of WT (*n* = 6–11) or *Nmnat2^V98M/R232Q^* (*n* = 4–24) mice at 2, 6, and 9–12 months. (**H**) SNAP amplitude (μV) of sciatic nerves of WT (*n* = 6–11) and *Nmnat2^V98M/R232Q^* (*n* = 4–24) mice at 2, 6, and 9–12 months. All data are presented as mean ± SEM. Statistical significance determined by 2-way ANOVA with multiple comparisons. **P < 0.01, ***P < 0.001, ****P < 0.0001.

To study the pathological effects of the V98M and R232Q *NMNAT2* mutations found in these patients, we used CRISPR-induced mutagenesis to create mice with the *Nmnat2^V98M^* or *Nmnat2^R232Q^* mutations (see Methods). Mice heterozygous for these individual mutations were mated to generate mice with compound heterozygous *Nmnat2^V98M^* and *Nmnat2^R232Q^* mutations (henceforth referred to as *Nmnat2^V98M/R232Q^* mice). These mice were viable with no evidence of embryonic or perinatal lethality. As patients with compound heterozygous variants in *NMNAT2* exhibit a chronic, motor-predominant peripheral neuropathy, we searched for similar phenotypes in the *Nmnat2^V98M/R232Q^* mice. Starting at 2 months, we assayed muscle strength using an inverted screen test and found that the mice exhibited age-dependent, progressive muscle weakness (Figure 2A). The human disorder involves predominantly distal muscle atrophy; therefore, we assayed hindlimb grip strength in the *Nmnat2^V98M/R232Q^* mice. We observed a decline in distal muscle strength (Figure 2B). Gait defects manifested in *Nmnat2^V98M/R232Q^* mice as young as 6 months old, concomitant with progressive lower limb muscular atrophy. The majority of mice displayed severe hindlimb wasting and difficulty walking by 9–12 months of age (Supplemental Video 1; supplemental material available online with this article; https://doi.org/10.1172/JCI159800DS1). Notably, while patients have episodic neuropathic pain, we did not elicit a nociceptive defect in tail flick testing of *Nmnat2^V98M/R232Q^* mice (Figure 2C).

### Nmnat2^V98M/R232Q^ mice have electrophysiologic features consistent with a motor neuropathy.

The decreased muscle strength observed in *Nmnat2^V98M/R232Q^* mice suggested motor neuron dysfunction. We measured motor fiber function using compound muscle action potential (CMAP) amplitudes and found significant deficits in *Nmnat2^V98M/R232Q^* mice. The abnormalities worsened with age, suggesting that motor axon numbers progressively diminished in parallel with decreasing overall strength (Figure 2, D and E). Next, we tested motor nerve conduction velocity (NCV). A decrease in NCV early in disease without altered CMAP amplitudes was indicative of demyelination, whereas a progressive drop in NCV concomitant with low CMAP amplitudes indicated large-diameter axon loss. The NCV in young *Nmnat2^V98M/R232Q^* mice was normal, indicating that the disease is primarily an axonal neuropathy; however, NCV did decrease with age, likely due to the eventual loss of large diameter axons (Figure 2F). Electrophysiologic sensory testing demonstrated that large, myelinated sensory axons were not affected in *Nmnat2^V98M/R232Q^* mice (Figure 2, G and H). Pain is transmitted by small and thinly myelinated fibers; thus, nerve conduction studies are typically unaffected (38). Rather, intraepidermal nerve fiber density (IENFD) analysis is a more sensitive measure of small fiber loss. In agreement with normal nociceptive function, we found that IENFD was unaffected in *Nmnat2^V98M/R232Q^* mice (Supplemental Figure 1). Altogether, these data demonstrate that *Nmnat2^V98M/R232Q^* mice had a motor axonal neuropathy, consistent with the chronic electrophysiological features of human patients with *NMNAT2*-associated neuropathy.

### Nmnat2 variants cause progressive axon loss and muscle wasting in mice.

**Figure 3:**
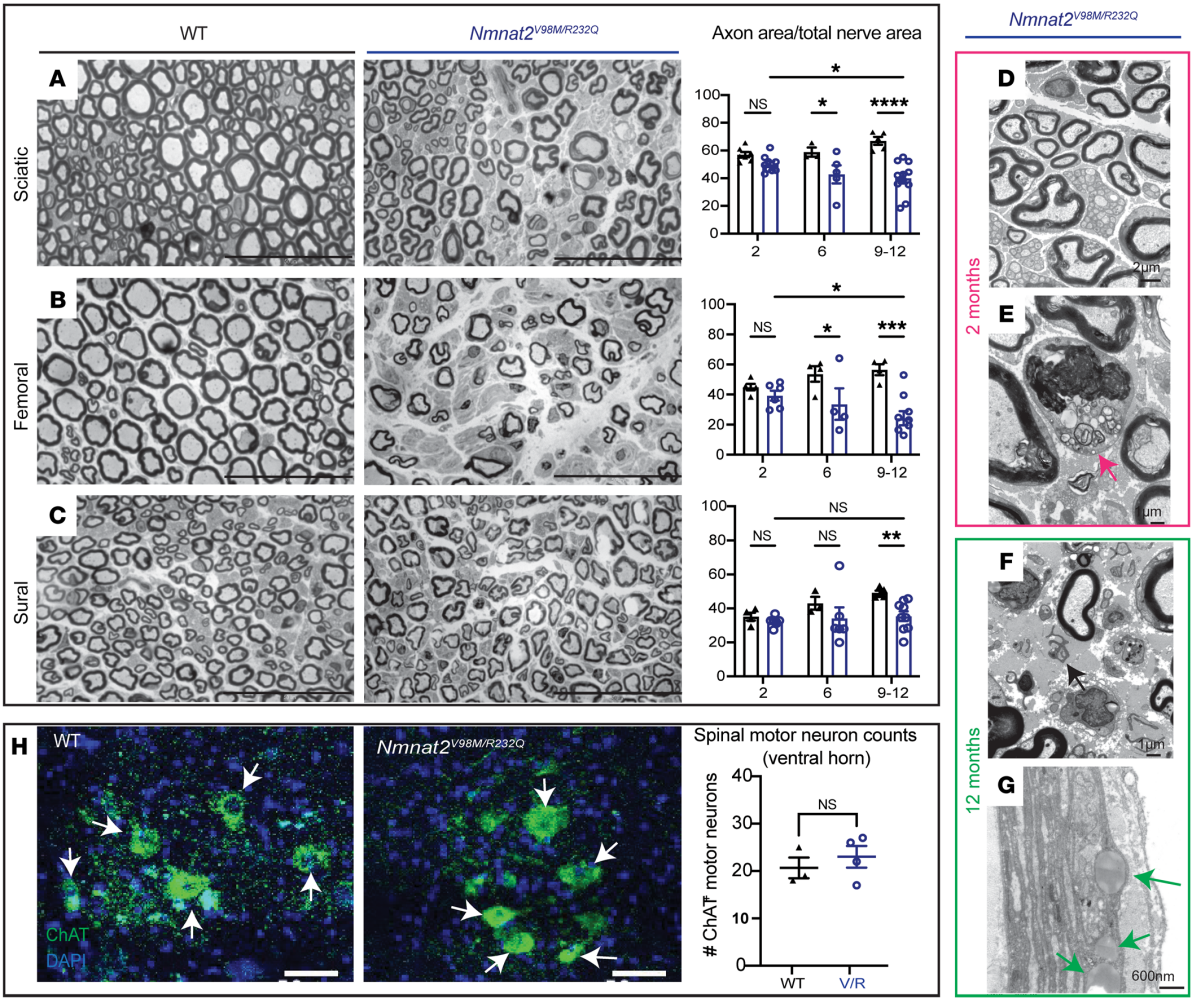
*Nmnat2* variants cause progressive axon loss in mice. (**A**–**C**) Representative images of sciatic (**A**), femoral (**B**), and sural (**C**) nerves in 9–12-month-old *Nmnat2^V98M/R232Q^* (*n* = 9) or WT (*n* = 5) mice. Percent axonal area/total nerve area are indicated to the right (*n* = 4–11 mice per age cohort, per genotype). Scale bars: 50 μm. (**D**) *Nmnat2^V98M/R232Q^* sciatic nerve (2 months): dense population of large and small myelinated axons with little intervening extracellular space. (**E**) *Nmnat2^V98M/R232Q^* sciatic nerve (2 months): macrophage containing axonal and myelin debris in the endoneurial. (**F**) *Nmnat2^V98M/R232Q^* sciatic nerve (12 months): patches of marked axon loss with increased collagen and wispy processes of SC. Scattered macrophages with axonal and myelin debris were identified. (**G**) *Nmnat2^V98M/R232Q^* sciatic nerve (12 months): presence of large perineurial droplets of neutral fat. (**H**) Representative images of ChAT immunostaining in 12-month-old *Nmnat2^V98M/R232Q^* (*n* = 4) or WT (*n* = 3) spinal cord (ventral horn), scale bars: 50 μm. Quantification of number of ChAT^+^ motor neuron cell bodies in the ventral horn to the right. All data are presented as mean ± SEM. Statistical significance determined by Student’s unpaired, 2-tailed *t* test or 2-way ANOVA with multiple comparisons. *P < 0.05, **P < 0.01, ***P < 0.001, ****P < 0.0001.

To further characterize the disease process in *Nmnat2^V98M/R232Q^* mice, we used light microscopic analysis to examine the pathology of select peripheral nerves including the sciatic (a mixed nerve), femoral (primarily motor), and sural (primarily sensory) in 2-month, 6-month, and 9–12-month old mice. The sciatic and femoral nerves exhibited severe, progressive axon loss. In contrast, we did not observe progressive axon loss in the sural nerve; however, total axon area was modestly different from *Nmnat^WT^* control mice at 9–12 months of age (Figure 3, A–C). Myelin thickness was not affected in any of the nerves we examined (Supplemental Figure 2).

We next performed electron microscopic analysis on the sciatic nerves of 2-month and 12-month-old *Nmnat2^V98M/R232Q^* mice. The appearance of the sciatic nerve at 2 months was normal and showed a dense population of large and small myelinated axons with little intervening extracellular space (Figure 3D). Schwann cells (SCs) and macrophages containing axonal and myelin debris were found in the endoneurial space (Figure 3E, arrow). The sciatic nerve at 12 months showed patches of marked axon loss with increased collagen and wispy processes of SCs (Figure 3F, arrow). Following axon degeneration, perineurial cells take up lipid droplets from myelin breakdown (39); indeed, we found large perineurial droplets of neutral fat in the 12-month sciatic nerve (Figure 3G, arrow). To confirm that the observed peripheral defects were not due to motor neuron cell death, we immunostained the spinal cords of 12-month-old *Nmnat2^WT^* and *Nmnat2^V98M/R232Q^* mice for the motor neuron marker choline acetyltransferase (ChAT) (Figure 3H). Motor neuron cell numbers in the ventral horn were equivalent between genotypes, and thus, axon loss in the nerves of *Nmnat2^V98M/R232Q^* mice was likely not due to motor neuron cell death. Taken together, these pathological features demonstrate a progressive peripheral axonal neuropathy.

**Figure 4:**
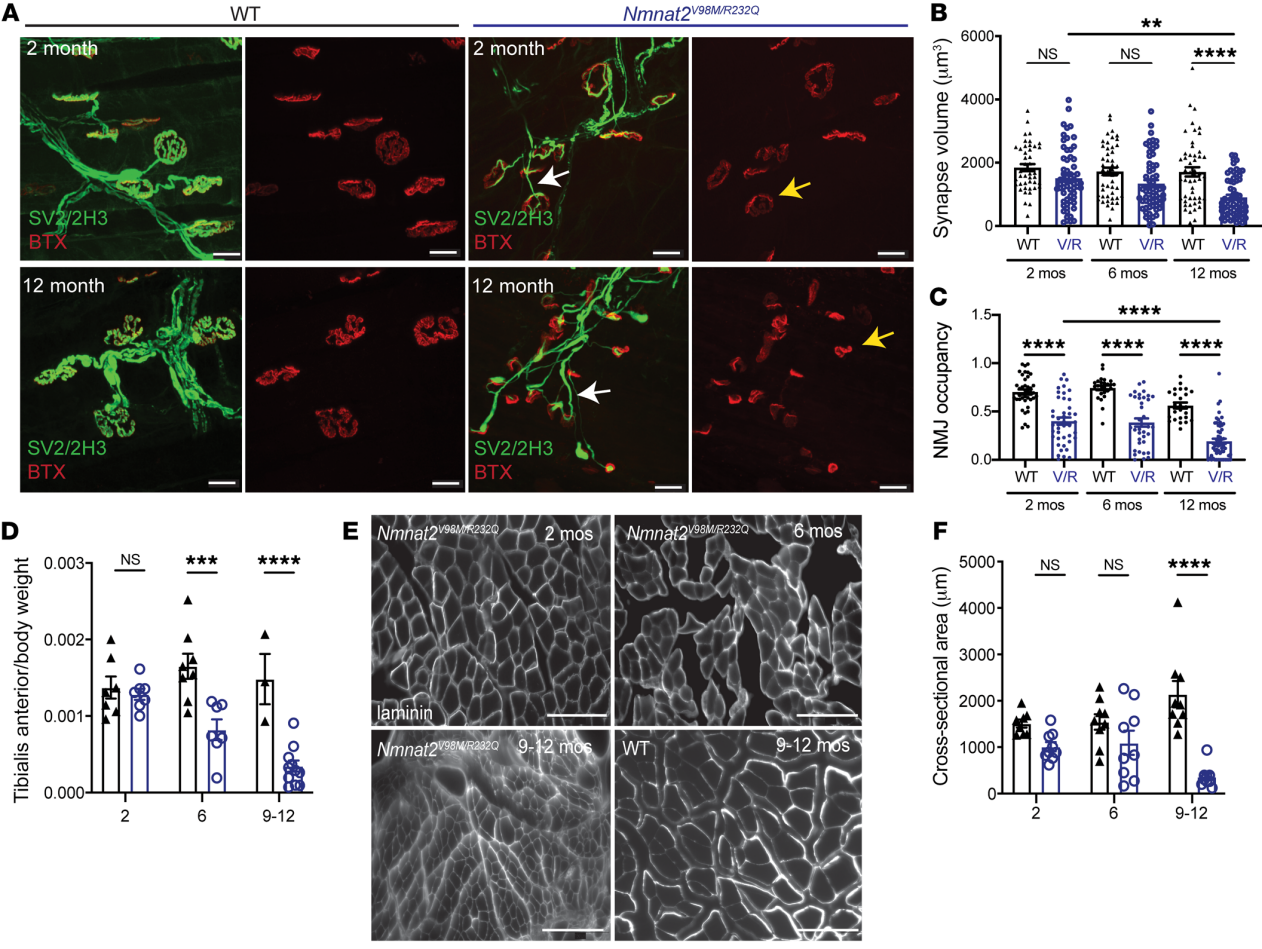
*Nmnat2* variants cause NMJ dysfunction and muscle wasting in mice. (**A**) Representative images of 2- and-12 month-old mouse NMJs stained for synaptic vesicle 2/neurofilament (green) and bungarotoxin (red). Scale bars: 20 μm. (**B**) Synapse volume quantification for NMJs from WT (*n* = 44–62 synapses) and *Nmnat2^V98M/R232Q^* (V/R; *n* = 48–63 synapses) mice. (**C**) NMJ occupancy quantification for NMJs from WT (*n* = 25–43 synapses) and *Nmnat2^V98M/R232Q^* (*n* = 25–55 synapses) mice at 2, 6, and 12 months. (**D**) Average tibialis anterior weight/body weight for WT and *Nmnat2^V98M/R232Q^* mice at 2, 6, and 9–12 months (*n* = 3–11 mice per age cohort, per genotype). (**E**) Representative images of laminin immunofluorescence in *Nmnat2^V98M/R232Q^* mouse tibialis anterior muscles at 2, 6, and 9–12 months. WT mouse tibialis anterior muscle at 12 months shown for comparison. The apparent fuzziness shown in the representative image of *Nmnat2^V98M/R232Q^* 9–12-month-old mouse muscle was a consistent genotype-dependent finding reflecting diffuse laminin staining. (**F**) Quantification of muscle fiber cross-sectional area (*n* = 3 mice per genotype, per age cohort). Scale bars: 150 μm. All data are presented as mean ± SEM. Statistical significance determined by Student’s unpaired, 2-tailed *t* test or 2-way ANOVA with multiple comparisons. **P < 0.01, ***P < 0.001, ****P < 0.0001.

We next examined the neuromuscular junctions in the hind paw lumbrical muscles of *Nmnat2^V98M/R232Q^* mice. We found the neuromuscular junction (NMJ) endplate size was slightly diminished in *Nmnat2^V98M/R232Q^* mice even as early as 2 months of age (Figure 4A, yellow arrows). The NMJ postsynaptic volume continued to progressively diminish over time, consistent with loss of presynaptic inputs (Figure 4, A and B). Apposition of the presynaptic nerve terminal and the postsynaptic endplate is a major determinant of NMJ functionality. Indeed, the ratio of overlap between presynaptic vesicles and the underlying acetylcholine receptor clusters (NMJ occupancy) was reduced in the NMJs of 2-month-old *Nmnat2^V98M/R232Q^* mice and continued to decrease over time (Figure 4, A and C). In addition, endplate complexity was decreased in 12 month old *Nmnat2^V98M/R232Q^* mice, whereas the prototypical pretzel-like endplate structure was still observed in 2 month old *Nmnat2^V98M/R232Q^* mice (Figure 4A, yellow arrows). Alterations in endplate size and complexity suggest repeated episodes of denervation and reinnervation. At steady state, the majority of *Nmnat2^V98M/R232Q^* endplates appeared partially innervated (Figure 4A); however, almost all preterminal motor axons were abnormally thin and smooth (Figure 4A, white arrows), a hallmark of sprouting axons (40). Sprouting is frequently observed in NMJs of motor neuron disease models and is evidence of continual axonal degeneration and regeneration (40–43). Taken together, these data indicate that decreased NMNAT2 activity causes progressive degeneration of terminal axons at the NMJ, which is consistent with a chronic motor neuropathy.

The patients with compound heterozygous variants in *NMNAT2* have both proximal and distal weakness with predominantly distal muscle atrophy, rendering them wheelchair bound. Loss of nerve terminals at the NMJ results in muscle fiber denervation and eventual muscle atrophy. *Nmnat2^V98M/R232Q^* mice exhibited a progressive reduction in hindlimb muscle mass (Figure 4D) that correlated with decreased fiber cross-sectional area in the tibialis anterior muscle (Figure 4, E and F). Together, these results confirm that the decreased activity of the *Nmnat2^V98M/R232Q^* mice leads to axon loss and subsequent muscle denervation and atrophy. Importantly, the *Nmnat2^V98M/R232Q^* mouse model recapitulates chronic motor features of the human syndrome, providing strong evidence that the *NMNAT2* variants are indeed pathogenic.

### Neuronal SARM1 is required for Nmnat2^V98M/R232Q^ neuropathy.

**Figure 5:**
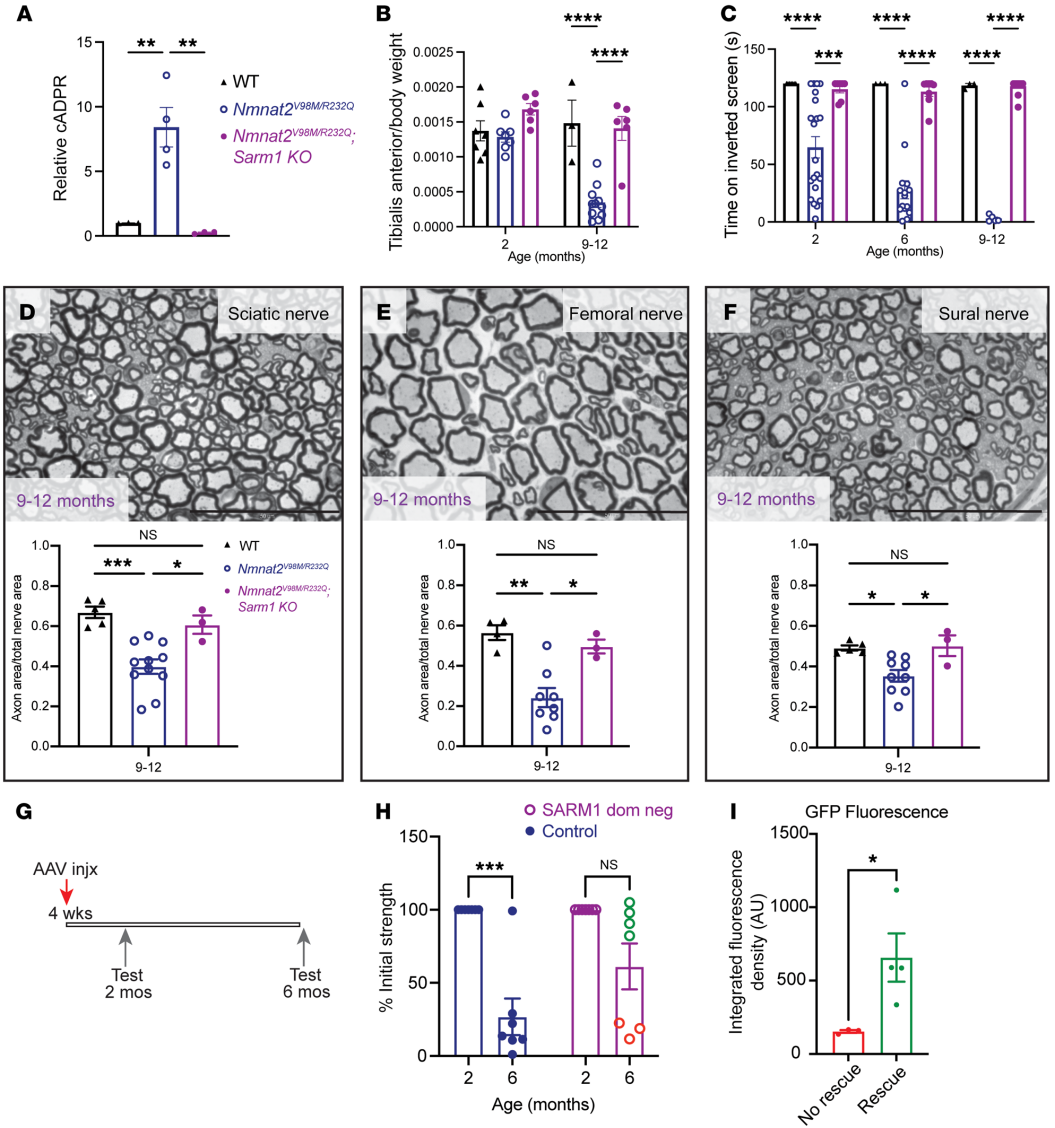
Neuronal SARM1 is required for *Nmnat2^V98M/R232Q^* neuropathy. (**A**) Relative cADPR levels in sciatic nerves of 2-month-old WT (*n* = 3), *Nmnat2^V98M/R232Q^* (*n* = 4), and *Nmnat2^V98M/R232Q^; Sarm1-KO* (*n* = 3) mice. Values normalized to WT cADPR levels (set to 1). Statistical significance determined by a Student’s unpaired *t* test. (**B**) Average tibialis anterior weight by body weight for WT, *Nmnat2^V98M/R232Q^,* and *Nmnat2^V98M/R232Q^; Sarm1-KO* mice in 2- and 9–12-month-old mice (*n* = 3–11 mice per age cohort, per genotype). (**C**) Average time suspended from an inverted screen (max. 120 seconds) for WT (*n* = 3–7), *Nmnat2^V98M/R232Q^* (*n* = 3–21), and *Nmnat2^V98M/R232Q^; Sarm1-KO* (*n* = 7–13) mice. (**D**–**F**) Representative images of sciatic (**D**), femoral (**E**), and sural (**F**) nerves in 9–12-month-old *Nmnat2^V98M/R232Q^; Sarm1-KO* mice. Scale bars: 50 μm. Percent axonal area/total nerve area is calculated below each corresponding nerve (*n* = 3–11 mice per age cohort, per genotype). Statistical significance was determined by 2-way ANOVA with multiple comparisons. (**G**) Schematic of AAV-SARM1-DN gene therapy experiment. (**H**) Percent initial performance on inverted screen test at 2 months and 6 months for EGFP (control) (*n* = 6) or SARM1-DN–injected (*n* = 7) *Nmnat2^V98M/R232Q^* mice. (**I**) Quantification of GFP fluorescence in the spinal cord of SARM1-DN injected *Nmnat2^V98M/R232Q^* mice, stratified by rescue (Rescue was determined as an endpoint performance (6m) greater than the mean control arm endpoint performance.) All data are presented as mean ± SEM. Statistical significance within treatment group was determined by a Student’s paired, 2-tailed *t* test. Statistical significance between treatment groups determined by a Student’s unpaired, 2-tailed *t* test.*P < 0.05, **P < 0.01, ***P < 0.001, ****P < 0.0001.

SARM1 is a prodegenerative enzyme activated by binding to the NAD^+^ precursor NMN at its allosteric site (9). NMNAT2 converts NMN to NAD^+^, thereby preventing the buildup of NMN and its interaction with SARM1. In *Nmnat2*-KO mice, the increase in NMN leads to axon projection abnormalities and perinatal death; however, *Nmnat2/Sarm1* double-KO mice are viable and completely resistant to injury-induced programmed axon degeneration (13). To determine whether SARM1 is activated in *Nmnat2^V98M/R232Q^* mice, we first monitored nerve levels of cyclic ADP ribose (cADPR), a specific biomarker of SARM1 NAD^+^ hydrolase activity (44). Metabolites were isolated from the sciatic nerve of 2-month-old *Nmnat2^V98M/R232Q^* mice and analyzed by liquid chromatography with tandem mass spectrometry (LC-MS/MS). We found that cADPR levels were elevated 8-fold compared with *Nmnat2^WT^,* and that this increase was fully SARM1-dependent (Figure 5A). This demonstrated that SARM1 was activated even at this early stage of the disease and suggested that cADPR is likely an useful biomarker for syndromes involving chronic SARM1 activation.

Next, we mated the *Nmnat2^V98M/R232Q^* mutant mice with *Sarm1*-KO mice to generate *Nmnat2^V98M/R232Q^*; *Sarm1*–KO mice. In contrast with *Nmnat2^V98M/R232Q^* (*Sarm1* WT) mice, *Nmnat2^V98M/R232Q^*; *Sarm1*–KO mice did not develop motor function deficits (Figure 5, B and C). We also performed morphological analysis of the sural, femoral, and sciatic nerves of *Nmnat2^V98M/R232Q^* and *Nmnat2^V98M/R232Q^*; *Sarm1*-KO mice. As with the functional studies, loss of *Sarm1* prevented axon degeneration even in the oldest *Nmnat2^V98M/R232Q^* mice (Figure 3A and Figure 5, D–F). These results confirm that the phenotypes associated with these pathogenic *NMNAT2* variants are SARM1-dependent and do not arise secondary to neomorphic functions of the mutant NMNAT2 enzymes. These results suggest that *Nmnat2^V98M/R232Q^* mice will be useful for testing treatment strategies for progressive neurodegenerative disease involving chronic SARM1 activation.

The development of small molecule and gene therapy SARM1 inhibitors is underway (45, 46); indeed, we previously showed that adeno-associated virus–mediated (AAV-mediated), neuron-specific expression of a potent SARM1 dominant-negative (SARM1-DN) variant blocks pathologic axon degeneration in models of acute nerve injury (45, 47). With the discovery that the *Nmnat2^V98M/R232Q^* motor neuropathy is SARM1-dependent, we next tested whether this SARM1 gene therapy approach could similarly block disease in these mice. One-month-old *Nmnat2^V98M/R232Q^* mice received intrathecal injections of AAV virions (6 × 10^11^) expressing SARM1-DN-EGFP or EGFP alone (control) under control of a neuron-specific synapsin promoter (Figure 5G). We assayed inverted screen performance at 2 months and 6 months for both groups and determined therapeutic efficacy by comparing 6-month to 2-month performance for each mouse. *Nmnat2^V98M/R232Q^* mice injected with EGFP alone displayed an approximately 73% decline in strength by 6 months of age (*P* < 0.0001) (Figure 5H). In contrast, *Nmnat2^V98M/R232Q^* mice injected with SARM1-DN exhibited a 39% decline (NS, *P >* 0.05) at 6 months of age (Figure 5H). Importantly, the SARM1-DN rescue of strength defects was dependent on the extent of viral infection and transgene expression in the spinal cord, i.e., higher expression correlated with higher endpoint performance (Figure 5I). These results demonstrated that neuron-specific expression of SARM1-DN potently protects *Nmnat2^V98M/R232Q^* mice from developing motor deficits and that neuron-autonomous SARM1 activity is pathogenic in *Nmnat2^V98M/R232Q^* mice.

### Macrophages orchestrate Nmnat2^V98M/R232Q^ neuropathy.

**Figure 6:**
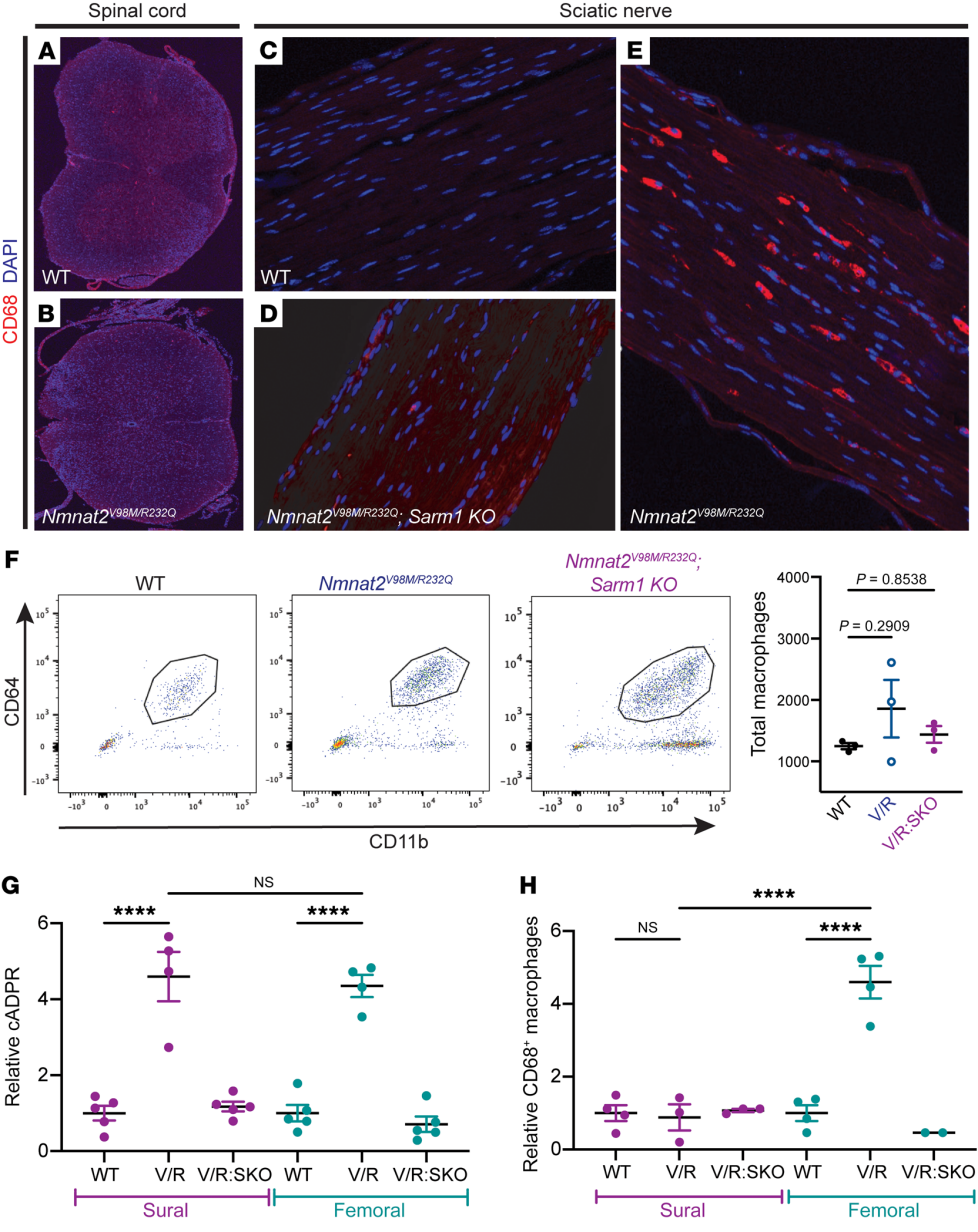
Activated macrophages accumulate in the peripheral nervous system of *Nmnat2^V98M/R232Q^* mice. (**A**–**E**) Representative images of CD68 immunofluorescence and DAPI signal in the spinal cord, original magnification, ×10 (**A** and **B**) and sciatic nerves, original magnification, ×20 (**C**–**E**) of 2-month-old WT, *Nmnat2^V98M/R232Q^,* and *Nmnat2^V98M/R232Q^; Sarm1-KO* mice. (**F**) Representative scatter plots and quantification of fluorescence-activated cell sorting of total sciatic nerve macrophages (CD64^+^ CD11b^+^) in 2-month-old WT (*n* = 3), *Nmnat2^V98M/R232Q^* (*n* = 3), and *Nmnat2^V98M/R232Q^; Sarm1-KO* (*n* = 3) mice. (**G**) Relative cADPR levels in sural and femoral nerves of 4-month-old WT (*n* = 5), *Nmnat2^V98M/R232Q^* (*n* = 4), and *Nmnat2^V98M/R232Q^; Sarm1-KO* (*n* = 5) mice. Values normalized to WT cADPR levels (set to 1). (**H**) Relative levels of CD68^+^ macrophages in sural and femoral nerves of 4-month-old WT (*n* = 4), *Nmnat2^V98M/R232Q^* (*n* = 3-4) and *Nmnat2^V98M/R232Q^; Sarm1-KO* (*n* = 2-3) mice. Values normalized to WT CD68^+^ macrophage levels (set to 1). All data are presented as mean ± SEM. Statistical significance determined by 1-way ANOVA with multiple comparisons, ****P < 0.0001.

After acute nerve injury, macrophages infiltrate the lesion and phagocytose axonal and myelin debris, clearing the injury site and promoting axonal regeneration (26–28). In models of chronic neurodegenerative disease, macrophages and their CNS counterpart, microglia, play complex immunomodulatory roles as both proinflammatory and antiinflammatory mediators (34, 35, 48–54). We immunostained central (spinal cord) and peripheral (sciatic nerve) nervous tissue with antibodies against the activated macrophage marker CD68 (55). There were few CD68^+^ cells in central and peripheral tissues of *Nmnat2^WT^* mice (Figure 6, A and C). In contrast, in sciatic nerves of 2-month-old *Nmnat2^V98M/R232Q^* mice, CD68^+^ activated macrophages (Figure 6E) were abundant, concomitant with a trend toward elevated total (CD64^+^CD11b^+^) nerve macrophages (Figure 6F). In contrast, Nmnat2^V98M/R232Q^ mice lacking SARM1 at the same age (Figure 6D) had significantly fewer CD68^+^ macrophages, and the number of CD64^+^CD11b^+^ macrophages in these animals was similar to the number seen in Nmnat2^WT^ mice (Figure 6F). CD68^+^ macrophages were not detected in the spinal cords of *Nmnat2^V98M/R232Q^* mice (Figure 6B), consistent with the predominantly peripheral nervous system defects seen in the patients.

The mixed sensory/motor sciatic nerve and motor-predominant femoral nerve were both preferentially affected in *Nmnat2^V98M/R232Q^* mice, while the sensory-predominant sural nerve was spared. Thus, we questioned whether differences in SARM1 activation between motor and sensory nerves could underlie their differential susceptibility to axon loss or macrophage activation. We first measured cADPR levels in the femoral and sural nerves of the *Nmnat2^V98M/R232Q^* mice. We found that both nerves exhibited robust SARM1 activation (Figure 6G). Next, we asked whether SARM1 activation was sufficient to induce macrophage activation in both nerves, akin to what we observed in the sciatic nerve. Femoral nerves of *Nmnat2^V98M/R232Q^* mice had elevated numbers of activated CD68^+^ macrophages compared with the nerves of *Nmnat2^WT^* and *Nmnat2^V98M/R232Q^; Sarm1-KO* mice (Figure 6H); in contrast, the sural nerves of *Nmnat2^V98M/R232Q^, Nmnat2^WT^,* and *Nmnat2^V98M/R232Q^; Sarm1-KO* mice had comparable levels of CD68^+^ macrophages (Figure 6H). Since cADPR was elevated in sciatic, femoral, and sural nerves, but macrophage activation preferentially occurred only in sciatic and femoral nerves, this finding strongly suggested that SARM1 activation was necessary — but not sufficient — for macrophage activation. In addition to SARM1 activation in motor axons, these additional unknown signals are required for preferential activation of the macrophage response.

NAD^+^ biosynthesis plays a role in programming macrophage immune responses (56); thus, we examined whether intrinsic defects exist in macrophages of *Nmnat2^V98M/R232Q^* mice due to impaired NMNAT2 activity. However, we found no differences in either basal or antigen-induced activation between *Nmnat2^WT^* and *Nmnat2^V98M/R232Q^*-derived murine peritoneal macrophages (Supplemental Figure 3). This is consistent with findings from studies examining other disorders that indicate that signals within the neural microenvironment shape macrophage activation (57–61). Here, this is demonstrated by increased numbers of activated macrophages in the sciatic nerves of Nmnat2^V98M/R232Q^ mice.

**Figure 7:**
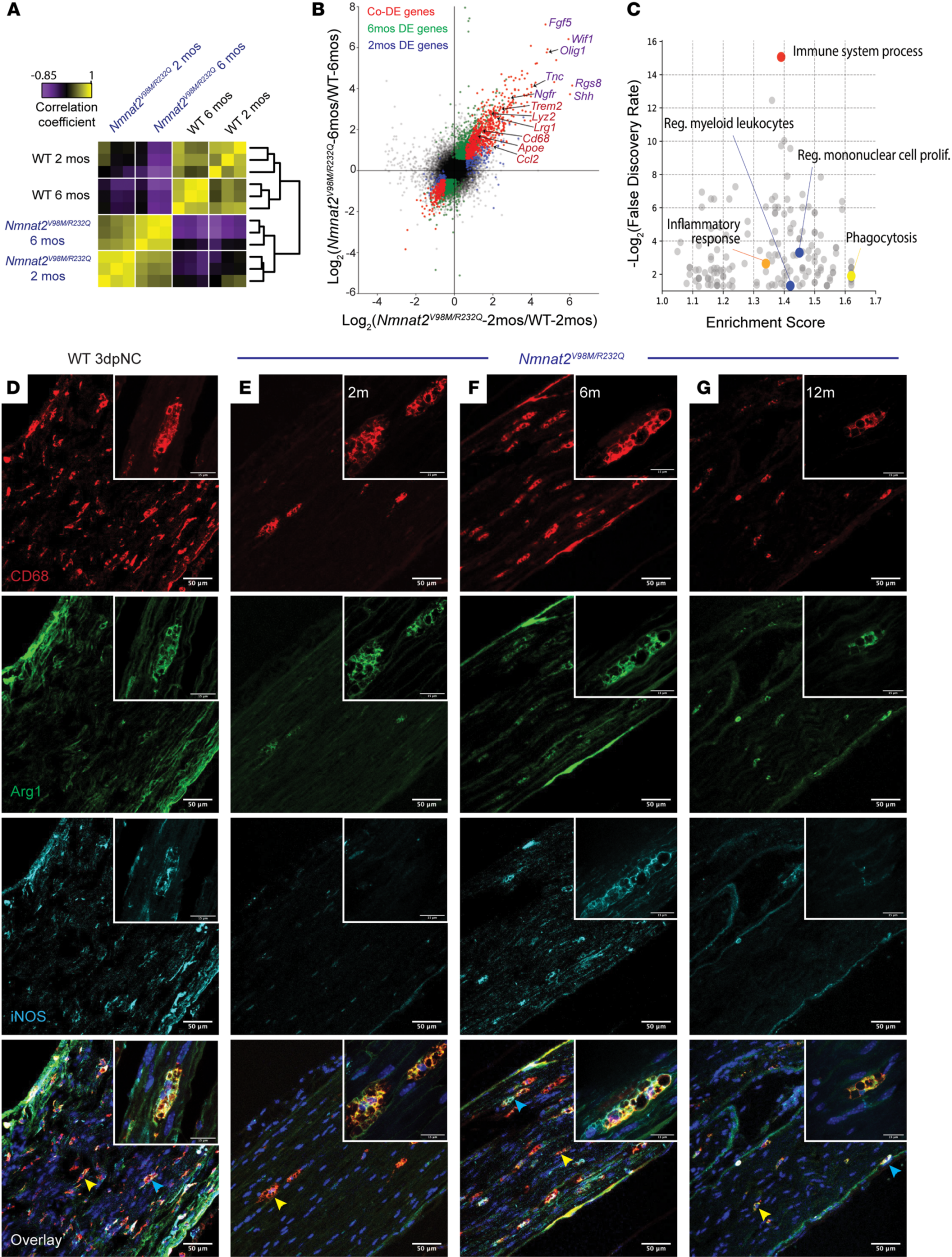
Macrophages are activated throughout disease and express markers of M1 and M2 polarization. (**A**) Sample correlation plot showing global transcriptomic analysis and hierarchical clustering of sciatic nerve macrophages from WT and *Nmnat2^V98M/R232Q^* mice. Each box represents 1 replicate (*n* = 3). (**B**) Volcano plot of significant codifferentially expressed (DE) genes in sciatic nerves of 2 month and 6 month *Nmnat2^V98M/R232Q^* old mice, highlighting activated macrophage markers (red) and repair SC signatures (purple). (**C**) GO analysis of genes enriched in sciatic nerves of 6-month-old *Nmnat2^V98M/R232Q^* mice. (**D**–**G**) Representative images of CD68, Arg1, and iNOS immunofluorescence in the sciatic nerves of (**D**) WT mice 3 days after nerve crush (3dpNC) and (**E**–**G**) *Nmnat2^V98M/R232Q^* sciatic nerves at 2, 6, and 12 months. Scale bars: 50 μm, 15 μm (insets). Yellow arrows indicate Arg1/CD68 colocalization and blue arrows represent Arg1/iNOS/CD68 colocalization. Inset depicts a magnified image of a polarized macrophage. All data are presented as mean ± SEM. Statistical significance determined by 1-way ANOVA with multiple comparisons.

We performed bulk RNA-Seq on the sciatic nerves of *Nmnat2^V98M/R232Q^* and *Nmnat2^WT^* mice at early and late disease stages to capture dynamic changes in the glial and immune cell milieu (Figure 7A). Global transcriptomic analysis revealed similarities within the nerves of *Nmnat2^V98M/R232Q^* mice at both 2 months and 6 months, which clustered more closely to each other than to nerves of *Nmnat2^WT^* mice. Closer inspection revealed sets of activated macrophage signatures (57) — including *Cd68, Trem2, Apoe, Lrg1,* and *Ccl2* — upregulated in the sciatic nerves of both 2-month-old and 6-month-old *Nmnat2^V98M/R232Q^* mice (Figure 7B), consistent with our observations of CD68^+^ nerve-associated macrophages in *Nmnat2^V98M/R232Q^* mice. Repair SC markers (62–64) such as *Fgf5, Shh, Ngfr,* and *Olig1* were also upregulated, suggesting a dedifferentiation program of myelinating SCs. Gene ontology (GO) analysis showed significant enrichment of genes related to the immune response, inflammation, and phagocytosis in both 2-month-old and 6-month-old *Nmnat2^V98M/R232Q^* mice (Figure 7C and Supplemental Tables 1 and 2). Altogether, these data demonstrate chronic activation of peripheral nervous system macrophages and an ongoing SC repair program in this mouse model.

Increased numbers of activated macrophages in the nerves of the *Nmnat2^V98M/R232Q^* mice raised the question of whether they were playing a beneficial or destructive role in the disorder. Classically, macrophages are categorized into 2 opposite extremes: proinflammatory (M1) or antiinflammatory (M2) (65), although intermediate, overlapping states are now recognized (66). Indeed, these states may influence the role macrophages play in disease development. M1 activation is canonically associated with expression of inducible nitric oxide synthase (iNOS), while M2 activation is associated with expression of arginase-1 (Arg1) (67). To investigate the activation state of macrophages in the *Nmnat2^V98M/R232Q^* neuropathy, we profiled expression of both iNOS and Arg1 in activated macrophages throughout disease. We found that all activated macrophages (CD68^+^) coexpressed Arg1 at all examined ages (Figure 7, E–G, yellow arrow), while expression of iNOS was more dynamic, with Arg1 and iNOS coexpression (Figure 7, E–G, blue arrow) in 0%, 34.16% ± 2.204 (*n* = 3, *P* < 0.0001), and 12.71% ± 3.776 (*n* = 3, *P* = 0.0039) of macrophages at 2, 6, and 12 months of age, respectively. This noncanonical activation state was also observed in the sciatic nerves of *Nmnat2^WT^* mice 3 days after nerve crush (Figure 7D, blue arrow), and has been similarly described in models of acute brain injury (68–70). Concomitant expression of both polarization signatures suggests that macrophages may respond to both proinflammatory and antiinflammatory signals within the nerve microenvironment and that macrophage biology in this system is more complex than a simple binary phenotype.

**Figure 8:**
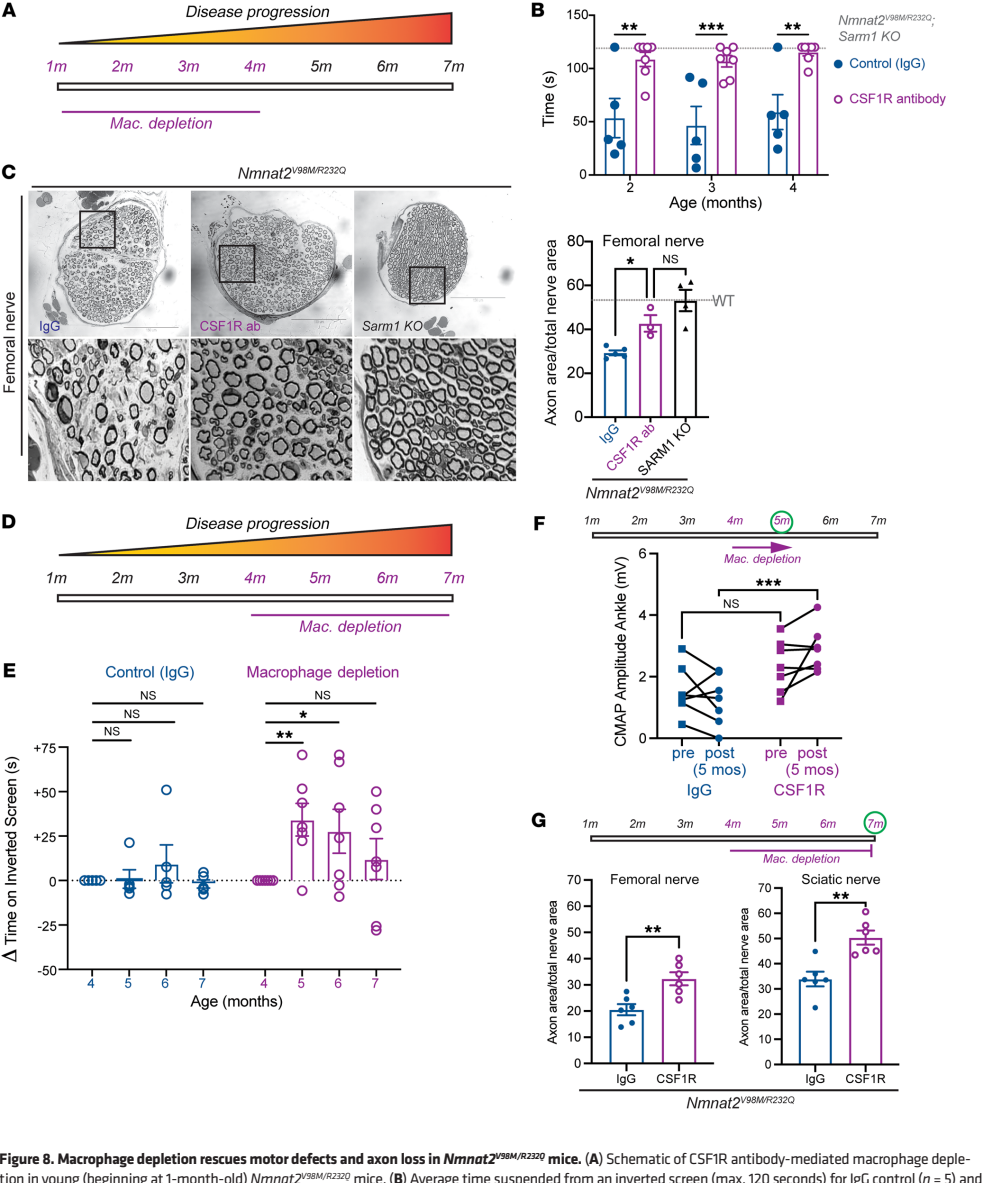
Macrophage depletion rescues motor defects and axon loss in *Nmnat2^V98M/R232Q^* mice. (**A**) Schematic of CSF1R antibody-mediated macrophage depletion in young (beginning at 1-month-old) *Nmnat2^V98M/R232Q^* mice. (**B**) Average time suspended from an inverted screen (max. 120 seconds) for IgG control (*n* = 5) and CSF1R-treated *Nmnat2^V98M/R232Q^* (*n* = 7) mice. Statistical significance was determined by 2-way ANOVA with multiple comparisons. (**C**) Representative images of femoral nerve from IgG (control) (*n* = 5), CSF1R (*n* = 3), or *Sarm1-KO* *Nmnat2^V98M/R232Q^* (*n* = 4) mice at 4 months. Scale bars: 150 μm. Percent axonal area/total nerve area for femoral nerve calculated to the right. (**D**) Schematic of CSF1R antibody-mediated macrophage depletion in aged (beginning at 4-month-old *Nmnat2^V98M/R232Q^* mice. (**E**) Change in inverted screen time (s) from pre-treatment (4 months) measured at 5, 6, and months, comparing CSF1R treatment (*n* = 7) and IgG (control) (*n* = 5). Statistical significance was determined by 2-way ANOVA with multiple comparisons. (**F**) CMAP amplitude (ankle) of *Nmnat2^V98M/R232Q^* mice before and after 1 month of CSF1R treatment (*n* = 7) or IgG treatment (*n* = 5). (**G**) Percent axonal area/total nerve area for femoral nerve and sciatic nerve calculated at 7 months (3 months of macrophage depletion (*n* = 6) or treatment with isotype control IgG (*n* = 6). All data are presented as mean ± SEM. Statistical significance determined by Student’s unpaired *t* test. *P < 0.05, **P < 0.01, ***P < 0.001.

Next, we employed a macrophage depletion strategy using colony stimulating factor 1 receptor (CSF1R) blockade (71, 72) to directly evaluate the function of macrophages in *Nmnat2^V98M/R232Q^* mice. The mice were treated with CSF1R monoclonal antibody or the isotype control antibody IgG every 3 weeks beginning at 1 month of age and were assessed using motor function tests at 2, 3, and 4 months of age (Figure 8A). The efficacy of the macrophage depletion treatment was confirmed by immunostaining for the pan-macrophage marker Iba1 alongside CD68 in sciatic nerves (Supplemental Figure 4, A–C). Given the preponderance of Arg1^+^ activated nerve macrophages, i.e., the canonical antiinflammatory macrophage phenotype, we predicted that macrophage depletion would worsen the neuropathy of *Nmnat2^V98M/R232Q^* mice. Remarkably, however, macrophage depletion completely blocked the development of muscle strength defects for the duration of the experiment. In contrast, *Nmnat2^V98M/R232Q^* mice treated with IgG continued to exhibit poor motor function (Figure 8B). Morphological examination of the femoral — predominantly motor — nerve showed that macrophage depletion significantly rescued axon loss in the nerve (Figure 8C), consistent with its ability to prevent motor function deficits. Together, these data show that macrophages promote axon degeneration that leads to motor function deficits in this neuropathic mouse model.

Given our finding that macrophages contribute to axonal dysfunction early in the disease, we next tested whether macrophage depletion would be therapeutic after the initiation of symptoms. By 4 months of age, *Nmnat2^V98M/R232Q^* mice exhibited profound pathological and functional motor disease (Figure 8, B and C). Thus, we administered the CSF1R monoclonal antibody (or control IgG) to 4-month-old *Nmnat2^V98M/R232Q^* mice to test whether macrophage depletion could halt or reverse disease progression (Figure 8D). One month after antibody treatment, the previously symptomatic mice demonstrated a significant increase in inverted screen performance, demonstrating a profound recovery of muscle strength (Figure 8E). Improvement in overall strength was accompanied by improved distal CMAP responses (Figure 8F). Such a treatment response indicated that endogenous reparative processes occurred after macrophage depletion to promote functional recovery. Indeed, in nerve and NMJ pathological studies, we detected ongoing axon degeneration and regeneration processes in *Nmnat2^V98M/R232Q^* mice at steady state. Rescue of muscle strength persisted until the mice were 7 months old, at which point muscle strength was maintained at pretreatment levels. Endpoint examination of macrophage-depleted peripheral nerves revealed significant rescue of sciatic and femoral nerve axon loss (Figure 8G).

CSF1R inhibition can alter macrophage polarization (73); thus, we examined whether the remaining population of macrophages after depletion exhibited altered activation or polarization phenotypes. We found a modest trend toward reduced activation of the remaining nerve macrophages in *Nmnat2^V98M/R232Q^* mice (Supplemental Figure 4D). However, we found no evidence of alterations to their polarization phenotype compared with IgG treated *Nmnat2^V98M/R232Q^* mice (Supplemental Figure 4, E and F, yellow and blue arrows), indicating that the constellation of polarization signals within the nerve microenvironment or within the remaining macrophages themselves was not altered by macrophage depletion.

Altogether, these data support a model wherein macrophage depletion blocks axon degeneration, tipping the balance toward innate axon reparative processes and, thus, symptom reversal. Importantly, the finding that macrophage depletion reversed both behavioral and electrophysiologic defects underscored the potential of acute macrophage-targeted therapies in chronic neurologic diseases.

## Discussion

Genetic deletion of NMNAT2 is perinatal lethal; however, when combined with SARM1 deletion, mice are viable and resistant to injury-induced axon degeneration (13). Recent advances in our understanding of the mechanisms underlying axon degeneration directly connected NMNAT2 loss to SARM1 activation through dynamic changes in the NMN-to-NAD^+^ ratio (9). These studies suggest that defects in NMNAT2 could predispose an individual to develop neurodegenerative disease; however, there is little direct evidence connecting mutations in the genes involved in axon degeneration to human neurological disease. Here, we examined 2 brothers with severe neuropathy associated with *NMNAT2* mutations. This neuropathy initially presented with sensory and motor symptoms and progressed to be a predominantly motor neuropathy with severe muscle wasting. To study the molecular mechanism underlying this syndrome, we developed a mouse model harboring the *Nmnat2^V98M/R232Q^* mutations. This mutant mouse recapitulated the cardinal motor features of the human syndrome. We found that NMNAT2 enzymatic deficiency lead to chronic SARM1 activation that, in turn, lead to non-cell-autonomous macrophage activation and axon loss. Our study has several important implications. First, the creation of our mouse model enables longitudinal examination of a SARMopathy and provides a powerful platform for testing novel axo-protective therapeutics in disorders of chronic SARM1 activation. Second, we found that a major prodegenerative role of SARM1 involved the activation of macrophages in parallel with its canonical cell-autonomous destructive functions. Finally, the identification of macrophages as key drivers of neuropathology suggested that macrophage depletion therapy could be efficacious in other diseases involving SARM1-mediated axon degeneration.

While axon degeneration is classically thought of as a programmed injury response to acutely damaged axons, there is growing evidence that this program is aberrantly activated in progressive neurodegenerative disease. Despite the abundance of acute injury models involving SARM1 activation, a chronic model that is more akin to human progressive neurodegenerative disorders is unavailable. Such a model is necessary for testing therapeutics under conditions of subacute SARM1 activation, which our work now shows involves previously unappreciated complex biological mechanisms. We found that SARM1 was activated at an early age in *Nmnat2^V98M/R232Q^* mice and that the disease is indeed fully SARM1-dependent. Moreover, this suggested that SARM1 activation and, thus, axon loss does not occur as an all-or-nothing event in chronic neurodegenerative disorders. Rather, this disorder appeared to be characterized by persistent SARM1 activity, suggesting that therapeutic intervention could be efficacious across a broad disease timeline in such disorders.

Patients with compound heterozygous variants *NMNAT2^V98M^* and *NMNAT2^R232Q^* develop a unique sensorimotor neuropathy involving both chronic and episodic symptomology. Chronic features of the disease are motor predominant, while episodic features involve prominent sensory and motor components. The patients typically develop acute episodes after infection, whereas the *Nmnat2^V98M/R232Q^* mouse model unsurprisingly shows no evidence of episodic disease features, likely due to housing in a pathogen-free environment. The mouse model does recapitulate cardinal features of the chronic symptomatology, including a motor axonal neuropathy, distal muscle wasting and weakness, and a progressive disease course. Unlike the patients, the *Nmnat2^V98M/R232Q^* mouse model had no evidence of a neuropathic pain phenotype, suggesting either species-dependent differences or that the episodic attacks seen in the patients but not in the mice contribute to the neuropathic symptoms. Interestingly, 2 sisters homozygous for a different *NMNAT2* variant (T94M) — one that largely retains enzymatic activity — also display peripheral neuropathy, albeit significantly milder than the patients described in this study (27).

Through this study, in both patients as well as the mice, it appeared that the peripheral nervous system was preferentially affected by NMNAT2 dysfunction, likely due to the longer length of peripheral axons. NMNAT2 has a very short half life and is transported from the soma to the axon, thus, distal regions are likely to be more sensitive to changes in NMNAT2 activity. Hence, the peripheral defects we observed are likely a result of axon-length-dependent SARM1 activation. The NMNAT2/SARM1 axon degeneration pathway functions in both sensory and motor neurons; yet, curiously, *Nmnat2^V98M/R232Q^* mice primarily develop a motor peripheral neuropathy. This is consistent with a *Nmnat2* hypomorphic allele that also displays peripheral motor defects (74). In contrast, in the *Nmnat2*-null mice, SARM1 drives loss of *both* sensory and motor nerves (75), begging the question as to why motor neurons are preferentially affected in *Nmnat2^V98M/R232Q^* mice. Potentially, motor neurons are more vulnerable to chronic, low-level SARM1 activation, either due to a decreased tolerance for NAD^+^ loss or to increased susceptibility to macrophage-induced degeneration. In support of this hypothesis, germline constitutively active SARM1 variants are enriched in patients with ALS (23, 24), suggesting that SARM1 activation results primarily in motor loss in vivo. Moreover, while both *Nmnat2^V98M/R232Q^* sural and femoral nerves exhibited robust SARM1 activation, there was no evidence of macrophage activation in the sural nerve. Additional study of differential motor versus sensory axon susceptibility is required to answer these fundamental questions.

Studies of many common chronic neurodegenerative disorders have implicated the immune system as a key driver of disease, and activated macrophages are major contributors to tissue damage (76). Similar to CD68^+^ microglia observed in patients with Alzheimer’s disease (77) and LPS-induced central neuroinflammation (78), CD68^+^ macrophages are abundant in the peripheral nervous system of *Nmnat2^V98M/R232Q^* mice, indicating that CD68^+^ macrophages are activated. Importantly, macrophage activation begins early in the disease, before significant axon loss has occurred, and in response to unknown signals within the neural microenvironment. We found that treating *Nmnat2^V98M/R232Q^* mice with a neuron-specific AAV expressing a SARM1-DN molecule to prevent SARM1 activation specifically in neurons prevents motor dysfunction. Altogether, these data argue that macrophage activation occurs via an extrinsic SARM1-dependent signal rather than a macrophage-autonomous program in this model.

An unexpected finding from our study was that immunodepletion of macrophages both prevents and reverses *Nmnat2-*associated motor defects. Indeed, macrophage depletion in young mice blocked the development of a motor neuropathy in *Nmnat2^V98M/R232Q^* mice. Moreover, recovery of overall strength and improved nerve electrophysiology in older, macrophage-depleted *Nmnat2^V98M/R232Q^* mice demonstrated that axon dysfunction could be reversed in the presence of persistent SARM1 activation; however, this effect lessens as the disease progresses. While all recent mechanistic progress on SARM1 has defined it as the central driver of a *cell-autonomous* degenerative program via its NAD^+^ hydrolase activity (4, 5), our data support what we believe to be a new paradigm wherein chronic axonal SARM1 activation can also orchestrate non-cell-autonomous axon degeneration. Importantly, these findings indicate that macrophages are downstream effectors of SARM1 activation in vivo, placing SARM1 at the nexus between neuroinflammation and neurodegeneration.

Examination of the macrophage polarization states in *Nmnat2^V98M/R232Q^* mice revealed a bias toward expression of Arg1, a canonical M2 signature gene. Contrary to the central tenets of M1/M2 polarization phenotypes, depletion of these Arg1^+^ macrophages in presymptomatic mice blocked rather than worsened the *Nmnat2^V98M/R232Q^* nerve pathology. We acknowledge that our work is by no means exhaustive of all genes involved in M1/M2 polarization; however, we suggest that binary polarization terms may be less meaningful in our system for describing macrophage phenotypes. Indeed, other groups have uncovered canonical markers of M1/M2 states coexpressed in individual cells after acute brain injury (68–70) and found that high Arg1 expression by microglia drives neuropathology in an AD model via immune suppression (79), underscoring the pitfalls of the existing macrophage/microglia classification framework in the nervous system.

So, how do macrophages induce axonal dysfunction in this syndrome? Evidence from studies of disease-associated microglia has established phagocytosis of live neurons, termed phagoptosis, as contributing to CNS inflammation and neurodegeneration (80). In the present study, the absence of motor defects or axon loss after 3 months of macrophage depletion in young *Nmnat2^V98M/R232Q^* mice suggests that macrophages target axons that are either functional at baseline or can recover (i.e., stressed-but-viable) in the absence of macrophage attack. Pathologic evidence in the nerves and NMJs of *Nmnat2*^V98M/R232Q^ mice points to a continual process of axon degeneration and regeneration, as has been observed in other chronic neurodegenerative diseases (81, 82). Moreover, reversal of motor deficits with macrophage depletion after disease onset suggest that there was effective and ongoing regeneration once axon degeneration was blocked.

Our study raises fundamental questions regarding the molecular crosstalk between the SARM1 degeneration pathway and the immune system. For example, what is the macrophage activation signal? Our data demonstrate that SARM1 activation was necessary but not sufficient for macrophage activation, as SARM1 activation in motor but not sensory axons induced macrophage activation. SARM1 activity drove calcium influx in damaged axons, which was followed by exposure of the phagocytic “eat-me” signal phosphatidylserine (PS) on the extracellular leaflet of the plasma membrane, and later, frank axon degeneration (83). SARM1 is required for PS exposure in response to axotomy- and vincristine-induced axon degeneration (84), and PS exposure is a driver of axon phagocytosis (85). These data suggest that phagocytic recognition of exposed PS may, indeed, be the primary mechanism for clearance of metabolically stressed-but-viable axons in vivo and that cell-autonomous axon degeneration caused by SARM1-mediated NAD^+^ depletion may only occur later as a last resort for terminally dysfunctional axons, akin to what has been observed in Drosophila (86). Further studies will be required to address the macrophage activation signal and its motor selectivity in this model.

Our data support a model wherein loss of vulnerable axons is accelerated by their engulfment by activated macrophages. Therefore, we hypothesize that macrophages are key initiators of disease in this model of a SARM1-dependent motor neuropathy, akin to their previously described roles in other common neurodegenerative diseases including MS, AD, Parkinson’s disease, and ALS (87). We predict that SARM1 activation eventually leads to neuron-autonomous axon degeneration that is not prevented by immunomodulatory therapy. Indeed, this may explain the waning rescue offered by macrophage depletion in older *Nmnat2^V98M/R232Q^* mice. The findings herein demonstrate an acute benefit for use of currently available macrophage-targeted therapy. Given that *Sarm1*-KO and SARM1-DN gene therapy both confer robust protection in this model, use of future small molecule and gene therapy SARM1 inhibitors in combination with immune targeted therapies could be optimal for long-term therapy of chronic SARMopathies.

While this study focuses on a very rare genetic cause of neuropathy, SARM1 has been implicated in an expanding number of other, more common neurodegenerative diseases including traumatic brain injury, diabetic neuropathy, chemotherapy-induced neuropathy, glaucoma, and retinal degeneration(3). Importantly, we and others have identified rare hypermorphic human *SARM1* alleles in patients with ALS (23, 24). Our work suggests that, in addition to SARM1 inhibition, these patients are also candidates for macrophage-targeted therapies. In summary, our study implies the existence of a SARM1-dependent, non-cell-autonomous pathway for axonal dysfunction that is amenable to targeted immunomodulatory therapy and presents a mechanistically defined mouse model of a pure SARMopathy in which to test such treatment strategies.

## Methods

### Whole-exome sequencing

Whole-exome sequencing and sequence analysis were performed by the Division of Genomic Diagnostics Genetic Counseling Core at Children’s Hospital of Philadelphia. Genomic DNA was extracted from peripheral blood following standard DNA extraction protocols. Targeted exons were captured with the Agilent SureSelect XT Clinical Research Exome kit per manufacturer’s protocol, sequenced on the Illumina HiSeq 2000 or 2500 platform with 100bp paired-end reads, and sequencing variants were identified using an in-house, custom-built bioinformatics pipeline as described previously (88). Mapping and analysis were based on the human genome build UCSC hg19 reference sequence. Single nucleotide variants, small deletions, and small insertions were detected. Suspected pathogenic variants were confirmed by Sanger sequencing.

### Constructs

The Takara HD InFusion Cloning Kit was used to introduce R232Q and V98M mutations into the complete open reading frame of the canonical 307 aa human *NMNAT2* allele. This was fused to a FLAG tag followed by a 10 aa linker sequence and C-terminal 2 X Strep Tag II cloned into the lentiviral vector FCIV.

### HEK293T transfection

HEK293T cells (ATCC) were cultured in DMEM with 2mM glutamine (Thermo Fisher Scientific) and 1% penicillin/streptomycin (Thermo Fisher Scientific), and 10% FBS. Cells were plated in 12-well format to reach 25%–35% confluence before transfection with polyethyleneimine (PEI, 1mg/mL, pH 7.0) using a ratio of 3:1 (PEI:plasmid DNA). 200ng StrepTag-NMNAT2-Flag expression vectors (reference allele, V98M, and R232Q) were transfected per well. 1mg/mL Cycloheximide (Sigma-Aldrich) was used to block protein synthesis 24 hours after transfection. Cells from one well per time point after treatment were suspended in 100μL of 100mM Tris-Cl (Corning), pH 8.0, and 150mM NaCl with Protease Inhibitor Cocktail (Buffer 1; Pierce), sonicated to fragment the genomic DNA, then mixed with 30μL of 4xNuPage LDS sample buffer (Invitrogen). After heating to 90°C for 5 minutes, equal amounts of each lysate were used for Western blotting.

### Western Blot

Cell lysates were resolved using SDS-PAGE on 4%–12% Bis-Tris Plus gel (Invitrogen), transferred to PVDF membrane (Invitrogen), and followed by immunoblotting for Flag (1:1,000 Mouse anti-flag M2 monoclonal, Sigma-Aldrich, F3165 ) and β-tubulin (1:1,000 anti-β-tubulin, DSHB, clone E7), and visualization by standard chemiluminescence.

### NMNAT recombinant protein expression and purification

Constructs described above, the human reference *NMNAT2* allele, and *NMNAT2* variants V98M or R232Q, were transfected into HEK293T cells in a 150 mm diameter cell culture dish. Cells were harvested 48 hours after transfection and resuspended in Buffer 1 and placed on ice during lysis by sonication. After centrifugation, the supernatant was mixed with PureCube HiCap Streptactin MagBeads (Cube Biotech) for 1 hour. Proteins were eluted with Buffer 1 plus 5 mM desthiobiotin (Sigma-Aldrich) and stored at –80°C. Concentrations were determined by the Bio-Rad protein assay. Protein purity was assessed by 4%–12% SDS-PAGE and Coomassie staining.

### NMNAT activity assay

NMNAT activity was defined as 1 mmol NAD^+^ generated per minute per mg of protein. The reaction was initiated by mixing purified NMNAT2 protein at 1–2 mg/mL with 100 mM ATP and 100 mM NMN in 10 mM HEPES, pH 7.4, and 5 mM MgCl_2_ at 37°C. The reactions were stopped by removing 100 μL from the reaction and mixing it with 100 μL 0.5 M perchloric acid (HClO_4_) on ice for 10 minutes. After centrifugation, the supernatant was mixed with 11 μL 3 M K_2_CO_3_ for neutralization. Samples were placed on ice for another 10 minutes and centrifuged. 45 μL of supernatant containing extracted metabolites was mixed with 5 μL 0.5 M potassium phosphate buffer and quantified by HPLC (Nexera X2) on a Kinetex column (100 × 3 mm, 2.6 μm; Phenomenex). An NAD^+^ standard (Sigma-Aldrich) was used to generate a standard curve for the quantification of NAD^+^ in the extraction. NAD^+^ production at 10 minutes, which is within the steady state, was used to calculate NMNAT activity.

### Generation of Nmnat2V98M/R232Q compound heterozygous mice

All animal experiments were performed under the direction of institutional animal study guidelines at Washington University (St. Louis, Missouri, USA). To generate *Nmnat2^V98M/R232Q^* mice via CRISPR/Cas9, guide RNAs were designed by the Washington University School of Medicine Genome Engineering & iPSC Center, and individually created gRNAs were microinjected into B6CBAF2/J pronuclei along with Cas9 protein and donor DNA oligonucleotides engineered to introduce the desired mutations by homology-directed repair. Properly mutated transgenic mice with individual mutations were confirmed by sequencing. Founder mice were initially mated to C57BL/6J mice and heterozygotes for each allele were subsequently mated together to generate compound heterozygotes. Compound heterozygotes were initially sequenced to confirm they carried both mutations, 1 from each parental chromosome (Supplemental files 1 and 2). Subsequent genotyping was performed by genomic PCR using the following primers: *Nmnat2* V98M (CCTATGCAAGATTCCTCTCAGT, ATCGTCGTGGTACCCCAA), *Nmnat2* R232Q (ACCATGATGTTGTTCTGGAAAC, GGTGCTCCAACATACTGCAT), and *Nmnat2* WT (ACCATGATGTTGTTCTGGAAAC, GTGCTCCAACACACTGCAG). Unless stated otherwise, both female and male mice were used for all experiments. *Sarm1*-KO mice were a gift from Marco Colonna in the Department of Pathology and Immunology, Washington University School of Medicine. *Sarm1*-KO mice were crossed to *Nmnat2^V98M/R232Q^* mice to produce *Nmnat2^V98M/R232Q^*; *Sarm1*-KO mice.

### Nerve structural analysis

Sciatic, sural, and femoral nerves were processed as previously described (15, 89). Sciatic nerves were embedded so that the most distal portion was sectioned. For the light microscope analysis of 400–600 nm, semithin sections were cut using Leica EM UC7 Ultramicrotome and placed onto microscopy slides. Toluidine blue staining and quantification were performed as previously described (89). All quantifications were performed by an investigator blinded to genotype.

### Tissue immunohistochemistry

#### Nervous tissue.

Six μm–thick sections of sciatic nerves and 20 μm–thick sections of spinal cord were prepared on a cryostat (Leica CM1860), mounted onto slides, and processed as previously described (15) using the following antibodies:(a) for *activated macrophages*, primary Ab CD68 (1:100, Bio-Rad, MCA1957GA) and secondary Ab anti-rat Cy3 (1:500, Jackson Immunoresearch, 112-165-143); (b) for *total macrophages*, primary Ab Iba-1 (1:500, Wako Chemicals, 019-19741) and secondary Ab anti-rabbit Alexa Fluor 488 (1:500, Invitrogen, A11034); (c) for *M1 macrophages*, primary Ab iNOS (1:50, Proteintech, 18985-1-AP) and secondary Ab anti-rabbit Alexa Fluor 647 (1:500, Invitrogen, A27040); (d) for *M2 macrophages*, primary Ab Arg1 (1:400, Proteintech, 66129-1-IG) and secondary Ab anti-mouse IgG1 (1:400, Invitrogen, A-21121); (e) for *GFP fluorescence*, rabbit anti-GFP Alexa Fluor 488 (1:200, Invitrogen, A-21311); (f) for *Motor neuron cell bodies*, primary Ab ChAT (1:100, Sigma-Aldrich, AB144P) and secondary Ab anti-rat Cy3 (1:250, Jackson Immunoresearch, 112-165-143).

Sciatic nerves were imaged with the Leica DMI 4000B confocal microscope at ×20 magnification. Spinal cords were imaged using a scheme covering 100% of the total spinal cord area with image stitching for quantification. ImageJ was used for quantification of integrated fluorescence density quantification from 3 independent sections per mouse. Motor neuron numbers were manually counted in the ventral horn using ImageJ.

#### Tibialis anterior muscle.

Ten μm-thick sections of the tibialis anterior muscle were processed as described (15) and stained with primary Ab laminin (1:100, Sigma-Aldrich, L9393) and secondary Ab anti-rabbit Cy3 (1:500, Invitrogen, A10520). Muscle fiber cross-sectional area was calculated by averaging fiber cross-sectional area from ≥ 150 muscle fibers per mouse from 3 independent magnification ×20 images.

### NMJ analysis

NMJ preparation and staining was performed as previously described (90). To analyze NMJ morphology, z-stack images were obtained. Maximal intensity projection images were analyzed to determine the volume of endplates using Imaris software. Fifteen to 20 NMJs were analyzed per mouse. NMJ occupancy was calculated as a ratio of presynaptic axonal area (SV2/2H3) to postsynaptic area. As the value approaches 1.0, the motor endplates are fully occupied by axons.

### Intraepidermal nerve fiber density quantification

Intraepidermal nerve fiber (IENF) staining and quantification were performed as previously described (15, 91). Footpads were imaged on a Leica DMI 4000B confocal microscope. IENF density was quantified as the number of PGP9.5^+^ axons that crossed the basement membrane normalized to the length of the basement membrane. IENF densities were averaged in 3 separate sections for each animal. Imaging and analysis were performed by an investigator blinded to genotype.

### Tissue metabolite measurements

Nervous tissue metabolites were extracted and measurements were acquired as previously described (89, 90).

### AAV constructs and virus injections

AAV constructs were created as previously described (45). Briefly, AAV8-hSYN-SARM1-DN-EGFP was generated by the viral vector core of the Hope Center for Neurological Disorders at Washington University in St. Louis. Under light anesthesia with Avertin, 6 × 10^11^ viral genomes were injected intrathecally at L6/S1. Viral expression was confirmed by detecting EGFP expression via immunohistochemical analysis of spinal cords.

### Nerve electrophysiology

CMAPs were acquired as previously described (92) using a Viking Quest electromyography device (Nicolet). Supramaximal stimulation was used for CMAPs. Sensory nerve action potentials (SNAPs) were acquired as previously described (15) using a Viking Quest electromyography device (Nicolet). Supramaximal stimulation was used for SNAPs.

### Behavioral tests

#### Tail flick assay.

The tail flick assay was performed as previously described (93). Briefly, mice were restrained horizontally, and the tips of their tails were submerged in a 55°C water bath. Latency to withdraw from the water bath was measured using a stopwatch. Five trials were performed and averaged for each mouse.

#### Inverted screen assay.

The inverted screen assay was performed as previously described with minor modifications (94). Mice were placed on a wire mesh screen. Latency to fall for each mouse was recorded, and each mouse underwent 3 trials with 5-minute rest periods. If a mouse did not fall off the mesh screen within 120 seconds, then that time was recorded, and the mouse was taken off the screen.

#### Hindlimb grip strength.

Hindlimb grip strength was measured using a computerized grip strength meter. The maximum force of each measurement was measured and recorded by the grip strength meter. Each mouse underwent 5 trials. If a mouse could not establish a grip on the bar, then the value for that trial was recorded as 0.

### Flow cytometry

Nerves were collected and kept on ice until dissociation. Nerves were then minced and incubated with gentle shaking for 20 minutes in digestion media containing 0.3% collagenase IV, 0.04% hyaluronidase, and 0.04% DNase in DMEM at 37°C. Cells were then washed and filtered through 70 μm strainers. Dead cells were excluded by propidium iodide (PI). Antibodies to the following proteins were used: CD11b (BioLegend, clone M1/70), CD45 (Thermo Fisher Scientific, clone 30-F11), and CD64 (BioLegend, clone X54-5/7.1). Cells were analyzed on an LSRII flow cytometer (BD Biosciences) and analyzed with FlowJo software.

### RNA-Seq and analysis

RNA-Seq and analysis was performed as previously described (57, 95). These data have been deposited in the Gene Expression Omnibus repository under the reference series ID GSE210403.

### CSF1R antibody injections

Macrophages were depleted by administering 1.5 mg of anti-mouse CSF1r (Bio X Cell, clone AFS98) or Rat IgG (Sigma-Aldrich, I4131) via i.p. injection every 3 weeks for a total of 4 injections.

### Macrophage activation assay

Peritoneal macrophages were harvested from individual mice by peritoneal lavage using ice-cold DMEM containing 1% FBS. Cells were then transferred into plates and subjected to either PBS or LPS treatment at a concentration of 10 ng/mL for 4 hours at 37°C.

### Statistics

Unless otherwise stated, data are reported as mean ± SEM. Between-group comparisons were made with 1-way and 2-way ANOVA with post hoc Holm-Sidák multiple comparison test or paired and unpaired 2-tailed *t* tests, as appropriate. 2-sided significance tests were used throughout and *P* < 0.05 was considered statistically significant. All statistics were calculated with the aid of GraphPad Prism 9 (https://www.graphpad.com/scientific-software/prism/) software.

### Study approval

Mice were housed and used following institutional animal study guidelines at Washington University in St. Louis. These protocols received approval from the Washington University IACUC. The patients’ parents provided written informed consent at Children’s Hospital of Philadelphia while the patients were minors. Once adults, both patients independently provided written informed consent in March 2021.

Author contributions

CBD, AJB, AD, and JM conceived the overall study. All authors contributed to the study design. CBD, AS, JZ, RES, PLW, YY, and AKYY performed the experiments and analyzed the data. SWY provided and interpreted clinical and genetic information. YS provided early intellectual input. CBD wrote the manuscript and prepared all figures. AJB, AD, and JM oversaw the analysis and revised the manuscript. All authors gave final approval of the manuscript.

## Supplementary Material

Supplemental data

Supplemental data sets 1-2

Supplemental data sets 3-4

Supplemental table 1

Supplemental table 2

Supplemental video 1

## Version 1 10/26/2022

In-Press Preview

## Version 2 12/01/2022

Electronic publication
