# Supplementary material for: Macrophage depletion blocks congenital SARM1-dependent neuropathy
Source: J Clin Invest. 2022 Dec 1;132(23):e159800. doi: 10.1172/JCI159800 (PMC9711884; doi:10.1172/JCI159800)
Supplement: Supplemental data [file jci-132-159800-s110.pdf]

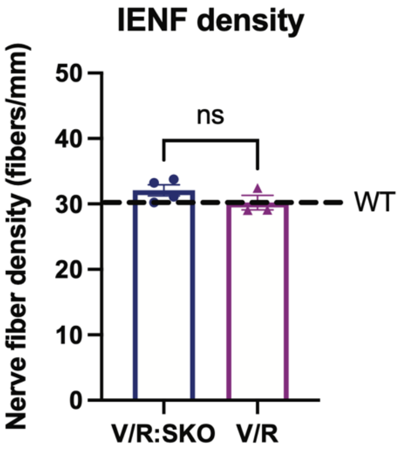

Supplemental Figure 1: Intraepidermal nerve fiber density is unaffected in *Nmnat2$^{V98M/R232Q}$* mice. Intraepidermal nerve fiber density of footpad skin of 12-month-old *Nmnat2$^{V98M/R232Q}$; Sarm1 KO* (n=4) and *Nmnat2$^{V98M/R232Q}$* (n=3) mice. All data are presented as mean ± SEM. Statistical significance was determined by Student's unpaired t-test. ns: not significant.

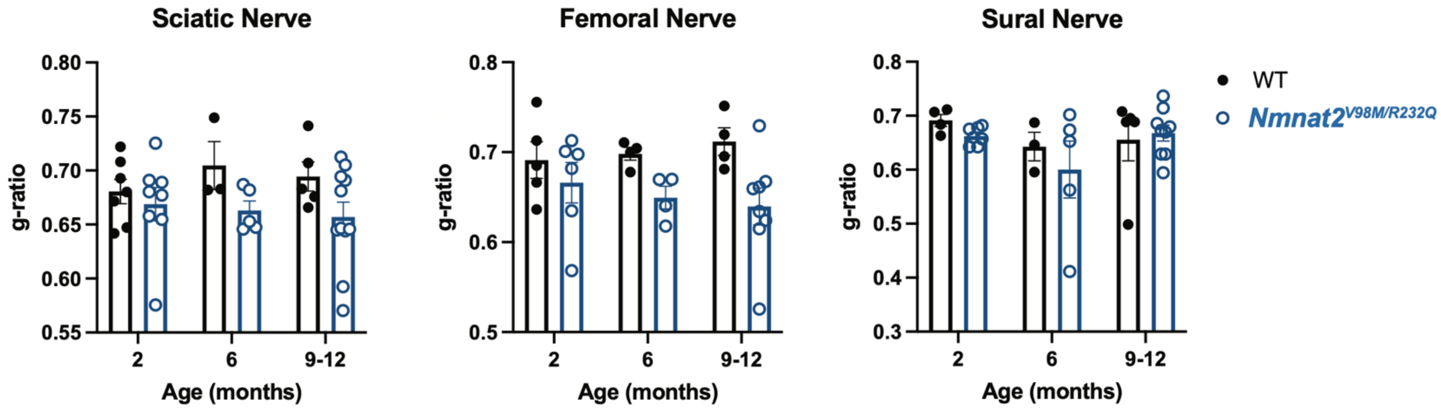

Supplemental Figure 2: Myelin thickness is preserved in *Nmnat2*$^{V98M/R232Q}$ mice. Average g-ratio in sciatic, femoral, and sural nerves at 2, 6, and 9-12 months of age, comparing *Nmnat2*$^{V98M/R232Q}$ mice (n=3-7) to WT mice (n=4-7). All data are presented as mean ± SEM.

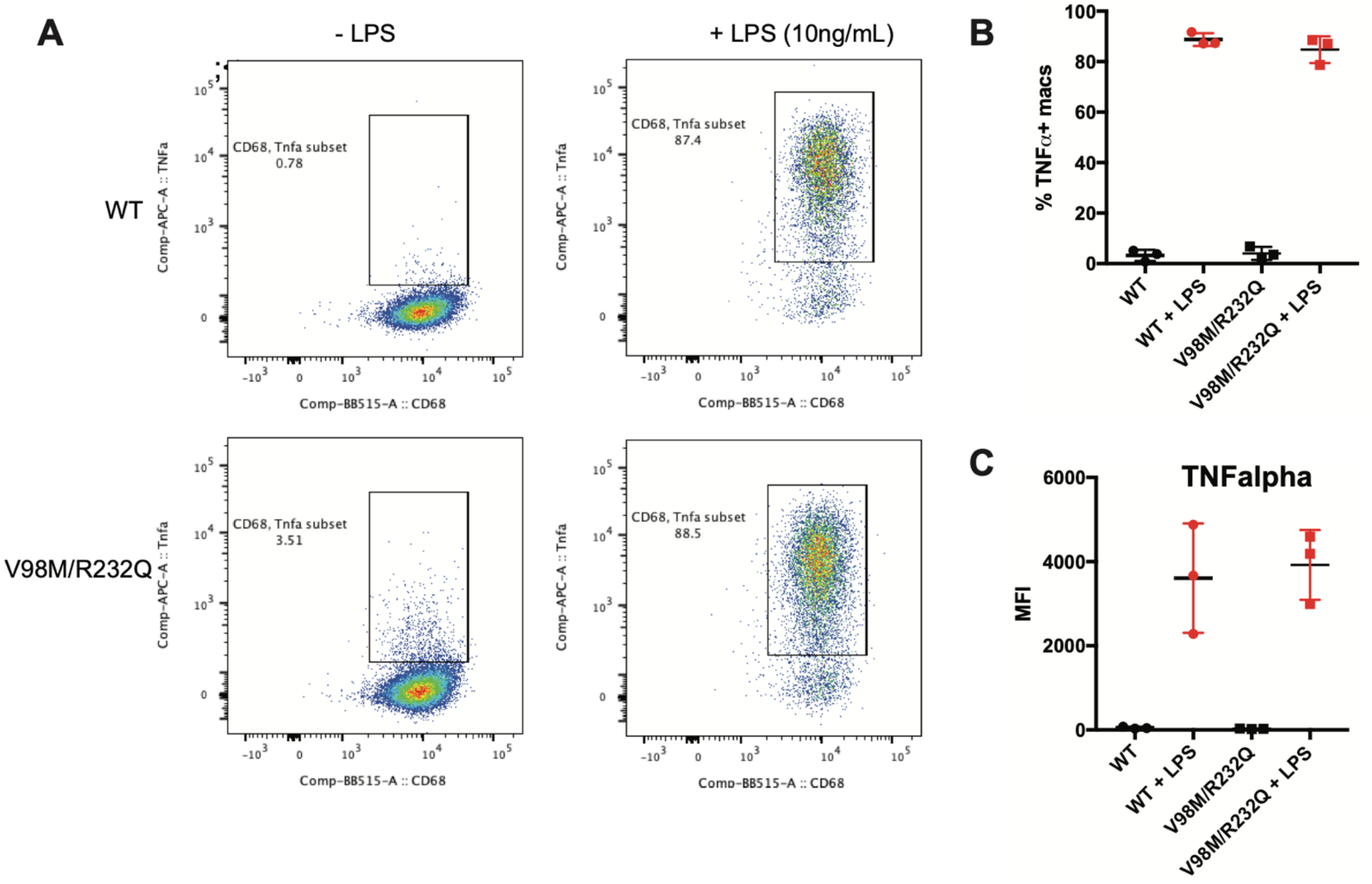

Supplemental Figure 3: Macrophage activation assay. A-B, Representative scatter plots (A) and quantification (B) of CD68$^+$ TNF-alpha$^+$ *Nmnat2*$^{V98M/R232Q}$ or WT peritoneal macrophages in the presence or absence of LPS (10ng/mL) stimulation for 4hrs (n=3). **C,** Mean fluorescence intensity (MFI) of TNF-alpha cell surface expression on CD68$^+$TNFalpha$^+$ peritoneal macrophages in the presence or absence of LPS (10ng/mL) stimulation for 4hrs. All data are presented as mean ± SEM.

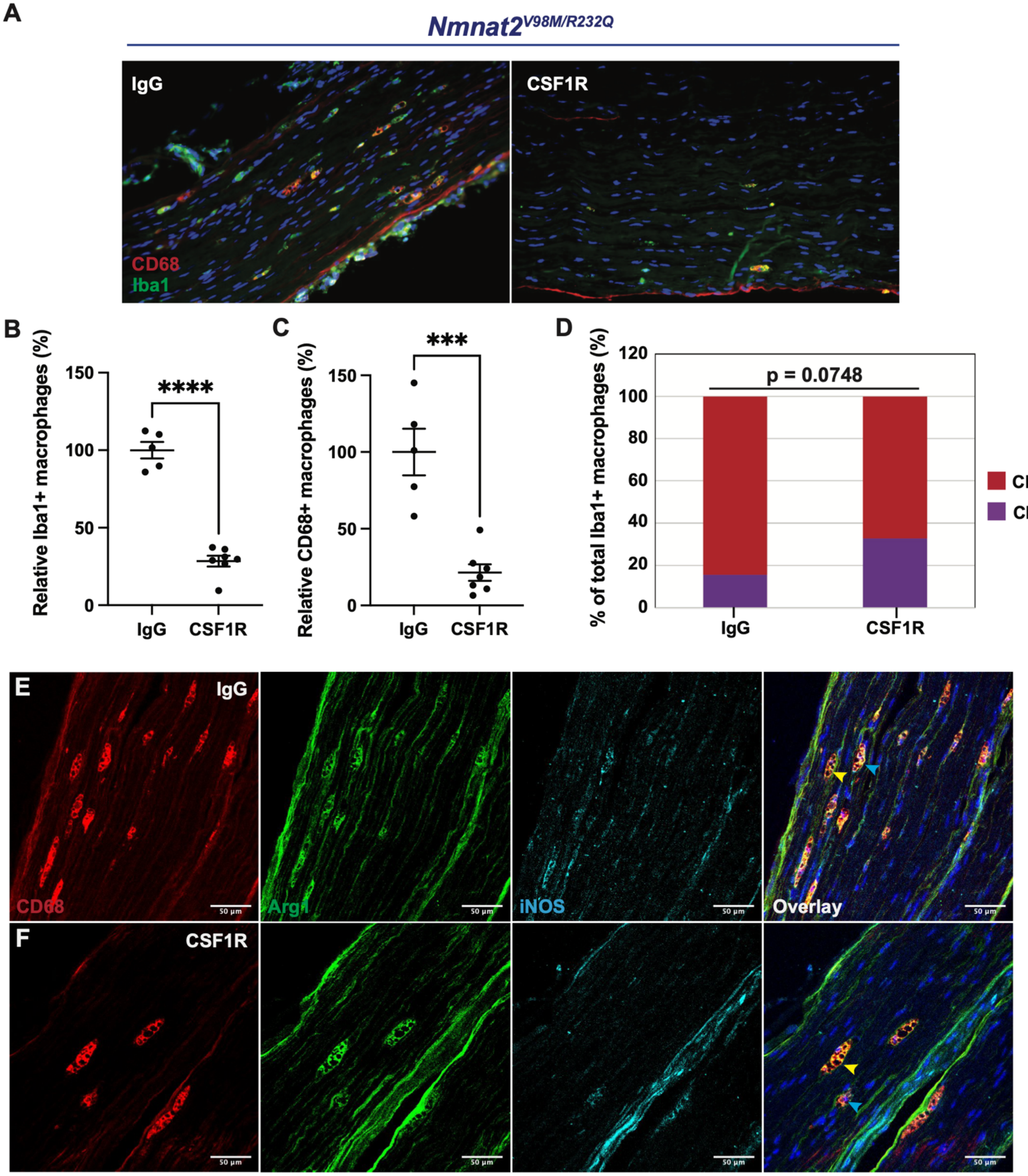

Supplemental Figure 4: **Macrophage depletion quantification. A**, Representative images of Iba1$^+$ and CD68$^+$ cells in *Nmnat2$^{V98M/R232Q}$* sciatic nerves after 3 months of IgG or CSF1R injections. **B-C**, Quantification of (B) Iba1$^+$ and (C) CD68+ macrophages in the sciatic nerves of CSF1R-injected (n=7) or IgG-injected (n=5) *Nmnat2$^{V98M/R232Q}$* mice (reported as a percentage of the IgG control). **D,** Quantification of the relative distribution of CD68$^-$ and CD68$^+$ total macrophages (Iba1+) in the sciatic nerves of CSF1R-injected (n=7) or IgG-injected (n=5) *Nmnat2$^{V98M/R232Q}$* mice. **E,** Representative images of CD68, Arg1, and iNOS immunofluorescence in in *Nmnat2$^{V98M/R232Q}$* sciatic nerves after 3 months of (E) IgG or (F) CSF1R injections. Scale bar represents 50μm. Yellow arrows indicate Arg1/CD68 co-localization and blue arrows represent Arg1/iNOS/CD68 co-localization. All data are presented as mean ± SEM. Statistical significance was determined by Student's unpaired t-test. ***p<0.001, ****p<0.0001.
