# Supplementary material for: Macrophage depletion blocks congenital SARM1-dependent neuropathy
Source: J Clin Invest. 2022 Dec 1;132(23):e159800. doi: 10.1172/JCI159800 (PMC9711884; doi:10.1172/JCI159800)
Supplement: Supplemental data sets 1-2 [file jci-132-159800-s111.pdf]

XC2119c mNmnat2 R232Q
the_Seq_start: TCACCAAAG
the_Seq_end: AGTCCTCCA
Test_Sequences:
WT_sp1: GTGGTACCCAGG
R232Q: GTGGTACCCCAA
Silent Block Mod: GTGGTACCCCGT
R232Q (Full ssODN):
CTTCCCCCCTCTCTTTAGATGGAAGTGATTGTTGGGGACTTTGGGATCGTCGTGGTACCCCAAGATGCAG
CGGACACAGACCGGATCATGAATCACTCCTCCATACTCCGCAAGTACAAAGTG
Silent Block Mod (Full ssODN):
CTTCCCCCCTCTCTTTAGATGGAAGTGATTGTTGGGGACTTTGGGATCGTCGTGGTACCCCGTGATGCAG
CGGACACAGACCGGATCATGAATCACTCCTCCATACTCCGCAAGTACAAAGTG

GEIC-Plate01-A01 TOTAL:3243 OrderedDict([('WT_sp1', 3192), ('R232Q', 0), ('Silent Block Mod', 2), ('R232Q (Full ssODN)', 0), ('Silent Block Mod (Full ssODN)', 1)])   [(0, 3228), (-1, 12), (-2, 2), (1, 1)]
TCACCAAAGTTGTTTCTCTCGCTTCCTCCTTCCCTCTCCCCCTTCCCCCCTCTCTTTAGATGGAAGTGAT
TGTTGGGGACTTTGGGATCGTCGTGGTACCCAGGGATGCAGCGGACACAGACCGGATCATGAATCACTCC
TCCATACTCCGCAAGTACAAAGTGAGTCCTCCA , 2844
TCACCAAAGTTGTTTCTCTCGCTTCCTCCTTCCCTCTCCCCCTTCCCCCCTCTCTTTAGATGGAAGTGAT
TGTTGGGGACTTTGGGATCGTCGTGGTACCCAGGGATGCAGCGGACACGGACCGGATCATGAATCACTCC
TCCATACTCCGCAAGTACAAAGTGAGTCCTCCA , 22
TCACCAAAGTTGTTTCTCTCGCTTCCTCCTTCCCTCTCCCCCTTCCCCCCTCTCTTTAGATGGAAGTGAT
TGTTGGGGACTTTGGGATCGTCGTGGTACCCAGGGATGCAGCGGACACAGACCGGATCATGAATCGCTCC
TCCATACTCCGCAAGTACAAAGTGAGTCCTCCA , 19
TCACCAAAGTTGTTTCTCTCGCTTCCTCCTTCCCTCTCCCCCTTCCCCCCTCTCTTTAGATGGAAGTGAT
TGTTGGGGACTGTGGGATCGTCGTGGTACCCAGGGATGCAGCGGACACAGACCGGATCATGAATCACTCC
TCCATACTCCGCAAGTACAAAGTGAGTCCTCCA , 14
TCACCAAAGTTGTTTCTCTCGCTTCCTCCTTCCCTCTCCCCCTTCCCCCCTCTCTTTAGATGGAAGTGAT
TGTTGGGGACTTTGGGATCGTCGTGGTACCCAGGGATGCAGCGGACACAGACCGGATCATGAATCACTCC
TCCATGCTCCGCAAGTACAAAGTGAGTCCTCCA , 5
TCACCAAAGTTGTTTCTCTCGCTTCCTCCTTCCCTCTCCCCCTTCCCCCCTCTCTTTAGATGGAAGTGAT
TGTTGGGGACGTTGGGATCGTCGTGGTACCCAGGGATGCAGCGGACACAGACCGGATCATGAATCACTCC
TCCATACTCCGCAAGTACAAAGTGAGTCCTCCA , 5
TCACCAAAGTTGTTTCTCTCGCTTCCTCCTTCCCTCTCCCCCTTCCCCCCTCTCTTTAGATGGAAGTGAT
TGTTGGGGACTTTGGGATCGTCGTGGTACCCAGGGATGCAGCGGACACAGACCAGATCATGAATCACTCC
TCCATACTCCGCAAGTACAAAGTGAGTCCTCCA , 5
TCACCAAAGTTGTTTCTCTCGCTTCCTCCTTCCCTCTCCCCCTTCCCCCCTCTCTTTAGATGGAAGTGAT
TGTTGGGGACTTTGGGATCGTCGTGGTACCCAGGGATGCAGCGGACACAGGCCGGATCATGAATCACTCC
TCCATACTCCGCAAGTACAAAGTGAGTCCTCCA , 4
TCACCAAAGTTGTTTCTCTCGCTTCCTCCTTCCCTCTCCCCCTTCCCCCCTCTCTTTAGATGGAAGTGAT
TGTTGGGGACTTTGGGATCGTCGTGGTACCCAGGGATGCAGCGGACACCGACCGGATCATGAATCACTCC
TCCATACTCCGCAAGTACAAAGTGAGTCCTCCA , 4
TCACCAAAGTTGTTTCTCTCGCTTCCTCCTTCCCTCTCCCCCTTCCCCCCTCTCTTTAGATGGAAGTGAT
TGTTGGGGACTTTGGGATCGTCGTGGTGCCCAGGGATGCAGCGGACACAGACCGGATCATGAATCACTCC
TCCATACTCCGCAAGTACAAAGTGAGTCCTCCA , 4
TCACCAAAGTTGTTTCTCTCGCTTCCTCCTTCCCTCTCCCCCTTCCCCCTCTCTTTAGATGGAAGTGATT
GTTGGGGACTTTGGGATCGTCGTGGTACCCAGGGATGCAGCGGACACAGACCGGATCATGAATCACTCCT
CCATACTCCGCAAGTACAAAGTGAGTCCTCCA , 4

TCACCAAAGTTGTTTCTCTCGCTTCCTCCTTCCCTCTCCCCCTTCCCCCCTCTCTTTAGATGGAAGTGAT
TGTTGGGGACTTTGGGATCGTCGTGGTACCCAGGGATGCAGCGGACACAGACCGGATCATGAATCACTCC
GCCATACTCCGCAAGTACAAAGTGAGTCCTCCA , 4

GEIC-Plate01-A02 TOTAL:3502 OrderedDict([('WT_sp1', 1710), ('R232Q', 0), ('Silent Block Mod', 1717), ('R232Q (Full ssODN)', 0), ('Silent Block Mod (Full ssODN)', 1575)])   [(0, 3491), (-1, 11)]
TCACCAAAGTTGTTTCTCTCGCTTCCTCCTTCCCTCTCCCCCTTCCCCCCTCTCTTTAGATGGAAGTGAT
TGTTGGGGACTTTGGGATCGTCGTGGTACCCCGTGATGCAGCGGACACAGACCGGATCATGAATCACTCC
TCCATACTCCGCAAGTACAAAGTGAGTCCTCCA , 1564
TCACCAAAGTTGTTTCTCTCGCTTCCTCCTTCCCTCTCCCCCTTCCCCCCTCTCTTTAGATGGAAGTGAT
TGTTGGGGACTTTGGGATCGTCGTGGTACCCAGGGATGCAGCGGACACAGACCGGATCATGAATCACTCC
TCCATACTCCGCAAGTACAAAGTGAGTCCTCCA , 1532
TCACCAAAGTTGTTTCTCTCGCTTCCTCCTTCCCTCTCCCCCTTCCCCCCTCTCTTTAGATGGAAGTGAT
TGTTGGGGACTTTGGGATCGTCGTGGTACCCAGGGATGCAGCGGACACGGACCGGATCATGAATCACTCC
TCCATACTCCGCAAGTACAAAGTGAGTCCTCCA , 13
TCACCAAAGTTGTTTCTCTCGCTTCCTCCTTCCCTCTCCCCCTTCCCCCCTCTCTTTAGATGGAAGTGAT
TGTTGGGGACTTTGGGATCGTCGTGGTACCCCGTGATGCAGCGGACACAGACCGGATCATGAATCGCTCC
TCCATACTCCGCAAGTACAAAGTGAGTCCTCCA , 13
TCACCAAAGTTGTTTCTCTCGCTTCCTCCTTCCCTCTCCCCCTTCCCCCCTCTCTTTAGATGGAAGTGAT
TGTTGGGGACGTTGGGATCGTCGTGGTACCCCGTGATGCAGCGGACACAGACCGGATCATGAATCACTCC
TCCATACTCCGCAAGTACAAAGTGAGTCCTCCA , 5
TCACCAAAGTTGTTTCTCTCGCTTCCTCCTTCCCTCTCCCCCTTCCCCCCTCTCTTTAGATGGAAGTGAT
TGTTGGGGACGTTGGGATCGTCGTGGTACCCAGGGATGCAGCGGACACAGACCGGATCATGAATCACTCC
TCCATACTCCGCAAGTACAAAGTGAGTCCTCCA , 5
TCACCAAAGTTGTTTCTCTCGCTTCCTCCTTCCCTCTCCCCCTTCCCCCCTCTCTTTAGATGGAAGTGAT
TGTTGGGGACTGTGGGATCGTCGTGGTACCCAGGGATGCAGCGGACACAGACCGGATCATGAATCACTCC
TCCATACTCCGCAAGTACAAAGTGAGTCCTCCA , 5
TCACCAAAGTTGTTTCTCTCGCTTCCTCCTTCCCTCTCCCCCTTCCCCCCTCTCTTTAGATGGAAGTGAT
TGTTGGGGACTTTGGGATCGTCGTGGTACCCAGTGATGCAGCGGACACAGACCGGATCATGAATCACTCC
TCCATACTCCGCAAGTACAAAGTGAGTCCTCCA , 4
TCACCAAAGTTGTTTCTCTCGCTTCCTCCTTCCCTCTCCCCCTTCCCCCCTCTCTTTAGATGGAAGTGAT
TGTTGGGGACTTTGGGATCGTCGTGGTACCCAGGGATGCAGCGGACACAGACCGGATCATGAATCACTCC
TCCATGCTCCGCAAGTACAAAGTGAGTCCTCCA , 4
TCACCAAAGTTGTTTCTCTCGCTTCCTCCTTCCCTCTCCCCCTTCCCCCCTCTCTTTAGATGGAAGTGAT
TGTTGGGGACTTTGGGATCGTCGTGGTCCCCCGTGATGCAGCGGACACAGACCGGATCATGAATCACTCC
TCCATACTCCGCAAGTACAAAGTGAGTCCTCCA , 4
TCACCAAAGTTGTTTCTCTCGCTTCCTCCTTCCCTCTCCCCCTTCCCCCCTCTCTTTAGATGGAAGTGAT
TGTTGGGGACTTTGGGATCGTCGTGGTACCCAGGGATGCAGCGGACACAGACCGGATCATGAATCGCTCC
TCCATACTCCGCAAGTACAAAGTGAGTCCTCCA , 3
TCACCAAAGTTGTTTCTCTCGCTTCCTCCTTCCCTCTCCCCCTTCCCCCCTCTCTTTAGATGGGAGTGAT
TGTTGGGGACTTTGGGATCGTCGTGGTACCCAGGGATGCAGCGGACACAGACCGGATCATGAATCACTCC
TCCATACTCCGCAAGTACAAAGTGAGTCCTCCA , 3

GEIC-Plate01-A03 TOTAL:4089 OrderedDict([('WT_sp1', 2044), ('R232Q', 1), ('Silent Block Mod', 1952), ('R232Q (Full ssODN)', 1), ('Silent Block Mod (Full ssODN)', 1759)])   [(0, 4071), (-1, 13), (1, 3), (-2, 2)]

TCACCAAAGTTGTTTCTCTCGCTTCCTCCTTCCCTCTCCCCCTTCCCCCCTCTCTTTAGATGGAAGTGAT
TGTTGGGGACTTTGGGATCGTCGTGGTACCCAGGGATGCAGCGGACACAGACCGGATCATGAATCACTCC
TCCATACTCCGCAAGTACAAAGTGAGTCCTCCA , 1850
TCACCAAAGTTGTTTCTCTCGCTTCCTCCTTCCCTCTCCCCCTTCCCCCCTCTCTTTAGATGGAAGTGAT
TGTTGGGGACTTTGGGATCGTCGTGGTACCCCGTGATGCAGCGGACACAGACCGGATCATGAATCACTCC
TCCATACTCCGCAAGTACAAAGTGAGTCCTCCA , 1740
TCACCAAAGTTGTTTCTCTCGCTTCCTCCTTCCCTCTCCCCCTTCCCCCCTCTCTTTAGATGGAAGTGAT
TGTTGGGGACTTTGGGATCGTCGTGGTACCCAGGGATGCAGCGGACACGGACCGGATCATGAATCACTCC
TCCATACTCCGCAAGTACAAAGTGAGTCCTCCA , 12
TCACCAAAGTTGTTTCTCTCGCTTCCTCCTTCCCTCTCCCCCTTCCCCCCTCTCTTTAGATGGAAGTGAT
TGTTGGGGACTGTGGGATCGTCGTGGTACCCCGTGATGCAGCGGACACAGACCGGATCATGAATCACTCC
TCCATACTCCGCAAGTACAAAGTGAGTCCTCCA , 9
TCACCAAAGTTGTTTCTCTCGCTTCCTCCTTCCCTCTCCCCCTTCCCCCCTCTCTTTAGATGGAAGTGAT
TGTTGGGGACTGTGGGATCGTCGTGGTACCCAGGGATGCAGCGGACACAGACCGGATCATGAATCACTCC
TCCATACTCCGCAAGTACAAAGTGAGTCCTCCA , 8
TCACCAAAGTTGTTTCTCTCGCTTCCTCCTTCCCTCTCCCCCTTCCCCCCTCTCTTTAGATGGAAGTGAT
TGTTGGGGACTTTGGGATCGTCGTGGTACCCCGTGATGCAGCGGACACAGACCGGATCATGAATCGCTCC
TCCATACTCCGCAAGTACAAAGTGAGTCCTCCA , 7
TCACCAAAGTTGTTTCTCTCGCTTCCTCCTTCCCTCTCCCCCTTCCCCCCTCTCTTTAGATGGAAGTGAT
TGTTGGGGACGTTGGGATCGTCGTGGTACCCCGTGATGCAGCGGACACAGACCGGATCATGAATCACTCC
TCCATACTCCGCAAGTACAAAGTGAGTCCTCCA , 7
TCACCAAAGTTGTTTCTCTCGCTTCCTCCTTCCCTCTCCCCCTTCCCCCCTCTCTTTAGATGGAAGTGAT
TGTTGGGGACTTTGGGATCGTCGTGGTACCCAGTGATGCAGCGGACACAGACCGGATCATGAATCACTCC
TCCATACTCCGCAAGTACAAAGTGAGTCCTCCA , 7
TCACCAAAGTTGTTTCTCTCGCTTCCTCCTTCCCTCTCCCCCTTCCCCCCTCTCTTTAGATGGAAGTGAT
TGTTGGGGACTTTGGGATCGTCGTGGTACCCCGTGATGCAGCGGACACAGACCGGATCATGAATCACTCC
TCCATACGCCGCAAGTACAAAGTGAGTCCTCCA , 6
TCACCAAAGTTGTTTCTCTCGCTTCCTCCTTCCCTCTCCCCCTTCCCCCCTCTCTTTAGATGGAAGTGAT
TGTTGGGGACTTTGGGATCGTCGTGGTACCCCGTGATGCAGCGGACACAGACCGGATCATGAATCACTCC
TCCATACTCTGCAAGTACAAAGTGAGTCCTCCA , 5
TCACCAAAGTTGTTTCTCTCGCTTCCTCCTTCCCTCTCCCCCTTCCCCCCTCTCTTTAGATGGAAGTGAT
TGTTGGGGACTTTGGGATCGTCGTGGTACCCAGGGATGCAGCGGACACAGACCGGATCATGAATCGCTCC
TCCATACTCCGCAAGTACAAAGTGAGTCCTCCA , 5
TCACCAAAGTTGTTTCTCTCGCTTCCTCCTTCCCTCTCCCCCTTCCCCCCTCTCTTTAGATGGAAGTGAT
TGTTGGGGACTTTGGGATCGTCGTGGTACCCAGGGATGCAGCGGACACAGACCAGATCATGAATCACTCC
TCCATACTCCGCAAGTACAAAGTGAGTCCTCCA , 5

GEIC-Plate01-A04 TOTAL:3377 OrderedDict([('WT_sp1', 3333), ('R232Q', 0), ('Silent Block Mod', 0), ('R232Q (Full ssODN)', 0), ('Silent Block Mod (Full ssODN)', 0)])   [(0, 3361), (-1, 13), (-2, 2), (1, 1)]
TCACCAAAGTTGTTTCTCTCGCTTCCTCCTTCCCTCTCCCCCTTCCCCCCTCTCTTTAGATGGAAGTGAT
TGTTGGGGACTTTGGGATCGTCGTGGTACCCAGGGATGCAGCGGACACAGACCGGATCATGAATCACTCC
TCCATACTCCGCAAGTACAAAGTGAGTCCTCCA , 2948
TCACCAAAGTTGTTTCTCTCGCTTCCTCCTTCCCTCTCCCCCTTCCCCCCTCTCTTTAGATGGAAGTGAT
TGTTGGGGACTTTGGGATCGTCGTGGTACCCAGGGATGCAGCGGACACGGACCGGATCATGAATCACTCC
TCCATACTCCGCAAGTACAAAGTGAGTCCTCCA , 14
TCACCAAAGTTGTTTCTCTCGCTTCCTCCTTCCCTCTCCCCCTTCCCCCCTCTCTTTAGATGGAAGTGAT
TGTTGGGGACTTTGGGATCGTCGTGGTACCCAGGGATGCAGCGGACACAGACCGGATCATGAATCGCTCC
TCCATACTCCGCAAGTACAAAGTGAGTCCTCCA , 12

TCACCAAAGTTGTTTCTCTCGCTTCCTCCTTCCCTCTCCCCCTTCCCCCCTCTCTTTAGATGGAAGTGAT
TGTTGGGGACGTTGGGATCGTCGTGGTACCCAGGGATGCAGCGGACACAGACCGGATCATGAATCACTCC
TCCATACTCCGCAAGTACAAAGTGAGTCCTCCA , 10
TCACCAAAGTTGTTTCTCTCGCTTCCTCCTTCCCTCTCCCCCTTCCCCCCTCTCTTTAGATGGAAGTGAT
TGTTGGGGACTGTGGGATCGTCGTGGTACCCAGGGATGCAGCGGACACAGACCGGATCATGAATCACTCC
TCCATACTCCGCAAGTACAAAGTGAGTCCTCCA , 9
TCACCAAAGTTGTTTCTCTCGCTTCCTCCTTCCCTCTCCCCCTTCCCCCCTCTCTTTAGATGGAAGTGAT
TGTTGGGGACTTTGGGATCGTCGTGGTACCCAGGGATGCAGCGGACACAGACCGGATCATGAATCACTCC
TCCATACGCCGCAAGTACAAAGTGAGTCCTCCA , 7
TCACCAAAGTTGTTTCTCTCGCTTCCTCCTTCCCTCTCCCCCTTCCCCCCTCTCTTTAGATGGAAGTGAT
TGTTGGGGACTTTGGGATCGTCGTGGTACCCAGGGATGCAGCGGACACAGGCCGGATCATGAATCACTCC
TCCATACTCCGCAAGTACAAAGTGAGTCCTCCA , 6
TCACCAAAGTTGTTTCTCTCGCTTCCTCCTTCCCTCTCCCCCTTCCCCCCTCTCTTTAGATGGAAGTGAT
TGTTGGGGACTTTGGGATCGTCGTGGTACCCAGGGATGCAGCGGACACCGACCGGATCATGAATCACTCC
TCCATACTCCGCAAGTACAAAGTGAGTCCTCCA , 6
TCACCAAAGTTGTTTCTCTCGCTTCCTCCCTCCCTCTCCCCCTTCCCCCCTCTCTTTAGATGGAAGTGAT
TGTTGGGGACTTTGGGATCGTCGTGGTACCCAGGGATGCAGCGGACACAGACCGGATCATGAATCACTCC
TCCATACTCCGCAAGTACAAAGTGAGTCCTCCA , 5
TCACCAAAGTTGTTTCTCTCGCTTCCTCCTTCCCTCTCCCCCTTCCCCCCTCTCTTTAGATGGAAGTGGT
TGTTGGGGACTTTGGGATCGTCGTGGTACCCAGGGATGCAGCGGACACAGACCGGATCATGAATCACTCC
TCCATACTCCGCAAGTACAAAGTGAGTCCTCCA , 5
TCACCAAAGTTGTTTCTCTCGCTTCCTCCTTCCCTCTCCCCCTTCCCCCCTCTCTTTAGATGGAAGTGAT
TGTTGGGGACTTTGGGATCGTCGTGGTACCCAGGGATGCAGCGGACACAGACCGGATCATGAATCACTCC
TCCATGCTCCGCAAGTACAAAGTGAGTCCTCCA , 4
TCACCAAAGTTGTTTCTCTCGCTTCCTCCTTCCCTCTCCCCCTTCCCCCCTCTCTTTAGATGGAAGTGAT
TGTTGGGGACTTTGGGATCGTCGTGGTACCCAGGGATGCAGCGGACACAGACCGGTTCATGAATCACTCC
TCCATACTCCGCAAGTACAAAGTGAGTCCTCCA , 4

GEIC-Plate01-A05 TOTAL:3458 OrderedDict([('WT_sp1', 1757), ('R232Q', 0), ('Silent Block Mod', 1607), ('R232Q (Full ssODN)', 0), ('Silent Block Mod (Full ssODN)', 1436)])   [(0, 3441), (-1, 12), (-2, 3), (1, 1), (-3, 1)]
TCACCAAAGTTGTTTCTCTCGCTTCCTCCTTCCCTCTCCCCCTTCCCCCCTCTCTTTAGATGGAAGTGAT
TGTTGGGGACTTTGGGATCGTCGTGGTACCCAGGGATGCAGCGGACACAGACCGGATCATGAATCACTCC
TCCATACTCCGCAAGTACAAAGTGAGTCCTCCA , 1534
TCACCAAAGTTGTTTCTCTCGCTTCCTCCTTCCCTCTCCCCCTTCCCCCCTCTCTTTAGATGGAAGTGAT
TGTTGGGGACTTTGGGATCGTCGTGGTACCCCGTGATGCAGCGGACACAGACCGGATCATGAATCACTCC
TCCATACTCCGCAAGTACAAAGTGAGTCCTCCA , 1419
TCACCAAAGTTGTTTCTCTCGCTTCCTCCTTCCCTCTCCCCCTTCCCCCCTCTCTTTAGATGGAAGTGAT
TGTTGGGGACTGTGGGATCGTCGTGGTACCCAGGGATGCAGCGGACACAGACCGGATCATGAATCACTCC
TCCATACTCCGCAAGTACAAAGTGAGTCCTCCA , 12
TCACCAAAGTTGTTTCTCTCGCTTCCTCCTTCCCTCTCCCCCTTCCCCCCTCTCTTTAGATGGAAGTGAT
TGTTGGGGACGTTGGGATCGTCGTGGTACCCAGGGATGCAGCGGACACAGACCGGATCATGAATCACTCC
TCCATACTCCGCAAGTACAAAGTGAGTCCTCCA , 10
TCACCAAAGTTGTTTCTCTCGCTTCCTCCTTCCCTCTCCCCCTTCCCCCCTCTCTTTAGATGGAAGTGAT
TGTTGGGGACGTTGGGATCGTCGTGGTACCCCGTGATGCAGCGGACACAGACCGGATCATGAATCACTCC
TCCATACTCCGCAAGTACAAAGTGAGTCCTCCA , 10
TCACCAAAGTTGTTTCTCTCGCTTCCTCCTTCCCTCTCCCCCTTCCCCCCTCTCTTTAGATGGAAGTGAT
TGTTGGGGACTTTGGGATCGTCGTGGTACCCCGTGATGCAGCGGACACAGACCGGATCATGAATCGCTCC

```
TCCATACTCCGCAAGTACAAAGTGAGTCCTCCA , 9
TCACCAAAGTTGTTTCTCTCGCTTCCTCCTTCCCTCTCCCCCTTCCCCCCTCTCTTTAGATGGAAGTGAT
TGTTGGGGACTTTGGGATCGTCGTGGTACCCAGGGATGCAGCGGACACGGACCGGATCATGAATCACTCC
TCCATACTCCGCAAGTACAAAGTGAGTCCTCCA , 8
TCACCAAAGTTGTTTCTCTCGCTTCCTCCTTCCCTCTCCCCCTTCCCCCCTCTCTTTAGATGGAAGTGAT
TGTTGGGGACTTTGGGGTCGTCGTGGTACCCAGGGATGCAGCGGACACAGACCGGATCATGAATCACTCC
TCCATACTCCGCAAGTACAAAGTGAGTCCTCCA , 6
TCACCAAAGTTGTTTCTCTCGCTTCCTCCTTCCCTCTCCCCCTTCCCCCCTCTCTTTAGATGGAAGTGAT
TGTTGGGGACTTTGGGATCGTCGTGGTACCCAGGGATGCAGCGGACACAGACCGGATCATGAATCGCTCC
TCCATACTCCGCAAGTACAAAGTGAGTCCTCCA , 6
TCACCAAAGTTGTTTCTCTCGCTTCCTCCTTCCCTCTCCCCCTTCCCCCCTCTCTTTAGATGGAAGTGAT
TGTTGGGGACTGTGGGATCGTCGTGGTACCCCGTGATGCAGCGGACACAGACCGGATCATGAATCACTCC
TCCATACTCCGCAAGTACAAAGTGAGTCCTCCA , 6
TCACCAAAGTTGTTTCTCTCGCTTCCTCCTTCCCTCTCCCCCTTCCCCCCTCTCTTTAGATGGAAGTGAT
TGTTGGGGACTTTGGGATCGTCGTGGTACCCCGTGATGCAGCGGACACAGACCAGATCATGAATCACTCC
TCCATACTCCGCAAGTACAAAGTGAGTCCTCCA , 5
TCACCAAAGTTGTTTCTCTCGCTTCCTCCTTCCCTCTCCCCCTTCCCCCCTCTCTTTAGATGGAAGTGAT
TGTTGGGGACTTTGGGATCGTCGTGGTCCCCAGGGATGCAGCGGACACAGACCGGATCATGAATCACTCC
TCCATACTCCGCAAGTACAAAGTGAGTCCTCCA , 5


GEIC-Plate01-A06 TOTAL:3210 OrderedDict([('WT_sp1', 1559), ('R232Q',
1), ('Silent Block Mod', 1598), ('R232Q (Full ssODN)', 1), ('Silent
Block Mod (Full ssODN)', 1452)])       [(0, 3193), (-1, 16), (1, 1)]
TCACCAAAGTTGTTTCTCTCGCTTCCTCCTTCCCTCTCCCCCTTCCCCCCTCTCTTTAGATGGAAGTGAT
TGTTGGGGACTTTGGGATCGTCGTGGTACCCCGTGATGCAGCGGACACAGACCGGATCATGAATCACTCC
TCCATACTCCGCAAGTACAAAGTGAGTCCTCCA , 1435
TCACCAAAGTTGTTTCTCTCGCTTCCTCCTTCCCTCTCCCCCTTCCCCCCTCTCTTTAGATGGAAGTGAT
TGTTGGGGACTTTGGGATCGTCGTGGTACCCAGGGATGCAGCGGACACAGACCGGATCATGAATCACTCC
TCCATACTCCGCAAGTACAAAGTGAGTCCTCCA , 1368
TCACCAAAGTTGTTTCTCTCGCTTCCTCCTTCCCTCTCCCCCTTCCCCCCTCTCTTTAGATGGAAGTGAT
TGTTGGGGACTTTGGGATCGTCGTGGTACCCAGGGATGCAGCGGACACGGACCGGATCATGAATCACTCC
TCCATACTCCGCAAGTACAAAGTGAGTCCTCCA , 11
TCACCAAAGTTGTTTCTCTCGCTTCCTCCTTCCCTCTCCCCCTTCCCCCCTCTCTTTAGATGGAAGTGAT
TGTTGGGGACTTTGGGATCGTCGTGGTACCCCGTGATGCAGCGGACACAGACCGGATCATGAATCGCTCC
TCCATACTCCGCAAGTACAAAGTGAGTCCTCCA , 8
TCACCAAAGTTGTTTCTCTCGCTTCCTCCTTCCCTCTCCCCCTTCCCCCCTCTCTTTAGATGGAAGTGAT
TGTTGGGGACGTTGGGATCGTCGTGGTACCCAGGGATGCAGCGGACACAGACCGGATCATGAATCACTCC
TCCATACTCCGCAAGTACAAAGTGAGTCCTCCA , 7
TCACCAAAGTTGTTTCTCTCGCTTCCTCCTTCCCTCTCCCCCTTCCCCCCTCTCTTTAGATGGAAGTGAT
TGTTGGGGACTGTGGGATCGTCGTGGTACCCAGGGATGCAGCGGACACAGACCGGATCATGAATCACTCC
TCCATACTCCGCAAGTACAAAGTGAGTCCTCCA , 7
TCACCAAAGTTGTTTCTCTCGCTTCCTCCTTCCCTCTCCCCCTTCCCCCCTCTCTTTAGATGGAAGTGAT
TGTTGGGGACTGTGGGATCGTCGTGGTACCCCGTGATGCAGCGGACACAGACCGGATCATGAATCACTCC
TCCATACTCCGCAAGTACAAAGTGAGTCCTCCA , 5
TCACCAAAGTTGTTTCTCTCGCTTCCTCCTTCCCTCTCCCCTTTCCCCCCTCTCTTTAGATGGAAGTGAT
TGTTGGGGACTTTGGGATCGTCGTGGTACCCAGGGATGCAGCGGACACAGACCGGATCATGAATCACTCC
TCCATACTCCGCAAGTACAAAGTGAGTCCTCCA , 5
TCACCAAAGTTGTTTCTCTCGCTTCCTCCTTCCCTCTCCCCCTTCCCCCCTCTCTTTAGATGGAAGTGAT
TGTTGGGGACTTTGGGATCGTCGTGGTACCCAGGGATGCAGCGGACACAGACCGGATCATGAATCGCTCC
```

TCCATACTCCGCAAGTACAAAGTGAGTCCTCCA , 4
TCACCAAAGTTGTTTCTCTCGCTTCCTCCTTCCCTCTCCCCCTTCCCCCCTCTCTTTAGATGGAAGTGAT
TGTTGGGGACTTTGGGATCGTCGTGGTACCCAGGGATGCAGCGGACACAGACCGGATCATGAATCACTCC
TCCATGCTCCGCAAGTACAAAGTGAGTCCTCCA , 4
TCACCAAAGTTGTTTCTCTCGCTTCCTCCTTCCCTCTCCCCCTTCCCCCCTCTCTTTAGATGGAAGTGAT
TGTTGGGGACTTTGGGATCGTCGTGGTACCCAGGGATGCAGCGGACACAGACCGGATCATGAATCACTCC
TCCATACGCCGCAAGTACAAAGTGAGTCCTCCA , 4
TCACCAAAGTTGTTTCTCTCGCTTCCTCCTTCCCTCTCCCCCTTCCCCCCTCTCTTTAGATGGAAGTGAG
TGTTGGGGACTTTGGGATCGTCGTGGTACCCAGGGATGCAGCGGACACAGACCGGATCATGAATCACTCC
TCCATACTCCGCAAGTACAAAGTGAGTCCTCCA , 4

GEIC-Plate01-A07 TOTAL:3347 OrderedDict([('WT_sp1', 1672), ('R232Q', 1), ('Silent Block Mod', 1606), ('R232Q (Full ssODN)', 1), ('Silent Block Mod (Full ssODN)', 1444)]) [(0, 3332), (-1, 13), (-37, 1), (-2, 1)]
TCACCAAAGTTGTTTCTCTCGCTTCCTCCTTCCCTCTCCCCCTTCCCCCCTCTCTTTAGATGGAAGTGAT
TGTTGGGGACTTTGGGATCGTCGTGGTACCCAGGGATGCAGCGGACACAGACCGGATCATGAATCACTCC
TCCATACTCCGCAAGTACAAAGTGAGTCCTCCA , 1458
TCACCAAAGTTGTTTCTCTCGCTTCCTCCTTCCCTCTCCCCCTTCCCCCCTCTCTTTAGATGGAAGTGAT
TGTTGGGGACTTTGGGATCGTCGTGGTACCCCGTGATGCAGCGGACACAGACCGGATCATGAATCACTCC
TCCATACTCCGCAAGTACAAAGTGAGTCCTCCA , 1429
TCACCAAAGTTGTTTCTCTCGCTTCCTCCTTCCCTCTCCCCCTTCCCCCCTCTCTTTAGATGGAAGTGAT
TGTTGGGGACTTTGGGATCGTCGTGGTACCCAGGGATGCAGCGGACACGGACCGGATCATGAATCACTCC
TCCATACTCCGCAAGTACAAAGTGAGTCCTCCA , 15
TCACCAAAGTTGTTTCTCTCGCTTCCTCCTTCCCTCTCCCCCTTCCCCCCTCTCTTTAGATGGAAGTGAT
TGTTGGGGACGTTGGGATCGTCGTGGTACCCAGGGATGCAGCGGACACAGACCGGATCATGAATCACTCC
TCCATACTCCGCAAGTACAAAGTGAGTCCTCCA , 13
TCACCAAAGTTGTTTCTCTCGCTTCCTCCTTCCCTCTCCCCCTTCCCCCCTCTCTTTAGATGGAAGTGAT
TGTTGGGGACGTTGGGATCGTCGTGGTACCCCGTGATGCAGCGGACACAGACCGGATCATGAATCACTCC
TCCATACTCCGCAAGTACAAAGTGAGTCCTCCA , 11
TCACCAAAGTTGTTTCTCTCGCTTCCTCCTTCCCTCTCCCCCTTCCCCCCTCTCTTTAGATGGAAGTGAT
TGTTGGGGACTTTGGGATCGTCGTGGTACCCCGTGATGCAGCGGACACAGACCGGATCATGAATCGCTCC
TCCATACTCCGCAAGTACAAAGTGAGTCCTCCA , 11
TCACCAAAGTTGTTTCTCTCGCTTCCTCCTTCCCTCTCCCCCTTCCCCCCTCTCTTTAGATGGAAGTGAT
TGTTGGGGACTGTGGGATCGTCGTGGTACCCCGTGATGCAGCGGACACAGACCGGATCATGAATCACTCC
TCCATACTCCGCAAGTACAAAGTGAGTCCTCCA , 8
TCACCAAAGTTGTTTCTCTCGCTTCCTCCTTCCCTCTCCCCCTTCCCCCCTCTCTTTAGATGGAAGTGAT
TGTTGGGGACTTTGGGATCGTCGTGGTACCCAGGGATGCAGCGGACACAGACCGGATCATGAATCGCTCC
TCCATACTCCGCAAGTACAAAGTGAGTCCTCCA , 6
TCACCAAAGTTGTTTCTCTCGCTTCCTCCTTCCCTCTCCCCCTTCCCCCCTCTCTTTAGATGGAAGTGAT
TGTTGGGGACTGTGGGATCGTCGTGGTACCCAGGGATGCAGCGGACACAGACCGGATCATGAATCACTCC
TCCATACTCCGCAAGTACAAAGTGAGTCCTCCA , 6
TCACCAAAGTTGTTTCTCTCGCTTCCTCCTTCCCTCTCCCCCTTCCCCCCTCTCTTTAGATGGAAGTGAT
TGTTGGGGACTTTGGGATCGTCGTGGTACCCAGTGATGCAGCGGACACAGACCGGATCATGAATCACTCC
TCCATACTCCGCAAGTACAAAGTGAGTCCTCCA , 5
TCACCAAAGTTGTTTCTCTCGCTTCCTCCTTCCCTCTCCCCCTTCCCCCCTCTCTTTAGATGGAAGTGAT
TGTTGGGGACTTTGGGATCGTCGTGGTACCCCGTGATGCAGCGGACACAGACCGGATCATGAATCACTCC
TCCATACTCCGCAAGTACCAAGTGAGTCCTCCA , 5
TCACCAAAGTTGTTTCTCTCGCTTCCTCCTTCCCTCTCCCCCTTCCCCCCTCTCTTTAGATGGAAGTGAT

TGTTGGGGACTTTGGGATCGTCGTGGTACCCCGTGATGCAGCGGACACAGACCGGATCATGAATCACTCC
TCCATACGCCGCAAGTACAAAGTGAGTCCTCCA , 5

GEIC-Plate01-A08 TOTAL:3553 OrderedDict([('WT_sp1', 3493), ('R232Q', 0), ('Silent Block Mod', 0), ('R232Q (Full ssODN)', 0), ('Silent Block Mod (Full ssODN)', 0)])   [(0, 3544), (-1, 9)]
TCACCAAAGTTGTTTCTCTCGCTTCCTCCTTCCCTCTCCCCCTTCCCCCCTCTCTTTAGATGGAAGTGAT
TGTTGGGGACTTTGGGATCGTCGTGGTACCCAGGGATGCAGCGGACACAGACCGGATCATGAATCACTCC
TCCATACTCCGCAAGTACAAAGTGAGTCCTCCA , 3092
TCACCAAAGTTGTTTCTCTCGCTTCCTCCTTCCCTCTCCCCCTTCCCCCCTCTCTTTAGATGGAAGTGAT
TGTTGGGGACTTTGGGATCGTCGTGGTACCCAGGGATGCAGCGGACACGGACCGGATCATGAATCACTCC
TCCATACTCCGCAAGTACAAAGTGAGTCCTCCA , 26
TCACCAAAGTTGTTTCTCTCGCTTCCTCCTTCCCTCTCCCCCTTCCCCCCTCTCTTTAGATGGAAGTGAT
TGTTGGGGACTTTGGGATCGTCGTGGTACCCAGGGATGCAGCGGACACAGACCGGATCATGAATCACTCC
TCCATACGCCGCAAGTACAAAGTGAGTCCTCCA , 12
TCACCAAAGTTGTTTCTCTCGCTTCCTCCTTCCCTCTCCCCCTTCCCCCCTCTCTTTAGATGGAAGTGAT
TGTTGGGGACTTTGGGATCGTCGTGGTACCCAGGGATGCAGCGGACACAGACCGGATCATGAATCGCTCC
TCCATACTCCGCAAGTACAAAGTGAGTCCTCCA , 11
TCACCAAAGTTGTTTCTCTCGCTTCCTCCTTCCCTCTCCCCCTTCCCCCCTCTCTTTAGATGGAAGTGAT
TGTTGGGGACTTTGGGATCGTCGTGGTACCCAGGGATGCAGCGGACACAGACCGGATCATGAATCACTCC
TCCATGCTCCGCAAGTACAAAGTGAGTCCTCCA , 8
TCACCAAAGTTGTTTCTCTCGCTTCCTCCTTCCCTCTCCCCCTTCCCCCCTCTCTTTAGATGGAAGTGAT
TGTTGGGGACTGTGGGATCGTCGTGGTACCCAGGGATGCAGCGGACACAGACCGGATCATGAATCACTCC
TCCATACTCCGCAAGTACAAAGTGAGTCCTCCA , 8
TCACCAAAGTTGTTTCTCTCGCTTCCTCCTTCCCTCTCCCCCTTCCCCCCTCTCTTTAGATGGAAGTGAT
TGTTGGGGACGTTGGGATCGTCGTGGTACCCAGGGATGCAGCGGACACAGACCGGATCATGAATCACTCC
TCCATACTCCGCAAGTACAAAGTGAGTCCTCCA , 7
TCACCAAAGTTGTTTCTCTCGCTTCCTCCTTCCCTCTCCCCCTTCCCCCCTCTCTTTAGATGGAAGTGAT
TGTTGGGGACTTTGGGATCGTCGTGGTACCCAGGGATGCAGCGGACACAGACCGGATCATGAATCACTCC
GCCATACTCCGCAAGTACAAAGTGAGTCCTCCA , 7
TCACCAAAGTTGTTTCTCTCGCTTCCTCCTTCCCTCTCCCCCTTCCCCCCTCTCTTTAGATGGAAGTGAT
TGTTGGGGACTTTGGGATCGTCGTGGTACCCAGGGATGCAGCGGACACCGACCGGATCATGAATCACTCC
TCCATACTCCGCAAGTACAAAGTGAGTCCTCCA , 6
TCACCAAAGTTGTTTCTCTCGCTTCCTCCTTCCCTCTCCCCCTTCCCCCCTCTCTTTAGATGGAAGTGAT
TGTTGGAGACTTTGGGATCGTCGTGGTACCCAGGGATGCAGCGGACACAGACCGGATCATGAATCACTCC
TCCATACTCCGCAAGTACAAAGTGAGTCCTCCA , 4
TCACCAAAGTTGTTTCTCTCGCTTCCTCCTTCCCTCTCCCCCTTCCCCCCTCTCTTTAGATGGAAGTGAT
TGTTGGGGACTTTGGGATCGTCGTGGTACCCAGGGATGCAGCGGACACAGACCGGATCATGAATCACGCC
TCCATACTCCGCAAGTACAAAGTGAGTCCTCCA , 4
TCACCAAAGTTGTTTCTCTCGCTTCCTCCTTCCCTCTCCCCCTTCCCCCCTCTCTTTAGATGGAAGTGAT
TGTTGGGGACTTTGGGATCGTCGTGGTGCCCAGGGATGCAGCGGACACAGACCGGATCATGAATCACTCC
TCCATACTCCGCAAGTACAAAGTGAGTCCTCCA , 4

GEIC-Plate01-B01 TOTAL:3985 OrderedDict([('WT_sp1', 3922), ('R232Q', 0), ('Silent Block Mod', 0), ('R232Q (Full ssODN)', 0), ('Silent Block Mod (Full ssODN)', 0)])   [(0, 3964), (-1, 18), (1, 2), (-3, 1)]
TCACCAAAGTTGTTTCTCTCGCTTCCTCCTTCCCTCTCCCCCTTCCCCCCTCTCTTTAGATGGAAGTGAT
TGTTGGGGACTTTGGGATCGTCGTGGTACCCAGGGATGCAGCGGACACAGACCGGATCATGAATCACTCC

TCCATACTCCGCAAGTACAAAGTGAGTCCTCCA , 3483
TCACCAAAGTTGTTTCTCTCGCTTCCTCCTTCCCTCTCCCCCTTCCCCCCTCTCTTTAGATGGAAGTGAT
TGTTGGGGACTTTGGGATCGTCGTGGTACCCAGGGATGCAGCGGACACGGACCGGATCATGAATCACTCC
TCCATACTCCGCAAGTACAAAGTGAGTCCTCCA , 25
TCACCAAAGTTGTTTCTCTCGCTTCCTCCTTCCCTCTCCCCCTTCCCCCCTCTCTTTAGATGGAAGTGAT
TGTTGGGGACTGTGGGATCGTCGTGGTACCCAGGGATGCAGCGGACACAGACCGGATCATGAATCACTCC
TCCATACTCCGCAAGTACAAAGTGAGTCCTCCA , 13
TCACCAAAGTTGTTTCTCTCGCTTCCTCCTTCCCTCTCCCCCTTCCCCCCTCTCTTTAGATGGAAGTGAT
TGTTGGGGACTTTGGGATCGTCGTGGTACCCAGGGATGCAGCGGACACAGACCGGATCATGAATCGCTCC
TCCATACTCCGCAAGTACAAAGTGAGTCCTCCA , 12
TCACCAAAGTTGTTTCTCTCGCTTCCTCCTTCCCTCTCCCCCTTCCCCCCTCTCTTTAGATGGAAGTGAT
TGTTGGGGACTTTGGGATCGTCGTGGTACCCAGGGATGCAGCGGACACAGACCGGATCATGAATCACTCC
TCCATGCTCCGCAAGTACAAAGTGAGTCCTCCA , 11
TCACCAAAGTTGTTTCTCTCGCTTCCTCCTTCCCTCTCCCCCTTCCCCCCTCTCTTTAGATGGAAGTGAT
TGTTGGGGACTTTGGGATCGTCGTGGTACCCAGGGATGCAGCGGACACCGACCGGATCATGAATCACTCC
TCCATACTCCGCAAGTACAAAGTGAGTCCTCCA , 9
TCACCAAAGTTGTTTCTCTCGCTTCCTCCTTCCCTCTCCCCCTTCCCCCCTCTCTTTAGATGGAAGTGAT
TGTTGGGGACTTTGGGATCGTCGTGGTACCCAGGGATGCAGCGGACACAGACCGGATCATGAATCACTCC
TCCATACGCCGCAAGTACAAAGTGAGTCCTCCA , 6
TCACCAAAGTTGTTTCTCTCGCTTCCTCCTTCCCTCTCCCCCTTCCCCCCTCTCTTTAGATGGAAGTGAT
TGTTGGGGACTTTGGGATCGTCGTGGTACCCAGGGATGCAGCGGACACAGACCAGATCATGAATCACTCC
TCCATACTCCGCAAGTACAAAGTGAGTCCTCCA , 6
TCACCAAAGTTGTTTCTCTCGCTTCCTCCTTCCCTCTCCCCCTTCCCCCCTCTCTTTAGATGGAAGTGAT
TGTTGGGGACTTTGGGATCGTCGTGGTACCCAGGGATGCAGCGGACACAGACCGGATCATGAATCACTCC
TCCATACTCCGCAAGTACCAAGTGAGTCCTCCA , 6
TCACCAAAGTTGTTTCTCTCGCTTCCTCCTTCCCTCTCCCCCTTCCCCCCTCTCTTTAGATGGAAGTGAT
TGTTGGGGACTTTGGGATCGTCGTGGTACCCAGTGATGCAGCGGACACAGACCGGATCATGAATCACTCC
TCCATACTCCGCAAGTACAAAGTGAGTCCTCCA , 4
TCACCAAAGTTGTTTCTCTCGCTTCCTCCTTCCCTCTCCCCCTTCCCCCTCTCTTTAGATGGAAGTGATT
GTTGGGGACTTTGGGATCGTCGTGGTACCCAGGGATGCAGCGGACACAGACCGGATCATGAATCACTCCT
CCATACTCCGCAAGTACAAAGTGAGTCCTCCA , 4
TCACCAAAGTTGTTTCTCTCGCTTCCTCCTTCCCTCTCCCCCTTCCCCCCTCTCTTTAGATGGAAGTGAG
TGTTGGGGACTTTGGGATCGTCGTGGTACCCAGGGATGCAGCGGACACAGACCGGATCATGAATCACTCC
TCCATACTCCGCAAGTACAAAGTGAGTCCTCCA , 4

GEIC-Plate01-B02 TOTAL:2724 OrderedDict([('WT_sp1', 1344), ('R232Q', 0), ('Silent Block Mod', 1323), ('R232Q (Full ssODN)', 0), ('Silent Block Mod (Full ssODN)', 1230)])   [(0, 2715), (-1, 8), (1, 1)]
TCACCAAAGTTGTTTCTCTCGCTTCCTCCTTCCCTCTCCCCCTTCCCCCCTCTCTTTAGATGGAAGTGAT
TGTTGGGGACTTTGGGATCGTCGTGGTACCCCGTGATGCAGCGGACACAGACCGGATCATGAATCACTCC
TCCATACTCCGCAAGTACAAAGTGAGTCCTCCA , 1217
TCACCAAAGTTGTTTCTCTCGCTTCCTCCTTCCCTCTCCCCCTTCCCCCCTCTCTTTAGATGGAAGTGAT
TGTTGGGGACTTTGGGATCGTCGTGGTACCCAGGGATGCAGCGGACACAGACCGGATCATGAATCACTCC
TCCATACTCCGCAAGTACAAAGTGAGTCCTCCA , 1200
TCACCAAAGTTGTTTCTCTCGCTTCCTCCTTCCCTCTCCCCCTTCCCCCCTCTCTTTAGATGGAAGTGAT
TGTTGGGGACTGTGGGATCGTCGTGGTACCCCGTGATGCAGCGGACACAGACCGGATCATGAATCACTCC
TCCATACTCCGCAAGTACAAAGTGAGTCCTCCA , 7
TCACCAAAGTTGTTTCTCTCGCTTCCTCCTTCCCTCTCCCCCTTCCCCCCTCTCTTTAGATGGAAGTGAT
TGTTGGGGACTTTGGGATCGTCGTGGTACCCAGGGATGCAGCGGACACGGACCGGATCATGAATCACTCC

TCCATACTCCGCAAGTACAAAGTGAGTCCTCCA , 6
TCACCAAAGTTGTTTCTCTCGCTTCCTCCTTCCCTCTCCCCCTTCCCCCCTCTCTTTAGATGGAAGTGAT
TGTTGGGGACTGTGGGATCGTCGTGGTACCCAGGGATGCAGCGGACACAGACCGGATCATGAATCACTCC
TCCATACTCCGCAAGTACAAAGTGAGTCCTCCA , 6
TCACCAAAGTTGTTTCTCTCGCTTCCTCCTTCCCTCTCCCCCTTCCCCCCTCTCTTTAGATGGAAGTGAT
TGTTGGGGACTTTGGGATCGTCGTGGTCCCCCGTGATGCAGCGGACACAGACCGGATCATGAATCACTCC
TCCATACTCCGCAAGTACAAAGTGAGTCCTCCA , 6
TCACCAAAGTTGTTTCTCTCGCTTCCTCCTTCCCTCTCCCCCTTCCCCCTTCTCTTTAGATGGAAGTGAT
TGTTGGGGACTTTGGGATCGTCGTGGTACCCCGTGATGCAGCGGACACAGACCGGATCATGAATCACTCC
TCCATACTCCGCAAGTACAAAGTGAGTCCTCCA , 5
TCACCAAAGTTGTTTCTCTCGCTTCCTCCTTCCCTCTCCCCCTTCCCCCCTCTCTTTAGATGGAAGTGAT
TGTTGGGGACTTTGGGATCGTCGTGGTACCCAGTGATGCAGCGGACACAGACCGGATCATGAATCACTCC
TCCATACTCCGCAAGTACAAAGTGAGTCCTCCA , 4
TCACCAAAGTTGTTTCTCTCGCTTCCTCCTTCCCTCTCCCCCTTCCCCCCTCTCTTTAGATGGAAGTGAT
TGTTGGGGACTTTGGGATCGTCGTGGTACCTCGTGATGCAGCGGACACAGACCGGATCATGAATCACTCC
TCCATACTCCGCAAGTACAAAGTGAGTCCTCCA , 4
TCACCAAAGTTGTTTCTCTCGCTTCCTCCTTCCCTCTCCCCCTTCCCCCCTCTCTTTAGATGGAAGTGAT
TGTTGGGGACTTTGGGATCGTCGTGGTACCCCGTGATGCAGCGGACACAGACCGGATCATGAATCGCTCC
TCCATACTCCGCAAGTACAAAGTGAGTCCTCCA , 4
TCACCAAAGTTGTTTCTCTCGCTTCCTCCTTCCCTCTCCCCCTTCCCCCCTCTCTTTAGATGGAAGTGAT
TGTTGGGGACTTTGGGATCGTCGTGGTACCCCGTGATGCAGCGGACACAGACCGGATCACGAATCACTCC
TCCATACTCCGCAAGTACAAAGTGAGTCCTCCA , 3
TCACCAAAGTTGTTTCTCTCGCTTCCTCCTTCCCTCTCCCCCTTCCCCCCTCTCTTTAGATGGAAGTGAT
TGTTGGGGACTTTGGGATCGTCGTGGTACCCAGGGATGCAGCGGACACAGACCAGATCATGAATCACTCC
TCCATACTCCGCAAGTACAAAGTGAGTCCTCCA , 3

GEIC-Plate01-B03 TOTAL:3996 OrderedDict([('WT_sp1', 3914), ('R232Q', 0), ('Silent Block Mod', 0), ('R232Q (Full ssODN)', 0), ('Silent Block Mod (Full ssODN)', 0)])   [(0, 3981), (-1, 12), (-2, 2), (1, 1)]
TCACCAAAGTTGTTTCTCTCGCTTCCTCCTTCCCTCTCCCCCTTCCCCCCTCTCTTTAGATGGAAGTGAT
TGTTGGGGACTTTGGGATCGTCGTGGTACCCAGGGATGCAGCGGACACAGACCGGATCATGAATCACTCC
TCCATACTCCGCAAGTACAAAGTGAGTCCTCCA , 3449
TCACCAAAGTTGTTTCTCTCGCTTCCTCCTTCCCTCTCCCCCTTCCCCCCTCTCTTTAGATGGAAGTGAT
TGTTGGGGACTTTGGGATCGTCGTGGTACCCAGGGATGCAGCGGACACGGACCGGATCATGAATCACTCC
TCCATACTCCGCAAGTACAAAGTGAGTCCTCCA , 26
TCACCAAAGTTGTTTCTCTCGCTTCCTCCTTCCCTCTCCCCCTTCCCCCCTCTCTTTAGATGGAAGTGAT
TGTTGGGGACTGTGGGATCGTCGTGGTACCCAGGGATGCAGCGGACACAGACCGGATCATGAATCACTCC
TCCATACTCCGCAAGTACAAAGTGAGTCCTCCA , 15
TCACCAAAGTTGTTTCTCTCGCTTCCTCCTTCCCTCTCCCCCTTCCCCCCTCTCTTTAGATGGAAGTGAT
TGTTGGGGACGTTGGGATCGTCGTGGTACCCAGGGATGCAGCGGACACAGACCGGATCATGAATCACTCC
TCCATACTCCGCAAGTACAAAGTGAGTCCTCCA , 13
TCACCAAAGTTGTTTCTCTCGCTTCCTCCTTCCCTCTCCCCCTTCCCCCCTCTCTTTAGATGGAAGTGAT
TGTTGGGGACTTTGGGATCGTCGTGGTACCCAGGGATGCAGCGGACACAGACCGGATCATGAATCGCTCC
TCCATACTCCGCAAGTACAAAGTGAGTCCTCCA , 9
TCACCAAAGTTGTTTCTCTCGCTTCCTCCTTCCCTCTCCCCCTTCCCCCCTCTCTTTAGATGGAAGTGAT
TGTTGGGGACTTTGGGATCGTCGTGGTACCCAGGGATGCAGCGGACACCGACCGGATCATGAATCACTCC
TCCATACTCCGCAAGTACAAAGTGAGTCCTCCA , 6
TCACCAAAGTTGTTTCTCTCGCTTCCTCCTTCCCTCTCCCCCTTCCCCCCTCTCTTTAGATGGAAGTGAT
TGTTGGGGACTTTGGGATCGTCGTGGTACCCAGGGATGCAGCGGACACAGACCGGATCATGAATCACTCC

TCCATACGCCGCAAGTACAAAGTGAGTCCTCCA , 6
TCACCAAAGTTGTTTCTCTCGCTTCCTCCTTCCCTCTCCCCCTTCCCCCCTCTCTTTAGATGGAAGTGAT
TGTTGGGGACTTTGGGATCGTCGTGGTAACCAGGGATGCAGCGGACACAGACCGGATCATGAATCACTCC
TCCATACTCCGCAAGTACAAAGTGAGTCCTCCA , 5
TCACCAAAGTTGTTTCTCTCGCTTCCTCCTTCCCTCTCCCCCTTCCCCCCTCTCTTTAGATGGAAGTGAT
TGTTGGGGACTTTGGGATCGTCGTGGTACCCAGGGATGCAGCGGACACAGACCGGATCATGAATCACTCC
GCCATACTCCGCAAGTACAAAGTGAGTCCTCCA , 5
TCACCAAAGTTGTTTCTCTCGCTTCCTCCTTCCCTCTCCCCCTTCCCCCCTCTCTTTAGATGGAAGTGAT
TGTTGGGGACTTTGGGATCGTCGTGGTACCCAGGGATGCAGCGGGCACAGACCGGATCATGAATCACTCC
TCCATACTCCGCAAGTACAAAGTGAGTCCTCCA , 5
TCACCAAAGTTGTTTCTCTCACTTCCTCCTTCCCTCTCCCCCTTCCCCCCTCTCTTTAGATGGAAGTGAT
TGTTGGGGACTTTGGGATCGTCGTGGTACCCAGGGATGCAGCGGACACAGACCGGATCATGAATCACTCC
TCCATACTCCGCAAGTACAAAGTGAGTCCTCCA , 4
TCACCAAAGTTGTTTCTCTCGCTTCCTCCTTCCCTCTCCCCCTTCCCCCTTCTCTTTAGATGGAAGTGAT
TGTTGGGGACTTTGGGATCGTCGTGGTACCCAGGGATGCAGCGGACACAGACCGGATCATGAATCACTCC
TCCATACTCCGCAAGTACAAAGTGAGTCCTCCA , 4

GEIC-Plate01-B04 TOTAL:4076 OrderedDict([('WT_sp1', 2041), ('R232Q', 0), ('Silent Block Mod', 1976), ('R232Q (Full ssODN)', 0), ('Silent Block Mod (Full ssODN)', 1842)])   [(0, 4064), (-1, 9), (1, 3)]
TCACCAAAGTTGTTTCTCTCGCTTCCTCCTTCCCTCTCCCCCTTCCCCCCTCTCTTTAGATGGAAGTGAT
TGTTGGGGACTTTGGGATCGTCGTGGTACCCAGGGATGCAGCGGACACAGACCGGATCATGAATCACTCC
TCCATACTCCGCAAGTACAAAGTGAGTCCTCCA , 1885
TCACCAAAGTTGTTTCTCTCGCTTCCTCCTTCCCTCTCCCCCTTCCCCCCTCTCTTTAGATGGAAGTGAT
TGTTGGGGACTTTGGGATCGTCGTGGTACCCCGTGATGCAGCGGACACAGACCGGATCATGAATCACTCC
TCCATACTCCGCAAGTACAAAGTGAGTCCTCCA , 1816
TCACCAAAGTTGTTTCTCTCGCTTCCTCCTTCCCTCTCCCCCTTCCCCCCTCTCTTTAGATGGAAGTGAT
TGTTGGGGACTGTGGGATCGTCGTGGTACCCAGGGATGCAGCGGACACAGACCGGATCATGAATCACTCC
TCCATACTCCGCAAGTACAAAGTGAGTCCTCCA , 10
TCACCAAAGTTGTTTCTCTCGCTTCCTCCTTCCCTCTCCCCCTTCCCCCCTCTCTTTAGATGGAAGTGAT
TGTTGGGGACTTTGGGATCGTCGTGGTACCCAGGGATGCAGCGGACACGGACCGGATCATGAATCACTCC
TCCATACTCCGCAAGTACAAAGTGAGTCCTCCA , 7
TCACCAAAGTTGTTTCTCTCGCTTCCTCCTTCCCTCTCCCCCTTCCCCCCTCTCTTTAGATGGAAGTGAT
TGTTGGGGACTGTGGGATCGTCGTGGTACCCCGTGATGCAGCGGACACAGACCGGATCATGAATCACTCC
TCCATACTCCGCAAGTACAAAGTGAGTCCTCCA , 6
TCACCAAAGTTGTTTCTCTCGCTTCCTCCTTCCCTCTCCCCCTTCCCCCCTCTCTTTAGATGGAAGTGAT
TGTTGGGGACTTTGGGATCGTCGTGGTACCCCGTGATGCAGCGGACACAGACCGGATCATGAATCGCTCC
TCCATACTCCGCAAGTACAAAGTGAGTCCTCCA , 6
TCACCAAAGTTGTTTCTCTCGCTTCCTCCTTCCCTCTCCCCCTTCCCCCCTCTCTTTAGATGGAAGTGAT
TGTTGGGGACGTTGGGATCGTCGTGGTACCCAGGGATGCAGCGGACACAGACCGGATCATGAATCACTCC
TCCATACTCCGCAAGTACAAAGTGAGTCCTCCA , 5
TCACCAAAGTTGTTTCTCTCGCTTCCTCCTTCCCTCTCCCCCTTCCCCCCTCTCTTTAGATGGAAGTGAT
TGTTGGGGACTTTGGGATCGTCGTGGTACCCAGGGATGCAGCGGACACAGACCGGATCATGAATCACTCC
TCCATGCTCCGCAAGTACAAAGTGAGTCCTCCA , 4
TCACCAAAGTTGTTTCTCTCGCTTCCTCCTTCCCTCTCCCCCTTCCCCCCTCTCTTTAGATGGAAGTGAT
TGTTGGGGACTTTGGGATCGTCGTGGTACCCAGGGATGCAGCGGACACAGACCGGATCATGAATCGCTCC
TCCATACTCCGCAAGTACAAAGTGAGTCCTCCA , 4
TCACCAAAGTTGTTTCTCTCGCTTCCTCCTTCCCTCTCCCCCTTCCCCCCTCTCTTTAGATGGAAGTGAT
TGTTGGGGACTTTGGGATCGTCGTGGTCCCCAGGGATGCAGCGGACACAGACCGGATCATGAATCACTCC

TCCATACTCCGCAAGTACAAAGTGAGTCCTCCA , 4
TCACCAAAGTTGTTTCTCTCGCCTCCTCCTTCCCTCTCCCCCTTCCCCCCTCTCTTTAGATGGAAGTGAT
TGTTGGGGACTTTGGGATCGTCGTGGTACCCAGGGATGCAGCGGACACAGACCGGATCATGAATCACTCC
TCCATACTCCGCAAGTACAAAGTGAGTCCTCCA , 3
TCACCAAAGTTGTTTCTCTCGCTTCCTCCTTCCCTCTCCCCCTTCCCCCCTCTCTTTAGATGGAAGTGAT
TGTTGGGGACTTTGGGATTGTCGTGGTACCCCGTGATGCAGCGGACACAGACCGGATCATGAATCACTCC
TCCATACTCCGCAAGTACAAAGTGAGTCCTCCA , 3

GEIC-Plate01-B05 TOTAL:4184 OrderedDict([('WT_sp1', 2039), ('R232Q', 2065), ('Silent Block Mod', 0), ('R232Q (Full ssODN)', 1890), ('Silent Block Mod (Full ssODN)', 0)])   [(0, 4173), (-1, 10), (1, 1)]
TCACCAAAGTTGTTTCTCTCGCTTCCTCCTTCCCTCTCCCCCTTCCCCCCTCTCTTTAGATGGAAGTGAT
TGTTGGGGACTTTGGGATCGTCGTGGTACCCCAAGATGCAGCGGACACAGACCGGATCATGAATCACTCC
TCCATACTCCGCAAGTACAAAGTGAGTCCTCCA , 1864
TCACCAAAGTTGTTTCTCTCGCTTCCTCCTTCCCTCTCCCCCTTCCCCCCTCTCTTTAGATGGAAGTGAT
TGTTGGGGACTTTGGGATCGTCGTGGTACCCAGGGATGCAGCGGACACAGACCGGATCATGAATCACTCC
TCCATACTCCGCAAGTACAAAGTGAGTCCTCCA , 1820
TCACCAAAGTTGTTTCTCTCGCTTCCTCCTTCCCTCTCCCCCTTCCCCCCTCTCTTTAGATGGAAGTGAT
TGTTGGGGACTTTGGGATCGTCGTGGTACCCAGGGATGCAGCGGACACGGACCGGATCATGAATCACTCC
TCCATACTCCGCAAGTACAAAGTGAGTCCTCCA , 13
TCACCAAAGTTGTTTCTCTCGCTTCCTCCTTCCCTCTCCCCCTTCCCCCCTCTCTTTAGATGGAAGTGAT
TGTTGGGGACGTTGGGATCGTCGTGGTACCCCAAGATGCAGCGGACACAGACCGGATCATGAATCACTCC
TCCATACTCCGCAAGTACAAAGTGAGTCCTCCA , 9
TCACCAAAGTTGTTTCTCTCGCTTCCTCCTTCCCTCTCCCCCTTCCCCCCTCTCTTTAGATGGAAGTGAT
TGTTGGGGACTTTGGGATCGTCGTGGTACCCCAAGATGCAGCGGACACAGACCGGATCATGAATCGCTCC
TCCATACTCCGCAAGTACAAAGTGAGTCCTCCA , 9
TCACCAAAGTTGTTTCTCTCGCTTCCTCCTTCCCTCTCCCCCTTCCCCCCTCTCTTTAGATGGAAGTGAT
TGTTGGGGACTTTGGGATCGTCGTGGTACCCAGGGATGCAGCGGACACAGACCGGATCATGAATCGCTCC
TCCATACTCCGCAAGTACAAAGTGAGTCCTCCA , 8
TCACCAAAGTTGTTTCTCTCGCTTCCTCCTTCCCTCTCCCCCTTCCCCCCTCTCTTTAGATGGAAGTGAT
TGTTGGGGACTTTGGGATCGTCGTGGTACCCCAAGATGCAGCGGACACAGACCGGATCATGAATCACTCC
TCCATGCTCCGCAAGTACAAAGTGAGTCCTCCA , 5
TCACCAAAGTTGTTTCTCTCGCTTCCTCCTTCCCTCTCCCCCTTCCCCCCTCTCTTTAGATGGAAGTGAT
TGTTGGGGACTTTGGGATCGTCGTGGTACCCAGGGATGCAGCGGACACAGACCGGATCATGAATCACTCC
TCCATGCTCCGCAAGTACAAAGTGAGTCCTCCA , 5
TCACCAAAGTTGTTTCTCTCGCTTCCTCCTTCCCTCTCCCCCTTCCCCCCTCTCTTTAGATGGAAGTGAT
TGTTGGGGACTTTGGGATCGTCGTGGTACCCAGGGATGCAGCGGACACAGACCGGATCATGAATCACTCC
TCCATACTCCGCAAGTACCAAGTGAGTCCTCCA , 5
TCACCAAAGTTGTTTCTCTCGCTTCCTCCTTCCCTCTCCCCCTTCCCCCCTCTCTTTAGATGGAAGTGAT
TGTTGGGGACTTTGGGATCGTCGTGGTACCCCAAGATGCAGCGGACACAGACCGGATCATGAATCACTCC
GCCATACTCCGCAAGTACAAAGTGAGTCCTCCA , 4
TCACCAAAGTTGTTTCTCTCGCTTCCTCCTTCCCTCTCCCCCTTCCCCCCTCTCTTTAGATGGAAGTGAT
TGTTGGGGACTTTGGGATCGTCGTGGTACCCAGGGATGCAGCGGACACAGACCGGATCATGAATCACTCC
TCCATACGCCGCAAGTACAAAGTGAGTCCTCCA , 4
TCACCAAAGTTGTTTCTCTCGCTTCCTCCTTCCCTCTCCCCCTTCCCCCCTCTCTTTAGATGGAAGTGAT
TGTTGGGGACTTTGGGATCGTCGTGGTACCCAAAGATGCAGCGGACACAGACCGGATCATGAATCACTCC
TCCATACTCCGCAAGTACAAAGTGAGTCCTCCA , 4

GEIC–Plate01–B06 TOTAL:3336 OrderedDict([('WT_sp1', 1715), ('R232Q', 1548), ('Silent Block Mod', 0), ('R232Q (Full ssODN)', 1400), ('Silent Block Mod (Full ssODN)', 0)])   [(0, 3318), (–1, 17), (1, 1)]
TCACCAAAGTTGTTTCTCTCGCTTCCTCCTTCCCTCTCCCCCTTCCCCCCTCTCTTTAGATGGAAGTGAT
TGTTGGGGACTTTGGGATCGTCGTGGTACCCAGGGATGCAGCGGACACAGACCGGATCATGAATCACTCC
TCCATACTCCGCAAGTACAAAGTGAGTCCTCCA , 1512
TCACCAAAGTTGTTTCTCTCGCTTCCTCCTTCCCTCTCCCCCTTCCCCCCTCTCTTTAGATGGAAGTGAT
TGTTGGGGACTTTGGGATCGTCGTGGTACCCCAAGATGCAGCGGACACAGACCGGATCATGAATCACTCC
TCCATACTCCGCAAGTACAAAGTGAGTCCTCCA , 1384
TCACCAAAGTTGTTTCTCTCGCTTCCTCCTTCCCTCTCCCCCTTCCCCCCTCTCTTTAGATGGAAGTGAT
TGTTGGGGACTTTGGGATCGTCGTGGTACCCAGGGATGCAGCGGACACGGACCGGATCATGAATCACTCC
TCCATACTCCGCAAGTACAAAGTGAGTCCTCCA , 21
TCACCAAAGTTGTTTCTCTCGCTTCCTCCTTCCCTCTCCCCCTTCCCCCCTCTCTTTAGATGGAAGTGAT
TGTTGGGGACTTTGGGATCGTCGTGGTACCCCAAGATGCAGCGGACACAGACCGGATCATGAATCGCTCC
TCCATACTCCGCAAGTACAAAGTGAGTCCTCCA , 11
TCACCAAAGTTGTTTCTCTCGCTTCCTCCTTCCCTCTCCCCCTTCCCCCCTCTCTTTAGATGGAAGTGAT
TGTTGGGGACGTTGGGATCGTCGTGGTACCCAGGGATGCAGCGGACACAGACCGGATCATGAATCACTCC
TCCATACTCCGCAAGTACAAAGTGAGTCCTCCA , 9
TCACCAAAGTTGTTTCTCTCGCTTCCTCCTTCCCTCTCCCCCTTCCCCCCTCTCTTTAGATGGAAGTGAT
TGTTGGGGACTGTGGGATCGTCGTGGTACCCAGGGATGCAGCGGACACAGACCGGATCATGAATCACTCC
TCCATACTCCGCAAGTACAAAGTGAGTCCTCCA , 6
TCACCAAAGTTGTTTCTCTCGCTTCCTCCTTCCCTCTCCCCCTTCCCCCCTCTCTTTAGATGGAAGTGAT
TGTTGGGGACTTTGGGATCGTCGTGGTACCCAGGGATGCAGCGGACACAGACCGGATCATGAATCGCTCC
TCCATACTCCGCAAGTACAAAGTGAGTCCTCCA , 4
TCACCAAAGTTGTTTCTCTCGCTTCCTCCTTCCCTCTCCCCCTTCCCCCCTCTCTTTAGATGGAAGTGAT
TGTTGGGGACTGTGGGATCGTCGTGGTACCCCAAGATGCAGCGGACACAGACCGGATCATGAATCACTCC
TCCATACTCCGCAAGTACAAAGTGAGTCCTCCA , 4
TCACCAAAGTTGTTTCTCTCGCTTCCTCCTTCCCTCTCCCCCTTCCCCCCTCTCTTTAGATGGAAGTGAT
TGTTGGGGACTTTGGGATCGTCGTGGTACCCAGGGATGCAGCAGACACAGACCGGATCATGAATCACTCC
TCCATACTCCGCAAGTACAAAGTGAGTCCTCCA , 4
TCACCAAAGTTGTTTCTCTCGCTTCCTCCTTCCCTCTCCCCCTTCCCCCCTCTCTTTAGATGGAAGTGAT
TGTTGGGGACTTTGGGATCGTCGTGGTACCCCAAGATGCAGCGGACACAGACCGGATCATGAATCACTCC
GCCATACTCCGCAAGTACAAAGTGAGTCCTCCA , 4
TCACCAAAGTTGTTTCTCTCGCTTCCTCCTTCCCTCTCCCCCTTCCCCCCTCTCTTTAGATGGAAGTGAT
TGTTGGGGACGTTGGGATCGTCGTGGTACCCCAAGATGCAGCGGACACAGACCGGATCATGAATCACTCC
TCCATACTCCGCAAGTACAAAGTGAGTCCTCCA , 3
TCACCAAAGTTGTTTCTCTCGCTTCCTCCTTCCCTCTCCCCCTTCCCCCCTCTCTTTAGATGGAAGTGAT
TGTTGGGGACTTTGGGATCGTCGTGGTACCCCAAGATGCAGCGGACACAGACCGGATCATGAATCACTCC
TCCATACTCCGCAAGTACCAAGTGAGTCCTCCA , 3

GEIC–Plate01–B07 TOTAL:104 OrderedDict([('WT_sp1', 103), ('R232Q', 0), ('Silent Block Mod', 0), ('R232Q (Full ssODN)', 0), ('Silent Block Mod (Full ssODN)', 0)])   [(0, 103), (–1, 1)]
TCACCAAAGTTGTTTCTCTCGCTTCCTCCTTCCCTCTCCCCCTTCCCCCCTCTCTTTAGATGGAAGTGAT
TGTTGGGGACTTTGGGATCGTCGTGGTACCCAGGGATGCAGCGGACACAGACCGGATCATGAATCACTCC
TCCATACTCCGCAAGTACAAAGTGAGTCCTCCA , 96
TCACCAAAGTTGTTTCTCTCGCTTCCTCCTTCCCTCTCCCCCTTCCCCCCTCTCTTTAGATGGAAGTGAT
TGTTGGGGACTTTGGGATCGTCGTGGTACCCAGGGATGCAGCGGACACAGACCGGATCATGAAGCACTCC
TCCATACTCCGCAAGTACAAAGTGAGTCCTCCA , 1

TCACCAAAGTTGTTTCTCTCGCTTCCTCCTTCCCTCTCCCCCTCCCCCCCCTCTTTAGATGGAAGTGATT
GTTGGGGACTTTGGGATCGTCGTGGTACCCAGGGATGCAGCGGACACAGACCGGATCATGAATCACTCCT
CCATACTCCGCAAGTACAAAGTGAGTCCTCCA , 1
TCACCAAAGTTGTTTCTCTCGCTTCCTCCTTCCCTCTCCCCCTTCCCCCCTCTCTTTAGATGGAAGTGAT
TGTTGGGGACTTTGGGATCGTCGTGGTACCCAGGGATGCAGCGGACACAGACCGGATCATGAATCACTCC
TCCATACTCTGCAAGTACAAAGTGAGTCCTCCA , 1
TCACCAAAGTTGTTTCTCTCGCTTCCTCCTTCCCTCTCCCCCTTCCCCCCTCTCTTTAGATGGAAGTGGT
TGTTGGGGACTTTGGGATAGTCGGGGTACCCAGGGATGCAGCGGACACAGGCCGGATCATGAATCACTCC
TCCATACTCCGCAAGTACAAAGTGAGTCCTCCA , 1
TCACCAAAGTTGTTTCTCTCGCTTCCTCCTTCCCTCTCCCCCTTCCCCCCTCTCTTTAGATGGAAGTGAT
TGTTGGGGACTTTGGGATCGTCGTGGTACCCAGGGATGCAGCGGACACAGACCGGATCATGAATCACTCC
TCCATACTCCACAAGTACAAAGTGAGTCCTCCA , 1
TCACCAAAGTTGTTTCTCTCGCCTCCTCCTTCCCTCTCCCCCTTCCCCCCTCTCTTTAGATGGAAGTGAT
TGTTGGGGACTTTGGGATCGTCGTGGTACCCAGGGATGCAGCGGACACAGACCGGATCATGAATCACTCC
TCCATACTCCGCAAGTACAAAGTGAGTCCTCCA , 1
TCACCAAAGTTGTTTCTCTCGCTTCCTCCTTCCCTCTCCCCCTTCCCCCCTCTCTTTAGATGGAAGTGAT
TGTTGGGTACTTTGGGATCGTCGTGGTACCCAGGGATGCAGCGGACACAGACCGGATCATGAATCACTCC
TCCATACTCCGCAAGTACAAAGTGAGTCCTCCA , 1
TCACCAAAGTTGTTTCTCTCGCTTCCTCCTTCCCTCTCCCCCTTCCCCCCTCTCTTTAGATGGAAGTGAT
TGTTGGGGACTTTGGGATCGTCGTGGTACCCAGGGATGCAGCGGACACGGACCGGATCATGAATCACTCC
TCCATACTCCGCAAGTACAAAGTGAGTCCTCCA , 1

GEIC-Plate01-B08 TOTAL:4124 OrderedDict([('WT_sp1', 4050), ('R232Q', 0), ('Silent Block Mod', 0), ('R232Q (Full ssODN)', 0), ('Silent Block Mod (Full ssODN)', 0)])   [(0, 4110), (-1, 12), (1, 2)]
TCACCAAAGTTGTTTCTCTCGCTTCCTCCTTCCCTCTCCCCCTTCCCCCCTCTCTTTAGATGGAAGTGAT
TGTTGGGGACTTTGGGATCGTCGTGGTACCCAGGGATGCAGCGGACACAGACCGGATCATGAATCACTCC
TCCATACTCCGCAAGTACAAAGTGAGTCCTCCA , 3597
TCACCAAAGTTGTTTCTCTCGCTTCCTCCTTCCCTCTCCCCCTTCCCCCCTCTCTTTAGATGGAAGTGAT
TGTTGGGGACTTTGGGATCGTCGTGGTACCCAGGGATGCAGCGGACACGGACCGGATCATGAATCACTCC
TCCATACTCCGCAAGTACAAAGTGAGTCCTCCA , 18
TCACCAAAGTTGTTTCTCTCGCTTCCTCCTTCCCTCTCCCCCTTCCCCCCTCTCTTTAGATGGAAGTGAT
TGTTGGGGACGTTGGGATCGTCGTGGTACCCAGGGATGCAGCGGACACAGACCGGATCATGAATCACTCC
TCCATACTCCGCAAGTACAAAGTGAGTCCTCCA , 17
TCACCAAAGTTGTTTCTCTCGCTTCCTCCTTCCCTCTCCCCCTTCCCCCCTCTCTTTAGATGGAAGTGAT
TGTTGGGGACTGTGGGATCGTCGTGGTACCCAGGGATGCAGCGGACACAGACCGGATCATGAATCACTCC
TCCATACTCCGCAAGTACAAAGTGAGTCCTCCA , 15
TCACCAAAGTTGTTTCTCTCGCTTCCTCCTTCCCTCTCCCCCTTCCCCCCTCTCTTTAGATGGAAGTGAT
TGTTGGGGACTTTGGGATCGTCGTGGTACCCAGGGATGCAGCGGACACAGACCGGATCATGAATCGCTCC
TCCATACTCCGCAAGTACAAAGTGAGTCCTCCA , 12
TCACCAAAGTTGTTTCTCTCGCTTCCTCCTTCCCTCTCCCCCTTCCCCCCTCTCTTTAGATGGAAGTGAT
TGTTGGGGACTTTGGGATCGTCGTGGTACCCAGGGATGCAGCGGACACCGACCGGATCATGAATCACTCC
TCCATACTCCGCAAGTACAAAGTGAGTCCTCCA , 8
TCACCAAAGTTGTTTCTCTCGCTTCCTCCTTCCCTCTCCCCCTTCCCCCCTCTCTTTAGATGGAAGTGAT
TGTTGGGGACTTTGGGATCGTCGTGGTACCCAGGGATGCAGCGGACACAGACCGGATCATGAATCACTCC
GCCATACTCCGCAAGTACAAAGTGAGTCCTCCA , 8
TCACCAAAGTTGTTTCTCTCGCTTCCTCCTTCCCTCTCCCCCTTCCCCCCTCTCTTTAGATGGAAGTGAT
TGTTGGGGACTTTGGGATCGTCGTGGTACCCAGGGATGCAGCGGACACAGACCGGATCATGAATCACTCC
TCCATGCTCCGCAAGTACAAAGTGAGTCCTCCA , 7

TCACCAAAGTTGTTTCTCTCGCTTCCTCCTTCCCTCTCCCCCTTCCCCCCTCTCTTTAGATGGAAGTGAT
TGTTGGGGACTTTGGGATCGTCGTGGTACCCAGGGATGCAGCGGACACAGACCGGATCATGAATCACTCC
TCCATACTCCGCAAGTACCAAGTGAGTCCTCCA , 7
TCACCAAAGTTGTTTCTCTCGCTTCCTCCTTCCCTCTCCCCCTTCCCCCCTCTCTTTAGATGGAAGTGAT
TGTTGGGGACTATGGGATCGTCGTGGTACCCAGGGATGCAGCGGACACAGACCGGATCATGAATCACTCC
TCCATACTCCGCAAGTACAAAGTGAGTCCTCCA , 6
TCACCAAAGTTGTTTCTCTCGCTTCCTCCTTCCCTCTCCCCCTTCCCCCCTCTCTTTAGATGGGAGTGAT
TGTTGGGGACTTTGGGATCGTCGTGGTACCCAGGGATGCAGCGGACACAGACCGGATCATGAATCACTCC
TCCATACTCCGCAAGTACAAAGTGAGTCCTCCA , 6
TCACCAAAGTTGTTTCTCTCGCTTCCTCCTTCCCTCTCCCCCTTCCCCCCTCTCCTTAGATGGAAGTGAT
TGTTGGGGACTTTGGGATCGTCGTGGTACCCAGGGATGCAGCGGACACAGACCGGATCATGAATCACTCC
TCCATACTCCGCAAGTACAAAGTGAGTCCTCCA , 5

GEIC-Plate01-C01 TOTAL:1965 OrderedDict([('WT_sp1', 1931), ('R232Q', 0), ('Silent Block Mod', 0), ('R232Q (Full ssODN)', 0), ('Silent Block Mod (Full ssODN)', 0)])   [(0, 1958), (-1, 7)]
TCACCAAAGTTGTTTCTCTCGCTTCCTCCTTCCCTCTCCCCCTTCCCCCCTCTCTTTAGATGGAAGTGAT
TGTTGGGGACTTTGGGATCGTCGTGGTACCCAGGGATGCAGCGGACACAGACCGGATCATGAATCACTCC
TCCATACTCCGCAAGTACAAAGTGAGTCCTCCA , 1716
TCACCAAAGTTGTTTCTCTCGCTTCCTCCTTCCCTCTCCCCCTTCCCCCCTCTCTTTAGATGGAAGTGAT
TGTTGGGGACTTTGGGATCGTCGTGGTACCCAGGGATGCAGCGGACACGGACCGGATCATGAATCACTCC
TCCATACTCCGCAAGTACAAAGTGAGTCCTCCA , 12
TCACCAAAGTTGTTTCTCTCGCTTCCTCCTTCCCTCTCCCCCTTCCCCCCTCTCTTTAGATGGAAGTGAT
TGTTGGGGACTTTGGGATCGTCGTGGTACCCAGGGATGCAGCGGACACAGACCGGATCATGAATCGCTCC
TCCATACTCCGCAAGTACAAAGTGAGTCCTCCA , 4
TCACCAAAGTTGTTTCTCTCGCTTCCTCCTTCCCTCTCCCCCTTCCCCCCTCTCTTTAGATGGAAGTGAT
TGTTGGGGACGTTGGGATCGTCGTGGTACCCAGGGATGCAGCGGACACAGACCGGATCATGAATCACTCC
TCCATACTCCGCAAGTACAAAGTGAGTCCTCCA , 4
TCACCAAAGTTGTTTCTCTCGCTTCCTCCTTCCCTCTCCCCCTTCCCCCCTCTCTTTAGATGGAAGTGAT
TGTTGGGGACTTTGGGATCGTCGTGGTACCCAGGGATGCAGCGGACACAGACCGGATCATGAATCACTCC
GCCATACTCCGCAAGTACAAAGTGAGTCCTCCA , 4
TCACCAAAGTTGTTTCTCTCGCTTCCTCCTTCCCTCTCCCCCTTCCCCCCTCTCTTTAGATGGAAGTGAT
TGTTGGGGACTTTGGGATCGTCGTGGTACCCAGGGATGCAGCGGACACCGACCGGATCATGAATCACTCC
TCCATACTCCGCAAGTACAAAGTGAGTCCTCCA , 3
TCACCAAAGTTGTTTCTCTCGCTTCCTCCTTCCCTCTCCCCCTTCCCCCCTCTCTTTAGATGGAAGTGAT
TGTTGGGGACTTTGGGATCGTCGTGGTACCCAGGGATGCAGCGGACACAGACCGGATCATGAATCACTCC
TCCATGCTCCGCAAGTACAAAGTGAGTCCTCCA , 3
TCACCAAAGTTGTTTCTCTCGCTTCCTCCTTCCCTCTCCCCCTTCCCCCCTCTCTTTAGATGGAAGTGAT
TGTTGGGGACTTTGGGATCGTCGTGGTACCTAGGGATGCAGCGGACACAGACCGGATCATGAATCACTCC
TCCATACTCCGCAAGTACAAAGTGAGTCCTCCA , 3
TCACCAAAGTTGTTTCTCTCGCTTCCTCCTTCCCTCTCCCCCTTCCCCCCTCTCTTTAGATGGAAGTGAT
TGTTGGGGACTTTGGGATCGTCGTGGTACCCAGGGATGCAGCGGACACAGACCGGATCATGAATCACTCC
TCCATACGCCGCAAGTACAAAGTGAGTCCTCCA , 3
TCACCAAAGTTGTTTCTCTCGCTTCCTCCTTCCCTCTCCCCCTTCCCCCCTCTCTTTAGATGGAAGTGAT
TGTTGGGGACTTTGGGATCGTCGTGGTACCCAGGGATGCGGCGGACACAGACCGGATCATGAATCACTCC
TCCATACTCCGCAAGTACAAAGTGAGTCCTCCA , 3
TCACCAAAGTTGTTTCTCTCGCTTCCTCCTTCCCTCTCCCCCTTCCCCCCTCTCTTTAGATGGAAGTGAT
TGTTGGGGACTTTGGGATCGTCGTGGTACCCAGGGATGCAGCAGACACAGACCGGATCATGAATCACTCC
TCCATACTCCGCAAGTACAAAGTGAGTCCTCCA , 3

TCACCAAAGTTGTTTCTCTCGCTTCCTCCTTCCCTCTCCCCCTTCCCCCCTCTCTTTAGATGGAAGTGAT
TGTTGGGGACTTTGGGATCGTCGTGGTACCCAGGGATGCAGCGGACACAGGCCGGATCATGAATCACTCC
TCCATACTCCGCAAGTACAAAGTGAGTCCTCCA , 2

GEIC-Plate01-C02 TOTAL:3906 OrderedDict([('WT_sp1', 1918), ('R232Q', 0), ('Silent Block Mod', 1916), ('R232Q (Full ssODN)', 0), ('Silent Block Mod (Full ssODN)', 1774)])   [(0, 3886), (-1, 16), (1, 2), (-29, 1), (-2, 1)]
TCACCAAAGTTGTTTCTCTCGCTTCCTCCTTCCCTCTCCCCCTTCCCCCCTCTCTTTAGATGGAAGTGAT
TGTTGGGGACTTTGGGATCGTCGTGGTACCCCGTGATGCAGCGGACACAGACCGGATCATGAATCACTCC
TCCATACTCCGCAAGTACAAAGTGAGTCCTCCA , 1748
TCACCAAAGTTGTTTCTCTCGCTTCCTCCTTCCCTCTCCCCCTTCCCCCCTCTCTTTAGATGGAAGTGAT
TGTTGGGGACTTTGGGATCGTCGTGGTACCCAGGGATGCAGCGGACACAGACCGGATCATGAATCACTCC
TCCATACTCCGCAAGTACAAAGTGAGTCCTCCA , 1720
TCACCAAAGTTGTTTCTCTCGCTTCCTCCTTCCCTCTCCCCCTTCCCCCCTCTCTTTAGATGGAAGTGAT
TGTTGGGGACTTTGGGATCGTCGTGGTACCCAGGGATGCAGCGGACACGGACCGGATCATGAATCACTCC
TCCATACTCCGCAAGTACAAAGTGAGTCCTCCA , 11
TCACCAAAGTTGTTTCTCTCGCTTCCTCCTTCCCTCTCCCCCTTCCCCCCTCTCTTTAGATGGAAGTGAT
TGTTGGGGACTGTGGGATCGTCGTGGTACCCCGTGATGCAGCGGACACAGACCGGATCATGAATCACTCC
TCCATACTCCGCAAGTACAAAGTGAGTCCTCCA , 6
TCACCAAAGTTGTTTCTCTCGCTTCCTCCTTCCCTCTCCCCCTTCCCCCCTCTCTTTAGATGGAAGTGAT
TGTTGGGGACGTTGGGATCGTCGTGGTACCCAGGGATGCAGCGGACACAGACCGGATCATGAATCACTCC
TCCATACTCCGCAAGTACAAAGTGAGTCCTCCA , 6
TCACCAAAGTTGTTTCTCTCGCTTCCTCCTTCCCTCTCCCCCTTCCCCCCTCTCTTTAGATGGAAGTGAT
TGTTGGGGACTTTGGGATCGTCGTGGTACCCAGGGATGCAGCGGACACAGGCCGGATCATGAATCACTCC
TCCATACTCCGCAAGTACAAAGTGAGTCCTCCA , 5
TCACCAAAGTTGTTTCTCTCGCTTCCTCCTTCCCTCTCCCCCTTCCCCCCTCTCTTTAGATGGAAGTGAT
TGTTGGGGACTTTGGGATCGTCGTGGTACCCCGGGATGCAGCGGACACAGACCGGATCATGAATCACTCC
TCCATACTCCGCAAGTACAAAGTGAGTCCTCCA , 5
TCACCAAAGTTGTTTCTCTCGCTTCCTCCTTCCCTCTCCCCCTTCCCCCCTCTCTTTAGATGGAAGTGAT
TGTTGGGGACTGTGGGATCGTCGTGGTACCCAGGGATGCAGCGGACACAGACCGGATCATGAATCACTCC
TCCATACTCCGCAAGTACAAAGTGAGTCCTCCA , 5
TCACCAAAGTTGTTTCTCTCGCTTCCTCCTTCCCTCTCCCCCTTCCCCCCTCTCTTTAGATGGAAGTGAT
TGTTGGGGACTTTGGGATCGTCGTGGTACCCAGTGATGCAGCGGACACAGACCGGATCATGAATCACTCC
TCCATACTCCGCAAGTACAAAGTGAGTCCTCCA , 4
TCACCAAAGTTGTTTCTCTCGCTTCCTCCTTCCCTCTCCCCCTTCCCCCCTCTCTTTAGATGGAAGTGAT
TGTTGGGGACTTTGGGATCGTCGTGGTACCCAGGGATGCAGCGGACACAGACCGGATCATGAATCACTCC
TCCATGCTCCGCAAGTACAAAGTGAGTCCTCCA , 4
TCACCAAAGTTGTTTCTCTCGCTTCCTCCTTCCCTCTCCCCCTTCCCCCCTCTCTTTAGATGGAAGTGAT
TGTTGGGGACTTTGGGATCGTCGTGGTACCCAGGGATGCAGCGGACACAGACCGGATCATGAATCACTCC
TCCATACGCCGCAAGTACAAAGTGAGTCCTCCA , 4
TCACCAAAGTTGTTTCTCTCGCTTCCTCCTTCCCTCTCCCCCTTCCCCCTCTCTTTAGATGGAAGTGATT
GTTGGGGACTTTGGGATCGTCGTGGTACCCCGTGATGCAGCGGACACAGACCGGATCATGAATCACTCCT
CCATACTCCGCAAGTACAAAGTGAGTCCTCCA , 4

GEIC-Plate01-C03 TOTAL:2829 OrderedDict([('WT_sp1', 2787), ('R232Q', 0), ('Silent Block Mod', 0), ('R232Q (Full ssODN)', 0), ('Silent Block Mod (Full ssODN)', 0)])   [(0, 2810), (-1, 14), (-2, 3), (1, 2)]

TCACCAAAGTTGTTTCTCTCGCTTCCTCCTTCCCTCTCCCCCTTCCCCCCTCTCTTTAGATGGAAGTGAT
TGTTGGGGACTTTGGGATCGTCGTGGTACCCAGGGATGCAGCGGACACAGACCGGATCATGAATCACTCC
TCCATACTCCGCAAGTACAAAGTGAGTCCTCCA , 2503
TCACCAAAGTTGTTTCTCTCGCTTCCTCCTTCCCTCTCCCCCTTCCCCCCTCTCTTTAGATGGAAGTGAT
TGTTGGGGACTTTGGGATCGTCGTGGTACCCAGGGATGCAGCGGACACGGACCGGATCATGAATCACTCC
TCCATACTCCGCAAGTACAAAGTGAGTCCTCCA , 13
TCACCAAAGTTGTTTCTCTCGCTTCCTCCTTCCCTCTCCCCCTTCCCCCCTCTCTTTAGATGGAAGTGAT
TGTTGGGGACTTTGGGATCGTCGTGGTACCCAGGGATGCAGCGGACACAGACCGGATCATGAATCGCTCC
TCCATACTCCGCAAGTACAAAGTGAGTCCTCCA , 7
TCACCAAAGTTGTTTCTCTCGCTTCCTCCTTCCCTCTCCCCCTTCCCCCCTCTCTTTAGATGGAAGTGAT
TGTTGGGGACTTTGGGATCGTCGTGGTACCCAGGGATGCAGCGGACACAGACCGGATCATGAATCACTCC
TCCATGCTCCGCAAGTACAAAGTGAGTCCTCCA , 6
TCACCAAAGTTGTTTCTCTCGCTTCCTCCTTCCCTCTCCCCCTTCCCCCCTCTCTTTAGATGGAAGTGAT
TGTTGGGGACTTTGGGATCGTCGTGGTACCCAGGGATGCAGCGGACACAGACCGGATCATGAATCACTCC
TCCATACTCTGCAAGTACAAAGTGAGTCCTCCA , 6
TCACCAAAGTTGTTTCTCTCGCTTCCTCCTTCCCTCTCCCCCTTCCCCCCTCTCTTTAGATGGAAGTGAT
TGTTGGGGACTTTGGGATCGTCGTGGTACCCAGGGATGCAGCGGACACAGGCCGGATCATGAATCACTCC
TCCATACTCCGCAAGTACAAAGTGAGTCCTCCA , 5
TCACCAAAGTTGTTTCTCTCGCTTCCTCCTTCCCTCTCCCCCTTCCCCCCTCTCTTTAGATGGAAGTGAT
TGTTGGGGACTTTGGGATCGTCGTGGTACCCAGGGATGCAGCGGACACCGACCGGATCATGAATCACTCC
TCCATACTCCGCAAGTACAAAGTGAGTCCTCCA , 5
TCACCAAAGTTGTTTCTCTCGCTTCCTCCTTCCCTCTCCCCCTTCCCCCCTCTCTTTAGATGGAAGTGAT
TGTTGGGGACTTTGGGATCGTCGTGGTACCCAGGGATGCAGCGGACACAGACCGGATCATGAATCACTCC
TCCATACGCCGCAAGTACAAAGTGAGTCCTCCA , 5
TCACCAAAGTTGTTTCTCTCGCTTCCTCCTTCCCTCTCCCCCTTCCCCCCTCTCTTTAGATGGAAGTGAT
TGTTGGGGACTTTGGGATCGTCGTGGTACCCAGGGATGCAGCGGACACAGACCAGATCATGAATCACTCC
TCCATACTCCGCAAGTACAAAGTGAGTCCTCCA , 5
TCACCAAAGTTGTTTCTCTCGCTTCCTCCTTCCCTCTCCCCCTTCCCCCCTCTCTTTAGATGGAAGTGAT
TGTTGGGGACTATGGGATCGTCGTGGTACCCAGGGATGCAGCGGACACAGACCGGATCATGAATCACTCC
TCCATACTCCGCAAGTACAAAGTGAGTCCTCCA , 4
TCACCAAAGTTGTTTCTCTCGCTTCCTCCTTCCCTCTCCCCCTTCCCCCCTCTCTTTAGATGGAAGTGAT
TGTTGGGGACTGTGGGATCGTCGTGGTACCCAGGGATGCAGCGGACACAGACCGGATCATGAATCACTCC
TCCATACTCCGCAAGTACAAAGTGAGTCCTCCA , 4
TCACCAAAGTTGTTTCTCTCGCTTCCTCCTTCCCTCTCCCCTTCCCCCCTCTCTTTAGATGGAAGTGATT
GTTGGGGACTTTGGGATCGTCGTGGTACCCAGGGATGCAGCGGACACAGACCGGATCATGAATCACTCCT
CCATACTCCGCAAGTACAAAGTGAGTCCTCCA , 3

GEIC-Plate01-C04 TOTAL:2796 OrderedDict([('WT_sp1', 2739), ('R232Q', 0), ('Silent Block Mod', 0), ('R232Q (Full ssODN)', 0), ('Silent Block Mod (Full ssODN)', 0)]) [(0, 2781), (-1, 13), (1, 1), (-3, 1)]
TCACCAAAGTTGTTTCTCTCGCTTCCTCCTTCCCTCTCCCCCTTCCCCCCTCTCTTTAGATGGAAGTGAT
TGTTGGGGACTTTGGGATCGTCGTGGTACCCAGGGATGCAGCGGACACAGACCGGATCATGAATCACTCC
TCCATACTCCGCAAGTACAAAGTGAGTCCTCCA , 2426
TCACCAAAGTTGTTTCTCTCGCTTCCTCCTTCCCTCTCCCCCTTCCCCCCTCTCTTTAGATGGAAGTGAT
TGTTGGGGACTTTGGGATCGTCGTGGTACCCAGGGATGCAGCGGACACGGACCGGATCATGAATCACTCC
TCCATACTCCGCAAGTACAAAGTGAGTCCTCCA , 15
TCACCAAAGTTGTTTCTCTCGCTTCCTCCTTCCCTCTCCCCCTTCCCCCCTCTCTTTAGATGGAAGTGAT
TGTTGGGGACTGTGGGATCGTCGTGGTACCCAGGGATGCAGCGGACACAGACCGGATCATGAATCACTCC
TCCATACTCCGCAAGTACAAAGTGAGTCCTCCA , 13

TCACCAAAGTTGTTTCTCTCGCTTCCTCCTTCCCTCTCCCCTTCCCCCCTCTCTTTAGATGGAAGTGAT
TGTTGGGGACTTTGGGATCGTCGTGGTACCCAGGGATGCAGCGGACACAGACCGGATCATGAATCGCTCC
TCCATACTCCGCAAGTACAAAGTGAGTCCTCCA , 12
TCACCAAAGTTGTTTCTCTCGCTTCCTCCTTCCCTCTCCCCTTCCCCCCTCTCTTTAGATGGAAGTGAT
TGTTGGGGACGTTGGGATCGTCGTGGTACCCAGGGATGCAGCGGACACAGACCGGATCATGAATCACTCC
TCCATACTCCGCAAGTACAAAGTGAGTCCTCCA , 7
TCACCAAAGTTGTTTCTCTCGCTTCCTCCTTCCCTCTCCCCTTCCCCCCTCTCTTTAGATGGAAGTGATT
GTTGGGGACTTTGGGATCGTCGTGGTACCCAGGGATGCAGCGGACACAGACCGGATCATGAATCACTCCT
CCATACTCCGCAAGTACAAAGTGAGTCCTCCA , 5
TCACCAAAGTTGTTTCTCTCGCTTCCTCCTTCCCTCTCCCCTTCCCCCCTCTCTTTAGATGGAAGTGAT
TGTTGGGGACTTTGGGGTCGTCGTGGTACCCAGGGATGCAGCGGACACAGACCGGATCATGAATCACTCC
TCCATACTCCGCAAGTACAAAGTGAGTCCTCCA , 5
TCACCAAAGTTGTTTCTCTCGCTTCCTCCTTCCCTCTCCCCTTCCCCCCTCTCTTTAGATGGAAGTGAT
TGTTGGGGACTTTGGGATCGTCGTGGTACCCAGGGATGCAGCGGACACAGACCGGATCATGAATCACTCC
TCCATACGCCGCAAGTACAAAGTGAGTCCTCCA , 5
TCACCAAAGTTGTTTCTCTCGCTTCCTCCTTCCCTCTCCCCTTCCCCCCTCTCTTTAGATGGAAGTGAT
TGTTGGGGACTTTGGGATCGTCGTGGTACCCAGGGATGCAGCGGACACCGACCGGATCATGAATCACTCC
TCCATACTCCGCAAGTACAAAGTGAGTCCTCCA , 4
TCACCAAAGTTGTTTCTCTCGCTTCCTCCTTCCCTCTCCCCTTCCCCCCTCTCTTTAGATGGAAGTGAT
TGTTGGGGACTTTGGGATCGTCGTGGTACCCAGGGATGCAGCGGACACAGACCAGATCATGAATCACTCC
TCCATACTCCGCAAGTACAAAGTGAGTCCTCCA , 4
TCACCAAAGTTGTTTCTCTCGCTTCCTCCTTCCCTCTCCCCTTCCCCCCTCTCTTTAGATGGAAGTGAT
TGTTGGGGACTTTGGGATCGTCGTAGTACCCAGGGATGCAGCGGACACAGACCGGATCATGAATCACTCC
TCCATACTCCGCAAGTACAAAGTGAGTCCTCCA , 3
TCACCAAAGTTGTTTCTCTCACTTCCTCCTTCCCTCTCCCCTTCCCCCCTCTCTTTAGATGGAAGTGAT
TGTTGGGGACTTTGGGATCGTCGTGGTACCCAGGGATGCAGCGGACACAGACCGGATCATGAATCACTCC
TCCATACTCCGCAAGTACAAAGTGAGTCCTCCA , 3

GEIC-Plate01-C05 TOTAL:3827 OrderedDict([('WT_sp1', 1897), ('R232Q', 1858), ('Silent Block Mod', 0), ('R232Q (Full ssODN)', 1696), ('Silent Block Mod (Full ssODN)', 0)]) [(0, 3812), (-1, 15)]
TCACCAAAGTTGTTTCTCTCGCTTCCTCCTTCCCTCTCCCCTTCCCCCCTCTCTTTAGATGGAAGTGAT
TGTTGGGGACTTTGGGATCGTCGTGGTACCCCAAGATGCAGCGGACACAGACCGGATCATGAATCACTCC
TCCATACTCCGCAAGTACAAAGTGAGTCCTCCA , 1678
TCACCAAAGTTGTTTCTCTCGCTTCCTCCTTCCCTCTCCCCTTCCCCCCTCTCTTTAGATGGAAGTGAT
TGTTGGGGACTTTGGGATCGTCGTGGTACCCAGGGATGCAGCGGACACAGACCGGATCATGAATCACTCC
TCCATACTCCGCAAGTACAAAGTGAGTCCTCCA , 1659
TCACCAAAGTTGTTTCTCTCGCTTCCTCCTTCCCTCTCCCCTTCCCCCCTCTCTTTAGATGGAAGTGAT
TGTTGGGGACTTTGGGATCGTCGTGGTACCCAGGGATGCAGCGGACACAGACCGGATCATGAATCGCTCC
TCCATACTCCGCAAGTACAAAGTGAGTCCTCCA , 10
TCACCAAAGTTGTTTCTCTCGCTTCCTCCTTCCCTCTCCCCTTCCCCCCTCTCTTTAGATGGAAGTGAT
TGTTGGGGACTTTGGGATCGTCGTGGTACCCAGGGATGCAGCGGACACGGACCGGATCATGAATCACTCC
TCCATACTCCGCAAGTACAAAGTGAGTCCTCCA , 8
TCACCAAAGTTGTTTCTCTCGCTTCCTCCTTCCCTCTCCCCTTCCCCCCTCTCTTTAGATGGAAGTGAT
TGTTGGGGACGTTGGGATCGTCGTGGTACCCAGGGATGCAGCGGACACAGACCGGATCATGAATCACTCC
TCCATACTCCGCAAGTACAAAGTGAGTCCTCCA , 7
TCACCAAAGTTGTTTCTCTCGCTTCCTCCTTCCCTCTCCCCTTCCCCCCTCTCTTTAGATGGAAGTGAT
TGTTGGGGACTTTGGGATCGTCGTGGTACCCCAAGATGCAGCGGACACAGACCGGATCATGAATCGCTCC
TCCATACTCCGCAAGTACAAAGTGAGTCCTCCA , 6

TCACCAAAGTTGTTTCTCTCGCTTCCTCCTTCCCTCTCCCCCTTCCCCCTCTCTTTAGATGGAAGTGATT
GTTGGGGACTTTGGGATCGTCGTGGTACCCAGGGATGCAGCGGACACAGACCGGATCATGAATCACTCCT
CCATACTCCGCAAGTACAAAGTGAGTCCTCCA , 5
TCACCAAAGTTGTTTCTCTCGCTTCCTCCTTCCCTCTCCCCCTTCCCCCCTCTCTTTAGATGGAAGTGAT
TGTTGGGGACTTTGGGATCGTCGTGGTACCCCAAGATGCAGCGGACACAGACCGGATCATGAATCACTCC
TCCATGCTCCGCAAGTACAAAGTGAGTCCTCCA , 5
TCACCAAAGTTGTTTCTCTCGCTTCCTCCTTCCCTCTCCCCCTTCCCCCCTCTCTTTAGATGGAAGTGAT
TGTTGGGGACTTTGGGATCGTCGTGGTACCCAAAGATGCAGCGGACACAGACCGGATCATGAATCACTCC
TCCATACTCCGCAAGTACAAAGTGAGTCCTCCA , 5
TCACCAAAGTTGTTTCTCTCGCTTCCTCCTTCCCTCTCCCCCTTCCCCCCTCTCTTTAGATGGAAGTGAT
TGTTGGGGACTGTGGGATCGTCGTGGTACCCCAAGATGCAGCGGACACAGACCGGATCATGAATCACTCC
TCCATACTCCGCAAGTACAAAGTGAGTCCTCCA , 5
TCACCAAAGTTGTTTCTCTCGCTTCCTCCTTCCCTCTCCCCCTTCCCCCCTCTCTTTAGATGGAAGTGAT
TGTTGGGGACTTTGGGATCGTCGTGGTACCCCAAGATGCAGCGGACACAGACCGGATCATGAATCACTCC
TCCATACGCCGCAAGTACAAAGTGAGTCCTCCA , 4
TCACCAAAGTTGTTTCTCTCGCTTCCTCCTTCCCTCTCCCCCTTCCCCCCTCTCTTTAGATGGAAGTGAT
TGTTGGGGACTTTGGGATCGTCGTGGTACCTCAAGATGCAGCGGACACAGACCGGATCATGAATCACTCC
TCCATACTCCGCAAGTACAAAGTGAGTCCTCCA , 4

GEIC-Plate01-C06 TOTAL:3705 OrderedDict([('WT_sp1', 1866), ('R232Q', 0), ('Silent Block Mod', 1757), ('R232Q (Full ssODN)', 0), ('Silent Block Mod (Full ssODN)', 1580)]) [(0, 3683), (-1, 20), (1, 1), (-2, 1)]
TCACCAAAGTTGTTTCTCTCGCTTCCTCCTTCCCTCTCCCCCTTCCCCCCTCTCTTTAGATGGAAGTGAT
TGTTGGGGACTTTGGGATCGTCGTGGTACCCAGGGATGCAGCGGACACAGACCGGATCATGAATCACTCC
TCCATACTCCGCAAGTACAAAGTGAGTCCTCCA , 1647
TCACCAAAGTTGTTTCTCTCGCTTCCTCCTTCCCTCTCCCCCTTCCCCCCTCTCTTTAGATGGAAGTGAT
TGTTGGGGACTTTGGGATCGTCGTGGTACCCCGTGATGCAGCGGACACAGACCGGATCATGAATCACTCC
TCCATACTCCGCAAGTACAAAGTGAGTCCTCCA , 1564
TCACCAAAGTTGTTTCTCTCGCTTCCTCCTTCCCTCTCCCCCTTCCCCCCTCTCTTTAGATGGAAGTGAT
TGTTGGGGACTTTGGGATCGTCGTGGTACCCAGGGATGCAGCGGACACGGACCGGATCATGAATCACTCC
TCCATACTCCGCAAGTACAAAGTGAGTCCTCCA , 14
TCACCAAAGTTGTTTCTCTCGCTTCCTCCTTCCCTCTCCCCCTTCCCCCCTCTCTTTAGATGGAAGTGAT
TGTTGGGGACTGTGGGATCGTCGTGGTACCCCGTGATGCAGCGGACACAGACCGGATCATGAATCACTCC
TCCATACTCCGCAAGTACAAAGTGAGTCCTCCA , 10
TCACCAAAGTTGTTTCTCTCGCTTCCTCCTTCCCTCTCCCCCTTCCCCCCTCTCTTTAGATGGAAGTGAT
TGTTGGGGACTGTGGGATCGTCGTGGTACCCAGGGATGCAGCGGACACAGACCGGATCATGAATCACTCC
TCCATACTCCGCAAGTACAAAGTGAGTCCTCCA , 7
TCACCAAAGTTGTTTCTCTCGCTTCCTCCTTCCCTCTCCCCCTTCCCCCCTCTCTTTAGATGGAAGTGAT
TGTTGGGGACTTTGGGATCGTCGTGGTACCCCGTGATGCAGCGGACACAGACCGGATCATGAATCACTCC
TCCATGCTCCGCAAGTACAAAGTGAGTCCTCCA , 7
TCACCAAAGTTGTTTCTCTCGCTTCCTCCTTCCCTCTCCCCCTTCCCCCCTCTCTTTAGATGGAAGTGAT
TGTTGGGGACTTTGGGATCGTCGTGGTACCCAGGGATGCAGCGGACACCGACCGGATCATGAATCACTCC
TCCATACTCCGCAAGTACAAAGTGAGTCCTCCA , 6
TCACCAAAGTTGTTTCTCTCGCTTCCTCCTTCCCTCTCCCCCTTCCCCCCTCTCTTTAGATGGAAGTGAT
TGTTGGGGACGTTGGGATCGTCGTGGTACCCCGTGATGCAGCGGACACAGACCGGATCATGAATCACTCC
TCCATACTCCGCAAGTACAAAGTGAGTCCTCCA , 6
TCACCAAAGTTGTTTCTCTCGCTTCCTCCTTCCCTCTCCCCCTTCCCCCCTCTCTTTAGATGGAAGTGAT
TGTTGGGGACTTTGGGATCGTCGTGGTACCCCGTGATGCAGCGGACACAGACCGGATCATGAATCACTCC

TCCATACTCCACAAGTACAAAGTGAGTCCTCCA , 6
TCACCAAAGTTGTTTCTCTCGCTTCCTCCTTCCCTCTCCCCCTTCCCCCCTCTCTTTAGATGGAAGTGAT
TGTTGGGGACTTTGGGATCGTCGTGGTACCCCGTGATGCAGCGGACACAGACCGGATCATGAATCGCTCC
TCCATACTCCGCAAGTACAAAGTGAGTCCTCCA , 5
TCACCAAAGTTGTTTCTCTCGCTTCCTCCTTCCCTCTCCCCCTTCCCCCCTCTCTTTAGATGGAAGTGAT
TGTTGGGGACTTTGGGATCGTCGTGGTACCCAGGGATGCAGCGGACACAGACCGGATCATGAATCGCTCC
TCCATACTCCGCAAGTACAAAGTGAGTCCTCCA , 5
TCACCAAAGTTGTTTCTCTCGCTTCCTCCTTCCCTCTCCCCCTTCCCCCCTCTCTTTAGATGGAAGTGAG
TGTTGGGGACTTTGGGATCGTCGTGGTACCCCGTGATGCAGCGGACACAGACCGGATCATGAATCACTCC
TCCATACTCCGCAAGTACAAAGTGAGTCCTCCA , 5

GEIC-Plate01-C07 TOTAL:2384 OrderedDict([('WT_sp1', 2335), ('R232Q', 0), ('Silent Block Mod', 0), ('R232Q (Full ssODN)', 0), ('Silent Block Mod (Full ssODN)', 0)])   [(0, 2370), (-1, 13), (-2, 1)]
TCACCAAAGTTGTTTCTCTCGCTTCCTCCTTCCCTCTCCCCCTTCCCCCCTCTCTTTAGATGGAAGTGAT
TGTTGGGGACTTTGGGATCGTCGTGGTACCCAGGGATGCAGCGGACACAGACCGGATCATGAATCACTCC
TCCATACTCCGCAAGTACAAAGTGAGTCCTCCA , 2023
TCACCAAAGTTGTTTCTCTCGCTTCCTCCTTCCCTCTCCCCCTTCCCCCCTCTCTTTAGATGGAAGTGAT
TGTTGGGGACTTTGGGATCGTCGTGGTACCCAGGGATGCAGCGGACACAGACCGGATCATGAATCGCTCC
TCCATACTCCGCAAGTACAAAGTGAGTCCTCCA , 12
TCACCAAAGTTGTTTCTCTCGCTTCCTCCTTCCCTCTCCCCCTTCCCCCCTCTCTTTAGATGGAAGTGAT
TGTTGGGGACTTTGGGATCGTCGTGGTACCCAGGGATGCAGCGGACACGGACCGGATCATGAATCACTCC
TCCATACTCCGCAAGTACAAAGTGAGTCCTCCA , 12
TCACCAAAGTTGTTTCTCTCGCTTCCTCCTTCCCTCTCCCCCTTCCCCCCTCTCTTTAGATGGAAGTGAT
TGTTGGGGACGTTGGGATCGTCGTGGTACCCAGGGATGCAGCGGACACAGACCGGATCATGAATCACTCC
TCCATACTCCGCAAGTACAAAGTGAGTCCTCCA , 11
TCACCAAAGTTGTTTCTCTCGCTTCCTCCTTCCCTCTCCCCCTTCCCCCCTCTCTTTAGATGGAAGTGAT
TGTTGGGGACTGTGGGATCGTCGTGGTACCCAGGGATGCAGCGGACACAGACCGGATCATGAATCACTCC
TCCATACTCCGCAAGTACAAAGTGAGTCCTCCA , 11
TCACCAAAGTTGTTTCTCTCGCTTCCTCCTTCCCTCTCCCCCTTCCCCCCTCTCTTTAGATGGAAGTGAT
TGTTGGGGACTTTGGGATCGTCGTGGTACCCAGGGATGCAGCGGACACCGACCGGATCATGAATCACTCC
TCCATACTCCGCAAGTACAAAGTGAGTCCTCCA , 7
TCACCAAAGTTGTTTCTCTCGCTTCCTCCTTCCCTCTCCCCCTTCCCCCCTCTCTTTAGATGGAAGTGAT
TGTTGGGGACTTTGGGATCGTCGTGGTACCCAGGGATGCAGCGGACACAGGCCGGATCATGAATCACTCC
TCCATACTCCGCAAGTACAAAGTGAGTCCTCCA , 6
TCACCAAAGTTGTTTCTCTCGCTTCCTCCTTCCCTCTCCCCCTTCCCCCCTCTCTTTAGATGGAAGTGAT
TGTTGGGGACTTTGAGATCGTCGTGGTACCCAGGGATGCAGCGGACACAGACCGGATCATGAATCACTCC
TCCATACTCCGCAAGTACAAAGTGAGTCCTCCA , 5
TCACCAAAGTTGTTTCTCTCGCTTCCTCCTTCCCTCTCCCCCTTCCCCCCTCTCTTTAGATGGAAGTGAT
TGTTGGGGACTTTGGGGTCGTCGTGGTACCCAGGGATGCAGCGGACACAGACCGGATCATGAATCACTCC
TCCATACTCCGCAAGTACAAAGTGAGTCCTCCA , 4
TCACCAAAGTTGTTTCTCTCGCTTCCTCCTTCCCTCTCCCCCTTCCCCCCTCTCTTTAGATGGAAGTGAT
TGTTGGGGACTTTGGGATCGTCGTGGTGCCCAGGGATGCAGCGGACACAGACCGGATCATGAATCACTCC
TCCATACTCCGCAAGTACAAAGTGAGTCCTCCA , 4
TCACCAAAGTTGTTTCTCTCGCTTCCTCCTTCCCTCTCCCCCTTCCCCCCTCTCTTTAGATGGAAGTGAT
TGTTGGGGACTTTGGGATCGTCGTGGTACCCAGGGATGCAGCGGACACAGACCGGATCATGAATCACTCC
TCCATGCTCCGCAAGTACAAAGTGAGTCCTCCA , 4
TCACCAAAGTTGTTTCTCTCGCTTCCTCCTTCCCTCTCCCCCTTCCCCCCTCTCTTTAGATGGAAGTGAT
TGTTGGGGACTTTGGGATCGTCGTGGTACCCAGGGATGCAGCGGACACAGACCGGATCATGAATCACTCC

TCCATACTCCGCAAGTACAAATTGAGTCCTCCA , 4

GEIC-Plate01-C08 TOTAL:3172 OrderedDict([('WT_sp1', 3102), ('R232Q', 0), ('Silent Block Mod', 0), ('R232Q (Full ssODN)', 0), ('Silent Block Mod (Full ssODN)', 0)]) [(0, 3162), (-1, 9), (-3, 1)]
TCACCAAAGTTGTTTCTCTCGCTTCCTCCTTCCCTCTCCCCCTTCCCCCCTCTCTTTAGATGGAAGTGAT
TGTTGGGGACTTTGGGATCGTCGTGGTACCCAGGGATGCAGCGGACACAGACCGGATCATGAATCACTCC
TCCATACTCCGCAAGTACAAAGTGAGTCCTCCA , 2739
TCACCAAAGTTGTTTCTCTCGCTTCCTCCTTCCCTCTCCCCCTTCCCCCCTCTCTTTAGATGGAAGTGAT
TGTTGGGGACTTTGGGATCGTCGTGGTACCCAGGGATGCAGCGGACACGGACCGGATCATGAATCACTCC
TCCATACTCCGCAAGTACAAAGTGAGTCCTCCA , 19
TCACCAAAGTTGTTTCTCTCGCTTCCTCCTTCCCTCTCCCCCTTCCCCCCTCTCTTTAGATGGAAGTGAT
TGTTGGGGACTGTGGGATCGTCGTGGTACCCAGGGATGCAGCGGACACAGACCGGATCATGAATCACTCC
TCCATACTCCGCAAGTACAAAGTGAGTCCTCCA , 13
TCACCAAAGTTGTTTCTCTCGCTTCCTCCTTCCCTCTCCCCCTTCCCCCCTCTCTTTAGATGGAAGTGAT
TGTTGGGGACGTTGGGATCGTCGTGGTACCCAGGGATGCAGCGGACACAGACCGGATCATGAATCACTCC
TCCATACTCCGCAAGTACAAAGTGAGTCCTCCA , 11
TCACCAAAGTTGTTTCTCTCGCTTCCTCCTTCCCTCTCCCCCTTCCCCCCTCTCTTTAGATGGAAGTGAT
TGTTGGGGACTTTGGGATCGTCGTGGTACCCAGGGATGCAGCGGACACAGACCGGATCATGAATCACTCC
GCCATACTCCGCAAGTACAAAGTGAGTCCTCCA , 11
TCACCAAAGTTGTTTCTCTCGCTTCCTCCTTCCCTCTCCCCCTTCCCCCCTCTCTTTAGATGGAAGTGAT
TGTTGGGGACTTTGGGATCGTCGTGGTACCCAGGGATGCAGCGGACACAGACCGGATCATGAATCGCTCC
TCCATACTCCGCAAGTACAAAGTGAGTCCTCCA , 10
TCACCAAAGTTGTTTCTCTCGCTTCCTCCTTCCCTCTCCCCCTTCCCCCCTCTCTTTAGATGGAAGTGAT
TGTTGGGGACTTTGGGATCGTCGTGGTACCCAGGGATGCAGCGGACACAGACCGGATCATGAATCACTCC
TCCATACGCCGCAAGTACAAAGTGAGTCCTCCA , 9
TCACCAAAGTTGTTTCTCTCGCTTCCTCCTTCCCTCTCCCCCTTCCCCCCTCTCTTTAGATGGAAGTGAT
TGTTGGGGACTTTGGGATCGTCGTTGTACCCAGGGATGCAGCGGACACAGACCGGATCATGAATCACTCC
TCCATACTCCGCAAGTACAAAGTGAGTCCTCCA , 7
TCACCAAAGTTGTTTCTCTCGCTTCCTCCTTCCCTCTCCCCCTTCCCCCCTCTCTTTAGATGGAAGTGAT
TGTTGGGGACTTTGGGATCGTCGTGGTACCCAGGGATGCAGCGGACACCGACCGGATCATGAATCACTCC
TCCATACTCCGCAAGTACAAAGTGAGTCCTCCA , 6
TCACCAAAGTTGTTTCTCTCGCTTCCTCCTTCCCTCTCCCCCTTCCCCCCTCTCTTTAGATGGAAGTGAG
TGTTGGGGACTTTGGGATCGTCGTGGTACCCAGGGATGCAGCGGACACAGACCGGATCATGAATCACTCC
TCCATACTCCGCAAGTACAAAGTGAGTCCTCCA , 5
TCACCAAAGTTGTTTCTCTCGCTTCCTCCTTCCCTCTCCCCCTTCCCCCCTCTCTTTAGATGGAAGTGAT
TGTTGGGGACTTTGGGATCGTCGTGGTACCCAGGGATGCAGCGGACACAGGCCGGATCATGAATCACTCC
TCCATACTCCGCAAGTACAAAGTGAGTCCTCCA , 4
TCACCAAAGTTGTTTCTCTCGCTTCCTCCTTCCCTCTCCCCCTTCCCCCCTCTCTTTAGATGGAAGTGAT
TGTTGGGGACTTTGGGATCGTCGTGGTACCCAGGGATGCAGCGGACACAGACCGGATCATGAATCACTCC
TCCATGCTCCGCAAGTACAAAGTGAGTCCTCCA , 4

GEIC-Plate01-D01 TOTAL:3634 OrderedDict([('WT_sp1', 1772), ('R232Q', 0), ('Silent Block Mod', 1769), ('R232Q (Full ssODN)', 0), ('Silent Block Mod (Full ssODN)', 1560)]) [(0, 3612), (-1, 22)]
TCACCAAAGTTGTTTCTCTCGCTTCCTCCTTCCCTCTCCCCCTTCCCCCCTCTCTTTAGATGGAAGTGAT
TGTTGGGGACTTTGGGATCGTCGTGGTACCCCGTGATGCAGCGGACACAGACCGGATCATGAATCACTCC
TCCATACTCCGCAAGTACAAAGTGAGTCCTCCA , 1537

TCACCAAAGTTGTTTCTCTCGCTTCCTCCTTCCCTCTCCCCCTTCCCCCCTCTCTTTAGATGGAAGTGAT
TGTTGGGGACTTTGGGATCGTCGTGGTACCCAGGGATGCAGCGGACACAGACCGGATCATGAATCACTCC
TCCATACTCCGCAAGTACAAAGTGAGTCCTCCA , 1509
TCACCAAAGTTGTTTCTCTCGCTTCCTCCTTCCCTCTCCCCCTTCCCCCCTCTCTTTAGATGGAAGTGAT
TGTTGGGGACTTTGGGATCGTCGTGGTACCCAGGGATGCAGCGGACACGGACCGGATCATGAATCACTCC
TCCATACTCCGCAAGTACAAAGTGAGTCCTCCA , 16
TCACCAAAGTTGTTTCTCTCGCTTCCTCCTTCCCTCTCCCCCTTCCCCCCTCTCTTTAGATGGAAGTGAT
TGTTGGGGACTGTGGGATCGTCGTGGTACCCCGTGATGCAGCGGACACAGACCGGATCATGAATCACTCC
TCCATACTCCGCAAGTACAAAGTGAGTCCTCCA , 13
TCACCAAAGTTGTTTCTCTCGCTTCCTCCTTCCCTCTCCCCCTTCCCCCCTCTCTTTAGATGGAAGTGAT
TGTTGGGGACTTTGGGATCGTCGTGGTACCCAGGGATGCAGCGGACACAGACCGGATCATGAATCGCTCC
TCCATACTCCGCAAGTACAAAGTGAGTCCTCCA , 11
TCACCAAAGTTGTTTCTCTCGCTTCCTCCTTCCCTCTCCCCCTTCCCCCCTCTCTTTAGATGGAAGTGAT
TGTTGGGGACGTTGGGATCGTCGTGGTACCCAGGGATGCAGCGGACACAGACCGGATCATGAATCACTCC
TCCATACTCCGCAAGTACAAAGTGAGTCCTCCA , 9
TCACCAAAGTTGTTTCTCTCGCTTCCTCCTTCCCTCTCCCCCTTCCCCCCTCTCTTTAGATGGAAGTGAT
TGTTGGGGACTTTGGGATCGTCGTGGTACCCAGGGATGCAGCGGACACCGACCGGATCATGAATCACTCC
TCCATACTCCGCAAGTACAAAGTGAGTCCTCCA , 8
TCACCAAAGTTGTTTCTCTCGCTTCCTCCTTCCCTCTCCCCCTTCCCCCCTCTCTTTAGATGGAAGTGAT
TGTTGGGGACTGTGGGATCGTCGTGGTACCCAGGGATGCAGCGGACACAGACCGGATCATGAATCACTCC
TCCATACTCCGCAAGTACAAAGTGAGTCCTCCA , 8
TCACCAAAGTTGTTTCTCTCGCTTCCTCCTTCCCTCTCCCCCTTCCCCCTCTCTTTAGATGGAAGTGATT
GTTGGGGACTTTGGGATCGTCGTGGTACCCAGGGATGCAGCGGACACAGACCGGATCATGAATCACTCCT
CCATACTCCGCAAGTACAAAGTGAGTCCTCCA , 8
TCACCAAAGTTGTTTCTCTCGCTTCCTCCTTCCCTCTCCCCCTTCCCCCCTCTCTTTAGATGGAAGTGAT
TGTTGGGGACTTTGGGATCGTCGTGGTACCCAGGGATGCAGCGGACACAGACCGGATCATGAATCACTCC
GCCATACTCCGCAAGTACAAAGTGAGTCCTCCA , 7
TCACCAAAGTTGTTTCTCTCGCTTCCTCCTTCCCTCTCCCCCTTCCCCCCTCTCTTTAGATGGAAGTGAT
TGTTGGGGACTTTGGGATCGTCGTGGTACCCAGGGATGCAGCGGACACAGACCGGATCATGAATCACTCC
TCCATACGCCGCAAGTACAAAGTGAGTCCTCCA , 6
TCACCAAAGTTGTTTCTCTCGCTTCCTCCTTCCCTCTCCCCCTTCCCCCCTCTCTTTAGATGGAAGTGAT
TGTTGGGGACTTTGGGATCGTCGTGGTACCCCGTGATGCAGCGGACACAGACCGGATCATGAATCGCTCC
TCCATACTCCGCAAGTACAAAGTGAGTCCTCCA , 5

GEIC-Plate01-D02 TOTAL:2344 OrderedDict([('WT_sp1', 1096), ('R232Q', 0), ('Silent Block Mod', 1201), ('R232Q (Full ssODN)', 0), ('Silent Block Mod (Full ssODN)', 1076)])   [(0, 2333), (-1, 11)]
TCACCAAAGTTGTTTCTCTCGCTTCCTCCTTCCCTCTCCCCCTTCCCCCCTCTCTTTAGATGGAAGTGAT
TGTTGGGGACTTTGGGATCGTCGTGGTACCCCGTGATGCAGCGGACACAGACCGGATCATGAATCACTCC
TCCATACTCCGCAAGTACAAAGTGAGTCCTCCA , 1062
TCACCAAAGTTGTTTCTCTCGCTTCCTCCTTCCCTCTCCCCCTTCCCCCCTCTCTTTAGATGGAAGTGAT
TGTTGGGGACTTTGGGATCGTCGTGGTACCCAGGGATGCAGCGGACACAGACCGGATCATGAATCACTCC
TCCATACTCCGCAAGTACAAAGTGAGTCCTCCA , 979
TCACCAAAGTTGTTTCTCTCGCTTCCTCCTTCCCTCTCCCCCTTCCCCCCTCTCTTTAGATGGAAGTGAT
TGTTGGGGACTTTGGGATCGTCGTGGTACCCAGGGATGCAGCGGACACGGACCGGATCATGAATCACTCC
TCCATACTCCGCAAGTACAAAGTGAGTCCTCCA , 9
TCACCAAAGTTGTTTCTCTCGCTTCCTCCTTCCCTCTCCCCCTTCCCCCCTCTCTTTAGATGGAAGTGAT
TGTTGGGGACTTTGGGATCGTCGTGGTACCCCGTGATGCAGCGGACACAGACCAGATCATGAATCACTCC
TCCATACTCCGCAAGTACAAAGTGAGTCCTCCA , 6

TCACCAAAGTTGTTTCTCTCGCTTCCTCCTTCCCTCTCCCCCTTCCCCCCTCTCTTTAGATGGAAGTGAT
TGTTGGGGACTTTGGGATCGTCGTGGTACCCAGTGATGCAGCGGACACAGACCGGATCATGAATCACTCC
TCCATACTCCGCAAGTACAAAGTGAGTCCTCCA , 5
TCACCAAAGTTGTTTCTCTCGCTTCCTCCTTCCCTCTCCCCCTTCCCCCCTCTCTTTAGATGGAAGTGAT
TGTTGGGGACTTTGGGATCGTCGTGGTACCCCGTGATGCAGCGGACACAGACCGGATCATGAATCGCTCC
TCCATACTCCGCAAGTACAAAGTGAGTCCTCCA , 5
TCACCAAAGTTGTTTCTCTCGCTTCCTCCTTCCCTCTCCCCCTTCCCCCCTCTCTTTAGATGGAAGTGAT
TGTTGGGGACTGTGGGATCGTCGTGGTACCCCGTGATGCAGCGGACACAGACCGGATCATGAATCACTCC
TCCATACTCCGCAAGTACAAAGTGAGTCCTCCA , 4
TCACCAAAGTTGTTTCTCTCGCTTCCTCCTTCCCTCTCCCCCTTCCCCCCTCTCTTTAGATGGAAGTGAT
TGTTGGGGACTTTGGGATCGTCGTGGTACCCAGGGATGCAGCGGACACAGACCGGATCATGAATCACTCC
TCCATGCTCCGCAAGTACAAAGTGAGTCCTCCA , 4
TCACCAAAGTTGTTTCTCTCGCTTCCTCCTTCCCTCTCCCCCTTCCCCCCTCTCTTTAGATGGAAGTGAT
TGTTGGGGACTTTGGGATCGTCGTGGTACCACGTGATGCAGCGGACACAGACCGGATCATGAATCACTCC
TCCATACTCCGCAAGTACAAAGTGAGTCCTCCA , 4
TCACCAAAGTTGTTTCTCTCGCTTCCTCCTTCCCTCTCCCCCTTCCCCCCTCTCTTTAGATGGAAGTGAT
TGTTGGGGACTTTGGGATCGTCGTGGTACCCCGTGATGCAGCGGACACAGACCGGATCATGAATCACTCC
TCCATACGCCGCAAGTACAAAGTGAGTCCTCCA , 4
TCACCAAAGTTGTTTCTCTCGCTTCCTCCTTCCCTCTCCCCCTTCCCCCCTCTCTTTAGATGGAAGTGAT
TGTTGGGGACTTTGGGATCGTCGTGGTACCCCGTGATGCAGCGGACACAGACTGGATCATGAATCACTCC
TCCATACTCCGCAAGTACAAAGTGAGTCCTCCA , 4
TCACCAAAGTTGTTTCTCTCGCTTCCTCCTTCCCTCTCCCCCTTCCCCCCTCTCTTTAGATGGAAGTGAT
TGTTGGGGACTTTGGGATCGTCGTGGTACCCCGTGATGCAGCGGACACAGACCGTATCATGAATCACTCC
TCCATACTCCGCAAGTACAAAGTGAGTCCTCCA , 3

GEIC-Plate01-D03 TOTAL:3200 OrderedDict([('WT_sp1', 3150), ('R232Q', 0), ('Silent Block Mod', 0), ('R232Q (Full ssODN)', 0), ('Silent Block Mod (Full ssODN)', 0)])   [(0, 3184), (-1, 15), (1, 1)]
TCACCAAAGTTGTTTCTCTCGCTTCCTCCTTCCCTCTCCCCCTTCCCCCCTCTCTTTAGATGGAAGTGAT
TGTTGGGGACTTTGGGATCGTCGTGGTACCCAGGGATGCAGCGGACACAGACCGGATCATGAATCACTCC
TCCATACTCCGCAAGTACAAAGTGAGTCCTCCA , 2821
TCACCAAAGTTGTTTCTCTCGCTTCCTCCTTCCCTCTCCCCCTTCCCCCCTCTCTTTAGATGGAAGTGAT
TGTTGGGGACTTTGGGATCGTCGTGGTACCCAGGGATGCAGCGGACACGGACCGGATCATGAATCACTCC
TCCATACTCCGCAAGTACAAAGTGAGTCCTCCA , 24
TCACCAAAGTTGTTTCTCTCGCTTCCTCCTTCCCTCTCCCCCTTCCCCCCTCTCTTTAGATGGAAGTGAT
TGTTGGGGACTTTGGGATCGTCGTGGTACCCAGGGATGCAGCGGACACAGACCGGATCATGAATCGCTCC
TCCATACTCCGCAAGTACAAAGTGAGTCCTCCA , 10
TCACCAAAGTTGTTTCTCTCGCTTCCTCCTTCCCTCTCCCCCTTCCCCCCTCTCTTTAGATGGAAGTGAT
TGTTGGGGACTGTGGGATCGTCGTGGTACCCAGGGATGCAGCGGACACAGACCGGATCATGAATCACTCC
TCCATACTCCGCAAGTACAAAGTGAGTCCTCCA , 9
TCACCAAAGTTGTTTCTCTCGCTTCCTCCTTCCCTCTCCCCCTTCCCCCCTCTCTTTAGATGGAAGTGAT
TGTTGGGGACTTTGGGATCGTCGTGGTACCCAGGGATGCAGCGGACACCGACCGGATCATGAATCACTCC
TCCATACTCCGCAAGTACAAAGTGAGTCCTCCA , 7
TCACCAAAGTTGTTTCTCTCGCTTCCTCCTTCCCTCTCCCCCTTCCCCCCTCTCTTTAGATGGAAGTGAT
TGTTGGGGACTTTGGGATCGTCGTGGTACCCAGGGATGCAGCAGACACAGACCGGATCATGAATCACTCC
TCCATACTCCGCAAGTACAAAGTGAGTCCTCCA , 6
TCACCAAAGTTGTTTCTCTCGCTTCCTCCTTCCCTCTCCCCCTTCCCCCTTCTCTTTAGATGGAAGTGAT
TGTTGGGGACTTTGGGATCGTCGTGGTACCCAGGGATGCAGCGGACACAGACCGGATCATGAATCACTCC
TCCATACTCCGCAAGTACAAAGTGAGTCCTCCA , 5

TCACCAAAGTTGTTTCTCTCGCTTCCTCCTTCCCTCTCCCCCTTCCCCCCTCTCTTTAGATGGAAGTGGT
TGTTGGGGACTTTGGGATCGTCGTGGTACCCAGGGATGCAGCGGACACAGACCGGATCATGAATCACTCC
TCCATACTCCGCAAGTACAAAGTGAGTCCTCCA , 5
TCACCAAAGTTGTTTCTCTCGCTTCCTCCTTCCCTCTCCCCCTTCCCCCCTCTCTTTAGATGGAAGTGAT
TGTTGGGGACTTTGGGATCGTCGTGGTACCCAGGGATGCAGCGGACACAGACCGGATCATGAATCACTCC
TCCATACTCCGCAAGTACCAAGTGAGTCCTCCA , 5
TCACCAAAGTTGTTTCTCTCGCTTCCTCCTTCCCTCTCCCCCTTCCCCCCTCTCTTTAGATGGAAGTGAT
TGTTGGGGACTTTGGGATCGTCGTGGTACCCAGGGATGCAGCGGACACAGGCCGGATCATGAATCACTCC
TCCATACTCCGCAAGTACAAAGTGAGTCCTCCA , 4
TCACCAAAGTTGTTTCTCTCGCTTCCTCCTTCCCTCTCCCCCTTCCCCCCTCTCTTTAGATGGAAGTGAT
TGTTGGGGACTTTGGGATCGTCGTGGTACCCAGGGATGCAGCGGACACAGACCGGATCATGAATCACTCC
TCCATACGCCGCAAGTACAAAGTGAGTCCTCCA , 4
TCACCAAAGTTGTTTCTCTCGCTTCCTCCTTCCCTCTCCCCCTTCCCCCCTCTCTTTAGATGGAAGTGAT
TGTTGGGGACTTTGGGATCTTCGTGGTACCCAGGGATGCAGCGGACACAGACCGGATCATGAATCACTCC
TCCATACTCCGCAAGTACAAAGTGAGTCCTCCA , 4

GEIC-Plate01-D04 TOTAL:3493 OrderedDict([('WT_sp1', 1783), ('R232Q', 0), ('Silent Block Mod', 1637), ('R232Q (Full ssODN)', 0), ('Silent Block Mod (Full ssODN)', 1481)])   [(0, 3479), (-1, 13), (-2, 1)]
TCACCAAAGTTGTTTCTCTCGCTTCCTCCTTCCCTCTCCCCCTTCCCCCCTCTCTTTAGATGGAAGTGAT
TGTTGGGGACTTTGGGATCGTCGTGGTACCCAGGGATGCAGCGGACACAGACCGGATCATGAATCACTCC
TCCATACTCCGCAAGTACAAAGTGAGTCCTCCA , 1577
TCACCAAAGTTGTTTCTCTCGCTTCCTCCTTCCCTCTCCCCCTTCCCCCCTCTCTTTAGATGGAAGTGAT
TGTTGGGGACTTTGGGATCGTCGTGGTACCCCGTGATGCAGCGGACACAGACCGGATCATGAATCACTCC
TCCATACTCCGCAAGTACAAAGTGAGTCCTCCA , 1462
TCACCAAAGTTGTTTCTCTCGCTTCCTCCTTCCCTCTCCCCCTTCCCCCCTCTCTTTAGATGGAAGTGAT
TGTTGGGGACTTTGGGATCGTCGTGGTACCCAGGGATGCAGCGGACACGGACCGGATCATGAATCACTCC
TCCATACTCCGCAAGTACAAAGTGAGTCCTCCA , 11
TCACCAAAGTTGTTTCTCTCGCTTCCTCCTTCCCTCTCCCCCTTCCCCCCTCTCTTTAGATGGAAGTGAT
TGTTGGGGACGTTGGGATCGTCGTGGTACCCAGGGATGCAGCGGACACAGACCGGATCATGAATCACTCC
TCCATACTCCGCAAGTACAAAGTGAGTCCTCCA , 9
TCACCAAAGTTGTTTCTCTCGCTTCCTCCTTCCCTCTCCCCCTTCCCCCCTCTCTTTAGATGGAAGTGAT
TGTTGGGGACGTTGGGATCGTCGTGGTACCCCGTGATGCAGCGGACACAGACCGGATCATGAATCACTCC
TCCATACTCCGCAAGTACAAAGTGAGTCCTCCA , 9
TCACCAAAGTTGTTTCTCTCGCTTCCTCCTTCCCTCTCCCCCTTCCCCCCTCTCTTTAGATGGAAGTGAT
TGTTGGGGACTGTGGGATCGTCGTGGTACCCAGGGATGCAGCGGACACAGACCGGATCATGAATCACTCC
TCCATACTCCGCAAGTACAAAGTGAGTCCTCCA , 9
TCACCAAAGTTGTTTCTCTCGCTTCCTCCTTCCCTCTCCCCCTTCCCCCCTCTCTTTAGATGGAAGTGAT
TGTTGGGGACTTTGGGATCGTCGTGGTACCCAGGGATGCAGCGGACACAGGCCGGATCATGAATCACTCC
TCCATACTCCGCAAGTACAAAGTGAGTCCTCCA , 7
TCACCAAAGTTGTTTCTCTCGCTTCCTCCTTCCCTCTCCCCCTTCCCCCCTCTCTTTAGATGGAAGTGAT
TGTTGGGGACTGTGGGATCGTCGTGGTACCCCGTGATGCAGCGGACACAGACCGGATCATGAATCACTCC
TCCATACTCCGCAAGTACAAAGTGAGTCCTCCA , 7
TCACCAAAGTTGTTTCTCTCGCTTCCTCCTTCCCTCTCCCCCTTCCCCCCTCTCTTTAGATGGAAGTGAT
TGTTGGGGACTTTGGGATCGTCGTGGTACCCCGTGATGCAGCGGACACAGACCGGATCATGAATCACTCC
TCCATACGCCGCAAGTACAAAGTGAGTCCTCCA , 5
TCACCAAAGTTGTTTCTCTCGCTTCCTCCTTCCCTCTCCCCCTTCCCCCCTCTCTTTAGATGGAAGTGAT
TGTTGGGGACTTTGGGATCGTCGTGGTACCCCGTGATGCAGCGGACACAGACCGGATCATGAATCACTCC
TCCATGCTCCGCAAGTACAAAGTGAGTCCTCCA , 5

TCACCAAAGTTGTTTCTCTCGCTTCCTCCTTCCCTCTCCCCCTTCCCCCCTCTCTTTAGATGGAAGTGAT
TGTTGGGGACTTTGGGATCGTCGTGGTACCCAGGGATGCAGCGGACACAGACCGGATCATGAATCGCTCC
TCCATACTCCGCAAGTACAAAGTGAGTCCTCCA , 4
TCACCAAAGTTGTTTCTCTCGCTTCCTCCTTCCCTCTCCCCCTTCCCCCCTCTCTTTAGATGGAAGTGAT
TGTTGGGGACTTTGGGATCGTCGTGGTACCCAGGGATGCAGCGGGCACAGACCGGATCATGAATCACTCC
TCCATACTCCGCAAGTACAAAGTGAGTCCTCCA , 4

GEIC-Plate01-D05 TOTAL:3857 OrderedDict([('WT_sp1', 1892), ('R232Q', 1843), ('Silent Block Mod', 0), ('R232Q (Full ssODN)', 1642), ('Silent Block Mod (Full ssODN)', 0)])   [(0, 3841), (-1, 15), (1, 1)]
TCACCAAAGTTGTTTCTCTCGCTTCCTCCTTCCCTCTCCCCCTTCCCCCCTCTCTTTAGATGGAAGTGAT
TGTTGGGGACTTTGGGATCGTCGTGGTACCCAGGGATGCAGCGGACACAGACCGGATCATGAATCACTCC
TCCATACTCCGCAAGTACAAAGTGAGTCCTCCA , 1674
TCACCAAAGTTGTTTCTCTCGCTTCCTCCTTCCCTCTCCCCCTTCCCCCCTCTCTTTAGATGGAAGTGAT
TGTTGGGGACTTTGGGATCGTCGTGGTACCCCAAGATGCAGCGGACACAGACCGGATCATGAATCACTCC
TCCATACTCCGCAAGTACAAAGTGAGTCCTCCA , 1621
TCACCAAAGTTGTTTCTCTCGCTTCCTCCTTCCCTCTCCCCCTTCCCCCCTCTCTTTAGATGGAAGTGAT
TGTTGGGGACTGTGGGATCGTCGTGGTACCCAGGGATGCAGCGGACACAGACCGGATCATGAATCACTCC
TCCATACTCCGCAAGTACAAAGTGAGTCCTCCA , 11
TCACCAAAGTTGTTTCTCTCGCTTCCTCCTTCCCTCTCCCCCTTCCCCCCTCTCTTTAGATGGAAGTGAT
TGTTGGGGACTTTGGGATCGTCGTGGTACCCCAAGATGCAGCGGACACAGACCGGATCATGAATCGCTCC
TCCATACTCCGCAAGTACAAAGTGAGTCCTCCA , 11
TCACCAAAGTTGTTTCTCTCGCTTCCTCCTTCCCTCTCCCCCTTCCCCCCTCTCTTTAGATGGAAGTGAT
TGTTGGGGACTGTGGGATCGTCGTGGTACCCCAAGATGCAGCGGACACAGACCGGATCATGAATCACTCC
TCCATACTCCGCAAGTACAAAGTGAGTCCTCCA , 10
TCACCAAAGTTGTTTCTCTCGCTTCCTCCTTCCCTCTCCCCCTTCCCCCCTCTCTTTAGATGGAAGTGAT
TGTTGGGGACGTTGGGATCGTCGTGGTACCCAGGGATGCAGCGGACACAGACCGGATCATGAATCACTCC
TCCATACTCCGCAAGTACAAAGTGAGTCCTCCA , 9
TCACCAAAGTTGTTTCTCTCGCTTCCTCCTTCCCTCTCCCCCTTCCCCCCTCTCTTTAGATGGAAGTGAT
TGTTGGGGACGTTGGGATCGTCGTGGTACCCCAAGATGCAGCGGACACAGACCGGATCATGAATCACTCC
TCCATACTCCGCAAGTACAAAGTGAGTCCTCCA , 9
TCACCAAAGTTGTTTCTCTCGCTTCCTCCTTCCCTCTCCCCCTTCCCCCCTCTCTTTAGATGGAAGTGAT
TGTTGGGGACTTTGGGATCGTCGTGGTACCCCAAGATGCAGCGGACACAGACCGGATCATGAATCACTCC
TCCATACGCCGCAAGTACAAAGTGAGTCCTCCA , 7
TCACCAAAGTTGTTTCTCTCGCTTCCTCCTTCCCTCTCCCCCTTCCCCCCTCTCTTTAGATGGAAGTGAT
TGTTGGGGACTTTGGGATCGTCGTGGTACCCAGGGATGCAGCGGACACGGACCGGATCATGAATCACTCC
TCCATACTCCGCAAGTACAAAGTGAGTCCTCCA , 7
TCACCAAAGTTGTTTCTCTCGCTTCCTCCTTCCCTCTCCCCCTTCCCCCCTCTCTTTAGATGGAAGTGAT
TGTTGGGGACTTTGGGATCGTCGTGGTACCCAGGGATGCAGCGGACACAGACCGGATCATGAATCGCTCC
TCCATACTCCGCAAGTACAAAGTGAGTCCTCCA , 6
TCACCAAAGTTGTTTCTCTCGCTTCCTCCTTCCCTCTCCCCCTTCCCCCCTCTCTTTAGATGGAAGTGAT
TGTTGGGGACTTTGGGATCGTCGTGGTCCCCCAAGATGCAGCGGACACAGACCGGATCATGAATCACTCC
TCCATACTCCGCAAGTACAAAGTGAGTCCTCCA , 6
TCACCAAAGTTGTTTCTCTCGCTTCCTCCTTCCCTCTCCCCCTTCCCCCCTCTCTTTAGATGGAAGTGAT
TGTTGGGGACTTTGGGATCGTCGTGGTACCCAAAGATGCAGCGGACACAGACCGGATCATGAATCACTCC
TCCATACTCCGCAAGTACAAAGTGAGTCCTCCA , 5

GEIC-Plate01-D06 TOTAL:3536 OrderedDict([('WT_sp1', 1753), ('R232Q',

0), ('Silent Block Mod', 1720), ('R232Q (Full ssODN)', 0), ('Silent Block Mod (Full ssODN)', 1536)])   [(0, 3516), (-1, 19), (1, 1)]
TCACCAAAGTTGTTTCTCTCGCTTCCTCCTTCCCTCTCCCCCTTCCCCCCTCTCTTTAGATGGAAGTGAT
TGTTGGGGACTTTGGGATCGTCGTGGTACCCAGGGATGCAGCGGACACAGACCGGATCATGAATCACTCC
TCCATACTCCGCAAGTACAAAGTGAGTCCTCCA , 1564
TCACCAAAGTTGTTTCTCTCGCTTCCTCCTTCCCTCTCCCCCTTCCCCCCTCTCTTTAGATGGAAGTGAT
TGTTGGGGACTTTGGGATCGTCGTGGTACCCCGTGATGCAGCGGACACAGACCGGATCATGAATCACTCC
TCCATACTCCGCAAGTACAAAGTGAGTCCTCCA , 1518
TCACCAAAGTTGTTTCTCTCGCTTCCTCCTTCCCTCTCCCCCTTCCCCCCTCTCTTTAGATGGAAGTGAT
TGTTGGGGACTTTGGGATCGTCGTGGTACCCAGGGATGCAGCGGACACGGACCGGATCATGAATCACTCC
TCCATACTCCGCAAGTACAAAGTGAGTCCTCCA , 15
TCACCAAAGTTGTTTCTCTCGCTTCCTCCTTCCCTCTCCCCCTTCCCCCCTCTCTTTAGATGGAAGTGAT
TGTTGGGGACTGTGGGATCGTCGTGGTACCCCGTGATGCAGCGGACACAGACCGGATCATGAATCACTCC
TCCATACTCCGCAAGTACAAAGTGAGTCCTCCA , 11
TCACCAAAGTTGTTTCTCTCGCTTCCTCCTTCCCTCTCCCCCTTCCCCCCTCTCTTTAGATGGAAGTGAT
TGTTGGGGACTGTGGGATCGTCGTGGTACCCAGGGATGCAGCGGACACAGACCGGATCATGAATCACTCC
TCCATACTCCGCAAGTACAAAGTGAGTCCTCCA , 10
TCACCAAAGTTGTTTCTCTCGCTTCCTCCTTCCCTCTCCCCCTTCCCCCCTCTCTTTAGATGGAAGTGAT
TGTTGGGGACTTTGGGATCGTCGTGGTACCCCGTGATGCAGCGGACACAGACCGGATCATGAATCGCTCC
TCCATACTCCGCAAGTACAAAGTGAGTCCTCCA , 9
TCACCAAAGTTGTTTCTCTCGCTTCCTCCTTCCCTCTCCCCCTTCCCCCCTCTCTTTAGATGGAAGTGAT
TGTTGGGGACTTTGGGATCGTCGTGGTACCCCGTGATGCAGCGGACACAGACCGGATCATGAATCACTCC
TCCATGCTCCGCAAGTACAAAGTGAGTCCTCCA , 8
TCACCAAAGTTGTTTCTCTCGCTTCCTCCTTCCCTCTCCCCCTTCCCCCCTCTCTTTAGATGGAAGTGAT
TGTTGGGGACTTTGGGATCGTCGTGGTACCCAGGGATGCAGCGGACACAGGCCGGATCATGAATCACTCC
TCCATACTCCGCAAGTACAAAGTGAGTCCTCCA , 4
TCACCAAAGTTGTTTCTCTCGCTTCCTCCTTCCCTCTCCCCCTTCCCCCCTCTCTTTAGATGGAAGTGAT
TGTTGGGGACTTTGGGATCGTCGTGGTACCCAGTGATGCAGCGGACACAGACCGGATCATGAATCACTCC
TCCATACTCCGCAAGTACAAAGTGAGTCCTCCA , 4
TCACCAAAGTTGTTTCTCTCGCTTCCTCCTTCCCTCTCCCCCTTTCCCCCTCTCTTTAGATGGAAGTGAT
TGTTGGGGACTTTGGGATCGTCGTGGTACCCCGTGATGCAGCGGACACAGACCGGATCATGAATCACTCC
TCCATACTCCGCAAGTACAAAGTGAGTCCTCCA , 4
TCACCAAAGTTGTTTCTCTCGCTTCCTCCTTCCCTCTCCCCCTTCCCCCCTCTCTTTAGATGGAAGTGAT
TGTTGGGGACTTTGGGATCGTCGTGGTACCCCGTGATGCAGCGGACACAGACCGGATCATGAATCACTCC
TCCATACTCCGCAAGTACCAAGTGAGTCCTCCA , 4
TCACCAAAGTTGTTTCTCTCGCTTCCTCCTTCCCTCTCCCCCTTCCCCCCTCTCTTTAGATGGAAGTGAT
TGTTGGGGACGTTGGGATCGTCGTGGTACCCAGGGATGCAGCGGACACAGACCGGATCATGAATCACTCC
TCCATACTCCGCAAGTACAAAGTGAGTCCTCCA , 4

GEIC-Plate01-D07 TOTAL:3602 OrderedDict([('WT_sp1', 3518), ('R232Q', 0), ('Silent Block Mod', 0), ('R232Q (Full ssODN)', 0), ('Silent Block Mod (Full ssODN)', 0)])   [(0, 3588), (-1, 13), (-29, 1)]
TCACCAAAGTTGTTTCTCTCGCTTCCTCCTTCCCTCTCCCCCTTCCCCCCTCTCTTTAGATGGAAGTGAT
TGTTGGGGACTTTGGGATCGTCGTGGTACCCAGGGATGCAGCGGACACAGACCGGATCATGAATCACTCC
TCCATACTCCGCAAGTACAAAGTGAGTCCTCCA , 2993
TCACCAAAGTTGTTTCTCTCGCTTCCTCCTTCCCTCTCCCCCTTCCCCCCTCTCTTTAGATGGAAGTGAT
TGTTGGGGACTTTGGGATCGTCGTGGTACCCAGGGATGCAGCGGACACGGACCGGATCATGAATCACTCC
TCCATACTCCGCAAGTACAAAGTGAGTCCTCCA , 27
TCACCAAAGTTGTTTCTCTCGCTTCCTCCTTCCCTCTCCCCCTTCCCCCCTCTCTTTAGATGGAAGTGAT

TGTTGGGGACTTTGGGATCGTCGTGGTACCCAGGGATGCAGCGGACACAGACCGGATCATGAATCGCTCC
TCCATACTCCGCAAGTACAAAGTGAGTCCTCCA , 25
TCACCAAAGTTGTTTCTCTCGCTTCCTCCTTCCCTCTCCCCCTTCCCCCCTCTCTTTAGATGGAAGTGAT
TGTTGGGGACGTTGGGATCGTCGTGGTACCCAGGGATGCAGCGGACACAGACCGGATCATGAATCACTCC
TCCATACTCCGCAAGTACAAAGTGAGTCCTCCA , 20
TCACCAAAGTTGTTTCTCTCGCTTCCTCCTTCCCTCTCCCCCTTCCCCCCTCTCTTTAGATGGAAGTGAT
TGTTGGGGACTGTGGGATCGTCGTGGTACCCAGGGATGCAGCGGACACAGACCGGATCATGAATCACTCC
TCCATACTCCGCAAGTACAAAGTGAGTCCTCCA , 14
TCACCAAAGTTGTTTCTCTCGCTTCCTCCTTCCCTCTCCCCCTTCCCCCCTCTCTTTAGATGGAAGTGAT
TGTTGGGGACTTTGGGATCGTCGTGGTACCCAGGGATGCAGCGGACACAGACCGGATCATGAATCACTCC
TCCATGCTCCGCAAGTACAAAGTGAGTCCTCCA , 14
TCACCAAAGTTGTTTCTCTCGCTTCCTCCTTCCCTCTCCCCCTTCCCCCCTCTCTTTAGATGGAAGTGAT
TGTTGGGGACTTTGGGATCGTCGTGGTACCCAGGGATGCAGCGGACACAGACCGGATCATGAATCACTCC
TCCATACTCCGCAAGTACCAAGTGAGTCCTCCA , 10
TCACCAAAGTTGTTTCTCTCGCTTCCTCCTTCCCTCTCCCCCTTCCCCCCTCTCTTTAGATGGAAGTGAT
TGTTGGGGACTTTGGGATCGTCGTGGTACCCAGGGATGCAGCGGACACAGACCGGATCATGAATCACTCC
GCCATACTCCGCAAGTACAAAGTGAGTCCTCCA , 8
TCACCAAAGTTGTTTCTCTCGCTTCCTCCTTCCCTCTCCCCCTTCCCCCCTCTCTTTAGATGGAAGTGAT
TGTTGGGGACTTTGGGATCGTCGTGGTACCCAGGGATGCAGCGGACACAGACCGGATCATGAATCACTCC
TCCATACGCCGCAAGTACAAAGTGAGTCCTCCA , 7
TCACCAAAGTTGTTTCTCTCGCTTCCTCCTTCCCTCTCCCCCTTCCCCCCTCTCTTTAGATGGAAGTGAT
TGTTGGGGACTTTGGGATCGTCGTGGTACCCAGGGATGCAGCGGACACCGACCGGATCATGAATCACTCC
TCCATACTCCGCAAGTACAAAGTGAGTCCTCCA , 6
TCACCAAAGTTGTTTCTCTCGCTTCCTCCTTCCCTCTCCCCCTTCCCCCCTCTCTTTAGATGGAAGTGAT
TGTTGGGGACTTTGGGATCGTCGTGGTACCCAGGGATGCAGCGGACACAGGCCGGATCATGAATCACTCC
TCCATACTCCGCAAGTACAAAGTGAGTCCTCCA , 6
TCACCAAAGTTGTTTCTCTCGCTTCCTCCTTCCCTCTCCCCCTTCCCCCCTCTCTTTAGATGGAAGTGAG
TGTTGGGGACTTTGGGATCGTCGTGGTACCCAGGGATGCAGCGGACACAGACCGGATCATGAATCACTCC
TCCATACTCCGCAAGTACAAAGTGAGTCCTCCA , 6

GEIC-Plate01-D08 TOTAL:3790 OrderedDict([('WT_sp1', 3726), ('R232Q', 0), ('Silent Block Mod', 1), ('R232Q (Full ssODN)', 0), ('Silent Block Mod (Full ssODN)', 1)]) [(0, 3770), (-1, 19), (1, 1)]
TCACCAAAGTTGTTTCTCTCGCTTCCTCCTTCCCTCTCCCCCTTCCCCCCTCTCTTTAGATGGAAGTGAT
TGTTGGGGACTTTGGGATCGTCGTGGTACCCAGGGATGCAGCGGACACAGACCGGATCATGAATCACTCC
TCCATACTCCGCAAGTACAAAGTGAGTCCTCCA , 3319
TCACCAAAGTTGTTTCTCTCGCTTCCTCCTTCCCTCTCCCCCTTCCCCCCTCTCTTTAGATGGAAGTGAT
TGTTGGGGACTTTGGGATCGTCGTGGTACCCAGGGATGCAGCGGACACAGACCGGATCATGAATCGCTCC
TCCATACTCCGCAAGTACAAAGTGAGTCCTCCA , 17
TCACCAAAGTTGTTTCTCTCGCTTCCTCCTTCCCTCTCCCCCTTCCCCCCTCTCTTTAGATGGAAGTGAT
TGTTGGGGACTTTGGGATCGTCGTGGTACCCAGGGATGCAGCGGACACGGACCGGATCATGAATCACTCC
TCCATACTCCGCAAGTACAAAGTGAGTCCTCCA , 17
TCACCAAAGTTGTTTCTCTCGCTTCCTCCTTCCCTCTCCCCCTTCCCCCCTCTCTTTAGATGGAAGTGAT
TGTTGGGGACTGTGGGATCGTCGTGGTACCCAGGGATGCAGCGGACACAGACCGGATCATGAATCACTCC
TCCATACTCCGCAAGTACAAAGTGAGTCCTCCA , 13
TCACCAAAGTTGTTTCTCTCGCTTCCTCCTTCCCTCTCCCCCTTCCCCCCTCTCTTTAGATGGAAGTGAT
TGTTGGGGACGTTGGGATCGTCGTGGTACCCAGGGATGCAGCGGACACAGACCGGATCATGAATCACTCC
TCCATACTCCGCAAGTACAAAGTGAGTCCTCCA , 10
TCACCAAAGTTGTTTCTCTCGCTTCCTCCTTCCCTCTCCCCCTTCCCCCCTCTCTTTAGATGGAAGTGAT

TGTTGGGGACTTTGGGATCGTCGTGGTACCCAGGGATGCAGCGGACACAGACCGGATCATGAATCACTCC
TCCATGCTCCGCAAGTACAAAGTGAGTCCTCCA , 7
TCACCAAAGTTGTTTCTCTCGCTTCCTCCTTCCCTCTCCCCCTTCCCCCCTCTCTTTAGATGGAAGTGAT
TGTTGGGGACTTTGGGATCGTCGTGGTACCCAGGGATGCAGCGGACACAGGCCGGATCATGAATCACTCC
TCCATACTCCGCAAGTACAAAGTGAGTCCTCCA , 6
TCACCAAAGTTGTTTCTCTCGCTTCCTCCTTCCCTCTCCCCCTTCCCCCCTCTCTTTAGATGGAAGTGAT
TGTTGGGGACTTTGGGATCGTCGTGGTACCCAGGGATGCAGAGGACACAGACCGGATCATGAATCACTCC
TCCATACTCCGCAAGTACAAAGTGAGTCCTCCA , 6
TCACCAAAGTTGTTTCTCTCGCTTCCTCCTTCCCTCTCCCCCTTCCCCCCTCTCTTTAGATGGAAGTGAT
TGTTGGGGACTTTGGGATCGTCGTGGTACCCAGGGATGCAGCGGACACAGACCGGATCATGAATCACTCC
GCCATACTCCGCAAGTACAAAGTGAGTCCTCCA , 5
TCACCAAAGTTGTTTCTCTCGCTTCCTCCTTCCCTCTCCCCCTTCCCCCCTCTCTTTAGATGGAAGTGAT
TGTTGGGGACTATGGGATCGTCGTGGTACCCAGGGATGCAGCGGACACAGACCGGATCATGAATCACTCC
TCCATACTCCGCAAGTACAAAGTGAGTCCTCCA , 4
TCACCAAAGTTGTTTCTCTCGCTTCCTCCTTCCCTCTCCCCCTTCCCCCCTCTCTTTAGATGGAAGTGAT
TGTTGGGGACTTTGGGATCGTCGTGGTACCCAGGGATGCAGCGGACACTGACCGGATCATGAATCACTCC
TCCATACTCCGCAAGTACAAAGTGAGTCCTCCA , 4
TCACCAAAGTTGTTTCTCTCGCTTCCTCCTTCCCTCTCCCCCTTCCCCCCTCTCTTTAGATGGAAGTGAT
TGTTGGGGACTTTGGGATCGTCGTGGTACCCAGGGATGCAGCGGACACCGACCGGATCATGAATCACTCC
TCCATACTCCGCAAGTACAAAGTGAGTCCTCCA , 4

GEIC-Plate01-E01 TOTAL:3408 OrderedDict([('WT_sp1', 1762), ('R232Q', 4), ('Silent Block Mod', 1552), ('R232Q (Full ssODN)', 3), ('Silent Block Mod (Full ssODN)', 1422)]) [(0, 3393), (-1, 12), (1, 3)]
TCACCAAAGTTGTTTCTCTCGCTTCCTCCTTCCCTCTCCCCCTTCCCCCCTCTCTTTAGATGGAAGTGAT
TGTTGGGGACTTTGGGATCGTCGTGGTACCCAGGGATGCAGCGGACACAGACCGGATCATGAATCACTCC
TCCATACTCCGCAAGTACAAAGTGAGTCCTCCA , 1536
TCACCAAAGTTGTTTCTCTCGCTTCCTCCTTCCCTCTCCCCCTTCCCCCCTCTCTTTAGATGGAAGTGAT
TGTTGGGGACTTTGGGATCGTCGTGGTACCCCGTGATGCAGCGGACACAGACCGGATCATGAATCACTCC
TCCATACTCCGCAAGTACAAAGTGAGTCCTCCA , 1409
TCACCAAAGTTGTTTCTCTCGCTTCCTCCTTCCCTCTCCCCCTTCCCCCCTCTCTTTAGATGGAAGTGAT
TGTTGGGGACTTTGGGATCGTCGTGGTACCCAGGGATGCAGCGGACACGGACCGGATCATGAATCACTCC
TCCATACTCCGCAAGTACAAAGTGAGTCCTCCA , 12
TCACCAAAGTTGTTTCTCTCGCTTCCTCCTTCCCTCTCCCCCTTCCCCCCTCTCTTTAGATGGAAGTGAT
TGTTGGGGACGTTGGGATCGTCGTGGTACCCAGGGATGCAGCGGACACAGACCGGATCATGAATCACTCC
TCCATACTCCGCAAGTACAAAGTGAGTCCTCCA , 11
TCACCAAAGTTGTTTCTCTCGCTTCCTCCTTCCCTCTCCCCCTTCCCCCCTCTCTTTAGATGGAAGTGAT
TGTTGGGGACTGTGGGATCGTCGTGGTACCCCGTGATGCAGCGGACACAGACCGGATCATGAATCACTCC
TCCATACTCCGCAAGTACAAAGTGAGTCCTCCA , 8
TCACCAAAGTTGTTTCTCTCGCTTCCTCCTTCCCTCTCCCCCTTCCCCCCTCTCTTTAGATGGAAGTGAT
TGTTGGGGACTGTGGGATCGTCGTGGTACCCAGGGATGCAGCGGACACAGACCGGATCATGAATCACTCC
TCCATACTCCGCAAGTACAAAGTGAGTCCTCCA , 7
TCACCAAAGTTGTTTCTCTCGCTTCCTCCTTCCCTCTCCCCCTTCCCCCCTCTCTTTAGATGGAAGTGAT
TGTTGGGGACGTTGGGATCGTCGTGGTACCCCGTGATGCAGCGGACACAGACCGGATCATGAATCACTCC
TCCATACTCCGCAAGTACAAAGTGAGTCCTCCA , 6
TCACCAAAGTTGTTTCTCTCGCTTCCTCCTTCCCTCTCCCCCTTCCCCCCTCTCTTTAGATGGAAGTGAT
TGTTGGGGACTTTGGGATCGTCGTGGTACCCAGGGATGCAGCGGACACCGACCGGATCATGAATCACTCC
TCCATACTCCGCAAGTACAAAGTGAGTCCTCCA , 5
TCACCAAAGTTGTTTCTCTCGCTTCCTCCTTCCCTCTCCCCCTTCCCCCCTCTCTTTAGATGGAAGTGAT

TGTTGGGGACTTTGGGATCGTCGTGGTACCCCGTGATGCAGCGGACACAGACCGGATCATGAATCACTCC
TCCATGCTCCGCAAGTACAAAGTGAGTCCTCCA , 5
TCACCAAAGTTGTTTCTCTCGCTTCCTCCTTCCCTCTCCCCCTTCCCCCCTCTCTTTAGATGGAAGTGAT
TGTTGGGGACTTTGGGATCGTCGTGGTACCCAGTGATGCAGCGGACACAGACCGGATCATGAATCACTCC
TCCATACTCCGCAAGTACAAAGTGAGTCCTCCA , 5
TCACCAAAGTTGTTTCTCTCGCTTCCTCCTTCCCTCTCCCCCTTCCCCCCTCTCTTTAGATGGAAGTGAT
TGTTGGGGACTTTGGGATCGTCGTGGTACCCCGTGATGCAGCGGACACAGACCAGATCATGAATCACTCC
TCCATACTCCGCAAGTACAAAGTGAGTCCTCCA , 4
TCACCAAAGTTGTTTCTCTCGCTTCCTCCTTCCCTCTCCCCCTTCCCCCTCTCTTTAGATGGAAGTGATT
GTTGGGGACTTTGGGATCGTCGTGGTACCCCGTGATGCAGCGGACACAGACCGGATCATGAATCACTCCT
CCATACTCCGCAAGTACAAAGTGAGTCCTCCA , 4

GEIC-Plate01-E02 TOTAL:2273 OrderedDict([('WT_sp1', 1050), ('R232Q', 0), ('Silent Block Mod', 1170), ('R232Q (Full ssODN)', 0), ('Silent Block Mod (Full ssODN)', 1053)]) [(0, 2254), (-1, 16), (1, 3)]
TCACCAAAGTTGTTTCTCTCGCTTCCTCCTTCCCTCTCCCCCTTCCCCCCTCTCTTTAGATGGAAGTGAT
TGTTGGGGACTTTGGGATCGTCGTGGTACCCCGTGATGCAGCGGACACAGACCGGATCATGAATCACTCC
TCCATACTCCGCAAGTACAAAGTGAGTCCTCCA , 1040
TCACCAAAGTTGTTTCTCTCGCTTCCTCCTTCCCTCTCCCCCTTCCCCCCTCTCTTTAGATGGAAGTGAT
TGTTGGGGACTTTGGGATCGTCGTGGTACCCAGGGATGCAGCGGACACAGACCGGATCATGAATCACTCC
TCCATACTCCGCAAGTACAAAGTGAGTCCTCCA , 920
TCACCAAAGTTGTTTCTCTCGCTTCCTCCTTCCCTCTCCCCCTTCCCCCCTCTCTTTAGATGGAAGTGAT
TGTTGGGGACGTTGGGATCGTCGTGGTACCCAGGGATGCAGCGGACACAGACCGGATCATGAATCACTCC
TCCATACTCCGCAAGTACAAAGTGAGTCCTCCA , 9
TCACCAAAGTTGTTTCTCTCGCTTCCTCCTTCCCTCTCCCCCTTCCCCCCTCTCTTTAGATGGAAGTGAT
TGTTGGGGACTGTGGGATCGTCGTGGTACCCCGTGATGCAGCGGACACAGACCGGATCATGAATCACTCC
TCCATACTCCGCAAGTACAAAGTGAGTCCTCCA , 7
TCACCAAAGTTGTTTCTCTCGCTTCCTCCTTCCCTCTCCCCCTTCCCCCCTCTCTTTAGATGGAAGTGAT
TGTTGGGGACTTTGGGATCGTCGTGGTACCCCGTGATGCAGCAGACACAGACCGGATCATGAATCACTCC
TCCATACTCCGCAAGTACAAAGTGAGTCCTCCA , 7
TCACCAAAGTTGTTTCTCTCGCTTCCTCCTTCCCTCTCCCCCTTCCCCCCTCTCTTTAGATGGAAGTGAT
TGTTGGGGACTTTGGGATCGTCGTGGTACCCAGGGATGCAGCGGACACGGACCGGATCATGAATCACTCC
TCCATACTCCGCAAGTACAAAGTGAGTCCTCCA , 6
TCACCAAAGTTGTTTCTCTCGCTTCCTCCTTCCCTCTCCCCCTTCCCCCCTCTCTTTAGATGGAAGTGAT
TGTTGGGGACTTTGGGATCGTCGTGGTACCCCGTGATGCAGCGGACACAGACCGGATCATGAATCACTCC
TCCATACGCCGCAAGTACAAAGTGAGTCCTCCA , 5
TCACCAAAGTTGTTTCTCTCGCTTCCTCCTTCCCTCTCCCCCTTCCCCCTCTCTTTAGATGGAAGTGATT
GTTGGGGACTTTGGGATCGTCGTGGTACCCCGTGATGCAGCGGACACAGACCGGATCATGAATCACTCCT
CCATACTCCGCAAGTACAAAGTGAGTCCTCCA , 4
TCACCAAAGTTGTTTCTCTCGCTTCCTCCTTCCCTCTCCCCCTTCCCCCCTCTCTTTAGATGGAAGTGAT
TGTTGGGGACTTTGGGATCGTCGTGGTACCCCGTGATGCAGCGGACACAGACCGGATCATGAATCGCTCC
TCCATACTCCGCAAGTACAAAGTGAGTCCTCCA , 4
TCACCAAAGTTGTTTCTCTCGCTTCCTCCTTCCCTCTCCCCCTTCCCCCCTCTCTTTAGATGGAAGTGAT
TGTTGGGGACGTTGGGATCGTCGTGGTACCCCGTGATGCAGCGGACACAGACCGGATCATGAATCACTCC
TCCATACTCCGCAAGTACAAAGTGAGTCCTCCA , 4
TCACCAAAGTTGTTTCTCTCGCTTCCTCCTTCCCTCTCCCCCTTCCCCCTCTCTTTAGATGGAAGTGATT
GTTGGGGACTTTGGGATCGTCGTGGTACCCAGGGATGCAGCGGACACAGACCGGATCATGAATCACTCCT
CCATACTCCGCAAGTACAAAGTGAGTCCTCCA , 3
TCACCAAAGTTGTTTCTCTCGCTTCCTCCTTCCCTCTCCCCCTTCCCCCCTCTCTTTAGATGGAAGTGAT

TGTTGGGGACTTTGGGATCGTCGTGGTACCCCGTGATGCAGCGGACACAGACCGGATCATGAATCACTCC
TCCATACTCCGCAAGTACCAAGTGAGTCCTCCA , 3

GEIC-Plate01-E03 TOTAL:2941 OrderedDict([('WT_sp1', 2885), ('R232Q', 0), ('Silent Block Mod', 0), ('R232Q (Full ssODN)', 0), ('Silent Block Mod (Full ssODN)', 0)])   [(0, 2927), (-1, 13), (-5, 1)]
TCACCAAAGTTGTTTCTCTCGCTTCCTCCTTCCCTCTCCCCCTTCCCCCCTCTCTTTAGATGGAAGTGAT
TGTTGGGGACTTTGGGATCGTCGTGGTACCCAGGGATGCAGCGGACACAGACCGGATCATGAATCACTCC
TCCATACTCCGCAAGTACAAAGTGAGTCCTCCA , 2553
TCACCAAAGTTGTTTCTCTCGCTTCCTCCTTCCCTCTCCCCCTTCCCCCCTCTCTTTAGATGGAAGTGAT
TGTTGGGGACTTTGGGATCGTCGTGGTACCCAGGGATGCAGCGGACACGGACCGGATCATGAATCACTCC
TCCATACTCCGCAAGTACAAAGTGAGTCCTCCA , 18
TCACCAAAGTTGTTTCTCTCGCTTCCTCCTTCCCTCTCCCCCTTCCCCCCTCTCTTTAGATGGAAGTGAT
TGTTGGGGACTTTGGGATCGTCGTGGTACCCAGGGATGCAGCGGACACAGACCGGATCATGAATCGCTCC
TCCATACTCCGCAAGTACAAAGTGAGTCCTCCA , 10
TCACCAAAGTTGTTTCTCTCGCTTCCTCCTTCCCTCTCCCCCTTCCCCCCTCTCTTTAGATGGAAGTGAT
TGTTGGGGACTGTGGGATCGTCGTGGTACCCAGGGATGCAGCGGACACAGACCGGATCATGAATCACTCC
TCCATACTCCGCAAGTACAAAGTGAGTCCTCCA , 10
TCACCAAAGTTGTTTCTCTCGCTTCCTCCTTCCCTCTCCCCCTTCCCCCCTCTCTTTAGATGGAAGTGAT
TGTTGGGGACGTTGGGATCGTCGTGGTACCCAGGGATGCAGCGGACACAGACCGGATCATGAATCACTCC
TCCATACTCCGCAAGTACAAAGTGAGTCCTCCA , 7
TCACCAAAGTTGTTTCTCTCGCTTCCTCCTTCCCTCTCCCCCTTCCCCCCTCTCTTTAGATGGAAGTGAT
TGTTGGGGACTTTGGGATCGTCGTGGTACCCAGGGATGCAGCGGACACCGACCGGATCATGAATCACTCC
TCCATACTCCGCAAGTACAAAGTGAGTCCTCCA , 5
TCACCAAAGTTGTTTCTCTCGCTTCCTCCTTCCCTCTCCCCCTTCCCCCCTCTCTTTAGATGGAAGTGAT
TGTTGGGGACTTTGGGATCGTCGTGGTACCCAGGGATGCAGCGGACACAGACCGGATCATGAATCACTCA
TCCATACTCCGCAAGTACAAAGTGAGTCCTCCA , 5
TCACCAAAGTTGTTTCTCTCGCTTCCTCCTTCCCTCTCCCCCTTCCCCCCTCTCTTTAGATGGAAGTGAT
TGTTGGGGACTTTGGGATCGTCGTGGTACCCAGGGATGCAGCGGACACAGACCGGATCATGAATCACTCC
TCCATGCTCCGCAAGTACAAAGTGAGTCCTCCA , 5
TCACCAAAGTTGTTTCTCTCGCTTCCTCCTTCCCTCTCCCCCTTCCCCCCTCTCTTTAGATGGAAGTGAT
TGTTGGGGACTTTGGGATCGTCGTGGTACCCAGGGATGCAGCGGACACAGACCGGATCATGAATCACTCC
GCCATACTCCGCAAGTACAAAGTGAGTCCTCCA , 5
TCACCAAAGTTGTTTCTCTCGCTTCCTCCTTCCCTCTCCCCCTTCCCCCCTCTCTTTAGATGGAAGTGAT
TGTTGGGGACTTTGGGATCGTCGTGGTACCCAGGGATGCAGCGGACACAGACCGGATCATGAATCACTCC
TCCATACGCCGCAAGTACAAAGTGAGTCCTCCA , 4
TCACCAAAGTTGTTTCTCTCGCTTCCTCCTTCCCTCTCCCCCTTCCCCCTCTCTTTAGATGGAAGTGATT
GTTGGGGACTTTGGGATCGTCGTGGTACCCAGGGATGCAGCGGACACAGACCGGATCATGAATCACTCCT
CCATACTCCGCAAGTACAAAGTGAGTCCTCCA , 4
TCACCAAAGTTGTTTCTCTCGCTTCCTCCTTCCCTCTCCCCCTTCCCCCTTCTCTTTAGATGGAAGTGAT
TGTTGGGGACTTTGGGATCGTCGTGGTACCCAGGGATGCAGCGGACACAGACCGGATCATGAATCACTCC
TCCATACTCCGCAAGTACAAAGTGAGTCCTCCA , 4

GEIC-Plate01-E04 TOTAL:3316 OrderedDict([('WT_sp1', 3262), ('R232Q', 0), ('Silent Block Mod', 0), ('R232Q (Full ssODN)', 0), ('Silent Block Mod (Full ssODN)', 0)])   [(0, 3305), (-1, 10), (1, 1)]
TCACCAAAGTTGTTTCTCTCGCTTCCTCCTTCCCTCTCCCCCTTCCCCCCTCTCTTTAGATGGAAGTGAT
TGTTGGGGACTTTGGGATCGTCGTGGTACCCAGGGATGCAGCGGACACAGACCGGATCATGAATCACTCC

TCCATACTCCGCAAGTACAAAGTGAGTCCTCCA , 2923
TCACCAAAGTTGTTTCTCTCGCTTCCTCCTTCCCTCTCCCCCTTCCCCCCTCTCTTTAGATGGAAGTGAT
TGTTGGGGACTGTGGGATCGTCGTGGTACCCAGGGATGCAGCGGACACAGACCGGATCATGAATCACTCC
TCCATACTCCGCAAGTACAAAGTGAGTCCTCCA , 13
TCACCAAAGTTGTTTCTCTCGCTTCCTCCTTCCCTCTCCCCCTTCCCCCCTCTCTTTAGATGGAAGTGAT
TGTTGGGGACTTTGGGATCGTCGTGGTACCCAGGGATGCAGCGGACACCGACCGGATCATGAATCACTCC
TCCATACTCCGCAAGTACAAAGTGAGTCCTCCA , 11
TCACCAAAGTTGTTTCTCTCGCTTCCTCCTTCCCTCTCCCCCTTCCCCCCTCTCTTTAGATGGAAGTGAT
TGTTGGGGACTTTGGGATCGTCGTGGTACCCAGGGATGCAGCGGACACGGACCGGATCATGAATCACTCC
TCCATACTCCGCAAGTACAAAGTGAGTCCTCCA , 11
TCACCAAAGTTGTTTCTCTCGCTTCCTCCTTCCCTCTCCCCCTTCCCCCCTCTCTTTAGATGGAAGTGAT
TGTTGGGGACGTTGGGATCGTCGTGGTACCCAGGGATGCAGCGGACACAGACCGGATCATGAATCACTCC
TCCATACTCCGCAAGTACAAAGTGAGTCCTCCA , 9
TCACCAAAGTTGTTTCTCTCGCTTCCTCCTTCCCTCTCCCCCTTCCCCCCTCTCTTTAGATGGAAGTGAT
TGTTGGGGACTTTGGGATCGTCGTGGTACCCAGGGATGCAGCGGACACAGACCGGATCATGAATCGCTCC
TCCATACTCCGCAAGTACAAAGTGAGTCCTCCA , 7
TCACCAAAGTTGTTTCTCTCGCTTCCTCCTTCCCTCTCCCCCTTCCCCCCTCTCTTTAGATGGAAGTGAT
TGTTGGGGACTTTGGGATCGTCGTGGTACCCAGGGATGCAGCGGACACAGACCAGATCATGAATCACTCC
TCCATACTCCGCAAGTACAAAGTGAGTCCTCCA , 6
TCACCAAAGTTGTTTCTCTCGCTTCCTCCTTCCCTCTCCCCCTTCCCCCCTCTCTTTAGATGGAAGTGGT
TGTTGGGGACTTTGGGATCGTCGTGGTACCCAGGGATGCAGCGGACACAGACCGGATCATGAATCACTCC
TCCATACTCCGCAAGTACAAAGTGAGTCCTCCA , 5
TCACCAAAGTTGTTTCTCTCGCTTCCTCCTTCCCTCTCCCCCTTCCCCCCTCTCTTTAGATGGAAGTGAT
TGTTGGGGACTTTGGGATCGTCGTGGTACCCAGGGATGCAGCGGACACAGACCGGATCATGAATCACTCC
TCCATACTCCGCAAGTACCAAGTGAGTCCTCCA , 5
TCACCAAAGTTGTTTCTCTCGCTTCCTCCTTCCCTCTCCCCCTTCCCCCCTCTCTTTAGATGGAAGTGAT
TGTTGGGGACTTTGGGATCGTCGTGGTACCCAGGGATGCAGCGGACACAGACCGGATCATGAATCTCTCC
TCCATACTCCGCAAGTACAAAGTGAGTCCTCCA , 4
TCACCAAAGTTGTTTCTCTCGCTTCCTCCTTCCCTCTCCCCCTTCCCCCTCTCTTTAGATGGAAGTGATT
GTTGGGGACTTTGGGATCGTCGTGGTACCCAGGGATGCAGCGGACACAGACCGGATCATGAATCACTCCT
CCATACTCCGCAAGTACAAAGTGAGTCCTCCA , 4
TCACCAAAGTTGTTTCTCTCGCTTCCTCCTTCCCTCTCCCCCTTCCCCCCTCTCTTTAGATGGAAGTGAT
TGTTGGGGACTTTGGGATCGTCGTGGTACCCAGGGATGCAGCGGACACAGACCGGATCATGAATCACTCC
TCCATGCTCCGCAAGTACAAAGTGAGTCCTCCA , 4

GEIC-Plate01-E05 TOTAL:3732 OrderedDict([('WT_sp1', 1794), ('R232Q', 1868), ('Silent Block Mod', 0), ('R232Q (Full ssODN)', 1727), ('Silent Block Mod (Full ssODN)', 0)])   [(0, 3717), (-1, 14), (1, 1)]
TCACCAAAGTTGTTTCTCTCGCTTCCTCCTTCCCTCTCCCCCTTCCCCCCTCTCTTTAGATGGAAGTGAT
TGTTGGGGACTTTGGGATCGTCGTGGTACCCCAAGATGCAGCGGACACAGACCGGATCATGAATCACTCC
TCCATACTCCGCAAGTACAAAGTGAGTCCTCCA , 1704
TCACCAAAGTTGTTTCTCTCGCTTCCTCCTTCCCTCTCCCCCTTCCCCCCTCTCTTTAGATGGAAGTGAT
TGTTGGGGACTTTGGGATCGTCGTGGTACCCAGGGATGCAGCGGACACAGACCGGATCATGAATCACTCC
TCCATACTCCGCAAGTACAAAGTGAGTCCTCCA , 1600
TCACCAAAGTTGTTTCTCTCGCTTCCTCCTTCCCTCTCCCCCTTCCCCCCTCTCTTTAGATGGAAGTGAT
TGTTGGGGACTTTGGGATCGTCGTGGTACCCAGGGATGCAGCGGACACGGACCGGATCATGAATCACTCC
TCCATACTCCGCAAGTACAAAGTGAGTCCTCCA , 10
TCACCAAAGTTGTTTCTCTCGCTTCCTCCTTCCCTCTCCCCCTTCCCCCCTCTCTTTAGATGGAAGTGAT
TGTTGGGGACTTTGGGATCGTCGTGGTACCCAGGGATGCAGCGGACACAGACCGGATCATGAATCGCTCC

TCCATACTCCGCAAGTACAAAGTGAGTCCTCCA , 8
TCACCAAAGTTGTTTCTCTCGCTTCCTCCTTCCCTCTCCCCCTTCCCCCCTCTCTTTAGATGGAAGTGAT
TGTTGGGGACGTTGGGATCGTCGTGGTACCCCAAGATGCAGCGGACACAGACCGGATCATGAATCACTCC
TCCATACTCCGCAAGTACAAAGTGAGTCCTCCA , 7
TCACCAAAGTTGTTTCTCTCGCTTCCTCCTTCCCTCTCCCCCTTCCCCCCTCTCTTTAGATGGAAGTGAT
TGTTGGGGACTGTGGGATCGTCGTGGTACCCCAAGATGCAGCGGACACAGACCGGATCATGAATCACTCC
TCCATACTCCGCAAGTACAAAGTGAGTCCTCCA , 7
TCACCAAAGTTGTTTCTCTCGCTTCCTCCTTCCCTCTCCCCCTTCCCCCCTCTCTTTAGATGGAAGTGAT
TGTTGGGGACTTTGGGATCGTCGTGGTCCCCCAAGATGCAGCGGACACAGACCGGATCATGAATCACTCC
TCCATACTCCGCAAGTACAAAGTGAGTCCTCCA , 6
TCACCAAAGTTGTTTCTCTCGCTTCCTCCTTCCCTCTCCCCCTTCCCCCCTCTCTTTAGATGGAAGTGAT
TGTTGGGGACGTTGGGATCGTCGTGGTACCCAGGGATGCAGCGGACACAGACCGGATCATGAATCACTCC
TCCATACTCCGCAAGTACAAAGTGAGTCCTCCA , 6
TCACCAAAGTTGTTTCTCTCGCTTCCTCCTTCCCTCTCCCCCTTCCCCCCTCTCTTTAGATGGAAGTGAT
TGTTGGGGACTTTGGGATCGTCGTGGTACCCCAAGATGCAGCGGACACAGACCGGATCATGAATCACTCC
TCCATGCTCCGCAAGTACAAAGTGAGTCCTCCA , 5
TCACCAAAGTTGTTTCTCTCGCTTCCTCCTTCCCTCTCCCCCTTCCCCCCTCTCTTTAGATGGAAGTGAT
TGTTGGGGACTTTGGGATCGTCGTGGTACCCAGGGATGCAGCGGACACAGGCCGGATCATGAATCACTCC
TCCATACTCCGCAAGTACAAAGTGAGTCCTCCA , 4
TCACCAAAGTTGTTTCTCTCGCTTCCTCCTTCCCTCTCCCCCTTCCCCCCTCTCTTTAGATGGAAGTGAT
TGTTGGGGACTTTGGGATCGTCGTGGTACCCAGGGATGCAGCGGACACCGACCGGATCATGAATCACTCC
TCCATACTCCGCAAGTACAAAGTGAGTCCTCCA , 4
TCACCAAAGTTGTTTCTCTCGCTTCCTCCTTCCCTCTCCCCCTTCCCCCCTCTCTTTAGATGGAAGTGAT
TGTTGGGGACTTTGGGATCGTCGTGGTGCCCCAAGATGCAGCGGACACAGACCGGATCATGAATCACTCC
TCCATACTCCGCAAGTACAAAGTGAGTCCTCCA , 4

GEIC-Plate01-E06 TOTAL:4484 OrderedDict([('WT_sp1', 2216), ('R232Q', 0), ('Silent Block Mod', 2146), ('R232Q (Full ssODN)', 0), ('Silent Block Mod (Full ssODN)', 1955)])   [(0, 4461), (-1, 22), (-2, 1)]
TCACCAAAGTTGTTTCTCTCGCTTCCTCCTTCCCTCTCCCCCTTCCCCCCTCTCTTTAGATGGAAGTGAT
TGTTGGGGACTTTGGGATCGTCGTGGTACCCAGGGATGCAGCGGACACAGACCGGATCATGAATCACTCC
TCCATACTCCGCAAGTACAAAGTGAGTCCTCCA , 2003
TCACCAAAGTTGTTTCTCTCGCTTCCTCCTTCCCTCTCCCCCTTCCCCCCTCTCTTTAGATGGAAGTGAT
TGTTGGGGACTTTGGGATCGTCGTGGTACCCCGTGATGCAGCGGACACAGACCGGATCATGAATCACTCC
TCCATACTCCGCAAGTACAAAGTGAGTCCTCCA , 1933
TCACCAAAGTTGTTTCTCTCGCTTCCTCCTTCCCTCTCCCCCTTCCCCCCTCTCTTTAGATGGAAGTGAT
TGTTGGGGACTGTGGGATCGTCGTGGTACCCAGGGATGCAGCGGACACAGACCGGATCATGAATCACTCC
TCCATACTCCGCAAGTACAAAGTGAGTCCTCCA , 11
TCACCAAAGTTGTTTCTCTCGCTTCCTCCTTCCCTCTCCCCCTTCCCCCCTCTCTTTAGATGGAAGTGAT
TGTTGGGGACTTTGGGATCGTCGTGGTACCCCGTGATGCAGCGGACACAGACCGGATCATGAATCGCTCC
TCCATACTCCGCAAGTACAAAGTGAGTCCTCCA , 10
TCACCAAAGTTGTTTCTCTCGCTTCCTCCTTCCCTCTCCCCCTTCCCCCCTCTCTTTAGATGGAAGTGAT
TGTTGGGGACTTTGGGATCGTCGTGGTACCCAGGGATGCAGCGGACACGGACCGGATCATGAATCACTCC
TCCATACTCCGCAAGTACAAAGTGAGTCCTCCA , 10
TCACCAAAGTTGTTTCTCTCGCTTCCTCCTTCCCTCTCCCCCTTCCCCCCTCTCTTTAGATGGAAGTGAT
TGTTGGGGACGTTGGGATCGTCGTGGTACCCAGGGATGCAGCGGACACAGACCGGATCATGAATCACTCC
TCCATACTCCGCAAGTACAAAGTGAGTCCTCCA , 8
TCACCAAAGTTGTTTCTCTCGCTTCCTCCTTCCCTCTCCCCCTTCCCCCCTCTCTTTAGATGGAAGTGAT
TGTTGGGGACTGTGGGATCGTCGTGGTACCCCGTGATGCAGCGGACACAGACCGGATCATGAATCACTCC

TCCATACTCCGCAAGTACAAAGTGAGTCCTCCA , 7
TCACCAAAGTTGTTTCTCTCGCTTCCTCCTTCCCTCTCCCCCTTCCCCCCTCTCTTTAGATGGAAGTGAT
TGTTGGGGACGTTGGGATCGTCGTGGTACCCCGTGATGCAGCGGACACAGACCGGATCATGAATCACTCC
TCCATACTCCGCAAGTACAAAGTGAGTCCTCCA , 6
TCACCAAAGTTGTTTCTCTCGCTTCCTCCTTCCCTCTCCCCCTTCCCCCCTCTCTTTAGATGGAAGTGAT
TGTTGGGGACTTTGGGATCGTCGTTGTACCCAGGGATGCAGCGGACACAGACCGGATCATGAATCACTCC
TCCATACTCCGCAAGTACAAAGTGAGTCCTCCA , 4
TCACCAAAGTTGTTTCTCTCGCTTCCTCCTTCCCTCTCCCCCTTCCCCCCTCTCTTTAGATGGAAGTGAT
TGTTGGGGACTTTGGGATCGTCGTGGTACCCCGTGATGCAGCGGACACAGACCAGATCATGAATCACTCC
TCCATACTCCGCAAGTACAAAGTGAGTCCTCCA , 4
TCACCAAAGTTGTTTCTCTCGCTTCCTCCTTCCCTCTCCCCCTTCCCCCCTCTCTTTAGATGGAAGTGAT
TGTTGGGGACTTTGGGATCGTCGCGGTACCCCGTGATGCAGCGGACACAGACCGGATCATGAATCACTCC
TCCATACTCCGCAAGTACAAAGTGAGTCCTCCA , 4
TCACCAAAGTTGTTTCTCTCGCTTCCTCCTTCCCTCTCCCCCTTCCCCCCTCTCTTTAGATGGAAGTGAT
TGTTGGGGACTTTGGGATCGTCGTGGTACCCAGGGATGCAGCGGACACAGACCGGATCATGAATCGCTCC
TCCATACTCCGCAAGTACAAAGTGAGTCCTCCA , 4

GEIC–Plate01–E07 TOTAL:4182 OrderedDict([('WT_sp1', 4122), ('R232Q', 0), ('Silent Block Mod', 1), ('R232Q (Full ssODN)', 0), ('Silent Block Mod (Full ssODN)', 1)])   [(0, 4167), (–1, 14), (1, 1)]
TCACCAAAGTTGTTTCTCTCGCTTCCTCCTTCCCTCTCCCCCTTCCCCCCTCTCTTTAGATGGAAGTGAT
TGTTGGGGACTTTGGGATCGTCGTGGTACCCAGGGATGCAGCGGACACAGACCGGATCATGAATCACTCC
TCCATACTCCGCAAGTACAAAGTGAGTCCTCCA , 3715
TCACCAAAGTTGTTTCTCTCGCTTCCTCCTTCCCTCTCCCCCTTCCCCCCTCTCTTTAGATGGAAGTGAT
TGTTGGGGACTTTGGGATCGTCGTGGTACCCAGGGATGCAGCGGACACGGACCGGATCATGAATCACTCC
TCCATACTCCGCAAGTACAAAGTGAGTCCTCCA , 28
TCACCAAAGTTGTTTCTCTCGCTTCCTCCTTCCCTCTCCCCCTTCCCCCCTCTCTTTAGATGGAAGTGAT
TGTTGGGGACTTTGGGATCGTCGTGGTACCCAGGGATGCAGCGGACACAGACCGGATCATGAATCACTCC
TCCATACGCCGCAAGTACAAAGTGAGTCCTCCA , 10
TCACCAAAGTTGTTTCTCTCGCTTCCTCCTTCCCTCTCCCCCTTCCCCCCTCTCTTTAGATGGAAGTGAT
TGTTGGGGACTTTGGGATCGTCGTGGTACCCAGGGATGCAGCGGACACAGGCCGGATCATGAATCACTCC
TCCATACTCCGCAAGTACAAAGTGAGTCCTCCA , 9
TCACCAAAGTTGTTTCTCTCGCTTCCTCCTTCCCTCTCCCCCTTCCCCCCTCTCTTTAGATGGAAGTGAT
TGTTGGGGACTTTGGGATCGTCGTGGTACCCAGGGATGCAGCGGACACCGACCGGATCATGAATCACTCC
TCCATACTCCGCAAGTACAAAGTGAGTCCTCCA , 9
TCACCAAAGTTGTTTCTCTCGCTTCCTCCTTCCCTCTCCCCCTTCCCCCCTCTCTTTAGATGGAAGTGAT
TGTTGGGGACTGTGGGATCGTCGTGGTACCCAGGGATGCAGCGGACACAGACCGGATCATGAATCACTCC
TCCATACTCCGCAAGTACAAAGTGAGTCCTCCA , 9
TCACCAAAGTTGTTTCTCTCGCTTCCTCCTTCCCTCTCCCCCTTCCCCCCTCTCTTTAGATGGAAGTGAT
TGTTGGGGACTTTGGGATCGTCGTGGTACCCAGGGATGCAGCGGACACAGACCGGATCATGAATCGCTCC
TCCATACTCCGCAAGTACAAAGTGAGTCCTCCA , 8
TCACCAAAGTTGTTTCTCTCGCTTCCTCCTTCCCTCTCCCCCTTCCCCCCTCTCTTTAGATGGAAGTGAT
TGTTGGGGACGTTGGGATCGTCGTGGTACCCAGGGATGCAGCGGACACAGACCGGATCATGAATCACTCC
TCCATACTCCGCAAGTACAAAGTGAGTCCTCCA , 7
TCACCAAAGTTGTTTCTCTCGCTTCCTCCTTCCCTCTCCCCCTTCCCCCCTCTCTTTAGATGGAAGTGAT
TGTTGGGGACTTTGGGATCGTCGTGGTACCCAGGGATGCAGCGGACACAGACCAGATCATGAATCACTCC
TCCATACTCCGCAAGTACAAAGTGAGTCCTCCA , 7
TCACCAAAGTTGTTTCTCTCGCTTCCTCCTTCCCTCTCCCCCTTCCCCCCTCTCTTTAGATGGAAGTGAT
TGTTGGGGACTTTGGGATCGTCGTGGTACCCAGGGATGCAGCGGACACAGACCGGATCATGAATCACTCC

GCCATACTCCGCAAGTACAAAGTGAGTCCTCCA , 5
TCACCAAAGTTGTTTCTCTCGCTTCCTCCTTCCCTCTCCCCCTTCCCCCTTCTCTTTAGATGGAAGTGAT
TGTTGGGGACTTTGGGATCGTCGTGGTACCCAGGGATGCAGCGGACACAGACCGGATCATGAATCACTCC
TCCATACTCCGCAAGTACAAAGTGAGTCCTCCA , 5
TCACCAAAGTTGTTTCTCTCGCTTCCTCCTTCCCTCTCCCCCTTCCCCCCTCTCTTTAGATGGAAGTGAT
TGTTGGGGACTTTGTGATCGTCGTGGTACCCAGGGATGCAGCGGACACAGACCGGATCATGAATCACTCC
TCCATACTCCGCAAGTACAAAGTGAGTCCTCCA , 5

GEIC-Plate01-E08 TOTAL:3724 OrderedDict([('WT_sp1', 3664), ('R232Q', 6), ('Silent Block Mod', 0), ('R232Q (Full ssODN)', 5), ('Silent Block Mod (Full ssODN)', 0)])   [(0, 3708), (-1, 13), (1, 3)]
TCACCAAAGTTGTTTCTCTCGCTTCCTCCTTCCCTCTCCCCCTTCCCCCCTCTCTTTAGATGGAAGTGAT
TGTTGGGGACTTTGGGATCGTCGTGGTACCCAGGGATGCAGCGGACACAGACCGGATCATGAATCACTCC
TCCATACTCCGCAAGTACAAAGTGAGTCCTCCA , 3274
TCACCAAAGTTGTTTCTCTCGCTTCCTCCTTCCCTCTCCCCCTTCCCCCCTCTCTTTAGATGGAAGTGAT
TGTTGGGGACTTTGGGATCGTCGTGGTACCCAGGGATGCAGCGGACACGGACCGGATCATGAATCACTCC
TCCATACTCCGCAAGTACAAAGTGAGTCCTCCA , 20
TCACCAAAGTTGTTTCTCTCGCTTCCTCCTTCCCTCTCCCCCTTCCCCCCTCTCTTTAGATGGAAGTGAT
TGTTGGGGACTGTGGGATCGTCGTGGTACCCAGGGATGCAGCGGACACAGACCGGATCATGAATCACTCC
TCCATACTCCGCAAGTACAAAGTGAGTCCTCCA , 14
TCACCAAAGTTGTTTCTCTCGCTTCCTCCTTCCCTCTCCCCCTTCCCCCCTCTCTTTAGATGGAAGTGAT
TGTTGGGGACTTTGGGATCGTCGTGGTACCCAGGGATGCAGCGGACACAGACCGGATCATGAATCGCTCC
TCCATACTCCGCAAGTACAAAGTGAGTCCTCCA , 12
TCACCAAAGTTGTTTCTCTCGCTTCCTCCTTCCCTCTCCCCCTTCCCCCCTCTCTTTAGATGGAAGTGAT
TGTTGGGGACTTTGGGATCGTCGTGGTACCCAGGGATGCAGCGGACACCGACCGGATCATGAATCACTCC
TCCATACTCCGCAAGTACAAAGTGAGTCCTCCA , 8
TCACCAAAGTTGTTTCTCTCGCTTCCTCCTTCCCTCTCCCCCTTCCCCCCTCTCTTTAGATGGAAGTGAT
TGTTGGGGACTTTGGGATCGTCGTGGTACCCAGGGATGCAGCGGACACAGACCGGATCATGAATCACTCC
TCCATGCTCCGCAAGTACAAAGTGAGTCCTCCA , 6
TCACCAAAGTTGTTTCTCTCGCTTCCTCCTTCCCTCTCCCCCTTCCCCCTCTCTTTAGATGGAAGTGATT
GTTGGGGACTTTGGGATCGTCGTGGTACCCAGGGATGCAGCGGACACAGACCGGATCATGAATCACTCCT
CCATACTCCGCAAGTACAAAGTGAGTCCTCCA , 6
TCACCAAAGTTGTTTCTCTCGCTTCCTCCTTCCCTCTCCCCCTTCCCCCCTCTCTTTAGATGGAAGTGAT
TGTTGGGGACTTTGGGATCGTCGTGGTACCCAGGGATGCGGCGGACACAGACCGGATCATGAATCACTCC
TCCATACTCCGCAAGTACAAAGTGAGTCCTCCA , 6
TCACCAAAGTTGTTTCTCTCGCTTCCTCCTTCCCTCTCCCCCTTCCCCCCTCTCTTTAGATGGAAGTGAT
TGTTGGGGACTTTGGGATCGTCGTGGTACCCAGGGATGCAGCAGACACAGACCGGATCATGAATCACTCC
TCCATACTCCGCAAGTACAAAGTGAGTCCTCCA , 6
TCACCAAAGTTGTTTCTCTCGCTTCCTCCTTCCCTCTCCCCCTTCCCCCCTCTCTTTAGATGGAAGTGAT
TGTTGGGGACTTTGGGATCGTCGTGGTACCCCAAGATGCAGCGGACACAGACCGGATCATGAATCACTCC
TCCATACTCCGCAAGTACAAAGTGAGTCCTCCA , 5
TCACCAAAGTTGTTTCTCTCGCTTCCTCCTTCCCTCTCCCCCTTCCCCCCTCTCTTTAGATGGAAGTGAT
TGTTGGGGACTTTGGGATCGTCGTGGTACCCAGGGATGCAGCGGACACAGGCCGGATCATGAATCACTCC
TCCATACTCCGCAAGTACAAAGTGAGTCCTCCA , 4
TCACCAAAGTTGTTTCTCTCGCTTCCTCCTTCCCTCTCCCCCTTCCCCCCTCTCTTTAGATGGAAGTGAT
TGTTGGGGACTCTGGGATCGTCGTGGTACCCAGGGATGCAGCGGACACAGACCGGATCATGAATCACTCC
TCCATACTCCGCAAGTACAAAGTGAGTCCTCCA , 4

GEIC–Plate01–F01 TOTAL:3361 OrderedDict([('WT_sp1', 1659), ('R232Q', 1620), ('Silent Block Mod', 0), ('R232Q (Full ssODN)', 1476), ('Silent Block Mod (Full ssODN)', 0)])   [(0, 3347), (-1, 12), (1, 1), (-2, 1)]
TCACCAAAGTTGTTTCTCTCGCTTCCTCCTTCCCTCTCCCCCTTCCCCCCTCTCTTTAGATGGAAGTGAT
TGTTGGGGACTTTGGGATCGTCGTGGTACCCAGGGATGCAGCGGACACAGACCGGATCATGAATCACTCC
TCCATACTCCGCAAGTACAAAGTGAGTCCTCCA , 1474
TCACCAAAGTTGTTTCTCTCGCTTCCTCCTTCCCTCTCCCCCTTCCCCCCTCTCTTTAGATGGAAGTGAT
TGTTGGGGACTTTGGGATCGTCGTGGTACCCCAAGATGCAGCGGACACAGACCGGATCATGAATCACTCC
TCCATACTCCGCAAGTACAAAGTGAGTCCTCCA , 1460
TCACCAAAGTTGTTTCTCTCGCTTCCTCCTTCCCTCTCCCCCTTCCCCCCTCTCTTTAGATGGAAGTGAT
TGTTGGGGACTTTGGGATCGTCGTGGTACCCCAAGATGCAGCGGACACAGACCGGATCATGAATCGCTCC
TCCATACTCCGCAAGTACAAAGTGAGTCCTCCA , 7
TCACCAAAGTTGTTTCTCTCGCTTCCTCCTTCCCTCTCCCCCTTCCCCCCTCTCTTTAGATGGAAGTGAT
TGTTGGGGACTTTGGGATCGTCGTGGTACCCAGGGATGCAGCGGACACGGACCGGATCATGAATCACTCC
TCCATACTCCGCAAGTACAAAGTGAGTCCTCCA , 6
TCACCAAAGTTGTTTCTCTCGCTTCCTCCTTCCCTCTCCCCCTTCCCCCCTCTCTTTAGATGGAAGTGAT
TGTTGGGGACTTTGGGATCGTCGTGGTACCCAGGGATGCAGCGGACACAGACCGGATCATGAATCGCTCC
TCCATACTCCGCAAGTACAAAGTGAGTCCTCCA , 5
TCACCAAAGTTGTTTCTCTCGCTTCCTCCTTCCCTCTCCCCCTTCCCCCCTCTCTTTAGATGGAAGTGAT
TGTTGGGGACTTTGGGATCGTCGTGGTACCCAGGGATGCAGCGGACACCGACCGGATCATGAATCACTCC
TCCATACTCCGCAAGTACAAAGTGAGTCCTCCA , 5
TCACCAAAGTTGTTTCTCTCGCTTCCTCCTTCCCTCTCCCCCTTCCCCCCTCTCTTTAGATGGAAGTGAT
TGTTGGGGACGTTGGGATCGTCGTGGTACCCAGGGATGCAGCGGACACAGACCGGATCATGAATCACTCC
TCCATACTCCGCAAGTACAAAGTGAGTCCTCCA , 5
TCACCAAAGTTGTTTCTCTCGCTTCCTCCTTCCCTCTCCCCCTTCCCCCCTCTCTTTAGATGGAAGTGAT
TGTTGGGGACTGTGGGATCGTCGTGGTACCCCAAGATGCAGCGGACACAGACCGGATCATGAATCACTCC
TCCATACTCCGCAAGTACAAAGTGAGTCCTCCA , 5
TCACCAAAGTTGTTTCTCTCGCTTCCTCCTTCCCTCTCCCCCTTCCCCCCTCTCTTTAGATGGAAGTGAT
TGTTGGGGACTTTGGGATCGTCGTGGTACCTCAAGATGCAGCGGACACAGACCGGATCATGAATCACTCC
TCCATACTCCGCAAGTACAAAGTGAGTCCTCCA , 5
TCACCAAAGTTGTTTCTCTCGCTTCCTCCTTCCCTCTCCCCCTTCCCCCCTCTCTTTAGATGGAAGTGAT
TGTTGGGGACTATGGGATCGTCGTGGTACCCAGGGATGCAGCGGACACAGACCGGATCATGAATCACTCC
TCCATACTCCGCAAGTACAAAGTGAGTCCTCCA , 4
TCACCAAAGTTGTTTCTCTCGCTTCCTCCTTCCCTCTCCCCCTTCCCCCCTCTCTTTAGATGGAAGTGAG
TGTTGGGGACTTTGGGATCGTCGTGGTACCCCAAGATGCAGCGGACACAGACCGGATCATGAATCACTCC
TCCATACTCCGCAAGTACAAAGTGAGTCCTCCA , 4
TCACCAAAGTTGTTTCTCTCGCTTCCTCCTTCCCTCTCCCCCTTCCCCCCTCTCTTTAGATGGAAGTGAT
TGTTGGGGACTTTGGGATCGTCGTGGTACCCCAAGATGCAGCGGACACAGACCAGATCATGAATCACTCC
TCCATACTCCGCAAGTACAAAGTGAGTCCTCCA , 4

GEIC–Plate01–F02 TOTAL:2679 OrderedDict([('WT_sp1', 1293), ('R232Q', 0), ('Silent Block Mod', 1319), ('R232Q (Full ssODN)', 0), ('Silent Block Mod (Full ssODN)', 1177)])   [(0, 2671), (-1, 6), (2, 1), (-2, 1)]
TCACCAAAGTTGTTTCTCTCGCTTCCTCCTTCCCTCTCCCCCTTCCCCCCTCTCTTTAGATGGAAGTGAT
TGTTGGGGACTTTGGGATCGTCGTGGTACCCCGTGATGCAGCGGACACAGACCGGATCATGAATCACTCC
TCCATACTCCGCAAGTACAAAGTGAGTCCTCCA , 1161
TCACCAAAGTTGTTTCTCTCGCTTCCTCCTTCCCTCTCCCCCTTCCCCCCTCTCTTTAGATGGAAGTGAT

TGTTGGGGACTTTGGGATCGTCGTGGTACCCAGGGATGCAGCGGACACAGACCGGATCATGAATCACTCC
TCCATACTCCGCAAGTACAAAGTGAGTCCTCCA , 1148
TCACCAAAGTTGTTTCTCTCGCTTCCTCCTTCCCTCTCCCCCTTCCCCCCTCTCTTTAGATGGAAGTGAT
TGTTGGGGACGTTGGGATCGTCGTGGTACCCCGTGATGCAGCGGACACAGACCGGATCATGAATCACTCC
TCCATACTCCGCAAGTACAAAGTGAGTCCTCCA , 10
TCACCAAAGTTGTTTCTCTCGCTTCCTCCTTCCCTCTCCCCCTTCCCCCCTCTCTTTAGATGGAAGTGAT
TGTTGGGGACGTTGGGATCGTCGTGGTACCCAGGGATGCAGCGGACACAGACCGGATCATGAATCACTCC
TCCATACTCCGCAAGTACAAAGTGAGTCCTCCA , 9
TCACCAAAGTTGTTTCTCTCGCTTCCTCCTTCCCTCTCCCCCTTCCCCCCTCTCTTTAGATGGAAGTGAT
TGTTGGGGACTGTGGGATCGTCGTGGTACCCCGTGATGCAGCGGACACAGACCGGATCATGAATCACTCC
TCCATACTCCGCAAGTACAAAGTGAGTCCTCCA , 7
TCACCAAAGTTGTTTCTCTCGCTTCCTCCTTCCCTCTCCCCCTTCCCCCCTCTCTTTAGATGGAAGTGAT
TGTTGGGGACTTTGGGATCGTCGTGGTACCCAGGGATGCAGCGGACACGGACCGGATCATGAATCACTCC
TCCATACTCCGCAAGTACAAAGTGAGTCCTCCA , 6
TCACCAAAGTTGTTTCTCTCGCTTCCTCCTTCCCTCTCCCCCTTCCCCCCTCTCTTTAGATGGAAGTGAT
TGTTGGGGACTTTGGGATCGTCGTGGTACCCCGTGATGCAGCGGACACAGACCGGATCATGAATCACTCC
GCCATACTCCGCAAGTACAAAGTGAGTCCTCCA , 6
TCACCAAAGTTGTTTCTCTCGCTTCCTCCTTCCCTCTCCCCCTTCCCCCCTCTCTTTAGATGGAAGTGAT
TGTTGGGGACTTTGGGATCGTCGTGGTACCCCGTGATGCAGCGGACACAGACCGGATCATGAATCGCTCC
TCCATACTCCGCAAGTACAAAGTGAGTCCTCCA , 4
TCACCAAAGTTGTTTCTCTCGCTTCCTCCTTCCCTCTCCCCCTTCCCCCCTCTCTTTAGATGGAAGTGAT
TGTTGGGGACTTTGGGATCGTCGTGGTACCCAGGGATGCAGCGGACACAGACCGGATCATGAATCACTCC
GCCATACTCCGCAAGTACAAAGTGAGTCCTCCA , 4
TCACCAAAGTTGTTTCTCTCGCTTCCTCCTTCCCTCTCCCCCTTCCCCCCTCTCTTTAGATGGAAGTGAT
TGTTGGGGACTTTGGGATCGTCGTGGTGCCCCGTGATGCAGCGGACACAGACCGGATCATGAATCACTCC
TCCATACTCCGCAAGTACAAAGTGAGTCCTCCA , 3
TCACCAAAGTTGTTTCTCTCGCTTCCTCCTTCCCTCTCCCCCTTCCCCCCTCTCTTTAGATGGAAGTGAT
TGTTGGGGACTTTGGGATCGTCGTGGTACCCAGGGATGCAGCGGACACAGACCGGATCATGAATCACTCC
TCCATGCTCCGCAAGTACAAAGTGAGTCCTCCA , 3
TCACCAAAGTTGTTTCTCTCGCTTCCTCCTTCCCTCTCCCCCTTCCCCCCTCTCTTTAGATGGAAGTGAT
TGTTGGGGACTTTGGGATCGTCGTGGTACCCCGTGATGCAGCGGATACAGACCGGATCATGAATCACTCC
TCCATACTCCGCAAGTACAAAGTGAGTCCTCCA , 3

GEIC-Plate01-F03 TOTAL:2861 OrderedDict([('WT_sp1', 2813), ('R232Q', 0), ('Silent Block Mod', 0), ('R232Q (Full ssODN)', 0), ('Silent Block Mod (Full ssODN)', 0)])   [(0, 2845), (-1, 14), (1, 1), (2, 1)]
TCACCAAAGTTGTTTCTCTCGCTTCCTCCTTCCCTCTCCCCCTTCCCCCCTCTCTTTAGATGGAAGTGAT
TGTTGGGGACTTTGGGATCGTCGTGGTACCCAGGGATGCAGCGGACACAGACCGGATCATGAATCACTCC
TCCATACTCCGCAAGTACAAAGTGAGTCCTCCA , 2482
TCACCAAAGTTGTTTCTCTCGCTTCCTCCTTCCCTCTCCCCCTTCCCCCCTCTCTTTAGATGGAAGTGAT
TGTTGGGGACTTTGGGATCGTCGTGGTACCCAGGGATGCAGCGGACACGGACCGGATCATGAATCACTCC
TCCATACTCCGCAAGTACAAAGTGAGTCCTCCA , 16
TCACCAAAGTTGTTTCTCTCGCTTCCTCCTTCCCTCTCCCCCTTCCCCCCTCTCTTTAGATGGAAGTGAT
TGTTGGGGACTGTGGGATCGTCGTGGTACCCAGGGATGCAGCGGACACAGACCGGATCATGAATCACTCC
TCCATACTCCGCAAGTACAAAGTGAGTCCTCCA , 14
TCACCAAAGTTGTTTCTCTCGCTTCCTCCTTCCCTCTCCCCCTTCCCCCCTCTCTTTAGATGGAAGTGAT
TGTTGGGGACTTTGGGATCGTCGTGGTACCCAGGGATGCAGCGGACACAGACCGGATCATGAATCGCTCC
TCCATACTCCGCAAGTACAAAGTGAGTCCTCCA , 10
TCACCAAAGTTGTTTCTCTCGCTTCCTCCTTCCCTCTCCCCCTTCCCCCCTCTCTTTAGATGGAAGTGAT

TGTTGGGGACTTTGGGATCGTCGTGGTACCCAGGGATGCAGCGGACACAGACCGGATCATGAATCACTCC
TCCATGCTCCGCAAGTACAAAGTGAGTCCTCCA , 7
TCACCAAAGTTGTTTCTCTCGCTTCCTCCTTCCCTCTCCCCCTTCCCCCCTCTCTTTAGATGGAAGTGAT
TGTTGGGGACGTTGGGATCGTCGTGGTACCCAGGGATGCAGCGGACACAGACCGGATCATGAATCACTCC
TCCATACTCCGCAAGTACAAAGTGAGTCCTCCA , 6
TCACCAAAGTTGTTTCTCTCGCTTCCTCCTTCCCTCTCCCCCTTCCCCCCTCTCTTTAGATGGAAGTGAT
TGTTGGGGACTTTGGGATCGTCGTGGTACCCAGGGATGCAGCGGACACAGACCGGATCATGAATCACGCC
TCCATACTCCGCAAGTACAAAGTGAGTCCTCCA , 5
TCACCAAAGTTGTTTCTCTCGCTTCCTCCTTCCCTCTCCCCCTTCCCCCCTCTCTTTAGATGGAAGTGAT
TGTTGGGGACTTTGGGATCGTCGTGGTACCCAGGGATGCAGCGGACACAGACCGGATCATGAATCACTCC
TCCATACGCCGCAAGTACAAAGTGAGTCCTCCA , 4
TCACCAAAGTTGTTTCTCTCGCTTCCTCCTTCCCTCTCCCCCTTCCCCCCTCTCCTTAGATGGAAGTGAT
TGTTGGGGACTTTGGGATCGTCGTGGTACCCAGGGATGCAGCGGACACAGACCGGATCATGAATCACTCC
TCCATACTCCGCAAGTACAAAGTGAGTCCTCCA , 4
TCACCAAAGTTGTTTCTCTCGCTTCCTCCTTCCCTCTCCCCCTTCCCCCCTCTCTTTAGATGGAAGTGAT
TGTTGGGGACTTTGGGATCGTCGTGGTACCCAGGGATGCAGCAGACACAGACCGGATCATGAATCACTCC
TCCATACTCCGCAAGTACAAAGTGAGTCCTCCA , 4
TCACCAAAGTTGTTTCTCTCGCTTCCTCCTTCCCTCTCCCCCTTCCCCCCTCTCTTTAGATAGAAGTGAT
TGTTGGGGACTTTGGGATCGTCGTGGTACCCAGGGATGCAGCGGACACAGACCGGATCATGAATCACTCC
TCCATACTCCGCAAGTACAAAGTGAGTCCTCCA , 4
TCACCAAAGTTGTTTCTCTCGCTTCCTCCTTCCCTCTCCCCCTTCCCCCCTCTCTTTAGATGGAAGTGAT
TGTTGGGGACTTTGGGATCGTCGTGGTACCCAGGGATGCAGCGGACACAGACCGGATCATGAATCACTCC
TCCATACTCCGCAGGTACAAAGTGAGTCCTCCA , 3

GEIC-Plate01-F04 TOTAL:3528 OrderedDict([('WT_sp1', 3452), ('R232Q', 0), ('Silent Block Mod', 0), ('R232Q (Full ssODN)', 0), ('Silent Block Mod (Full ssODN)', 0)])   [(0, 3513), (-1, 14), (1, 1)]
TCACCAAAGTTGTTTCTCTCGCTTCCTCCTTCCCTCTCCCCCTTCCCCCCTCTCTTTAGATGGAAGTGAT
TGTTGGGGACTTTGGGATCGTCGTGGTACCCAGGGATGCAGCGGACACAGACCGGATCATGAATCACTCC
TCCATACTCCGCAAGTACAAAGTGAGTCCTCCA , 3062
TCACCAAAGTTGTTTCTCTCGCTTCCTCCTTCCCTCTCCCCCTTCCCCCCTCTCTTTAGATGGAAGTGAT
TGTTGGGGACGTTGGGATCGTCGTGGTACCCAGGGATGCAGCGGACACAGACCGGATCATGAATCACTCC
TCCATACTCCGCAAGTACAAAGTGAGTCCTCCA , 18
TCACCAAAGTTGTTTCTCTCGCTTCCTCCTTCCCTCTCCCCCTTCCCCCCTCTCTTTAGATGGAAGTGAT
TGTTGGGGACTTTGGGATCGTCGTGGTACCCAGGGATGCAGCGGACACGGACCGGATCATGAATCACTCC
TCCATACTCCGCAAGTACAAAGTGAGTCCTCCA , 15
TCACCAAAGTTGTTTCTCTCGCTTCCTCCTTCCCTCTCCCCCTTCCCCCCTCTCTTTAGATGGAAGTGAT
TGTTGGGGACTGTGGGATCGTCGTGGTACCCAGGGATGCAGCGGACACAGACCGGATCATGAATCACTCC
TCCATACTCCGCAAGTACAAAGTGAGTCCTCCA , 12
TCACCAAAGTTGTTTCTCTCGCTTCCTCCTTCCCTCTCCCCCTTCCCCCCTCTCTTTAGATGGAAGTGAT
TGTTGGGGACTTTGGGATCGTCGTGGTACCCAGGGATGCAGCGGACACAGACCGGATCATGAATCGCTCC
TCCATACTCCGCAAGTACAAAGTGAGTCCTCCA , 10
TCACCAAAGTTGTTTCTCTCGCTTCCTCCTTCCCTCTCCCCCTTCCCCCCTCTCTTTAGATGGAAGTGAT
TGTTGGGGACTTTGGGATCGTCGTGGTACCCAGGGATGCAGCGGACACAGACCGGATCATGAATCACTCC
TCCATACTCCGCAAGTACCAAGTGAGTCCTCCA , 9
TCACCAAAGTTGTTTCTCTCGCTTCCTCCTTCCCTCTCCCCCTTCCCCCCTCTCTTTAGATGGAAGTGAT
TGTTGGGGACTTTGGGATCGTCGTGGTACCCAGGGATGCAGCGGACACCGACCGGATCATGAATCACTCC
TCCATACTCCGCAAGTACAAAGTGAGTCCTCCA , 7
TCACCAAAGTTGTTTCTCTCGCTTCCTCCTTCCCTCTCCCCCTTCCCCCCTCCCTTTAGATGGAAGTGAT

TGTTGGGGACTTTGGGATCGTCGTGGTACCCAGGGATGCAGCGGACACAGACCGGATCATGAATCACTCC
TCCATACTCCGCAAGTACAAAGTGAGTCCTCCA , 6
TCACCAAAGTTGTTTCTCTCGCTTCCTCCTTCCCTCTCCCCCTTCCCCCCTCTCTTTAGATGGAAGTGAT
TGTTGGGGACTTTGGGATCGTCGTGGTACCCAGGGATGCAGCGGACACAGACCGGATCATGAATCACTCC
GCCATACTCCGCAAGTACAAAGTGAGTCCTCCA , 6
TCACCAAAGTTGTTTCTCTCGCTTCCTCCTTCCCTCTCCCCCTTCCCCCCTCTCTTTAGATGGAAGTGAT
TGTTGGGGACTTTGGGATCGTCGTGGTACCCAGGGATGCAGCGGACACAGACCGGATCATGAATCACTCC
TCCATACGCCGCAAGTACAAAGTGAGTCCTCCA , 5
TCACCAAAGTTGTTTCTCTCGCTTCCTCCTTCCCTCTCCCCCTTCCCCCCTCTCTTTAGATGGAAGTGAT
TGTTGGGGACTTTGGGATCGTCGTGGTCCCCAGGGATGCAGCGGACACAGACCGGATCATGAATCACTCC
TCCATACTCCGCAAGTACAAAGTGAGTCCTCCA , 5
TCACCAAAGTTGTTTCTCTCGCTTCCTCCTTCCCTCTCCCCCTTCCCCCCTCTCTTTAGATGGAAGTGAT
TGTTGGGGACTTTGGGATCGTCGTGGTACCCAGGGATGCGGCGGACACAGACCGGATCATGAATCACTCC
TCCATACTCCGCAAGTACAAAGTGAGTCCTCCA , 5

GEIC-Plate01-F05 TOTAL:3841 OrderedDict([('WT_sp1', 1903), ('R232Q', 1861), ('Silent Block Mod', 1), ('R232Q (Full ssODN)', 1688), ('Silent Block Mod (Full ssODN)', 1)])   [(0, 3827), (-1, 14)]
TCACCAAAGTTGTTTCTCTCGCTTCCTCCTTCCCTCTCCCCCTTCCCCCCTCTCTTTAGATGGAAGTGAT
TGTTGGGGACTTTGGGATCGTCGTGGTACCCAGGGATGCAGCGGACACAGACCGGATCATGAATCACTCC
TCCATACTCCGCAAGTACAAAGTGAGTCCTCCA , 1694
TCACCAAAGTTGTTTCTCTCGCTTCCTCCTTCCCTCTCCCCCTTCCCCCCTCTCTTTAGATGGAAGTGAT
TGTTGGGGACTTTGGGATCGTCGTGGTACCCCAAGATGCAGCGGACACAGACCGGATCATGAATCACTCC
TCCATACTCCGCAAGTACAAAGTGAGTCCTCCA , 1665
TCACCAAAGTTGTTTCTCTCGCTTCCTCCTTCCCTCTCCCCCTTCCCCCCTCTCTTTAGATGGAAGTGAT
TGTTGGGGACTTTGGGATCGTCGTGGTACCCAGGGATGCAGCGGACACGGACCGGATCATGAATCACTCC
TCCATACTCCGCAAGTACAAAGTGAGTCCTCCA , 13
TCACCAAAGTTGTTTCTCTCGCTTCCTCCTTCCCTCTCCCCCTTCCCCCCTCTCTTTAGATGGAAGTGAT
TGTTGGGGACTGTGGGATCGTCGTGGTACCCAGGGATGCAGCGGACACAGACCGGATCATGAATCACTCC
TCCATACTCCGCAAGTACAAAGTGAGTCCTCCA , 11
TCACCAAAGTTGTTTCTCTCGCTTCCTCCTTCCCTCTCCCCCTTCCCCCCTCTCTTTAGATGGAAGTGAT
TGTTGGGGACGTTGGGATCGTCGTGGTACCCCAAGATGCAGCGGACACAGACCGGATCATGAATCACTCC
TCCATACTCCGCAAGTACAAAGTGAGTCCTCCA , 7
TCACCAAAGTTGTTTCTCTCGCTTCCTCCTTCCCTCTCCCCCTTCCCCCCTCTCTTTAGATGGAAGTGAT
TGTTGGGGACTTTGGGATCGTCGTGGTACCCAGGGATGCAGCGGACACAGACCGGATCATGAATCACTCC
TCCATGCTCCGCAAGTACAAAGTGAGTCCTCCA , 7
TCACCAAAGTTGTTTCTCTCGCTTCCTCCTTCCCTCTCCCCCTTCCCCCCTCTCTTTAGATGGAAGTGAT
TGTTGGGGACTTTGGGATCGTCGTGGTACCCAGGGATGCAGCGGACACAGACCGGATCATGAATCACTCC
TCCATACGCCGCAAGTACAAAGTGAGTCCTCCA , 7
TCACCAAAGTTGTTTCTCTCGCTTCCTCCTTCCCTCTCCCCCTTCCCCCCTCTCTTTAGATGGAAGTGAT
TGTTGGGGACTGTGGGATCGTCGTGGTACCCCAAGATGCAGCGGACACAGACCGGATCATGAATCACTCC
TCCATACTCCGCAAGTACAAAGTGAGTCCTCCA , 7
TCACCAAAGTTGTTTCTCTCGCTTCCTCCTTCCCTCTCCCCCTTCCCCCCTCTCTTTAGATGGAAGTGAT
TGTTGGGGACTTTGGGATCGTCGTGGTACCCCAAGATGCAGCGGACACAGACCGGATCATGAATCGCTCC
TCCATACTCCGCAAGTACAAAGTGAGTCCTCCA , 6
TCACCAAAGTTGTTTCTCTCGCTTCCTCCTTCCCTCTCCCCCTTCCCCCCTCTCTTTAGATGGAAGTGAT
TGTTGGGGACTTTGGGATCGTCGTGGTACCCAGGGATGCAGCGGACACAGACCGGATCATGAATCGCTCC
TCCATACTCCGCAAGTACAAAGTGAGTCCTCCA , 5
TCACCAAAGTTGTTTCTCTCGCTTCCTCCTTCCCTCTCCCCCTTCCCCCCTCTCTTTAGATGGAAGTGAT

TGTTGGGGACGTTGGGATCGTCGTGGTACCCAGGGATGCAGCGGACACAGACCGGATCATGAATCACTCC
TCCATACTCCGCAAGTACAAAGTGAGTCCTCCA , 4
TCACCAAAGTTGTTTCTCTCGCTTCCTCCTTCCCTCTCCCCCTTCCCCCCTCTCTTTAGATGGAAGTGAT
TGTTGGGGACTTTGGGATCGTCGTGGTACCCCAAGATGCAGCGGACACAGACCGGATCATGAATCACTCC
TCCATACGCCGCAAGTACAAAGTGAGTCCTCCA , 4

GEIC-Plate01-F06 TOTAL:3400 OrderedDict([('WT_sp1', 1636), ('R232Q', 0), ('Silent Block Mod', 0), ('R232Q (Full ssODN)', 0), ('Silent Block Mod (Full ssODN)', 0)])   [(-1, 1739), (0, 1647), (-2, 14)]
TCACCAAAGTTGTTTCTCTCGCTTCCTCCTTCCCTCTCCCCCTTCCCCCCTCTCTTTAGATGGAAGTGAT
TGTTGGGGACTTTGGGATCGTCGTGGTCCCAGGGATGCAGCGGACACAGACCGGATCATGAATCACTCCT
CCATACTCCGCAAGTACAAAGTGAGTCCTCCA , 1553
TCACCAAAGTTGTTTCTCTCGCTTCCTCCTTCCCTCTCCCCCTTCCCCCCTCTCTTTAGATGGAAGTGAT
TGTTGGGGACTTTGGGATCGTCGTGGTACCCAGGGATGCAGCGGACACAGACCGGATCATGAATCACTCC
TCCATACTCCGCAAGTACAAAGTGAGTCCTCCA , 1450
TCACCAAAGTTGTTTCTCTCGCTTCCTCCTTCCCTCTCCCCCTTCCCCCCTCTCTTTAGATGGAAGTGAT
TGTTGGGGACTGTGGGATCGTCGTGGTACCCAGGGATGCAGCGGACACAGACCGGATCATGAATCACTCC
TCCATACTCCGCAAGTACAAAGTGAGTCCTCCA , 11
TCACCAAAGTTGTTTCTCTCGCTTCCTCCTTCCCTCTCCCCCTTCCCCCCTCTCTTTAGATGGAAGTGAT
TGTTGGGGACTTTGGGATCGTCGTGGTACCCAGGGATGCAGCGGACACAGACCGGATCATGAATCGCTCC
TCCATACTCCGCAAGTACAAAGTGAGTCCTCCA , 10
TCACCAAAGTTGTTTCTCTCGCTTCCTCCTTCCCTCTCCCCTTCCCCCCTCTCTTTAGATGGAAGTGATT
GTTGGGGACTTTGGGATCGTCGTGGTACCCAGGGATGCAGCGGACACAGACCGGATCATGAATCACTCCT
CCATACTCCGCAAGTACAAAGTGAGTCCTCCA , 4
TCACCAAAGTTGTTTCTCTCGCTTCCTCCTTCCCTCTCCCCCTTCCCCCCTCTCTTTAGATGGAAGTGAT
TGTTGGGGACTTTGGGATCGTCGTGGTACCCAGGGATGCAGCGGACACCGACCGGATCATGAATCACTCC
TCCATACTCCGCAAGTACAAAGTGAGTCCTCCA , 4
TCACCAAAGTTGTTTCTCTCGCTTCCTCCTTCCCTCTCCCCCTTCCCCCCTCTCTTTAGATGGAAGTGAT
TGTTGGGGACTTTGGGATCGTCGTGGTACCCAGGGATGCAGCGGACACGGACCGGATCATGAATCACTCC
TCCATACTCCGCAAGTACAAAGTGAGTCCTCCA , 4
TCACCAAAGTTGTTTCTCTCGCTTCCTCCTTCCCTCTCCCCCTTCCCCCCTCTCTTTAGATGGAAGTGAT
TGTTGGGGACTTTGGGATCGTCGTGGTACCCAGGGATGCAGCGGACACAGACCGGATCATGAATCACTCC
TCCATACGCCGCAAGTACAAAGTGAGTCCTCCA , 4
TCACCAAAGTTGTTTCTCTCGCTTCCTCCTTCCCTCTCCCCCTTCCCCCCTCTCTTTAGATGGAAGTGAT
TGTTGGGGACGTTGGGATCGTCGTGGTACCCAGGGATGCAGCGGACACAGACCGGATCATGAATCACTCC
TCCATACTCCGCAAGTACAAAGTGAGTCCTCCA , 4
TCACCAAAGTTGTTTCTCTCGCTTCCTCCTTCCCTCTCCCCCTTCCCCCCTCTCTTTAGATGGAAGTGAT
TGTTGGGGACTTTGGGATCGTCGTGGTCCCAGGGATGCAGCGGACACAGACCGGATCATGAATCGCTCCT
CCATACTCCGCAAGTACAAAGTGAGTCCTCCA , 4
TCACCAAAGTTGTTTCTCTCGCTTCCTCCTTCCCTCTCCCCCTTCCCCCCTCTCTTTAGATGGAAGTGAT
TGTTGGGGACTTTGGGATCGTCGTGGTACCCAGGGATGCAGCGGACACAGGCCGGATCATGAATCACTCC
TCCATACTCCGCAAGTACAAAGTGAGTCCTCCA , 3
TCACCAAAGTTGTTTCTCTCGCTTCCTCCTTCCCTCTCCCCCTTCCCCCCTCTCTTTAGATGGAAGTGAT
TGTTGGGGACTTTGGGATCGTCGTGGTACCCAGGGATGCAGCGGACACGGACCGGATCATGAATCGCTCC
TCCATACTCCGCAAGTACAAAGTGAGTCCTCCA , 3

GEIC-Plate01-F07 TOTAL:3518 OrderedDict([('WT_sp1', 3469), ('R232Q', 0), ('Silent Block Mod', 1), ('R232Q (Full ssODN)', 0), ('Silent Block

Mod (Full ssODN)', 1)])   [(0, 3504), (-1, 13), (1, 1)]
TCACCAAAGTTGTTTCTCTCGCTTCCTCCTTCCCTCTCCCCCTTCCCCCCTCTCTTTAGATGGAAGTGAT
TGTTGGGGACTTTGGGATCGTCGTGGTACCCAGGGATGCAGCGGACACAGACCGGATCATGAATCACTCC
TCCATACTCCGCAAGTACAAAGTGAGTCCTCCA , 3100
TCACCAAAGTTGTTTCTCTCGCTTCCTCCTTCCCTCTCCCCCTTCCCCCCTCTCTTTAGATGGAAGTGAT
TGTTGGGGACTTTGGGATCGTCGTGGTACCCAGGGATGCAGCGGACACGGACCGGATCATGAATCACTCC
TCCATACTCCGCAAGTACAAAGTGAGTCCTCCA , 22
TCACCAAAGTTGTTTCTCTCGCTTCCTCCTTCCCTCTCCCCCTTCCCCCCTCTCTTTAGATGGAAGTGAT
TGTTGGGGACGTTGGGATCGTCGTGGTACCCAGGGATGCAGCGGACACAGACCGGATCATGAATCACTCC
TCCATACTCCGCAAGTACAAAGTGAGTCCTCCA , 15
TCACCAAAGTTGTTTCTCTCGCTTCCTCCTTCCCTCTCCCCCTTCCCCCCTCTCTTTAGATGGAAGTGAT
TGTTGGGGACTGTGGGATCGTCGTGGTACCCAGGGATGCAGCGGACACAGACCGGATCATGAATCACTCC
TCCATACTCCGCAAGTACAAAGTGAGTCCTCCA , 13
TCACCAAAGTTGTTTCTCTCGCTTCCTCCTTCCCTCTCCCCCTTCCCCCCTCTCTTTAGATGGAAGTGAT
TGTTGGGGACTTTGGGATCGTCGTGGTACCCAGGGATGCAGCGGACACAGACCGGATCATGAATCGCTCC
TCCATACTCCGCAAGTACAAAGTGAGTCCTCCA , 11
TCACCAAAGTTGTTTCTCTCGCTTCCTCCTTCCCTCTCCCCCTTCCCCCCTCTCTTTAGATGGAAGTGAT
TGTTGGGGACTTTGGGATCGTCGTGGTACCCAGGGATGCAGCGGACACAGACCGGATCATGAATCACTCC
TCCATGCTCCGCAAGTACAAAGTGAGTCCTCCA , 7
TCACCAAAGTTGTTTCTCTCGCTTCCTCCTTCCCTCTCCCCCTTCCCCCCTCTCTTTAGATGGAAGTGAT
TGTTGGGGACTTTGGGATCGTCGTGGTACCCAGGGATGCAGCGGACACCGACCGGATCATGAATCACTCC
TCCATACTCCGCAAGTACAAAGTGAGTCCTCCA , 6
TCACCAAAGTTGTTTCTCTCGCTTCCTCCTTCCCTCTCCCCCTTCCCCCCTCTCTTTAGATGGAAGTGAT
TGTTGGGGACTTTGGGATCGTCGTGGTACCCAGGGATGCAGCGGACACAGACCGGATCATGAATCACTCC
TCCATACGCCGCAAGTACAAAGTGAGTCCTCCA , 6
TCACCAAAGTTGTTTCTCTCGCTTCCTCCTTCCCTCTCCCCCTTCCCCCCTCTCTTTAGATGGAAGTGAT
TGTTGGGGACTTTGGGATCGTCGTGGTACCCAGGGATGCAGCGGACACAGACCGGATCATGAATCACTCC
TCCATACTCCGCAAGTACCAAGTGAGTCCTCCA , 5
TCACCAAAGTTGTTTCTCTCGCTTCCTCCTTCCCTCTCCCCCTTCCCCCCTCTCTTTAGATGGAAGTGAT
TGTTGGGGACTTTGGGATCGTCGTGGTACCCAGGGATGCAGCGGACACAGGCCGGATCATGAATCACTCC
TCCATACTCCGCAAGTACAAAGTGAGTCCTCCA , 4
TCACCAAAGTTGTTTCTCTCGCTTCCTCCTTCCCTCTCCCCCTTCCCCCCTCTCTTTAGATGGAAGTGAA
TGTTGGGGACTTTGGGATCGTCGTGGTACCCAGGGATGCAGCGGACACAGACCGGATCATGAATCACTCC
TCCATACTCCGCAAGTACAAAGTGAGTCCTCCA , 4
TCACCAAAGTTGTTTCTCTCGCTTCCTCCTTCCCTCTCCCCCTTCCCCCCTCTCTTTAGATGGAAGTGAT
TGTTGGGGACTTTGGGATCGTCGTGGTACCCAGGGATGCAGCGGGCACAGACCGGATCATGAATCACTCC
TCCATACTCCGCAAGTACAAAGTGAGTCCTCCA , 4

GEIC-Plate01-F08 TOTAL:2893 OrderedDict([('WT_sp1', 2838), ('R232Q', 0), ('Silent Block Mod', 0), ('R232Q (Full ssODN)', 0), ('Silent Block Mod (Full ssODN)', 0)])   [(0, 2880), (-1, 13)]
TCACCAAAGTTGTTTCTCTCGCTTCCTCCTTCCCTCTCCCCCTTCCCCCCTCTCTTTAGATGGAAGTGAT
TGTTGGGGACTTTGGGATCGTCGTGGTACCCAGGGATGCAGCGGACACAGACCGGATCATGAATCACTCC
TCCATACTCCGCAAGTACAAAGTGAGTCCTCCA , 2525
TCACCAAAGTTGTTTCTCTCGCTTCCTCCTTCCCTCTCCCCCTTCCCCCCTCTCTTTAGATGGAAGTGAT
TGTTGGGGACGTTGGGATCGTCGTGGTACCCAGGGATGCAGCGGACACAGACCGGATCATGAATCACTCC
TCCATACTCCGCAAGTACAAAGTGAGTCCTCCA , 13
TCACCAAAGTTGTTTCTCTCGCTTCCTCCTTCCCTCTCCCCCTTCCCCCCTCTCTTTAGATGGAAGTGAT
TGTTGGGGACTTTGGGATCGTCGTGGTACCCAGGGATGCAGCGGACACGGACCGGATCATGAATCACTCC

TCCATACTCCGCAAGTACAAAGTGAGTCCTCCA , 11
TCACCAAAGTTGTTTCTCTCGCTTCCTCCTTCCCTCTCCCCCTTCCCCCCTCTCTTTAGATGGAAGTGAT
TGTTGGGGACTGTGGGATCGTCGTGGTACCCAGGGATGCAGCGGACACAGACCGGATCATGAATCACTCC
TCCATACTCCGCAAGTACAAAGTGAGTCCTCCA , 10
TCACCAAAGTTGTTTCTCTCGCTTCCTCCTTCCCTCTCCCCCTTCCCCCCTCTCTTTAGATGGAAGTGAT
TGTTGGGGACTTTGGGATCGTCGTGGTACCCAGGGATGCAGCGGACACAGACCGGATCATGAATCGCTCC
TCCATACTCCGCAAGTACAAAGTGAGTCCTCCA , 8
TCACCAAAGTTGTTTCTCTCGCTTCCTCCTTCCCTCTCCCCCTTCCCCCCTCTCTTTAGATGGAAGTGAT
TGTTGGGGACTTTGGGATCGTCGTGGTACCCAGGGATGCAGCGGACACAGACCGGATCATGAATCACTCC
TCCATGCTCCGCAAGTACAAAGTGAGTCCTCCA , 5
TCACCAAAGTTGTTTCTCTCGCTTCCTCCTTCCCTCTCCCCCTTCCCCCCTCTCTTTAGATGGAAGTGAT
TGTTGGGGACTTTGGGATCGTCGTGGTACCCAGGGATGCAGCGGACACAGGCCGGATCATGAATCACTCC
TCCATACTCCGCAAGTACAAAGTGAGTCCTCCA , 4
TCACCAAAGTTGTTTCTCTCGCTTCCTCCTTCCCTCTCCCCCTTCCCCCCTCTCTTTAGATGGAAGTGAT
TGTTGGGGACTTTGGGATCGTCGTGGTACCCAGGGATGCAGCGGACACGGACCGGATCATGAATCGCTCC
TCCATACTCCGCAAGTACAAAGTGAGTCCTCCA , 4
TCACCAAAGTTGTTTCTCTCGCTTCCTCCTTCCCTCTCCCCCTTCCCCCCTCTCTTTAGATGGAAGTGAT
TGTTGGGGACTTTGGGATCGTCGTGGTACCCAGGGATGCAGCAGACACAGACCGGATCATGAATCACTCC
TCCATACTCCGCAAGTACAAAGTGAGTCCTCCA , 4
TCACCAAAGTTGTTTCTCTCGCTTCCTCCTTCCCCCTCCCCCTTCCCCCCTCTCTTTAGATGGAAGTGAT
TGTTGGGGACTTTGGGATCGTCGTGGTACCCAGGGATGCAGCGGACACAGACCGGATCATGAATCACTCC
TCCATACTCCGCAAGTACAAAGTGAGTCCTCCA , 4
TCACCAAAGTTGTTTCTCTCGCTTCCTCCTTCCCTCTCCCCCTTCCCCCCTCTCTTTAGATGGAAGTGAT
TGTTGGGGACTTTGGGATCGTCGTGGTACCCAGGGATGCAGCGGACACAGACCGGATCATGAATCACTCC
TCCATACTCCGCGAGTACAAAGTGAGTCCTCCA , 4
TCACCAAAGTTGTTTCTCTCGCTTCCTCCTTCCCTCTCCCCTTCCCCCCTCTCTTTAGATGGAAGTGATT
GTTGGGGACTTTGGGATCGTCGTGGTACCCAGGGATGCAGCGGACACAGACCGGATCATGAATCACTCCT
CCATACTCCGCAAGTACAAAGTGAGTCCTCCA , 3

GEIC-Plate01-G01 TOTAL:3656 OrderedDict([('WT_sp1', 1821), ('R232Q', 1742), ('Silent Block Mod', 0), ('R232Q (Full ssODN)', 1585), ('Silent Block Mod (Full ssODN)', 0)]) [(0, 3642), (-1, 13), (-3, 1)]
TCACCAAAGTTGTTTCTCTCGCTTCCTCCTTCCCTCTCCCCCTTCCCCCCTCTCTTTAGATGGAAGTGAT
TGTTGGGGACTTTGGGATCGTCGTGGTACCCAGGGATGCAGCGGACACAGACCGGATCATGAATCACTCC
TCCATACTCCGCAAGTACAAAGTGAGTCCTCCA , 1645
TCACCAAAGTTGTTTCTCTCGCTTCCTCCTTCCCTCTCCCCCTTCCCCCCTCTCTTTAGATGGAAGTGAT
TGTTGGGGACTTTGGGATCGTCGTGGTACCCCAAGATGCAGCGGACACAGACCGGATCATGAATCACTCC
TCCATACTCCGCAAGTACAAAGTGAGTCCTCCA , 1572
TCACCAAAGTTGTTTCTCTCGCTTCCTCCTTCCCTCTCCCCCTTCCCCCCTCTCTTTAGATGGAAGTGAT
TGTTGGGGACTTTGGGATCGTCGTGGTACCCAGGGATGCAGCGGACACAGACCGGATCATGAATCGCTCC
TCCATACTCCGCAAGTACAAAGTGAGTCCTCCA , 9
TCACCAAAGTTGTTTCTCTCGCTTCCTCCTTCCCTCTCCCCCTTCCCCCCTCTCTTTAGATGGAAGTGAT
TGTTGGGGACTTTGGGATCGTCGTGGTACCCAGGGATGCAGCGGACACGGACCGGATCATGAATCACTCC
TCCATACTCCGCAAGTACAAAGTGAGTCCTCCA , 9
TCACCAAAGTTGTTTCTCTCGCTTCCTCCTTCCCTCTCCCCCTTCCCCCCTCTCTTTAGATGGAAGTGAT
TGTTGGGGACTTTGGGATCGTCGTGGTACCCCAAGATGCAGCGGACACAGACCGGATCATGAATCACTCC
TCCATACGCCGCAAGTACAAAGTGAGTCCTCCA , 9
TCACCAAAGTTGTTTCTCTCGCTTCCTCCTTCCCTCTCCCCCTTCCCCCCTCTCTTTAGATGGAAGTGAT
TGTTGGGGACTGTGGGATCGTCGTGGTACCCAGGGATGCAGCGGACACAGACCGGATCATGAATCACTCC

TCCATACTCCGCAAGTACAAAGTGAGTCCTCCA , 7
TCACCAAAGTTGTTTCTCTCGCTTCCTCCTTCCCTCTCCCCCTTCCCCCCTCTCTTTAGATGGAAGTGAT
TGTTGGGGACGTTGGGATCGTCGTGGTACCCCAAGATGCAGCGGACACAGACCGGATCATGAATCACTCC
TCCATACTCCGCAAGTACAAAGTGAGTCCTCCA , 6
TCACCAAAGTTGTTTCTCTCGCTTCCTCCTTCCCTCTCCCCCTTCCCCCCTCTCTTTAGATGGAAGTGAT
TGTTGGGGACGTTGGGATCGTCGTGGTACCCAGGGATGCAGCGGACACAGACCGGATCATGAATCACTCC
TCCATACTCCGCAAGTACAAAGTGAGTCCTCCA , 5
TCACCAAAGTTGTTTCTCTCGCTTCCTCCTTCCCTCTCCCCCTTCCCCCCTCTCTTTAGATGGAAGTGAT
TGTTGGGGACTTTGGGATCGTCGTGGTACCCCAAGATGCAGCGGACACAGACCGGATCATGAATCACTCC
TCCATGCTCCGCAAGTACAAAGTGAGTCCTCCA , 5
TCACCAAAGTTGTTTCTCTCGCTTCCTCCTTCCCTCTCCCCCTTCCCCCCTCTCTTTAGATGGAAGTGAT
TGTTGGGGACTTTGGGATCGTCGTGGTACCCAGGGATGCAGCGGACACAGGCCGGATCATGAATCACTCC
TCCATACTCCGCAAGTACAAAGTGAGTCCTCCA , 4
TCACCAAAGTTGTTTCTCTCGCTTCCTCCTTCCCTCTCCCCCTTCCCCCCTCTCTTTAGATGGAAGTGAT
TGTTGGGGACTTTGGGATCGTCGTGGTACCCAGGGATGCAGCGGACACAGACCGGATCATGAATCACTCC
TCCATGCTCCGCAAGTACAAAGTGAGTCCTCCA , 4
TCACCAAAGTTGTTTCTCTCGCTTCCTCCTTCCCTCTCCCCCTTCCCCCCTCTCTTTAGATGGAAGTGAT
TGTTGGGGACTTTGGGATCGTCGTGGTACCCAGGGATGCAGCGGACACAGACCAGATCATGAATCACTCC
TCCATACTCCGCAAGTACAAAGTGAGTCCTCCA , 4

GEIC-Plate01-G02 TOTAL:3039 OrderedDict([('WT_sp1', 1483), ('R232Q', 1486), ('Silent Block Mod', 0), ('R232Q (Full ssODN)', 1345), ('Silent Block Mod (Full ssODN)', 0)])   [(0, 3030), (-1, 8), (-2, 1)]
TCACCAAAGTTGTTTCTCTCGCTTCCTCCTTCCCTCTCCCCCTTCCCCCCTCTCTTTAGATGGAAGTGAT
TGTTGGGGACTTTGGGATCGTCGTGGTACCCAGGGATGCAGCGGACACAGACCGGATCATGAATCACTCC
TCCATACTCCGCAAGTACAAAGTGAGTCCTCCA , 1333
TCACCAAAGTTGTTTCTCTCGCTTCCTCCTTCCCTCTCCCCCTTCCCCCCTCTCTTTAGATGGAAGTGAT
TGTTGGGGACTTTGGGATCGTCGTGGTACCCCAAGATGCAGCGGACACAGACCGGATCATGAATCACTCC
TCCATACTCCGCAAGTACAAAGTGAGTCCTCCA , 1327
TCACCAAAGTTGTTTCTCTCGCTTCCTCCTTCCCTCTCCCCCTTCCCCCCTCTCTTTAGATGGAAGTGAT
TGTTGGGGACTTTGGGATCGTCGTGGTACCCAGGGATGCAGCGGACACGGACCGGATCATGAATCACTCC
TCCATACTCCGCAAGTACAAAGTGAGTCCTCCA , 12
TCACCAAAGTTGTTTCTCTCGCTTCCTCCTTCCCTCTCCCCCTTCCCCCCTCTCTTTAGATGGAAGTGAT
TGTTGGGGACTGTGGGATCGTCGTGGTACCCCAAGATGCAGCGGACACAGACCGGATCATGAATCACTCC
TCCATACTCCGCAAGTACAAAGTGAGTCCTCCA , 11
TCACCAAAGTTGTTTCTCTCGCTTCCTCCTTCCCTCTCCCCCTTCCCCCCTCTCTTTAGATGGAAGTGAT
TGTTGGGGACGTTGGGATCGTCGTGGTACCCCAAGATGCAGCGGACACAGACCGGATCATGAATCACTCC
TCCATACTCCGCAAGTACAAAGTGAGTCCTCCA , 9
TCACCAAAGTTGTTTCTCTCGCTTCCTCCTTCCCTCTCCCCCTTCCCCCCTCTCTTTAGATGGAAGTGAT
TGTTGGGGACTTTGGGATCGTCGTGGTACCCCAAGATGCAGCGGACACAGACCGGATCATGAATCACTCC
TCCATACGCCGCAAGTACAAAGTGAGTCCTCCA , 7
TCACCAAAGTTGTTTCTCTCGCTTCCTCCTTCCCTCTCCCCCTTCCCCCCTCTCTTTAGATGGAAGTGAT
TGTTGGGGACTGTGGGATCGTCGTGGTACCCAGGGATGCAGCGGACACAGACCGGATCATGAATCACTCC
TCCATACTCCGCAAGTACAAAGTGAGTCCTCCA , 7
TCACCAAAGTTGTTTCTCTCGCTTCCTCCTTCCCTCTCCCCCTTCCCCCCTCTCTTTAGATGGAAGTGAT
TGTTGGGGACGTTGGGATCGTCGTGGTACCCAGGGATGCAGCGGACACAGACCGGATCATGAATCACTCC
TCCATACTCCGCAAGTACAAAGTGAGTCCTCCA , 6
TCACCAAAGTTGTTTCTCTCGCTTCCTCCTTCCCTCTCCCCCTTCCCCCCTCTCTTTAGATGGAAGTGAT
TGTTGGGGACTTTGGGATCGTCGTGGTACCCCAAGATGCAGCGGACACAGACCGGATCATGAATCGCTCC

TCCATACTCCGCAAGTACAAAGTGAGTCCTCCA , 5
TCACCAAAGTTGTTTCTCTCGCTTCCTCCTTCCCTCTCCCCCTTCCCCCCTCTCTTTAGATGGAAGTGAT
TGTTGGGGACTTTGGGATCGTCGTGGTACCCCAAGATGCAGCGGACACAGACCGGATCATGAATCACTCC
GCCATACTCCGCAAGTACAAAGTGAGTCCTCCA , 4
TCACCAAAGTTGTTTCTCTCGCTTCCTCCTTCCCTCTCCCCCTTCCCCCCTCTCTTTAGATGGAAGTGAT
TGTTGGGGACTTTGGGATCGTCGTGGTACCCAGGGATGCAGCGGACACAGGCCGGATCATGAATCACTCC
TCCATACTCCGCAAGTACAAAGTGAGTCCTCCA , 3
TCACCAAAGTTGTTTCTCTCGCTTCCTCCTTCCCTCTCCCCCTTCCCCCCTCTCTTTAGATGGAAGTGAT
TGTTGGGGACTTTGGGATCGTCGTGGTACCCAGGGATGCAGCGGACACAGACCGGATCATGAATCGCTCC
TCCATACTCCGCAAGTACAAAGTGAGTCCTCCA , 3

GEIC-Plate01-G03 TOTAL:3089 OrderedDict([('WT_sp1', 3030), ('R232Q', 0), ('Silent Block Mod', 0), ('R232Q (Full ssODN)', 0), ('Silent Block Mod (Full ssODN)', 0)])   [(0, 3075), (-1, 13), (1, 1)]
TCACCAAAGTTGTTTCTCTCGCTTCCTCCTTCCCTCTCCCCCTTCCCCCCTCTCTTTAGATGGAAGTGAT
TGTTGGGGACTTTGGGATCGTCGTGGTACCCAGGGATGCAGCGGACACAGACCGGATCATGAATCACTCC
TCCATACTCCGCAAGTACAAAGTGAGTCCTCCA , 2610
TCACCAAAGTTGTTTCTCTCGCTTCCTCCTTCCCTCTCCCCCTTCCCCCCTCTCTTTAGATGGAAGTGAT
TGTTGGGGACTTTGGGATCGTCGTGGTACCCAGGGATGCAGCGGACACGGACCGGATCATGAATCACTCC
TCCATACTCCGCAAGTACAAAGTGAGTCCTCCA , 25
TCACCAAAGTTGTTTCTCTCGCTTCCTCCTTCCCTCTCCCCCTTCCCCCCTCTCTTTAGATGGAAGTGAT
TGTTGGGGACGTTGGGATCGTCGTGGTACCCAGGGATGCAGCGGACACAGACCGGATCATGAATCACTCC
TCCATACTCCGCAAGTACAAAGTGAGTCCTCCA , 12
TCACCAAAGTTGTTTCTCTCGCTTCCTCCTTCCCTCTCCCCCTTCCCCCCTCTCTTTAGATGGAAGTGAT
TGTTGGGGACTGTGGGATCGTCGTGGTACCCAGGGATGCAGCGGACACAGACCGGATCATGAATCACTCC
TCCATACTCCGCAAGTACAAAGTGAGTCCTCCA , 12
TCACCAAAGTTGTTTCTCTCGCTTCCTCCTTCCCTCTCCCCCTTCCCCCCTCTCTTTAGATGGAAGTGAT
TGTTGGGGACTTTGGGATCGTCGTGGTACCCAGGGATGCAGCGGACACAGACCGGATCATGAATCACTCC
GCCATACTCCGCAAGTACAAAGTGAGTCCTCCA , 12
TCACCAAAGTTGTTTCTCTCGCTTCCTCCTTCCCTCTCCCCCTTCCCCCCTCTCTTTAGATGGAAGTGAT
TGTTGGGGACTTTGGGATCGTCGTGGTACCCAGGGATGCAGCGGACACAGACCGGATCATGAATCACTCC
TCCATACGCCGCAAGTACAAAGTGAGTCCTCCA , 9
TCACCAAAGTTGTTTCTCTCGCTTCCTCCTTCCCTCTCCCCCTTCCCCCCTCTCTTTAGATGGAAGTGAT
TGTTGGGGACTTTGGGATCGTCGTGGTACCCAGGGATGCAGCGGACACAGACCGGATCATGAATCGCTCC
TCCATACTCCGCAAGTACAAAGTGAGTCCTCCA , 9
TCACCAAAGTTGTTTCTCTCGCTTCCTCCTTCCCTCTCCCCCTTCCCCCCTCTCTTTAGATGGAAGTGAT
TGTTGGGGACTTTGGGATCGTCGTGGTACCCAGGGATGCAGCGGACACCGACCGGATCATGAATCACTCC
TCCATACTCCGCAAGTACAAAGTGAGTCCTCCA , 8
TCACCAAAGTTGTTTCTCTCGCTTCCTCCTTCCCTCTCCCCCTTCCCCCTCTCTTTAGATGGAAGTGATT
GTTGGGGACTTTGGGATCGTCGTGGTACCCAGGGATGCAGCGGACACAGACCGGATCATGAATCACTCCT
CCATACTCCGCAAGTACAAAGTGAGTCCTCCA , 8
TCACCAAAGTTGTTTCTCTCGCTTCCTCCTTCCCTCTCCCCCTTCCCCCCTCTCTTTAGATGGAAGTGAG
TGTTGGGGACTTTGGGATCGTCGTGGTACCCAGGGATGCAGCGGACACAGACCGGATCATGAATCACTCC
TCCATACTCCGCAAGTACAAAGTGAGTCCTCCA , 6
TCACCAAAGTTGTTTCTCTCGCTTCCTCCTTCCCTCTCCCCCTTCCCCCCTCTCTTTAGATGGAAGTGAT
TGTTGGGGACTTTGGGGTCGTCGTGGTACCCAGGGATGCAGCGGACACAGACCGGATCATGAATCACTCC
TCCATACTCCGCAAGTACAAAGTGAGTCCTCCA , 5
TCACCAAAGTTGTTTCTCTCGCTTCCTCCTTCCCTCTCCCCCTTCCCCCCTCTCTTTAGATGGAAGTGAT
TGTTGGGGACTTTGTGATCGTCGTGGTACCCAGGGATGCAGCGGACACAGACCGGATCATGAATCACTCC

TCCATACTCCGCAAGTACAAAGTGAGTCCTCCA , 5

GEIC-Plate01-G04 TOTAL:3957 OrderedDict([('WT_sp1', 3900), ('R232Q', 0), ('Silent Block Mod', 1), ('R232Q (Full ssODN)', 0), ('Silent Block Mod (Full ssODN)', 1)])   [(0, 3941), (-1, 15), (-2, 1)]
TCACCAAAGTTGTTTCTCTCGCTTCCTCCTTCCCTCTCCCCCTTCCCCCCTCTCTTTAGATGGAAGTGAT
TGTTGGGGACTTTGGGATCGTCGTGGTACCCAGGGATGCAGCGGACACAGACCGGATCATGAATCACTCC
TCCATACTCCGCAAGTACAAAGTGAGTCCTCCA , 3450
TCACCAAAGTTGTTTCTCTCGCTTCCTCCTTCCCTCTCCCCCTTCCCCCCTCTCTTTAGATGGAAGTGAT
TGTTGGGGACTTTGGGATCGTCGTGGTACCCAGGGATGCAGCGGACACGGACCGGATCATGAATCACTCC
TCCATACTCCGCAAGTACAAAGTGAGTCCTCCA , 21
TCACCAAAGTTGTTTCTCTCGCTTCCTCCTTCCCTCTCCCCCTTCCCCCCTCTCTTTAGATGGAAGTGAT
TGTTGGGGACTGTGGGATCGTCGTGGTACCCAGGGATGCAGCGGACACAGACCGGATCATGAATCACTCC
TCCATACTCCGCAAGTACAAAGTGAGTCCTCCA , 18
TCACCAAAGTTGTTTCTCTCGCTTCCTCCTTCCCTCTCCCCCTTCCCCCCTCTCTTTAGATGGAAGTGAT
TGTTGGGGACTTTGGGATCGTCGTGGTACCCAGGGATGCAGCGGACACAGACCGGATCATGAATCGCTCC
TCCATACTCCGCAAGTACAAAGTGAGTCCTCCA , 17
TCACCAAAGTTGTTTCTCTCGCTTCCTCCTTCCCTCTCCCCCTTCCCCCCTCTCTTTAGATGGAAGTGAT
TGTTGGGGACTTTGGGATCGTCGTGGTACCCAGGGATGCAGCGGACACCGACCGGATCATGAATCACTCC
TCCATACTCCGCAAGTACAAAGTGAGTCCTCCA , 11
TCACCAAAGTTGTTTCTCTCGCTTCCTCCTTCCCTCTCCCCCTTCCCCCCTCTCTTTAGATGGAAGTGAT
TGTTGGGGACGTTGGGATCGTCGTGGTACCCAGGGATGCAGCGGACACAGACCGGATCATGAATCACTCC
TCCATACTCCGCAAGTACAAAGTGAGTCCTCCA , 10
TCACCAAAGTTGTTTCTCTCGCTTCCTCCTTCCCTCTCCCCCTTCCCCCCTCTCTTTAGATGGAAGTGAT
TGTTGGGGACTTTGGGATCGTCGTGGTACCCAGGGATGCAGCGGACACAGACCGGATCATGAATCACTCC
TCCATACGCCGCAAGTACAAAGTGAGTCCTCCA , 8
TCACCAAAGTTGTTTCTCTCGCTTCCTCCTTCCCTCTCCCCCTTCCCCCCTCTCTTTAGATGGAAGTGAG
TGTTGGGGACTTTGGGATCGTCGTGGTACCCAGGGATGCAGCGGACACAGACCGGATCATGAATCACTCC
TCCATACTCCGCAAGTACAAAGTGAGTCCTCCA , 7
TCACCAAAGTTGTTTCTCTCGCTTCCTCCTTCCCTCTCCCCCTTCCCCCCTCTCTTTAGATGGAAGTGAT
TGTTGGGGACTTTGGGATCGTCGTGGTACCCAGGGATGCAGCGGACACTGACCGGATCATGAATCACTCC
TCCATACTCCGCAAGTACAAAGTGAGTCCTCCA , 5
TCACCAAAGTTGTTTCTCTCGCTTCCTCCTTCCCTCTCCCCCTTCCCCCCTCTCTTTAGATGGAAGTGAT
TGTTGGGGACTTTGGGATCGTCGTGGTACCCAGGGATGCAGCGGACACAGACCGGATCATGAATCACTCC
TCCATGCTCCGCAAGTACAAAGTGAGTCCTCCA , 5
TCACCAAAGTTGTTTCTCTCGCTTCCTCCTTCCCTCTCCCCCTTCCCCCCTCTCTTTAGATGGAAGTGAT
TGTTGGGGACTTTGGGATCGTCGTGGTACCCAGGGATGCAGCGGACACAGACCGGATCATGAATCACTCC
TCCATACTCCGCAAGTACCAAGTGAGTCCTCCA , 5
TCACCAAAGTTGTTTCTCTCGCTTCCTCCTTCCCTCTCCCCCTTCCCCCCTCTCTTTAGATGGAAGTGAT
TGTTGGGGACTTTGGGATCGTCGTGGTACCCAGGGATGCAGCGGACACAGACCGGATCATGAATCACTCC
GCCATACTCCGCAAGTACAAAGTGAGTCCTCCA , 5

GEIC-Plate01-G05 TOTAL:3305 OrderedDict([('WT_sp1', 1578), ('R232Q', 0), ('Silent Block Mod', 1621), ('R232Q (Full ssODN)', 0), ('Silent Block Mod (Full ssODN)', 1422)])   [(0, 3295), (-1, 8), (1, 1), (-29, 1)]
TCACCAAAGTTGTTTCTCTCGCTTCCTCCTTCCCTCTCCCCCTTCCCCCCTCTCTTTAGATGGAAGTGAT
TGTTGGGGACTTTGGGATCGTCGTGGTACCCCGTGATGCAGCGGACACAGACCGGATCATGAATCACTCC

TCCATACTCCGCAAGTACAAAGTGAGTCCTCCA , 1409
TCACCAAAGTTGTTTCTCTCGCTTCCTCCTTCCCTCTCCCCCTTCCCCCCTCTCTTTAGATGGAAGTGAT
TGTTGGGGACTTTGGGATCGTCGTGGTACCCAGGGATGCAGCGGACACAGACCGGATCATGAATCACTCC
TCCATACTCCGCAAGTACAAAGTGAGTCCTCCA , 1351
TCACCAAAGTTGTTTCTCTCGCTTCCTCCTTCCCTCTCCCCCTTCCCCCCTCTCTTTAGATGGAAGTGAT
TGTTGGGGACGTTGGGATCGTCGTGGTACCCCGTGATGCAGCGGACACAGACCGGATCATGAATCACTCC
TCCATACTCCGCAAGTACAAAGTGAGTCCTCCA , 12
TCACCAAAGTTGTTTCTCTCGCTTCCTCCTTCCCTCTCCCCCTTCCCCCCTCTCTTTAGATGGAAGTGAT
TGTTGGGGACTTTGGGATCGTCGTGGTACCCAGGGATGCAGCGGACACGGACCGGATCATGAATCACTCC
TCCATACTCCGCAAGTACAAAGTGAGTCCTCCA , 12
TCACCAAAGTTGTTTCTCTCGCTTCCTCCTTCCCTCTCCCCCTTCCCCCCTCTCTTTAGATGGAAGTGAT
TGTTGGGGACTTTGGGATCGTCGTGGTACCCCGTGATGCAGCGGACACAGACCGGATCATGAATCGCTCC
TCCATACTCCGCAAGTACAAAGTGAGTCCTCCA , 11
TCACCAAAGTTGTTTCTCTCGCTTCCTCCTTCCCTCTCCCCCTTCCCCCCTCTCTTTAGATGGAAGTGAT
TGTTGGGGACTGTGGGATCGTCGTGGTACCCCGTGATGCAGCGGACACAGACCGGATCATGAATCACTCC
TCCATACTCCGCAAGTACAAAGTGAGTCCTCCA , 10
TCACCAAAGTTGTTTCTCTCGCTTCCTCCTTCCCTCTCCCCCTTCCCCCCTCTCTTTAGATGGAAGTGAT
TGTTGGGGACGTTGGGATCGTCGTGGTACCCAGGGATGCAGCGGACACAGACCGGATCATGAATCACTCC
TCCATACTCCGCAAGTACAAAGTGAGTCCTCCA , 9
TCACCAAAGTTGTTTCTCTCGCTTCCTCCTTCCCTCTCCCCCTTCCCCCCTCTCTTTAGATGGAAGTGAT
TGTTGGGGACTGTGGGATCGTCGTGGTACCCAGGGATGCAGCGGACACAGACCGGATCATGAATCACTCC
TCCATACTCCGCAAGTACAAAGTGAGTCCTCCA , 6
TCACCAAAGTTGTTTCTCTCGCTTCCTCCTTCCCTCTCCCCCTTCCCCCCTCTCTTTAGATGGAAGTGAT
TGTTGGGGACTTTGGGATCGTCGTGGTACCCAGGGATGCAGCGGACACAGACCGGATCATGAATCACTCC
TCCATGCTCCGCAAGTACAAAGTGAGTCCTCCA , 5
TCACCAAAGTTGTTTCTCTCGCTTCCTCCTTCCCTCTCCCCCTTCCCCCCTCTCTTTAGATGGAAGTGAT
TGTTGGGGACTTTGGGATCGTCGTGGTACCCCGTGATGCAGCGGACACAGACCGGATCATGAATCACTCC
TCCATGCTCCGCAAGTACAAAGTGAGTCCTCCA , 5
TCACCAAAGTTGTTTCTCTCGCTTCCTCCTTCCCTCTCCCCCTTCCCCCCTCTCTTTAGATGGAAGTGAT
TGTTGGGGACTTTGGGATCGTCGTGGTACCCCGTGATGCAGCGGACACAGACCGGATCATGAATCACTCC
GCCATACTCCGCAAGTACAAAGTGAGTCCTCCA , 4
TCACCAAAGTTGTTTCTCTCGCTTCCTCCTTCCCTCTCCCCCTTCCCCCCTCTCTTTAGATGGAAGTGAT
TGTTGGGGACTTTGGGATCGTCGTGGTACCCAGGGATGCAGCGGACACAGACCGGATCATGAATCGCTCC
TCCATACTCCGCAAGTACAAAGTGAGTCCTCCA , 4

GEIC-Plate01-G06 TOTAL:3155 OrderedDict([('WT_sp1', 1613), ('R232Q', 0), ('Silent Block Mod', 1492), ('R232Q (Full ssODN)', 0), ('Silent Block Mod (Full ssODN)', 1368)])   [(0, 3144), (-1, 11)]
TCACCAAAGTTGTTTCTCTCGCTTCCTCCTTCCCTCTCCCCCTTCCCCCCTCTCTTTAGATGGAAGTGAT
TGTTGGGGACTTTGGGATCGTCGTGGTACCCAGGGATGCAGCGGACACAGACCGGATCATGAATCACTCC
TCCATACTCCGCAAGTACAAAGTGAGTCCTCCA , 1462
TCACCAAAGTTGTTTCTCTCGCTTCCTCCTTCCCTCTCCCCCTTCCCCCCTCTCTTTAGATGGAAGTGAT
TGTTGGGGACTTTGGGATCGTCGTGGTACCCCGTGATGCAGCGGACACAGACCGGATCATGAATCACTCC
TCCATACTCCGCAAGTACAAAGTGAGTCCTCCA , 1353
TCACCAAAGTTGTTTCTCTCGCTTCCTCCTTCCCTCTCCCCCTTCCCCCCTCTCTTTAGATGGAAGTGAT
TGTTGGGGACGTTGGGATCGTCGTGGTACCCCGTGATGCAGCGGACACAGACCGGATCATGAATCACTCC
TCCATACTCCGCAAGTACAAAGTGAGTCCTCCA , 10
TCACCAAAGTTGTTTCTCTCGCTTCCTCCTTCCCTCTCCCCCTTCCCCCCTCTCTTTAGATGGAAGTGAT
TGTTGGGGACTTTGGGATCGTCGTGGTACCCAGGGATGCAGCGGACACGGACCGGATCATGAATCACTCC

TCCATACTCCGCAAGTACAAAGTGAGTCCTCCA , 8
TCACCAAAGTTGTTTCTCTCGCTTCCTCCTTCCCTCTCCCCCTTCCCCCCTCTCTTTAGATGGAAGTGAT
TGTTGGGGACGTTGGGATCGTCGTGGTACCCAGGGATGCAGCGGACACAGACCGGATCATGAATCACTCC
TCCATACTCCGCAAGTACAAAGTGAGTCCTCCA , 6
TCACCAAAGTTGTTTCTCTCGCTTCCTCCTTCCCTCTCCCCCTTCCCCCCTCTCTTTAGATGGAAGTGAT
TGTTGGGGACTGTGGGATCGTCGTGGTACCCAGGGATGCAGCGGACACAGACCGGATCATGAATCACTCC
TCCATACTCCGCAAGTACAAAGTGAGTCCTCCA , 5
TCACCAAAGTTGTTTCTCTCGCTTCCTCCTTCCCTCTCCCCCTTCCCCCCTCTCTTTAGATGGAAGTGAT
TGTTGGGGACTTTGGGATCGTCGTGGTACCCAGGGATGCAGCGGACACAGACCGGATCATGAATCACTCC
TACATACTCCGCAAGTACAAAGTGAGTCCTCCA , 4
TCACCAAAGTTGTTTCTCTCGCTTCCTCCTTCCCTCTCCCCCTTCCCCCCTCTCTTTAGATGGAAGTGAT
TGTTGGGGACTTTGGGATCGTCGTGGTACCCCGTGATGCAGCGGACACAGACCGGATCATGAATCGCTCC
TCCATACTCCGCAAGTACAAAGTGAGTCCTCCA , 4
TCACCAAAGTTGTTTCTCTCGCTTCCTCCTTCCCTCTCCCCCTTCCCCCCTCTCTTTAGATGGAAGTGAT
TGTTGGGGACTTTGGGATCGTCGTGGTACCCCGTGATGCAGCGGACACAGGCCGGATCATGAATCACTCC
TCCATACTCCGCAAGTACAAAGTGAGTCCTCCA , 4
TCACCAAAGTTGTTTCTCTCGCTTCCTCCTTCCCTCTCCCCCTTCCCCCCTCTCTTTAGATGGAAGTGAT
TGTTGGGGACTGTGGGATCGTCGTGGTACCCCGTGATGCAGCGGACACAGACCGGATCATGAATCACTCC
TCCATACTCCGCAAGTACAAAGTGAGTCCTCCA , 3
TCACCAAAGTTGTTTCTCTCGCTTCCTCCTTCCCTCTCCCCCTTCCCCCCTCTCTTTAGATGGAAGTGAT
TGTTGGGGACTATGGGATCGTCGTGGTACCCAGGGATGCAGCGGACACAGACCGGATCATGAATCACTCC
TCCATACTCCGCAAGTACAAAGTGAGTCCTCCA , 3
TCACCAAAGTTGTTTCTCTCGCTTCCTCCTTCCCTCTCCCCCTTCCCCCCTCTCTTTAGATGGAAGTGAT
TGTTGGGGACTTTGGGATCTTCGTGGTACCCCGTGATGCAGCGGACACAGACCGGATCATGAATCACTCC
TCCATACTCCGCAAGTACAAAGTGAGTCCTCCA , 3

GEIC-Plate01-G07 TOTAL:3109 OrderedDict([('WT_sp1', 3049), ('R232Q', 0), ('Silent Block Mod', 0), ('R232Q (Full ssODN)', 0), ('Silent Block Mod (Full ssODN)', 0)])   [(0, 3096), (-1, 12), (1, 1)]
TCACCAAAGTTGTTTCTCTCGCTTCCTCCTTCCCTCTCCCCCTTCCCCCCTCTCTTTAGATGGAAGTGAT
TGTTGGGGACTTTGGGATCGTCGTGGTACCCAGGGATGCAGCGGACACAGACCGGATCATGAATCACTCC
TCCATACTCCGCAAGTACAAAGTGAGTCCTCCA , 2708
TCACCAAAGTTGTTTCTCTCGCTTCCTCCTTCCCTCTCCCCCTTCCCCCCTCTCTTTAGATGGAAGTGAT
TGTTGGGGACTTTGGGATCGTCGTGGTACCCAGGGATGCAGCGGACACGGACCGGATCATGAATCACTCC
TCCATACTCCGCAAGTACAAAGTGAGTCCTCCA , 26
TCACCAAAGTTGTTTCTCTCGCTTCCTCCTTCCCTCTCCCCCTTCCCCCCTCTCTTTAGATGGAAGTGAT
TGTTGGGGACTTTGGGATCGTCGTGGTACCCAGGGATGCAGCGGACACAGACCGGATCATGAATCGCTCC
TCCATACTCCGCAAGTACAAAGTGAGTCCTCCA , 13
TCACCAAAGTTGTTTCTCTCGCTTCCTCCTTCCCTCTCCCCCTTCCCCCCTCTCTTTAGATGGAAGTGAT
TGTTGGGGACTGTGGGATCGTCGTGGTACCCAGGGATGCAGCGGACACAGACCGGATCATGAATCACTCC
TCCATACTCCGCAAGTACAAAGTGAGTCCTCCA , 12
TCACCAAAGTTGTTTCTCTCGCTTCCTCCTTCCCTCTCCCCCTTCCCCCCTCTCTTTAGATGGAAGTGAT
TGTTGGGGACGTTGGGATCGTCGTGGTACCCAGGGATGCAGCGGACACAGACCGGATCATGAATCACTCC
TCCATACTCCGCAAGTACAAAGTGAGTCCTCCA , 11
TCACCAAAGTTGTTTCTCTCGCTTCCTCCTTCCCTCTCCCCCTTCCCCCCTCTCTTTAGATGGAAGTGAT
TGTTGGGGACTTTGGGATCGTCGTGGTACCCAGGGATGCAGCGGACACCGACCGGATCATGAATCACTCC
TCCATACTCCGCAAGTACAAAGTGAGTCCTCCA , 10
TCACCAAAGTTGTTTCTCTCGCTTCCTCCTTCCCTCTCCCCCTTCCCCCCTCTCTTTAGATGGAAGTGAT
TGTTGGGGACTTTGGGATCGTCGTGGTACCCAGGGATGCAGCGGACACAGACCGGATCATGAATCACTCC

TCCATACGCCGCAAGTACAAAGTGAGTCCTCCA , 6
TCACCAAAGTTGTTTCTCTCGCTTCCTCCTTCCCTCTCCCCCTTCCCCCCTCTCTTTAGATGGAAGTGAG
TGTTGGGGACTTTGGGATCGTCGTGGTACCCAGGGATGCAGCGGACACAGACCGGATCATGAATCACTCC
TCCATACTCCGCAAGTACAAAGTGAGTCCTCCA , 5
TCACCAAAGTTGTTTCTCTCGCTTCCTCCTTCCCTCTCCCCCTTCCCCCCTCTCTTTAGATGGAAGTGAT
TGTTGGGGACTTTGGGATCGTCGTGGTCCCCAGGGATGCAGCGGACACAGACCGGATCATGAATCACTCC
TCCATACTCCGCAAGTACAAAGTGAGTCCTCCA , 4
TCACCAAAGTTGTTTCTCTCGCTTCCTCCTTCCCTCTCCCCCTTCCCCCTTCTCTTTAGATGGAAGTGAT
TGTTGGGGACTTTGGGATCGTCGTGGTACCCAGGGATGCAGCGGACACAGACCGGATCATGAATCACTCC
TCCATACTCCGCAAGTACAAAGTGAGTCCTCCA , 4
TCACCAAAGTTGTTTCTCTCGCTTCCTCCTTCCCTCTCCCCTTCCCCCCTCTCTTTAGATGGAAGTGATT
GTTGGGGACTTTGGGATCGTCGTGGTACCCAGGGATGCAGCGGACACAGACCGGATCATGAATCACTCCT
CCATACTCCGCAAGTACAAAGTGAGTCCTCCA , 3
TCACCAAAGTTGTTTCTCTCGCTTCCTCCTTCCCTCTCCCCCTTCCCCCTCTCTTTAGATGGAAGTGATT
GTTGGGGACTTTGGGATCGTCGTGGTACCCAGGGATGCAGCGGACACAGACCGGATCATGAATCACTCCT
CCATACTCCGCAAGTACAAAGTGAGTCCTCCA , 3

GEIC-Plate01-G08 TOTAL:2802 OrderedDict([('WT_sp1', 2769), ('R232Q', 0), ('Silent Block Mod', 0), ('R232Q (Full ssODN)', 0), ('Silent Block Mod (Full ssODN)', 0)]) [(0, 2792), (-1, 9), (1, 1)]
TCACCAAAGTTGTTTCTCTCGCTTCCTCCTTCCCTCTCCCCCTTCCCCCCTCTCTTTAGATGGAAGTGAT
TGTTGGGGACTTTGGGATCGTCGTGGTACCCAGGGATGCAGCGGACACAGACCGGATCATGAATCACTCC
TCCATACTCCGCAAGTACAAAGTGAGTCCTCCA , 2504
TCACCAAAGTTGTTTCTCTCGCTTCCTCCTTCCCTCTCCCCCTTCCCCCCTCTCTTTAGATGGAAGTGAT
TGTTGGGGACTTTGGGATCGTCGTGGTACCCAGGGATGCAGCGGACACGGACCGGATCATGAATCACTCC
TCCATACTCCGCAAGTACAAAGTGAGTCCTCCA , 17
TCACCAAAGTTGTTTCTCTCGCTTCCTCCTTCCCTCTCCCCCTTCCCCCCTCTCTTTAGATGGAAGTGAT
TGTTGGGGACTTTGGGATCGTCGTGGTACCCAGGGATGCAGCGGACACAGACCGGATCATGAATCGCTCC
TCCATACTCCGCAAGTACAAAGTGAGTCCTCCA , 9
TCACCAAAGTTGTTTCTCTCGCTTCCTCCTTCCCTCTCCCCCTTCCCCCCTCTCTTTAGATGGAAGTGAT
TGTTGGGGACGTTGGGATCGTCGTGGTACCCAGGGATGCAGCGGACACAGACCGGATCATGAATCACTCC
TCCATACTCCGCAAGTACAAAGTGAGTCCTCCA , 9
TCACCAAAGTTGTTTCTCTCGCTTCCTCCTTCCCTCTCCCCCTTCCCCCCTCTCTTTAGATGGAAGTGAT
TGTTGGGGACTGTGGGATCGTCGTGGTACCCAGGGATGCAGCGGACACAGACCGGATCATGAATCACTCC
TCCATACTCCGCAAGTACAAAGTGAGTCCTCCA , 8
TCACCAAAGTTGTTTCTCTCGCTTCCTCCTTCCCTCTCCCCCTTCCCCCCTCTCTTTAGATGGAAGTGAT
TGTTGGGGACTTTGGGATCGTCGTGGTACCCAGGGATGCAGCGGACACAGACCGGATCATGAATCACTCC
TCCATACGCCGCAAGTACAAAGTGAGTCCTCCA , 6
TCACCAAAGTTGTTTCTCTTGCTTCCTCCTTCCCTCTCCCCCTTCCCCCCTCTCTTTAGATGGAAGTGAT
TGTTGGGGACTTTGGGATCGTCGTGGTACCCAGGGATGCAGCGGACACAGACCGGATCATGAATCACTCC
TCCATACTCCGCAAGTACAAAGTGAGTCCTCCA , 5
TCACCAAAGTTGTTTCTCTCGCTTCCTCCTTCCCTCTCCCCCTTCCCCCCTCTCTTTAGATGGAAGTGAT
TGTTGGGGACTTTGGGGTCGTCGTGGTACCCAGGGATGCAGCGGACACAGACCGGATCATGAATCACTCC
TCCATACTCCGCAAGTACAAAGTGAGTCCTCCA , 4
TCACCAAAGTTGTTTCTCTCGCTTCCTCCTTCCCTCTCCCCCTTCCCCCCTCTCTTTAGATGGAAGTGAT
TGTTGGGGACTTTGGGATCGTCGTGGTACCCAGGGATGCAGCGGACACCGACCGGATCATGAATCACTCC
TCCATACTCCGCAAGTACAAAGTGAGTCCTCCA , 4
TCACCAAAGTTGTTTCTCTCGCTTCCTCCTTCCCTCTCCCCCTTCCCCCCTCTCTTTAGATGGAAGTGGT
TGTTGGGGACTTTGGGATCGTCGTGGTACCCAGGGATGCAGCGGACACAGACCGGATCATGAATCACTCC

TCCATACTCCGCAAGTACAAAGTGAGTCCTCCA , 4
TCACCAAAGTTGTTTCTCTCGCTTCCTCCTTCCCTCTCCCCCTTCCCCCCTCTCTTTAGATGGAAGTGAT
TGTTGGGGACTTTGGGATCGTCGTGGTACCCAGGGATGCAGCGGACACAGGCCGGATCATGAATCACTCC
TCCATACTCCGCAAGTACAAAGTGAGTCCTCCA , 3
TCACCAAAGTTGTTTCTCTCGCTTCCTCCTTCCCTCTCCCCCTTCCCCCCTCTCTTTAGATGGAAGTGAT
TGTTGGAGACTTTGGGATCGTCGTGGTACCCAGGGATGCAGCGGACACAGACCGGATCATGAATCACTCC
TCCATACTCCGCAAGTACAAAGTGAGTCCTCCA , 3

GEIC–Plate01–H01 TOTAL:2251 OrderedDict([('WT_sp1', 1110), ('R232Q', 1), ('Silent Block Mod', 1097), ('R232Q (Full ssODN)', 1), ('Silent Block Mod (Full ssODN)', 1003)]) [(0, 2245), (–1, 5), (–2, 1)]
TCACCAAAGTTGTTTCTCTCGCTTCCTCCTTCCCTCTCCCCCTTCCCCCCTCTCTTTAGATGGAAGTGAT
TGTTGGGGACTTTGGGATCGTCGTGGTACCCCGTGATGCAGCGGACACAGACCGGATCATGAATCACTCC
TCCATACTCCGCAAGTACAAAGTGAGTCCTCCA , 995
TCACCAAAGTTGTTTCTCTCGCTTCCTCCTTCCCTCTCCCCCTTCCCCCCTCTCTTTAGATGGAAGTGAT
TGTTGGGGACTTTGGGATCGTCGTGGTACCCAGGGATGCAGCGGACACAGACCGGATCATGAATCACTCC
TCCATACTCCGCAAGTACAAAGTGAGTCCTCCA , 988
TCACCAAAGTTGTTTCTCTCGCTTCCTCCTTCCCTCTCCCCCTTCCCCCCTCTCTTTAGATGGAAGTGAT
TGTTGGGGACTGTGGGATCGTCGTGGTACCCCGTGATGCAGCGGACACAGACCGGATCATGAATCACTCC
TCCATACTCCGCAAGTACAAAGTGAGTCCTCCA , 5
TCACCAAAGTTGTTTCTCTCGCTTCCTCCTTCCCTCTCCCCCTTCCCCCCTCTCTTTAGATGGAAGTGAT
TGTTGGGGACGTTGGGATCGTCGTGGTACCCAGGGATGCAGCGGACACAGACCGGATCATGAATCACTCC
TCCATACTCCGCAAGTACAAAGTGAGTCCTCCA , 5
TCACCAAAGTTGTTTCTCTCGCTTCCTCCTTCCCTCTCCCCCTTCCCCCCTCTCTTTAGATGGAAGTGAT
TGTTGGGGACTTTGGGATCGTCGTGGTACCCAGGGATGCAGCGGACACAGACCGGATCATGAATCGCTCC
TCCATACTCCGCAAGTACAAAGTGAGTCCTCCA , 4
TCACCAAAGTTGTTTCTCTCGCTTCCTCCTTCCCTCTCCCCCTTCCCCCCTCTCTTTAGATGGAAGTGAT
TGTTGGGGACTGTGGGATCGTCGTGGTACCCAGGGATGCAGCGGACACAGACCGGATCATGAATCACTCC
TCCATACTCCGCAAGTACAAAGTGAGTCCTCCA , 4
TCACCAAAGTTGTTTCTCTCGCTTCCTCCTTCCCTCTCCCCCTTCCCCCCTCTCTTTAGATGGAAGTGAT
TGTTGGGGACTTTGGGATCGTCGTGGTACCCCGTGATGCAGCGGACACAGACCGGATCATGAATCACTCC
TCCATGCTCCGCAAGTACAAAGTGAGTCCTCCA , 4
TCACCAAAGTTGTTTCTCTCGCTTCCTCCTTCCCTCTCCCCCTTCCCCCCTCTCTTTAGATGGAAGTGAT
TGTTGGGGACTTTGGGATCGTCGTGGTACCCCGTGATGCAGCGGACACAGACCGGATCATGAATCGCTCC
TCCATACTCCGCAAGTACAAAGTGAGTCCTCCA , 4
TCACCAAAGTTGTTTCTCTCGCTTCCTCCTTCCCTCTCCCCCTTCCCCCCTCTCTTTAGATGGAAGTGAT
TGTTGGGGACTTTGGGATCGTCGTGGTACCCAGGGATGCAGCGGACACCGACCGGATCATGAATCACTCC
TCCATACTCCGCAAGTACAAAGTGAGTCCTCCA , 3
TCACCAAAGTTGTTTCTCTCGCTTCCTCCTTCCCTCTCCCCCTTCCCCCCTCTCTTTAGATGGAAGTGAT
TGTTGGGGACTTTGGGATCGTCGTGGTACCCAGGGATGCAGCGGACACGGACCGGATCATGAATCACTCC
TCCATACTCCGCAAGTACAAAGTGAGTCCTCCA , 3
TCACCAAAGTTGTTTCTCTCGCTTCCTCCTTCCCTCTCCCCCTTCCCCCCTCTCTTTAGATGGAAGTGAT
TGTTGGGGACTTTGGGATCGTCGTGGTACCCAGGGATGCAGCGGACACAGACCGGATCATGAATCACGCC
TCCATACTCCGCAAGTACAAAGTGAGTCCTCCA , 3
TCACCAAAGTTGTTTCTCTCGCTTCCTCCTTCCCTCTCCCCCTTCCCCCCTCTCTTTAGATGGAAGTGAT
TGTTGGGGACTTTGGGATCGTCGTGGTACCCAGGGATGCAGCGTACACAGACCGGATCATGAATCACTCC
TCCATACTCCGCAAGTACAAAGTGAGTCCTCCA , 3

GEIC-Plate01-H02 TOTAL:78 OrderedDict([('WT_sp1', 39), ('R232Q', 37), ('Silent Block Mod', 1), ('R232Q (Full ssODN)', 36), ('Silent Block Mod (Full ssODN)', 1)])   [(0, 78)]
TCACCAAAGTTGTTTCTCTCGCTTCCTCCTTCCCTCTCCCCCTTCCCCCCTCTCTTTAGATGGAAGTGAT
TGTTGGGGACTTTGGGATCGTCGTGGTACCCCAAGATGCAGCGGACACAGACCGGATCATGAATCACTCC
TCCATACTCCGCAAGTACAAAGTGAGTCCTCCA , 35
TCACCAAAGTTGTTTCTCTCGCTTCCTCCTTCCCTCTCCCCCTTCCCCCCTCTCTTTAGATGGAAGTGAT
TGTTGGGGACTTTGGGATCGTCGTGGTACCCAGGGATGCAGCGGACACAGACCGGATCATGAATCACTCC
TCCATACTCCGCAAGTACAAAGTGAGTCCTCCA , 31
TCACCAAAGTTGTTTCTCTCGCTTCCTCCTTCCCTCTCCCCCTTCCCCCCTCTCTTTAGATGGAAGTGAT
TGTTGGGGACGTTGGGATCGTCGTGGTACCCAGGGATGCAGCGGACACAGACCGGATCATGAATCACTCC
TCCATACTCCGCAAGTACAAAGTGAGTCCTCCA , 1
TCACCAAAGTTGTTCCTCTCGCTTCCTCCTTCCCTCTCCCCCTTCCCCCCTCTCTTTAGATGGAAGTGAT
TGTTGGGGACTTTGGGATCGTCGTGGTACCCAGGGATGCAGCGGACACAGACCGGATCATGAATCACTCC
TCCATACTCCGCAAGTACAAAGTGAGTCCTCCA , 1
TCACCAAAGTTGTTTCTCTCGCTTCCTCCTTCCCTCTCCCCCTTCCCCCCTCTCTTTAGATGGAAGTGAT
TGTTAGGGACTTTGGGATCGTCGTGGTACCCAGGGATGCAGCGGACACAGACCGGATCATGAATCACTCC
TCCATACTCCGCAAGTACAAAGTGAGTCCTCCA , 1
TCACCAAAGTTGTTTCTCTCGCTTCCTCCTTCCCTCTCCCCCTTCCCCCCTCTCTTTAGATGGAAGTGAT
TGTTGGGGACTTTGGGATCGTCGTGGTACCCAGGGATGCAGCGGACACAGACCGGATCATAAATCACTCC
TCCATACTCCGCAAGTACAAAGTGAGTCCTCCA , 1
TCACCAAAGTTGTTTCTCTCGCTTCCTCCTTCCCTCTCCCCCTTCCCCCCTCTCTTTAGATGGAAGTGAT
TGTTGGGGACTTTGGGATCGTCGTGGTACCCAGGGATGCAGCGGACACGGACCGGATCATGAATCACTCC
TCCATACTCCGCAAGTACAAAGTGAGTCCTCCA , 1
TCACCAAAGTTGTTTCTCTCGCTTCCTCCTTCCCTCTCCCCCTTCCCCCCTCTCTTTAGATGGAAGTGAT
TGTTGGGGACTTTGGGATCGTCGTGGTACCCAGGGATGCAGCGGACACAGACCGGATCATGAATCACTCC
TCCATACCCCGCAAGTACAAAGTGAGTCCTCCA , 1
TCACCAAAGTTGTTTCTCTCGCTTCCTCCTTCCCTCTCCCCCTTCCCCCCTCTCTTTAGATGGAAGTGAT
TGTTGGGGACTTTGGGATCGTCGTGGTACCCCGTGATGCAGCGGACACAGACCGGATCATGAATCACTCC
TCCATACTCCGCAAGTACAAAGTGAGTCCTCCA , 1
TCACCAAAGTTGTTTCTCTCGCTTCCTCCTTCCCTCTCCCCCTTCCCCCCTCTCTTTAGATGGAAGTGAT
TGTTGGGGACTTTGGGATCGTCGTGGTACCCAGGGATGCAGCGGACACAGACCGGATCATGAATCACTCC
TCCATATTCCGCAAGTACAAAGTGAGTCCTCCA , 1
TCACCAAAGTTGTTTTTCTCGCTTCCTCCTTCCCTCTCCCCCTTCCCCCCTCTCTTTAGATGGAAGTGAT
TGTTGGGGACTTTGGGATCGTCGTGGTACCCCAAGATGCAGCGGACACAGACCGGATCATGAATCACTCC
TCCATACTCCGCAAGTACAAAGTGAGTCCTCCA , 1
TCACCAAAGTTGTTTCTCTCGCTTCCTCCTTCCCTCTCCCCCTTCCCCCCTCTCTTTAGATGGGAGTGAT
TGTTGGGGACTTTGGGATCGTCGTGGTACCCCAAGATGCAGCGGACACAGACCGGATCATGAATCGCTCC
TCCATACTCCGCAAGTACAAAGTGAGTCCTCCA , 1

GEIC-Plate01-H03 TOTAL:3394 OrderedDict([('WT_sp1', 3345), ('R232Q', 0), ('Silent Block Mod', 0), ('R232Q (Full ssODN)', 0), ('Silent Block Mod (Full ssODN)', 0)])   [(0, 3378), (-1, 14), (-2, 2)]
TCACCAAAGTTGTTTCTCTCGCTTCCTCCTTCCCTCTCCCCCTTCCCCCCTCTCTTTAGATGGAAGTGAT
TGTTGGGGACTTTGGGATCGTCGTGGTACCCAGGGATGCAGCGGACACAGACCGGATCATGAATCACTCC
TCCATACTCCGCAAGTACAAAGTGAGTCCTCCA , 2978
TCACCAAAGTTGTTTCTCTCGCTTCCTCCTTCCCTCTCCCCCTTCCCCCCTCTCTTTAGATGGAAGTGAT
TGTTGGGGACTGTGGGATCGTCGTGGTACCCAGGGATGCAGCGGACACAGACCGGATCATGAATCACTCC
TCCATACTCCGCAAGTACAAAGTGAGTCCTCCA , 16

TCACCAAAGTTGTTTCTCTCGCTTCCTCCTTCCCTCTCCCCCTTCCCCCCTCTCTTTAGATGGAAGTGAT
TGTTGGGGACGTTGGGATCGTCGTGGTACCCAGGGATGCAGCGGACACAGACCGGATCATGAATCACTCC
TCCATACTCCGCAAGTACAAAGTGAGTCCTCCA , 15
TCACCAAAGTTGTTTCTCTCGCTTCCTCCTTCCCTCTCCCCCTTCCCCCCTCTCTTTAGATGGAAGTGAT
TGTTGGGGACTTTGGGATCGTCGTGGTACCCAGGGATGCAGCGGACACGGACCGGATCATGAATCACTCC
TCCATACTCCGCAAGTACAAAGTGAGTCCTCCA , 14
TCACCAAAGTTGTTTCTCTCGCTTCCTCCTTCCCTCTCCCCCTTCCCCCCTCTCTTTAGATGGAAGTGAT
TGTTGGGGACTTTGGGATCGTCGTGGTACCCAGGGATGCAGCGGACACCGACCGGATCATGAATCACTCC
TCCATACTCCGCAAGTACAAAGTGAGTCCTCCA , 9
TCACCAAAGTTGTTTCTCTCGCTTCCTCCTTCCCTCTCCCCCTTCCCCCCTCTCTTTAGATGGAAGTGAT
TGTTGGGGACTTTGGGATCGTCGTGGTACCCAGGGATGCAGCGGACACAGACCGGATCATGAATCGCTCC
TCCATACTCCGCAAGTACAAAGTGAGTCCTCCA , 7
TCACCAAAGTTGTTTCTCTCGCTTCCTCCTTCCCTCTCCCCCTTCCCCCCTCTCTTTAGATGGAAGTGAT
TGTTGGGGACTTTGGGATCGTCGTGGTACCCAGGGATGCAGCGGACACAGGCCGGATCATGAATCACTCC
TCCATACTCCGCAAGTACAAAGTGAGTCCTCCA , 5
TCACCAAAGTTGTTTCTCTCGCTTCCTCCTTCCCTCTCCCCCTTCCCCCCTCTCTTTAGATGGAAGTGAT
TGTTGGGGACTTTGGGATCGTCGTGGTACCCAGGGATGCAGCGGACACAGACCGGATCATGAATCACTCC
TCCATGCTCCGCAAGTACAAAGTGAGTCCTCCA , 5
TCACCAAAGTTGTTTCTCTCGCTTCCTCCTTCCCTCTCCCCCTTCCCCCTCTCTTTAGATGGAAGTGATT
GTTGGGGACTTTGGGATCGTCGTGGTACCCAGGGATGCAGCGGACACAGACCGGATCATGAATCACTCCT
CCATACTCCGCAAGTACAAAGTGAGTCCTCCA , 5
TCACCAAAGTTGTTTCTCTCGCTTCCTCCTTCCCTCTCCCCCTTCCCCCCTCTCTTTAGATGGAAGTGAT
TGTTGGGGACTTTGGGATCGTCGTGGTACCCAGGGATGCAGCGGACACAGACCGGATCATGAATCACTCC
TCCACACTCCGCAAGTACAAAGTGAGTCCTCCA , 4
TCACCAAAGTTGTTTCTCTCGCTTCCTCCTTCCCTCTCCCCCTTCCCCCTTCTCTTTAGATGGAAGTGAT
TGTTGGGGACTTTGGGATCGTCGTGGTACCCAGGGATGCAGCGGACACAGACCGGATCATGAATCACTCC
TCCATACTCCGCAAGTACAAAGTGAGTCCTCCA , 4
TCACCAAAGTTGTTTCTCTCGCTTCCTCCTTCCCTCTCCCCCTTCCCCCCTCTCTTTAGATGGAAGTGGT
TGTTGGGGACTTTGGGATCGTCGTGGTACCCAGGGATGCAGCGGACACAGACCGGATCATGAATCACTCC
TCCATACTCCGCAAGTACAAAGTGAGTCCTCCA , 4

GEIC-Plate01-H04 TOTAL:3517 OrderedDict([('WT_sp1', 1767), ('R232Q', 0), ('Silent Block Mod', 1678), ('R232Q (Full ssODN)', 0), ('Silent Block Mod (Full ssODN)', 1514)])   [(0, 3505), (-1, 12)]
TCACCAAAGTTGTTTCTCTCGCTTCCTCCTTCCCTCTCCCCCTTCCCCCCTCTCTTTAGATGGAAGTGAT
TGTTGGGGACTTTGGGATCGTCGTGGTACCCAGGGATGCAGCGGACACAGACCGGATCATGAATCACTCC
TCCATACTCCGCAAGTACAAAGTGAGTCCTCCA , 1550
TCACCAAAGTTGTTTCTCTCGCTTCCTCCTTCCCTCTCCCCCTTCCCCCCTCTCTTTAGATGGAAGTGAT
TGTTGGGGACTTTGGGATCGTCGTGGTACCCCGTGATGCAGCGGACACAGACCGGATCATGAATCACTCC
TCCATACTCCGCAAGTACAAAGTGAGTCCTCCA , 1500
TCACCAAAGTTGTTTCTCTCGCTTCCTCCTTCCCTCTCCCCCTTCCCCCCTCTCTTTAGATGGAAGTGAT
TGTTGGGGACTTTGGGATCGTCGTGGTACCCAGGGATGCAGCGGACACGGACCGGATCATGAATCACTCC
TCCATACTCCGCAAGTACAAAGTGAGTCCTCCA , 14
TCACCAAAGTTGTTTCTCTCGCTTCCTCCTTCCCTCTCCCCCTTCCCCCCTCTCTTTAGATGGAAGTGAT
TGTTGGGGACTGTGGGATCGTCGTGGTACCCCGTGATGCAGCGGACACAGACCGGATCATGAATCACTCC
TCCATACTCCGCAAGTACAAAGTGAGTCCTCCA , 13
TCACCAAAGTTGTTTCTCTCGCTTCCTCCTTCCCTCTCCCCCTTCCCCCCTCTCTTTAGATGGAAGTGAT
TGTTGGGGACGTTGGGATCGTCGTGGTACCCAGGGATGCAGCGGACACAGACCGGATCATGAATCACTCC
TCCATACTCCGCAAGTACAAAGTGAGTCCTCCA , 11

TCACCAAAGTTGTTTCTCTCGCTTCCTCCTTCCCTCTCCCCCTTCCCCCCTCTCTTTAGATGGAAGTGAT
TGTTGGGGACTTTGGGATCGTCGTGGTACCCCGTGATGCAGCGGACACAGACCGGATCATGAATCGCTCC
TCCATACTCCGCAAGTACAAAGTGAGTCCTCCA , 11
TCACCAAAGTTGTTTCTCTCGCTTCCTCCTTCCCTCTCCCCCTTCCCCCCTCTCTTTAGATGGAAGTGAT
TGTTGGGGACTTTGGGATCGTCGTGGTACCCAGGGATGCAGCGGACACAGACCGGATCATGAATCGCTCC
TCCATACTCCGCAAGTACAAAGTGAGTCCTCCA , 7
TCACCAAAGTTGTTTCTCTCGCTTCCTCCTTCCCTCTCCCCCTTCCCCCCTCTCTTTAGATGGAAGTGAT
TGTTGGGGACTTTGGGATCGTCGTGGTACCACGTGATGCAGCGGACACAGACCGGATCATGAATCACTCC
TCCATACTCCGCAAGTACAAAGTGAGTCCTCCA , 6
TCACCAAAGTTGTTTCTCTCGCTTCCTCCTTCCCTCTCCCCCTTCCCCCCTCTCTTTAGATGGAAGTGAT
TGTTGGGGACGTTGGGATCGTCGTGGTACCCCGTGATGCAGCGGACACAGACCGGATCATGAATCACTCC
TCCATACTCCGCAAGTACAAAGTGAGTCCTCCA , 5
TCACCAAAGTTGTTTCTCTCGCTTCCTCCTTCCCTCTCCCCCTTCCCCCTCTCTTTAGATGGAAGTGATT
GTTGGGGACTTTGGGATCGTCGTGGTACCCAGGGATGCAGCGGACACAGACCGGATCATGAATCACTCCT
CCATACTCCGCAAGTACAAAGTGAGTCCTCCA , 4
TCACCAAAGTTGTTTCTCTCGCTTCCTCCTTCCCTCTCCCCCTTCCCCCCTCTCTTTAGATGGAAGTGAT
TGTTGGGGACTTTGGGATCGTCGTGGTACCCAGGGATGCAGCGGACACAGACCGGATCATGAATCACGCC
TCCATACTCCGCAAGTACAAAGTGAGTCCTCCA , 4
TCACCAAAGTTGTTTCTCTCGCTTCCTCCTTCCCTCTCCCCCTTCCCCCCTCTCTTTAGATGGAAGTGAT
TGTTGGGGACTTTGGGATCGTCGTGGTACCCAGGGATGCAGCGGACACAGACCGGATCATGAATCACTCC
TCCATACTCCGCAAGTACCAAGTGAGTCCTCCA , 4

GEIC-Plate01-H05 TOTAL:3296 OrderedDict([('WT_sp1', 1650), ('R232Q', 1567), ('Silent Block Mod', 0), ('R232Q (Full ssODN)', 1402), ('Silent Block Mod (Full ssODN)', 0)])   [(0, 3284), (-1, 10), (-29, 1), (-3, 1)]
TCACCAAAGTTGTTTCTCTCGCTTCCTCCTTCCCTCTCCCCCTTCCCCCCTCTCTTTAGATGGAAGTGAT
TGTTGGGGACTTTGGGATCGTCGTGGTACCCAGGGATGCAGCGGACACAGACCGGATCATGAATCACTCC
TCCATACTCCGCAAGTACAAAGTGAGTCCTCCA , 1458
TCACCAAAGTTGTTTCTCTCGCTTCCTCCTTCCCTCTCCCCCTTCCCCCCTCTCTTTAGATGGAAGTGAT
TGTTGGGGACTTTGGGATCGTCGTGGTACCCCAAGATGCAGCGGACACAGACCGGATCATGAATCACTCC
TCCATACTCCGCAAGTACAAAGTGAGTCCTCCA , 1387
TCACCAAAGTTGTTTCTCTCGCTTCCTCCTTCCCTCTCCCCCTTCCCCCCTCTCTTTAGATGGAAGTGAT
TGTTGGGGACTTTGGGATCGTCGTGGTACCCAGGGATGCAGCGGACACGGACCGGATCATGAATCACTCC
TCCATACTCCGCAAGTACAAAGTGAGTCCTCCA , 10
TCACCAAAGTTGTTTCTCTCGCTTCCTCCTTCCCTCTCCCCCTTCCCCCCTCTCTTTAGATGGAAGTGAT
TGTTGGGGACGTTGGGATCGTCGTGGTACCCAGGGATGCAGCGGACACAGACCGGATCATGAATCACTCC
TCCATACTCCGCAAGTACAAAGTGAGTCCTCCA , 8
TCACCAAAGTTGTTTCTCTCGCTTCCTCCTTCCCTCTCCCCCTTCCCCCCTCTCTTTAGATGGAAGTGAT
TGTTGGGGACTGTGGGATCGTCGTGGTACCCAGGGATGCAGCGGACACAGACCGGATCATGAATCACTCC
TCCATACTCCGCAAGTACAAAGTGAGTCCTCCA , 8
TCACCAAAGTTGTTTCTCTCGCTTCCTCCTTCCCTCTCCCCCTTCCCCCCTCTCTTTAGATGGAAGTGAT
TGTTGGGGACGTTGGGATCGTCGTGGTACCCCAAGATGCAGCGGACACAGACCGGATCATGAATCACTCC
TCCATACTCCGCAAGTACAAAGTGAGTCCTCCA , 7
TCACCAAAGTTGTTTCTCTCGCTTCCTCCTTCCCTCTCCCCCTTCCCCCCTCTCTTTAGATGGAAGTGAT
TGTTGGGGACTTTGGGATCGTCGTGGTACCCAGGGATGCAGCGGACACAGACCGGATCATGAATCGCTCC
TCCATACTCCGCAAGTACAAAGTGAGTCCTCCA , 7
TCACCAAAGTTGTTTCTCTCGCTTCCTCCTTCCCTCTCCCCCTTCCCCCCTCTCTTTAGATGGAAGTGAT
TGTTGGGGACTTTGGGATCGTCGTGGTACCCAGGGATGCAGCGGACACAGACCGGATCATGAATCACTCC

TCCATGCTCCGCAAGTACAAAGTGAGTCCTCCA , 7
TCACCAAAGTTGTTTCTCTCGCTTCCTCCTTCCCTCTCCCCCTTCCCCCCTCTCTTTAGATGGAAGTGAT
TGTTGGGGACTTTGGGATCGTCGTGGTACCCCAAGATGCAGCGGACACAGACCGGATCATGAATCACTCC
TCCATACTCCGCAAGTACCAAGTGAGTCCTCCA , 5
TCACCAAAGTTGTTTCTCTCGCTTCCTCCTTCCCTCTCCCCCTTCCCCCCTCTCTTTAGATGGAAGTGAT
TGTTGGGGACTGTGGGATCGTCGTGGTACCCCAAGATGCAGCGGACACAGACCGGATCATGAATCACTCC
TCCATACTCCGCAAGTACAAAGTGAGTCCTCCA , 5
TCACCAAAGTTGTTTCTCTCGCTTCCTCCTTCCCTCTCCCCCTTCCCCCCTCTCTTTAGATGGAAGTGAT
TGTTGGGGACTTTGGGATCGTCGTGGTACCCCAAGATGCAGCGGACACAGACCGGATCATGAATCGCTCC
TCCATACTCCGCAAGTACAAAGTGAGTCCTCCA , 5
TCACCAAAGTTGTTTCTCTCGCTTCCTCCTTCCCTCTCCCCCTTCCCCCCTCTCTTTAGATGGAAGTGAT
TGTTGGGGACTTTGGGATCGTCGTGGTACCCCAAGATGCAGCGGACACAGACCGGATCATGAATCACTCC
TCCATACGCCGCAAGTACAAAGTGAGTCCTCCA , 4

GEIC-Plate01-H06 TOTAL:1672 OrderedDict([('WT_sp1', 811), ('R232Q', 0), ('Silent Block Mod', 826), ('R232Q (Full ssODN)', 0), ('Silent Block Mod (Full ssODN)', 747)]) [(0, 1669), (-1, 3)]
TCACCAAAGTTGTTTCTCTCGCTTCCTCCTTCCCTCTCCCCCTTCCCCCCTCTCTTTAGATGGAAGTGAT
TGTTGGGGACTTTGGGATCGTCGTGGTACCCCGTGATGCAGCGGACACAGACCGGATCATGAATCACTCC
TCCATACTCCGCAAGTACAAAGTGAGTCCTCCA , 739
TCACCAAAGTTGTTTCTCTCGCTTCCTCCTTCCCTCTCCCCCTTCCCCCCTCTCTTTAGATGGAAGTGAT
TGTTGGGGACTTTGGGATCGTCGTGGTACCCAGGGATGCAGCGGACACAGACCGGATCATGAATCACTCC
TCCATACTCCGCAAGTACAAAGTGAGTCCTCCA , 717
TCACCAAAGTTGTTTCTCTCGCTTCCTCCTTCCCTCTCCCCCTTCCCCCCTCTCTTTAGATGGAAGTGAT
TGTTGGGGACTTTGGGATCGTCGTGGTACCCCGTGATGCAGCGGACACAGACCGGATCATGAATCGCTCC
TCCATACTCCGCAAGTACAAAGTGAGTCCTCCA , 6
TCACCAAAGTTGTTTCTCTCGCTTCCTCCTTCCCTCTCCCCCTTCCCCCCTCTCTTTAGATGGAAGTGAT
TGTTGGGGACTTTGGGATCGTCGTGGTACCCAGGGATGCAGCGGACACGGACCGGATCATGAATCACTCC
TCCATACTCCGCAAGTACAAAGTGAGTCCTCCA , 5
TCACCAAAGTTGTTTCTCTCGCTTCCTCCTTCCCTCTCCCCCTTCCCCCCTCTCTTTAGATGGAAGTGAT
TGTTGGGGACTTTGGGATCGTCGTGGTACCCAGGGATGCAGCGGACACAGACCTGATCATGAATCACTCC
TCCATACTCCGCAAGTACAAAGTGAGTCCTCCA , 5
TCACCAAAGTTGTTTCTCTCGCTTCCTCCTTCCCTCTCCCCCTTCCCCCCTCTCTTTAGATGGAAGTGAT
TGTTGGGGACTTTGGGATCGTCGTGGTACCCCATGATGCAGCGGACACAGACCGGATCATGAATCACTCC
TCCATACTCCGCAAGTACAAAGTGAGTCCTCCA , 5
TCACCAAAGTTGTTTCTCTCGCTTCCTCCTTCCCTCTCCCCCTTCCCCCCTCTCTTTAGATGGAAGTGAT
TGTTGGGGACTTTGGGATCGTCATGGTACCCCGTGATGCAGCGGACACAGACCGGATCATGAATCACTCC
TCCATACTCCGCAAGTACAAAGTGAGTCCTCCA , 5
TCACCAAAGTTGTTTCTCTCGCTTCCTCCTTCCCTCTCCCCCTTCCCCCCTCTCTTTAGATGGAAGTGAT
TGTTGGGGACTGTGGGATCGTCGTGGTACCCCGTGATGCAGCGGACACAGACCGGATCATGAATCACTCC
TCCATACTCCGCAAGTACAAAGTGAGTCCTCCA , 4
TCACCAAAGTTGTTTCTCTCGCTTCCTCCTTCCCTCTCCCCCTTCCCCCCTCTCTTTAGATGGAAGTGAT
TGTTGGGGACGTTGGGATCGTCGTGGTACCCAGGGATGCAGCGGACACAGACCGGATCATGAATCACTCC
TCCATACTCCGCAAGTACAAAGTGAGTCCTCCA , 4
TCACCAAAGTTGTTTCTCTCGCTTCCTCCTTCCCTCTCCCCCTTCCCCCCTCTCTTTAGATGGAAGTGAT
TGTTGGGGACTTTGGGATCGTCGTGGTACCACGTGATGCAGCGGACACAGACCGGATCATGAATCACTCC
TCCATACTCCGCAAGTACAAAGTGAGTCCTCCA , 4
TCACCAAAGTTGTTTCTCTCGCTTCCTCCTTCCCTCTCCCCCTTCCCCCCTCTCTTTAGATGGAAGTGAT
TGTTGGGGACTTTGGGATCGTCGTGGTACCCAGGGATGTAGCGGACACAGACCGGATCATGAATCACTCC

TCCATACTCCGCAAGTACAAAGTGAGTCCTCCA , 3
TCACCAAAGTTGTTTCTCTCGCTTCCTCCTTCCCTCTCCCCCTTCCCCCCTCTCTTTAGATGGAAGTGAT
TGTTGGGGACTTTGGGATCGTCGTGGTACCCAGTGATGCAGCGGACACAGACCGGATCATGAATCACTCC
TCCATACTCCGCAAGTACAAAGTGAGTCCTCCA , 3

GEIC-Plate01-H07 TOTAL:260 OrderedDict([('WT_sp1', 248), ('R232Q', 0), ('Silent Block Mod', 0), ('R232Q (Full ssODN)', 0), ('Silent Block Mod (Full ssODN)', 0)]) [(0, 259), (-2, 1)]
TCACCAAAGTTGTTTCTCTCGCTTCCTCCTTCCCTCTCCCCCTTCCCCCCTCTCTTTAGATGGAAGTGAT
TGTTGGGGACTTTGGGATCGTCGTGGTACCCAGGGATGCAGCGGACACAGACCGGATCATGAATCACTCC
TCCATACTCCGCAAGTACAAAGTGAGTCCTCCA , 214
TCACCAAAGTTGTTTCTCTCGCTTCCTCCTTCCCTCTCCCCCTTCCCCCCTCTCTTTAGATGGAAGTGAT
TGTTGGGGACTTTGGGATCGTCGTGGTACCCAGGGATGCAGCGGACACGGACCGGATCATGAATCACTCC
TCCATACTCCGCAAGTACAAAGTGAGTCCTCCA , 3
TCACCAAAGTTGTTTCTCTCGCTTCCTCCTTCCCTCTCCCCCTTCCCCCCTCTCTTTAGATGGAAGTGAT
TGTTGGGGACGTTGGGATCGTCGTGGTACCCAGGGATGCAGCGGACACAGACCGGATCATGAATCACTCC
TCCATACTCCGCAAGTACAAAGTGAGTCCTCCA , 2
TCACCAAAGTTGTTTCTCTCGCTTCCTCCTTCCCTCTCCCCCTTCCCCCCTCTCTATAGATGGAAGTGAT
TGTTGGGGACTTTGGGATCGTCGTGGTACCCAGGGATGCAGCAGACTCAGACCGGACCATGAATCACTCC
GCCATACTCCGCAAGTACAAAGTGAGTCCTCCA , 1
TCACCAAAGTTGTTTCTCTCGCTTCCTCCTTCCCTCTCCCCCTTCCCCCCTCTCTTTAGATGGAAGTGAT
TGTTGGGGACTTTGGGATCGTCGTGGTACCCAGGGATGCAGCGGACACAGACCGGATCATGAATCACTCC
TCCATACTCCGCAAGTACAAATTGAGTCCTCCA , 1
TCACCAAAGTTGTTTCTCTCGCTTCCTCCTTCCCTCTCCCCCTTCCCCCCTCTCTTTAGATGGAAGTGAT
TGTTGGGGACTTTGGGATCGTCGTGGTACCCAGGGATGCAGCGGACACAGCCCGGATCATGAATCACTCC
TCCATACTCCGCAAGTACAAAGTGAGTCCTCCA , 1
TCACCAAAGTTGTTTCTCTCGATTGCTCCTTCCCTCTCCCCCTTCCCCCCTCTCTTTAGGTTGAAGTGAT
TGTTGGGGACTTTGGGATCGTCGTGGTACCCAGAGATGCAGCGGACACAGAACGAATCATGGATCACTCC
TCCATACTCCGCATGTACGAAGTGAGTCCTCCA , 1
TCACCAAAGTTGTTTCTCTCGCTTCCTCCTTCCCTCTCCCCCTTCCCCCCTCTCTTTAGATGGAAGTGAT
TGTTGGGGACTTTGGGATCGTCGTGGTACTCAGGGATGCAGCGGACACAGACCGGATCATGAATCACTCC
TCCATACGCCGCAAGTACAAAGTGAGTCCTCCA , 1
TCACCAAAGTTGTTTCCCTCGCTTCCTCCTTCCCTCTCCCCCTTCCCCCCTCTCTTTAGATGGAAGTGAT
TGTTGGGGACTTTGGGATCGTCGTGGTACCCAGGGATGCAGCGGACACAGACCGGATCATGAATCACTCC
TCCATACTCCGCAAGTACAAAGTGAGTCCTCCA , 1
TCACCAAAGTTGTTTCTCTCGCTTCCTCCTTCCCTCTCCCCCTTCCCCCCTCTCTTTAGATGGAAGTGAT
TGTTGGGGTCTTTGGGATCGTCGTGGTTCCCAGGGATGCAGCGGACACAGACCGGATCATGAATCGCTCC
TCCATACTCCGCAAGTACAAAGTGAGTCCTCCA , 1
TCACCAAAGTTGTTTCTCTCGCCTCCTCCTTCCCTCTCCCCCTTCCCCCCTCTCTTTAGATGGAAGTGAT
TGTTGGGGACTTTGGGATCGTCGTGGTACCCAGGGATGCAGCGGACACAGACCGGATCATGAATCACTCC
TCCATACTCCGCAAGTACAAAGTGAGTCCTCCA , 1
TCACCAAAGTTGTTTCTCTCGCTTCCTCCTTCCCTCTCCCCCTTCCCCCCTCTCTTTAGATGGAAGTGAT
TGTTGGGGACTTTGGGATCGCCGTGGTACCCAGGGATGCAGCGGACACAGACCGGATCATGAATCACTCC
TCCATACTCCGCAAGTACAAAGTGAGTCCTCCA , 1
