# Supplementary material for: Macrophage depletion blocks congenital SARM1-dependent neuropathy
Source: J Clin Invest. 2022 Dec 1;132(23):e159800. doi: 10.1172/JCI159800 (PMC9711884; doi:10.1172/JCI159800)
Supplement: Supplemental data sets 3-4 [file jci-132-159800-s112.pdf]

XC101918 mNmnat2 V98M
the_Seq_start: AGCCTCTCTT
the_Seq_end: TGGCTGAGG
Test_Sequences:
sp2: CCTGCAGTGTGT
T95M: TGTGCAGTGTGT
T95M only: TCTGCAGTGTGT
T95M Full ssODN:
GCCTCTCTTGTCCCGGCAGGGTGGACCCATGGGAGTGCTATCAGGACACCTGGCAGACAATGTGCAGTGT
GTTGGAGCACCATCGAGACCTGATGAAGGTAAGATGGGCTGTGGCTGAGG
V98M: CATGCAGTATGT
V98M_only: CCTGCAGTATGT
V98M Full ssODN:
GCCTCTCTTGTCCCGGCAGGGTGGACCCATGGGAGTGCTATCAGGACACCTGGCAGACAACATGCAGTAT
GTTGGAGCACCATCGAGACCTGATGAAGGTAAGATGGGCTGTGGCTGAGG
Silent Block only: CTTGTAGCGTGT
Silent Block only Full ssODN:
CCTCTCTTGTCCCGGCAGGGTGGACCCATGGGAGTGCTATCAGGACACCTGGCAGACAACTTGTAGCGTG
TTGGAGCACCATCGAGACCTGATGAAGGTAAGATGGGCTGTGGCTGAGGTTGCGTGG

GEIC-Plate04-A01 TOTAL:3474 OrderedDict([('sp2', 1755), ('T95M', 1699), ('T95M only', 0), ('T95M Full ssODN', 1640), ('V98M', 0), ('V98M_only', 1), ('V98M Full ssODN', 0), ('Silent Block only', 0), ('Silent Block only Full ssODN', 0)])   [(0, 3474)]
AGCCTCTCTTGTCCCGGCAGGGTGGACCCATGGGAGTGCTATCAGGACACCTGGCAGACAACCTGCAGTG
TGTTGGAGCACCATCGAGACCTGATGAAGGTAAGATGGGCTGTGGCTGAGG , 1707
AGCCTCTCTTGTCCCGGCAGGGTGGACCCATGGGAGTGCTATCAGGACACCTGGCAGACAATGTGCAGTG
TGTTGGAGCACCATCGAGACCTGATGAAGGTAAGATGGGCTGTGGCTGAGG , 1640
AGCCTCTCTTGTCCCGGCAGGGTGGACCCATGGGAGTGCTATCAGGACACCTGGCGGACAACCTGCAGTG
TGTTGGAGCACCATCGAGACCTGATGAAGGTAAGATGGGCTGTGGCTGAGG , 3
AGCCTCTCTTGTCCCGGCAGGGGGGACCCATGGGAGTGCTATCAGGACACCTGGCAGACAACCTGCAGTG
TGTTGGAGCACCATCGAGACCTGATGAAGGTAAGATGGGCTGTGGCTGAGG , 3
AGCCTCTCTTGTCCCGGCAGGGTGGACCCATGGGAGTGCTATCAGGACACCTGGCAGACAACCTGCAGTG
GGTTGGAGCACCATCGAGACCTGATGAAGGTAAGATGGGCTGTGGCTGAGG , 3
AGCCTCTCTTGTCCCGGCAGGGCGGACCCATGGGAGTGCTATCAGGACACCTGGCAGACAACCTGCAGTG
TGTTGGAGCACCATCGAGACCTGATGAAGGTAAGATGGGCTGTGGCTGAGG , 3
AGCCTCTCTTGTCCCGGCAGGGTGGACCCATGGGAGTGCTATCAGGACACCTGGCAGACAATGTGCAGTG
TGTTAGAGCACCATCGAGACCTGATGAAGGTAAGATGGGCTGTGGCTGAGG , 2
AGCCTCTCTTGTCCCGGCAGGGTGGACCCATGGGAGTACTATCAGGACACCTGGCAGACAATGTGCAGTG
TGTTGGAGCACCATCGAGACCTGATGAAGGTAAGATGGGCTGTGGCTGAGG , 2
AGCCTCTCTTGTCCCGGCAGGGTGGACCCATGGGAGTGCTATCAGGACGCCTGGCAGACAATGTGCAGTG
TGTTGGAGCACCATCGAGACCTGATGAAGGTAAGATGGGCTGTGGCTGAGG , 2
AGCCTCTCTTGTCCCGGCAGGGTGGACCCATGGGAGTGCTATCAGGACACCTGGCAGACAACCTGCAGTG
TGTTGGAGCACCATCGGGACCTGATGAAGGTAAGATGGGCTGTGGCTGAGG , 2
AGCCTCTCTTGTCCCGGCAGGGTGGACCCATGGGAGTGCTATCAGGACACCTGGCAGACAACCTGCAGTG
TGTTGGAGCACCATCGAGACCTGATGAGGGTAAGATGGGCTGTGGCTGAGG , 2
AGCCTCTCTTGTCCCGGCAGGGTGGACCCATGGGAGAGCTATCAGGACACCTGGCAGACAATGTGCAGTG
TGTTGGAGCACCATCGAGACCTGATGAAGGTAAGATGGGCTGTGGCTGAGG , 2

GEIC-Plate04-A02 TOTAL:3057 OrderedDict([('sp2', 3039), ('T95M', 0), ('T95M only', 0), ('T95M Full ssODN', 0), ('V98M', 1), ('V98M_only', 1), ('V98M Full ssODN', 1), ('Silent Block only', 0), ('Silent Block only Full ssODN', 0)])   [(0, 3055), (-1, 2)]
AGCCTCTCTTGTCCCGGCAGGGTGGACCCATGGGAGTGCTATCAGGACACCTGGCAGACAACCTGCAGTG
TGTTGGAGCACCATCGAGACCTGATGAAGGTAAGATGGGCTGTGGCTGAGG , 2915
AGCCTCTCTTGTCCCGGCAGGGCGGACCCATGGGAGTGCTATCAGGACACCTGGCAGACAACCTGCAGTG
TGTTGGAGCACCATCGAGACCTGATGAAGGTAAGATGGGCTGTGGCTGAGG , 10
AGCCTCTCTTGTCCCGGCAGGGTGGACCCATGGGAGTGCTATCAGGACACCTGGCAGACAACCTGCAGTG
TGTTGGAGCACCATCGAGGCCTGATGAAGGTAAGATGGGCTGTGGCTGAGG , 9
AGCCTCTCTTGTCCCGGCAGGGGGGACCCATGGGAGTGCTATCAGGACACCTGGCAGACAACCTGCAGTG
TGTTGGAGCACCATCGAGACCTGATGAAGGTAAGATGGGCTGTGGCTGAGG , 6
AGCCTCTCTTGTCCCGGCAGGGTGGACCCATGGGGGTGCTATCAGGACACCTGGCAGACAACCTGCAGTG
TGTTGGAGCACCATCGAGACCTGATGAAGGTAAGATGGGCTGTGGCTGAGG , 4
AGCCTCTCTTGCCCCGGCAGGGTGGACCCATGGGAGTGCTATCAGGACACCTGGCAGACAACCTGCAGTG
TGTTGGAGCACCATCGAGACCTGATGAAGGTAAGATGGGCTGTGGCTGAGG , 4
AGCCTCTCTTGTCCCGGCAGGGTGGACCCATGGGAGTGCTATCAGGACACCTGGCGGACAACCTGCAGTG
TGTTGGAGCACCATCGAGACCTGATGAAGGTAAGATGGGCTGTGGCTGAGG , 3
AGCCTCTCTTGTCCCGGCAGGGTGGACCCATGGGAGTGCCATCAGGACACCTGGCAGACAACCTGCAGTG
TGTTGGAGCACCATCGAGACCTGATGAAGGTAAGATGGGCTGTGGCTGAGG , 3
AGCCTCTCTTGTCCCGGCAGGGTGGACCCATGGGAGTGCTATCAGGACACCTGGCAGACAACCTGCAGTG
CGTTGGAGCACCATCGAGACCTGATGAAGGTAAGATGGGCTGTGGCTGAGG , 3
AGCCTCTCTTGTCCCGGCAGGGTGGACCCATGGGAGTGCTATCAGGACACCTGGCAGACAACCTGCAGTG
TGTTGGAGCACCATCGAGACCTGATGAAGGTAAGGTGGGCTGTGGCTGAGG , 3
AGCCTCTCTTGTCCCGGCAGGGTGGATCCATGGGAGTGCTATCAGGACACCTGGCAGACAACCTGCAGTG
TGTTGGAGCACCATCGAGACCTGATGAAGGTAAGATGGGCTGTGGCTGAGG , 3
AGCCTCTCTTGTCCCGGCAGGGTGGACCCATGGGAGGGCTATCAGGACACCTGGCAGACAACCTGCAGTG
TGTTGGAGCACCATCGAGACCTGATGAAGGTAAGATGGGCTGTGGCTGAGG , 3

GEIC-Plate04-A03 TOTAL:3123 OrderedDict([('sp2', 3091), ('T95M', 0), ('T95M only', 1), ('T95M Full ssODN', 0), ('V98M', 0), ('V98M_only', 2), ('V98M Full ssODN', 0), ('Silent Block only', 0), ('Silent Block only Full ssODN', 0)])   [(0, 3119), (-1, 4)]
AGCCTCTCTTGTCCCGGCAGGGTGGACCCATGGGAGTGCTATCAGGACACCTGGCAGACAACCTGCAGTG
TGTTGGAGCACCATCGAGACCTGATGAAGGTAAGATGGGCTGTGGCTGAGG , 2958
AGCCTCTCTTGTCCCGGCAGGGTGGACCCATGGGAGTGCTATCAGGACACCTGGCAGACAACCTGCAGTG
TGTTGGAGCACCATCGAGGCCTGATGAAGGTAAGATGGGCTGTGGCTGAGG , 5
AGCCTCTCTTGTCCCGGCAGGGGGGACCCATGGGAGTGCTATCAGGACACCTGGCAGACAACCTGCAGTG
TGTTGGAGCACCATCGAGACCTGATGAAGGTAAGATGGGCTGTGGCTGAGG , 4
AGCCTCTCTTGTCCCGGCAGGGTGGACCCATGGGAGTGCTATCAGGACACCTGGCAGACAACCTGCAGTG
TGCTGGAGCACCATCGAGACCTGATGAAGGTAAGATGGGCTGTGGCTGAGG , 4
AGCCTCTCTTGTCCCGGCAGGGTGGACCCATGGGAGTGCTATCAGGACACCTGGCAGACAACCTGCAGCG
TGTTGGAGCACCATCGAGACCTGATGAAGGTAAGATGGGCTGTGGCTGAGG , 4
AGCCTCTCTTGTCCCGGCAGGGAGGACCCATGGGAGTGCTATCAGGACACCTGGCAGACAACCTGCAGTG
TGTTGGAGCACCATCGAGACCTGATGAAGGTAAGATGGGCTGTGGCTGAGG , 4
AGCCTCTCTTGTCCCGGCAGGGTGGACCCATGGGAGTGCTATCAGGACACCTGGCAGACAACCTGCAGTG
GGTTGGAGCACCATCGAGACCTGATGAAGGTAAGATGGGCTGTGGCTGAGG , 4

AGCCTCTCTTGTCCCGGCAGGGTGGACCCATGGGAGTGCTATCAGGACACCTGGCAGACAGCCTGCAGTG
TGTTGGAGCACCATCGAGACCTGATGAAGGTAAGATGGGCTGTGGCTGAGG , 4
AGCCTCTCTTGTCCCGGCAGGGCGGACCCATGGGAGTGCTATCAGGACACCTGGCAGACAACCTGCAGTG
TGTTGGAGCACCATCGAGACCTGATGAAGGTAAGATGGGCTGTGGCTGAGG , 4
AGCCTCTCTTGTCCCGGCAGGGTGGACCCATGGGAGTGCTATCAGGACACCCGGCAGACAACCTGCAGTG
TGTTGGAGCACCATCGAGACCTGATGAAGGTAAGATGGGCTGTGGCTGAGG , 3
AGCCTCTCTTGTCCCAGCAGGGTGGACCCATGGGAGTGCTATCAGGACACCTGGCAGACAACCTGCAGTG
TGTTGGAGCACCATCGAGACCTGATGAAGGTAAGATGGGCTGTGGCTGAGG , 3
AGCCTCTCTTGTCCCGGCAGGGTGGACCCATGGGAGTGCTATCAGGACACCTGGCAGACAACCCGCAGTG
TGTTGGAGCACCATCGAGACCTGATGAAGGTAAGATGGGCTGTGGCTGAGG , 3

GEIC-Plate04-A04 TOTAL:3079 OrderedDict([('sp2', 3062), ('T95M', 0), ('T95M only', 2), ('T95M Full ssODN', 0), ('V98M', 1), ('V98M_only', 0), ('V98M Full ssODN', 1), ('Silent Block only', 0), ('Silent Block only Full ssODN', 0)])   [(0, 3078), (-1, 1)]
AGCCTCTCTTGTCCCGGCAGGGTGGACCCATGGGAGTGCTATCAGGACACCTGGCAGACAACCTGCAGTG
TGTTGGAGCACCATCGAGACCTGATGAAGGTAAGATGGGCTGTGGCTGAGG , 2952
AGCCTCTCTTGTCCCGGCAGGGTGGACCCATGGGAGTGCTATCAGGACACCTGGCAGACAACCTGCAGTG
TGTTGGAGCGCCATCGAGACCTGATGAAGGTAAGATGGGCTGTGGCTGAGG , 4
AGCCTCTCTTGTCCCGGCAGGGGGGACCCATGGGAGTGCTATCAGGACACCTGGCAGACAACCTGCAGTG
TGTTGGAGCACCATCGAGACCTGATGAAGGTAAGATGGGCTGTGGCTGAGG , 4
AGCCTCTCTTGTCCCGGCAGGGTGGACCCATGGGAGTGCTATCAGGACACCTGGCAGACAACCTGCAGTG
TGTTGGAGTACCATCGAGACCTGATGAAGGTAAGATGGGCTGTGGCTGAGG , 3
AGCCTCTCTTGTCCCGGCAGGGTGGACCCATGGGGGTGCTATCAGGACACCTGGCAGACAACCTGCAGTG
TGTTGGAGCACCATCGAGACCTGATGAAGGTAAGATGGGCTGTGGCTGAGG , 3
AGCCTCTCTTGTCCCGGCAGGGTGGACCCATGGGAGTGCTATCAGGACACCTGGCAGACAACCTGCAGTG
TGTTGGAGCACCATCGAGGCCTGATGAAGGTAAGATGGGCTGTGGCTGAGG , 3
AGCCTCTCTTGTCCCAGCAGGGTGGACCCATGGGAGTGCTATCAGGACACCTGGCAGACAACCTGCAGTG
TGTTGGAGCACCATCGAGACCTGATGAAGGTAAGATGGGCTGTGGCTGAGG , 3
AGCCTCTCTTGTCCCGGCAGGGCGGACCCATGGGAGTGCTATCAGGACACCTGGCAGACAACCTGCAGTG
TGTTGGAGCACCATCGAGACCTGATGAAGGTAAGATGGGCTGTGGCTGAGG , 3
AGCCTCTCTTGTCCCGGCAGGGTGGACCCATGGGAGTGCTATCAGGACACCTGGCAGACAACCTGCAGTG
TGTTGGAGCACCATCGGGACCTGATGAAGGTAAGATGGGCTGTGGCTGAGG , 2
AGCCTCTCTTGTCCCGGCAGGGTGGACCCACGGGAGTGCTATCAGGACACCTGGCAGACAACCTGCAGTG
TGTTGGAGCACCATCGAGACCTGATGAAGGTAAGATGGGCTGTGGCTGAGG , 2
AGCCTCTCTTGTCCCGGCAGGGTGGACCCATGGGAGTGCTATCGGGACACCTGGCAGACAACCTGCAGTG
TGTTGGAGCACCATCGAGACCTGATGAAGGTAAGATGGGCTGTGGCTGAGG , 2
AGCCTCTCTTGTCCCGGCAGGGTGGACCCATGGGAGTGCTATCAGGACACCTGGCAGACAACCTGCAGTG
TGTTGGAGCACCATCGAGACCCGATGAAGGTAAGATGGGCTGTGGCTGAGG , 2

GEIC-Plate04-A05 TOTAL:1726 OrderedDict([('sp2', 1712), ('T95M', 0), ('T95M only', 0), ('T95M Full ssODN', 0), ('V98M', 2), ('V98M_only', 0), ('V98M Full ssODN', 1), ('Silent Block only', 0), ('Silent Block only Full ssODN', 0)])   [(0, 1724), (-4, 1), (-1, 1)]
AGCCTCTCTTGTCCCGGCAGGGTGGACCCATGGGAGTGCTATCAGGACACCTGGCAGACAACCTGCAGTG
TGTTGGAGCACCATCGAGACCTGATGAAGGTAAGATGGGCTGTGGCTGAGG , 1634
AGCCTCTCTTGTCCCGGCAGGGTGGACCCATGGGAGTGCTATCAGGACACCTGGCAGACAACCTGCAGTG
TGTTGGAGCACCATCGGGACCTGATGAAGGTAAGATGGGCTGTGGCTGAGG , 3

AGCCTCTCTTGTCCCGGCAGGGTGGAACCATGGGAGTGCTATCAGGACACCTGGCAGACAACCTGCAGTG
TGTTGGAGCACCATCGAGACCTGATGAAGGTAAGATGGGCTGTGGCTGAGG , 3
AGCCTCTCTTGTCCCGGCAGGGTGGACCCATGGGAGTGCTATCAGGACACCTGGCAGACAACCTGCAGTG
TGTTGGAGCACCATCGAGACCTGATGAGGGTAAGATGGGCTGTGGCTGAGG , 3
AGCCTCTCTTGTCCCGGCAGGGGGGACCCATGGGAGTGCTATCAGGACACCTGGCAGACAACCTGCAGTG
TGTTGGAGCACCATCGAGACCTGATGAAGGTAAGATGGGCTGTGGCTGAGG , 3
AGCCTCTCTTGTCCCGGCGGGGTGGACCCATGGGAGTGCTATCAGGACACCTGGCAGACAACCTGCAGTG
TGTTGGAGCACCATCGAGACCTGATGAAGGTAAGATGGGCTGTGGCTGAGG , 2
AGCCTCTCTTGTCCCGGCAGGGTGGACCCATGGGAGCGCTATCAGGACACCTGGCAGACAACCTGCAGTG
TGTTGGAGCACCATCGAGACCTGATGAAGGTAAGATGGGCTGTGGCTGAGG , 2
AGCCTCTCTTGTCCCGGCAGGGTGGACCCATGGGAGTGCTATCAGGACACCTGGCAGACAACCTGCAGTG
TGTTGGAGCACCATCGAGACCTGATGAAGGTAAGACGGGCTGTGGCTGAGG , 2
AGCCTCTCTTGTCCCGGCAGGGTGGACCCATGGGAGTGCTATCAGGACACCTGGCAGACAACCTGCAGTG
TGTTGGAGCACCATCGAGACCTGATGAAGGTAAGATGGGCGGTGGCTGAGG , 2
AGCCTCTCTTGTCCCGGCAGGGTGGACCCATGGGAGTGCTATCAGGACACCTGGCAGACAACCTGCAGTG
CGTTGGAGCACCATCGAGACCTGATGAAGGTAAGATGGGCTGTGGCTGAGG , 2
AGCCTCTCTTGACCCGGCAGGGTGGACCCATGGGAGTGCTATCAGGACACCTGGCAGACAACCTGCAGTG
TGTTGGAGCACCATCGAGACCTGATGAAGGTAAGATGGGCTGTGGCTGAGG , 2
AGCCTCTCTTGTCCCGGCAGGGAGGACCCATGGGAGTGCTATCAGGACACCTGGCAGACAACCTGCAGTG
TGTTGGAGCACCATCGAGACCTGATGAAGGTAAGATGGGCTGTGGCTGAGG , 2

GEIC-Plate04-A06 TOTAL:3111 OrderedDict([('sp2', 3086), ('T95M', 0), ('T95M only', 1), ('T95M Full ssODN', 0), ('V98M', 3), ('V98M_only', 0), ('V98M Full ssODN', 3), ('Silent Block only', 0), ('Silent Block only Full ssODN', 0)]) [(0, 3108), (-1, 3)]
AGCCTCTCTTGTCCCGGCAGGGTGGACCCATGGGAGTGCTATCAGGACACCTGGCAGACAACCTGCAGTG
TGTTGGAGCACCATCGAGACCTGATGAAGGTAAGATGGGCTGTGGCTGAGG , 2989
AGCCTCTCTTGTCCCGGCAGGGGGGACCCATGGGAGTGCTATCAGGACACCTGGCAGACAACCTGCAGTG
TGTTGGAGCACCATCGAGACCTGATGAAGGTAAGATGGGCTGTGGCTGAGG , 5
AGCCTCTCTTGTCCCGGTAGGGTGGACCCATGGGAGTGCTATCAGGACACCTGGCAGACAACCTGCAGTG
TGTTGGAGCACCATCGAGACCTGATGAAGGTAAGATGGGCTGTGGCTGAGG , 3
AGCCTCTCTTGTCCCGGCAGGGTGGACCCATGGGAGTGCCATCAGGACACCTGGCAGACAACCTGCAGTG
TGTTGGAGCACCATCGAGACCTGATGAAGGTAAGATGGGCTGTGGCTGAGG , 3
AGCCTCTCTTGTCCCGGCAGGGTGGACCCATGGGAGTGCTATCAGGACACCTGGCAGACAACATGCAGTA
TGTTGGAGCACCATCGAGACCTGATGAAGGTAAGATGGGCTGTGGCTGAGG , 3
AGCCTCTCTTGTCCCGGCAGGGTGGACCCATGGGAGTGCTATCAGGACACCTGGCAGACAACCTGCAGTG
TGTTGGAGCACCATCGAGGCCTGATGAAGGTAAGATGGGCTGTGGCTGAGG , 3
AGCCTCTCTTGTCCCGGCAGGGTGGACCCATGGGAGTGCTATCAGGACACCTGGCAGACAACCTGCAGTG
GGTTGGAGCACCATCGAGACCTGATGAAGGTAAGATGGGCTGTGGCTGAGG , 3
AGCCTCTCTTGTCCCGGCAGGGTGGACCCATGGGAGTGCTATCAGGACACCTGGCAGGCAACCTGCAGTG
TGTTGGAGCACCATCGAGACCTGATGAAGGTAAGATGGGCTGTGGCTGAGG , 3
AGCCTCTCTTGTCCCGGCAGGGTGGACCCATGGGAGTACTATCAGGACACCTGGCAGACAACCTGCAGTG
TGTTGGAGCACCATCGAGACCTGATGAAGGTAAGATGGGCTGTGGCTGAGG , 2
AGCCTCTCTTGTCCCGGCAGGGTGGACCCATGGGAGTGCTATCAGGACACCTGGCAGACAACCTGCAGTG
TGTTGGAGCACCATCGAGACCTGATGAGGGTAAGATGGGCTGTGGCTGAGG , 2
AGCCTCTCTTGTCCCGGCAGGGTGGACCCATGGGAGTGCTATCGGGACACCTGGCAGACAACCTGCAGTG
TGTTGGAGCACCATCGAGACCTGATGAAGGTAAGATGGGCTGTGGCTGAGG , 2
AGCCTCTCTTGTCCCGGCAGGGTGGGCCCATGGGAGTGCTATCAGGACACCTGGCAGACAACCTGCAGTG
TGTTGGAGCACCATCGAGACCTGATGAAGGTAAGATGGGCTGTGGCTGAGG , 2

GEIC-Plate04-A07 TOTAL:2121 OrderedDict([('sp2', 2100), ('T95M', 0), ('T95M only', 1), ('T95M Full ssODN', 0), ('V98M', 3), ('V98M_only', 0), ('V98M Full ssODN', 3), ('Silent Block only', 0), ('Silent Block only Full ssODN', 0)])   [(0, 2117), (-1, 4)]
AGCCTCTCTTGTCCCGGCAGGGTGGACCCATGGGAGTGCTATCAGGACACCTGGCAGACAACCTGCAGTG
TGTTGGAGCACCATCGAGACCTGATGAAGGTAAGATGGGCTGTGGCTGAGG , 2019
AGCCTCTCTTGTCCCGGCAGGGTGGACCCATGGGAGTGCTATCAGGACACCTGGCAGACAACCTGCAGTG
TGTTGGAGCACCATCGGGACCTGATGAAGGTAAGATGGGCTGTGGCTGAGG , 4
AGCCTCTCTTGTCCCGGCAGGGGGGACCCATGGGAGTGCTATCAGGACACCTGGCAGACAACCTGCAGTG
TGTTGGAGCACCATCGAGACCTGATGAAGGTAAGATGGGCTGTGGCTGAGG , 4
AGCCTCTCTTGTCCCGGCAGGGTGGACCCATGGGAGTGCTATCAGGACACCTGGCAGACAACCTGCAGTG
GGTTGGAGCACCATCGAGACCTGATGAAGGTAAGATGGGCTGTGGCTGAGG , 3
AGCCTCTCTTGTCCCGGCAGGGTGGACCCATGGGGGTGCTATCAGGACACCTGGCAGACAACCTGCAGTG
TGTTGGAGCACCATCGAGACCTGATGAAGGTAAGATGGGCTGTGGCTGAGG , 3
AGCCTCTCTTGTCCCGGCAGGGTGGACCCATGGGAGTGCTATCAGGACACCTGGCAGACAACATGCAGTA
TGTTGGAGCACCATCGAGACCTGATGAAGGTAAGATGGGCTGTGGCTGAGG , 3
AGCCTCTCTTGTCCCGGCAGGGTGGACCCGTGGGAGTGCTATCAGGACACCTGGCAGACAACCTGCAGTG
TGTTGGAGCACCATCGAGACCTGATGAAGGTAAGATGGGCTGTGGCTGAGG , 3
AGCCTCTCTTGTCCCGGCAGGGCGGACCCATGGGAGTGCTATCAGGACACCTGGCAGACAACCTGCAGTG
TGTTGGAGCACCATCGAGACCTGATGAAGGTAAGATGGGCTGTGGCTGAGG , 3
AGCCTCTCTTGTCCCGGCAGGGTGGACCCATGGGAGTGCTATCAGGACACCTGGCAGACAACCTGCAGTG
TGTTGGAGCACTATCGAGACCTGATGAAGGTAAGATGGGCTGTGGCTGAGG , 2
AGCCTCTCTTGTCACGGCAGGGTGGACCCATGGGAGTGCTATCAGGACACCTGGCAGACAACCTGCAGTG
TGTTGGAGCACCATCGAGACCTGATGAAGGTAAGATGGGCTGTGGCTGAGG , 2
AGCCTCTCTTGTCCCGGCAGGGTGGACCCATGGGAGTGCTATCAGGACACCTGGCAGACAACCTGCAGTG
TGTTGGAGCACCATCGAGACCTGATGAAGGTAAGATGGCTGTGGCTGAGG , 2
AGCCTCTCTTGTCCCGGCAGGGTGGACCCATGGGAGTGCTATCAGGACACCTGGCAGACAACCTGTAGTG
TGTTGGAGCACCATCGAGACCTGATGAAGGTAAGATGGGCTGTGGCTGAGG , 2

GEIC-Plate04-A08 TOTAL:2992 OrderedDict([('sp2', 1534), ('T95M', 1439), ('T95M only', 1), ('T95M Full ssODN', 1388), ('V98M', 1), ('V98M_only', 0), ('V98M Full ssODN', 1), ('Silent Block only', 0), ('Silent Block only Full ssODN', 0)])   [(0, 2992)]
AGCCTCTCTTGTCCCGGCAGGGTGGACCCATGGGAGTGCTATCAGGACACCTGGCAGACAACCTGCAGTG
TGTTGGAGCACCATCGAGACCTGATGAAGGTAAGATGGGCTGTGGCTGAGG , 1477
AGCCTCTCTTGTCCCGGCAGGGTGGACCCATGGGAGTGCTATCAGGACACCTGGCAGACAATGTGCAGTG
TGTTGGAGCACCATCGAGACCTGATGAAGGTAAGATGGGCTGTGGCTGAGG , 1388
AGCCTCTCTTGTCCCGGCAGGGGGGACCCATGGGAGTGCTATCAGGACACCTGGCAGACAATGTGCAGTG
TGTTGGAGCACCATCGAGACCTGATGAAGGTAAGATGGGCTGTGGCTGAGG , 4
AGCCTCTCTTGTCCCGGCAGGGCGGACCCATGGGAGTGCTATCAGGACACCTGGCAGACAACCTGCAGTG
TGTTGGAGCACCATCGAGACCTGATGAAGGTAAGATGGGCTGTGGCTGAGG , 4
AGCCTCTCTTGTCCCGGCAGGGTGGACCCATGGGAGTGCTATCAGGACACCTGGCAGACAATGTGCAGTG
TGTTGGAGCACCATCGAGACCCGATGAAGGTAAGATGGGCTGTGGCTGAGG , 3
AGCCTCTCTTGTCCCGGCAGGGGGGACCCATGGGAGTGCTATCAGGACACCTGGCAGACAACCTGCAGTG
TGTTGGAGCACCATCGAGACCTGATGAAGGTAAGATGGGCTGTGGCTGAGG , 3
AGCCTCTCTTGTCCCGGCAGGGTGGACCCATGGGAGTGCTATCAGGACACCTGGCAGATAATGTGCAGTG
TGTTGGAGCACCATCGAGACCTGATGAAGGTAAGATGGGCTGTGGCTGAGG , 3

AGCCTCTCTTGTCCCGGCAGGGTGGACCCATGGGAGTGCTATCAGGACACCTGGCATACAATGTGCAGTG
TGTTGGAGCACCATCGAGACCTGATGAAGGTAAGATGGGCTGTGGCTGAGG , 3
AGCCTCTCTTGTCCCGGCAGGGTGGACCCATGGGAGTGCTATCAGGACACCTGGCAGACAATGGGCAGTG
TGTTGGAGCACCATCGAGACCTGATGAAGGTAAGATGGGCTGTGGCTGAGG , 2
AGCCTCTCTTGTCCCGGTAGGGTGGACCCATGGGAGTGCTATCAGGACACCTGGCAGACAACCTGCAGTG
TGTTGGAGCACCATCGAGACCTGATGAAGGTAAGATGGGCTGTGGCTGAGG , 2
AGCCTCTCTTGTCCCGGCAGGGTGGACCCATGGGAGTGCTATCAGGACACCTGGCAGACAACCTGCAGCG
TGTTGGAGCACCATCGAGACCTGATGAAGGTAAGATGGGCTGTGGCTGAGG , 2
AGCCTCTCTTGTCCCGGCAGGGTGGACCCATGGGAGTGCTATCAGGACACCTGGCAGACAACCTGCAGTG
TGTTGGAGCACCATCGAGACCTGATGAAGGTAAGGTGGGCTGTGGCTGAGG , 2

GEIC-Plate04-B01 TOTAL:3719 OrderedDict([('sp2', 1816), ('T95M', 6), ('T95M only', 0), ('T95M Full ssODN', 6), ('V98M', 1869), ('V98M_only', 5), ('V98M Full ssODN', 1814), ('Silent Block only', 0), ('Silent Block only Full ssODN', 0)])   [(0, 3716), (-1, 3)]
AGCCTCTCTTGTCCCGGCAGGGTGGACCCATGGGAGTGCTATCAGGACACCTGGCAGACAACATGCAGTA
TGTTGGAGCACCATCGAGACCTGATGAAGGTAAGATGGGCTGTGGCTGAGG , 1814
AGCCTCTCTTGTCCCGGCAGGGTGGACCCATGGGAGTGCTATCAGGACACCTGGCAGACAACCTGCAGTG
TGTTGGAGCACCATCGAGACCTGATGAAGGTAAGATGGGCTGTGGCTGAGG , 1742
AGCCTCTCTTGTCCCGGCAGGGTGGACCCATGGGAGTGCTATCAGGACACCTGGCAGACAATGTGCAGTG
TGTTGGAGCACCATCGAGACCTGATGAAGGTAAGATGGGCTGTGGCTGAGG , 6
AGCCTCTCTTGTCCCGGCAGGGTGGACCCATGGGAGTGCTATCAGGACACCTGGCAGACAACCTGCAGTA
TGTTGGAGCACCATCGAGACCTGATGAAGGTAAGATGGGCTGTGGCTGAGG , 5
AGCCTCTCTTGTCCCGGCAGGGGGGACCCATGGGAGTGCTATCAGGACACCTGGCAGACAACCTGCAGTG
TGTTGGAGCACCATCGAGACCTGATGAAGGTAAGATGGGCTGTGGCTGAGG , 4
AGCCTCTCTTGTCCCGGCAGGGTGGACCCATGGGAGTGCTATCAGGACACCTGGCAGACAACCTGCAGTG
TGTTGGAGCACCATCGAGGCCTGATGAAGGTAAGATGGGCTGTGGCTGAGG , 4
AGCCTCTCTTGTCCCGGCAGGGTGGACCCATGGGAGTGCTATCAGGACACCTGGCAGACAACATGCAGTA
TGTTGGGGCACCATCGAGACCTGATGAAGGTAAGATGGGCTGTGGCTGAGG , 4
AGCCTCTCTTGTCCCGGCAGGGCGGACCCATGGGAGTGCTATCAGGACACCTGGCAGACAACATGCAGTA
TGTTGGAGCACCATCGAGACCTGATGAAGGTAAGATGGGCTGTGGCTGAGG , 3
AGCCTCTCTTGTCCCGGCAGGGTGGACCCATGGGAGTGCTATCAGGACACCTGGCAGACAACATGCAGTG
TGTTGGAGCACCATCGAGACCTGATGAAGGTAAGATGGGCTGTGGCTGAGG , 3
AGCCTCTCTTGTCCCGGCAGGGCGGACCCATGGGAGTGCTATCAGGACACCTGGCAGACAACCTGCAGTG
TGTTGGAGCACCATCGAGACCTGATGAAGGTAAGATGGGCTGTGGCTGAGG , 3
AGCCTCTCTTGTCCCGGTAGGGTGGACCCATGGGAGTGCTATCAGGACACCTGGCAGACAACCTGCAGTG
TGTTGGAGCACCATCGAGACCTGATGAAGGTAAGATGGGCTGTGGCTGAGG , 2
AGCCTCTCTTGTCCCGGCAGGGTGGACCCATGGGATTGCTATCAGGACACCTGGCAGACAACATGCAGTA
TGTTGGAGCACCATCGAGACCTGATGAAGGTAAGATGGGCTGTGGCTGAGG , 2

GEIC-Plate04-B02 TOTAL:2023 OrderedDict([('sp2', 2004), ('T95M', 0), ('T95M only', 1), ('T95M Full ssODN', 0), ('V98M', 0), ('V98M_only', 0), ('V98M Full ssODN', 0), ('Silent Block only', 0), ('Silent Block only Full ssODN', 0)])   [(0, 2019), (-1, 4)]
AGCCTCTCTTGTCCCGGCAGGGTGGACCCATGGGAGTGCTATCAGGACACCTGGCAGACAACCTGCAGTG
TGTTGGAGCACCATCGAGACCTGATGAAGGTAAGATGGGCTGTGGCTGAGG , 1915
AGCCTCTCTTGTCCCGGCAGGGGGGACCCATGGGAGTGCTATCAGGACACCTGGCAGACAACCTGCAGTG
TGTTGGAGCACCATCGAGACCTGATGAAGGTAAGATGGGCTGTGGCTGAGG , 7

AGCCTCTCTTGTCCCGGCAGGGTGGACCCATGGGAGTGCTATCAGGACACCTGGCAGACAACCTGCAGTG
TGTTGGAGCACCATCGAGGCCTGATGAAGGTAAGATGGGCTGTGGCTGAGG , 7
AGCCTCTCTTGTCCCGGCAGGGTGGACCCATGGGAGTGCTATCAGGACACCTGGCAGACAACCTGCAGTG
TGTTGGGGCACCATCGAGACCTGATGAAGGTAAGATGGGCTGTGGCTGAGG , 4
AGCCTCTCTTGTCCCGGCAGGGTGGACCCATGGGAGTGCTATCAGGACACCTGGCAGACAACCTGCAGTG
TGTTGGAGCACCATCGAGACCTGATGAAGGTAAGATGAGCTGTGGCTGAGG , 3
AGCCTCTCTTGTCCCGGCAGGGTGGATCCATGGGAGTGCTATCAGGACACCTGGCAGACAACCTGCAGTG
TGTTGGAGCACCATCGAGACCTGATGAAGGTAAGATGGGCTGTGGCTGAGG , 3
AGCCTCTCTTGTCCCGGCAGGGTGGACCCATGGGAGTGCTATCAGGACACCTGGCAGACAACCTGCGGTG
TGTTGGAGCACCATCGAGACCTGATGAAGGTAAGATGGGCTGTGGCTGAGG , 3
AGCCTCTCTTGTCCCGGCAGGGTGGACCCATGGGCGTGCTATCAGGACACCTGGCAGACAACCTGCAGTG
TGTTGGAGCACCATCGAGACCTGATGAAGGTAAGATGGGCTGTGGCTGAGG , 2
AGCCTCTCTTGTCCCGGCAGGGTGGACCCATGGGAGTGCTATCAGGACACCTGGCAGACAACCTGCAGTG
TGTTGGAGCACCATCGAGACCTGACGAAGGTAAGATGGGCTGTGGCTGAGG , 2
AGCCTCTCTTGTCCCGGCAGGGTGGACCCATGGGAGTGCTATCAGGACACCTGGCAGACAACCTGCAGTG
TGTTGGAGCACCATCGAGACCTGGTGAAGGTAAGATGGGCTGTGGCTGAGG , 2
AGCCTCTCTTGTCCCGGCAGGGTGGACCCATGGGAGTGCTATCAGGACACCTGGCAGACAACCTGCAGTG
TGTTGGAGCACCATCGGGACCTGATGAAGGTAAGATGGGCTGTGGCTGAGG , 2
AGCCTCTCTTGTCCCGGCAGGGTGGACCCATGGGAGTGCTATCAGGGCACCTGGCAGACAACCTGCAGTG
TGTTGGAGCACCATCGAGACCTGATGAAGGTAAGATGGGCTGTGGCTGAGG , 2

GEIC-Plate04-B03 TOTAL:3252 OrderedDict([('sp2', 3224), ('T95M', 0), ('T95M only', 0), ('T95M Full ssODN', 0), ('V98M', 1), ('V98M_only', 0), ('V98M Full ssODN', 1), ('Silent Block only', 0), ('Silent Block only Full ssODN', 0)]) [(0, 3249), (-1, 3)]
AGCCTCTCTTGTCCCGGCAGGGTGGACCCATGGGAGTGCTATCAGGACACCTGGCAGACAACCTGCAGTG
TGTTGGAGCACCATCGAGACCTGATGAAGGTAAGATGGGCTGTGGCTGAGG , 3114
AGCCTCTCTTGTCCCGGCAGGGGGGACCCATGGGAGTGCTATCAGGACACCTGGCAGACAACCTGCAGTG
TGTTGGAGCACCATCGAGACCTGATGAAGGTAAGATGGGCTGTGGCTGAGG , 7
AGCCTCTCTTGTCCCGGCAGGGTGGACCCATGGGAGTGCTATCAGGACACCTGGCAGACAACCTGCAGTG
GGTTGGAGCACCATCGAGACCTGATGAAGGTAAGATGGGCTGTGGCTGAGG , 6
AGCCTCTCTTGTCCCGGCAGGGTGGACCCATGGGAGTGCTATCAGGACACCCGGCAGACAACCTGCAGTG
TGTTGGAGCACCATCGAGACCTGATGAAGGTAAGATGGGCTGTGGCTGAGG , 5
AGCCTCTCTTGTCCCGGCAGGGTGGACCCATGGGAGTGCTATCAGGACACCTGGCAGACAACCTGCAGCG
TGTTGGAGCACCATCGAGACCTGATGAAGGTAAGATGGGCTGTGGCTGAGG , 4
AGCCTCTCTTGTCCCGGCAGGGTGGACCCATGGGAGTGCTATCAGGACACCTGGCAGACAACCTGCAGTG
TGTTGGAGCGCCATCGAGACCTGATGAAGGTAAGATGGGCTGTGGCTGAGG , 3
AGCCTCTCTTGTCCCGGCAGGGTGGACCCATGGGAGTGCTATCAGGACACCTGGCAGACAACCTGCAGTG
TGTTGGAGCACCATCGAGGCCTGATGAAGGTAAGATGGGCTGTGGCTGAGG , 3
AGCCTCTCTTGTCCCGGCAGGGTGGACCCATGGGAGTGCTATCAGGACACCTGGCAGACAACCTGCAGTG
TGTTGGAGCACCATCGAGTCCTGATGAAGGTAAGATGGGCTGTGGCTGAGG , 3
AGCCTCTCTTGTCCCGGCAGGGTGGACCCATGGGAGTGCTATCAGGACACCTGGCAGACAACCTGCAGTG
TGTTGGAGCACCATCGGGACCTGATGAAGGTAAGATGGGCTGTGGCTGAGG , 2
AGCCTCTCTTGTCCCGGCAGGGTGGACCCATGGGAGTGCTATCAGGACACCTGGCAGACAACCTGCAGTG
TGTTGGAGCACCATCAAGACCTGATGAAGGTAAGATGGGCTGTGGCTGAGG , 2
AGCCTCTCTTGTCCCGGCAGGGTGGACCCATGGGAGTGCTATCAGGACACCTGGCAGACAACCTGTAGTG
TGTTGGAGCACCATCGAGACCTGATGAAGGTAAGATGGGCTGTGGCTGAGG , 2
AGCCTCTCTTGTCCCGGCAGGGTGGACCCATGGGAGTGCTATCAGGACACCTGGTAGACAACCTGCAGTG
TGTTGGAGCACCATCGAGACCTGATGAAGGTAAGATGGGCTGTGGCTGAGG , 2

GEIC-Plate04-B04 TOTAL:3078 OrderedDict([('sp2', 3065), ('T95M', 0), ('T95M only', 1), ('T95M Full ssODN', 0), ('V98M', 1), ('V98M_only', 1), ('V98M Full ssODN', 1), ('Silent Block only', 0), ('Silent Block only Full ssODN', 0)])   [(0, 3076), (-1, 2)]
AGCCTCTCTTGTCCCGGCAGGGTGGACCCATGGGAGTGCTATCAGGACACCTGGCAGACAACCTGCAGTG
TGTTGGAGCACCATCGAGACCTGATGAAGGTAAGATGGGCTGTGGCTGAGG , 2946
AGCCTCTCTTGTCCCGGCAGGGGGGACCCATGGGAGTGCTATCAGGACACCTGGCAGACAACCTGCAGTG
TGTTGGAGCACCATCGAGACCTGATGAAGGTAAGATGGGCTGTGGCTGAGG , 7
AGCCTCTCTTGTCCCGGCAGGGTGGACCCATGGGAGTGCTATCAGGACACCCGGCAGACAACCTGCAGTG
TGTTGGAGCACCATCGAGACCTGATGAAGGTAAGATGGGCTGTGGCTGAGG , 4
AGCCTCTCTTGTCCCGGCAGGGTGGACCCATGGGAGTGCTATCAGGACACCTGGCAGACAACCTGCAGTG
TGTTGGAGCACCATCGAGGCCTGATGAAGGTAAGATGGGCTGTGGCTGAGG , 3
AGCCTCTCTTGTCCCGGCAGGGTGGACCCATGGGAGTGCTATCAGGACACCTGGCAGACAACCTGCAGTG
TGTTGGGGCACCATCGAGACCTGATGAAGGTAAGATGGGCTGTGGCTGAGG , 3
AGCCTCTCTTGTCCCGGCAGGGTGGACTCATGGGAGTGCTATCAGGACACCTGGCAGACAACCTGCAGTG
TGTTGGAGCACCATCGAGACCTGATGAAGGTAAGATGGGCTGTGGCTGAGG , 3
AGCCTCTCTTGTCCCGGCAGGGAGGACCCATGGGAGTGCTATCAGGACACCTGGCAGACAACCTGCAGTG
TGTTGGAGCACCATCGAGACCTGATGAAGGTAAGATGGGCTGTGGCTGAGG , 3
AGCCTCTCTTGTCCCGGCAGGGTGGACCCATGGGAGTGCTATCAGGACACCTGACAGACAACCTGCAGTG
TGTTGGAGCACCATCGAGACCTGATGAAGGTAAGATGGGCTGTGGCTGAGG , 3
AGCCTCTCTTGTCCCGGCAGGGTGGACCCATGGGAGTGCTATCAGGACACCTGGCAGACAACGTGCAGTG
TGTTGGAGCACCATCGAGACCTGATGAAGGTAAGATGGGCTGTGGCTGAGG , 2
AGCCTCTCTTGTCCCGGCAGGGTGGACCCATGGGAGTACTATCAGGACACCTGGCAGACAACCTGCAGTG
TGTTGGAGCACCATCGAGACCTGATGAAGGTAAGATGGGCTGTGGCTGAGG , 2
AGCCTCTCTTGTCCCGGCAGGGTGGACCCATGGGAGTGCTATCAGGACACCTGGCAGACAACCTGCAGTG
TGTTGGAGCACCATCGAGACCTGATGAAAGTAAGATGGGCTGTGGCTGAGG , 2
AGCCTCTCTTGTCCCGGCAGGGTGGACCCATGGGAGTGCTATCAGGACACCTGGCAGACAACCTGCTGTG
TGTTGGAGCACCATCGAGACCTGATGAAGGTAAGATGGGCTGTGGCTGAGG , 2

GEIC-Plate04-B05 TOTAL:3563 OrderedDict([('sp2', 3529), ('T95M', 0), ('T95M only', 0), ('T95M Full ssODN', 0), ('V98M', 1), ('V98M_only', 0), ('V98M Full ssODN', 1), ('Silent Block only', 0), ('Silent Block only Full ssODN', 0)])   [(0, 3560), (-1, 3)]
AGCCTCTCTTGTCCCGGCAGGGTGGACCCATGGGAGTGCTATCAGGACACCTGGCAGACAACCTGCAGTG
TGTTGGAGCACCATCGAGACCTGATGAAGGTAAGATGGGCTGTGGCTGAGG , 3399
AGCCTCTCTTGTCCCGGCAGGGTGGACCCATGGGAGTGCTATCAGGACACCTGGCAGACAACCTGCAGTG
TGTTGGAGCACCATCGAGGCCTGATGAAGGTAAGATGGGCTGTGGCTGAGG , 8
AGCCTCTCTTGTCCCGGCAGGGTGGACCCATGGGAGTGCTATCAGGACACCTGGCAGACAACCTGCAGTG
TGTTGGAGCACCATTGAGACCTGATGAAGGTAAGATGGGCTGTGGCTGAGG , 4
AGCCTCTCTTGTCCCGGCAGGGTGGACCCATGGGAGTGCTATCAGGACACCTGGCAGACAACCTGCAGCG
TGTTGGAGCACCATCGAGACCTGATGAAGGTAAGATGGGCTGTGGCTGAGG , 4
AGCCTCTCTTGTCCCGGCAGGGCGGACCCATGGGAGTGCTATCAGGACACCTGGCAGACAACCTGCAGTG
TGTTGGAGCACCATCGAGACCTGATGAAGGTAAGATGGGCTGTGGCTGAGG , 4
AGCCTCTCTTGTCCCGGCAGGGTGGACCCATGGGAGTGCTATCGGGACACCTGGCAGACAACCTGCAGTG
TGTTGGAGCACCATCGAGACCTGATGAAGGTAAGATGGGCTGTGGCTGAGG , 3
AGCCTCTCTTGTCCCGGCAGGGGGGACCCATGGGAGTGCTATCAGGACACCTGGCAGACAACCTGCAGTG
TGTTGGAGCACCATCGAGACCTGATGAAGGTAAGATGGGCTGTGGCTGAGG , 3

AGCCTCTCTTGTCCCGGCAGGGTGGACCCATGGGAGTGCTATCAGGACACCTGGCAGACAACCTGCAGTG
TGTTGGAGCGCCATCGAGACCTGATGAAGGTAAGATGGGCTGTGGCTGAGG , 3
AGCCTCTCTTGTCCCAGCAGGGTGGACCCATGGGAGTGCTATCAGGACACCTGGCAGACAACCTGCAGTG
TGTTGGAGCACCATCGAGACCTGATGAAGGTAAGATGGGCTGTGGCTGAGG , 3
AGCCTCTCTTGTCCCGGCAGGGTGGACCCATGGGAGTGCTATCAGGACACCTGGCAGACAACCTGCAGTG
TGTTGGAGCACCATCGAGACCTGGTGAAGGTAAGATGGGCTGTGGCTGAGG , 3
AGCCTCTCTTGTCCCGGCAGGGTGGACCCATGGGAGTGCTATCAGGACGCCTGGCAGACAACCTGCAGTG
TGTTGGAGCACCATCGAGACCTGATGAAGGTAAGATGGGCTGTGGCTGAGG , 3
AGCCTCTCTTGTCCCGGCAGGGTGGACCCATGGGAGTGCTATCAGGACACCTGGCAGACAACCTGCAGTG
TGTTGGAGCACCATCGAGACCTGATGAAGGTATGATGGGCTGTGGCTGAGG , 3

GEIC-Plate04-B06 TOTAL:1642 OrderedDict([('sp2', 1625), ('T95M', 0), ('T95M only', 0), ('T95M Full ssODN', 0), ('V98M', 2), ('V98M_only', 0), ('V98M Full ssODN', 2), ('Silent Block only', 0), ('Silent Block only Full ssODN', 0)])   [(0, 1639), (-1, 3)]
AGCCTCTCTTGTCCCGGCAGGGTGGACCCATGGGAGTGCTATCAGGACACCTGGCAGACAACCTGCAGTG
TGTTGGAGCACCATCGAGACCTGATGAAGGTAAGATGGGCTGTGGCTGAGG , 1547
AGCCTCTCTTGTCCCGGCAGGGTGGACCCATGGGAGTGCTATCAGGACACCTGGCAGACAACCTGCAGTG
TGTTGGGGCACCATCGAGACCTGATGAAGGTAAGATGGGCTGTGGCTGAGG , 4
AGCCTCTCTTGTCCCGGCAGGGTGGCCCATGGGAGTGCTATCAGGACACCTGGCAGACAACCTGCAGTG
TGTTGGAGCACCATCGAGACCTGATGAAGGTAAGATGGGCTGTGGCTGAGG , 4
AGCCTCTCTTGTCCCGGCAGGGAGGACCCATGGGAGTGCTATCAGGACACCTGGCAGACAACCTGCAGTG
TGTTGGAGCACCATCGAGACCTGATGAAGGTAAGATGGGCTGTGGCTGAGG , 4
AGCCTCTCTTGTCCCGGCAGGGTGGACCCATGGGAGTGCTATCAGGACACCTGGCAGACAACCTGCAGTG
TGTTGGAGCACCATCGAGGCCTGATGAAGGTAAGATGGGCTGTGGCTGAGG , 3
AGCCTCTCTTGTCCCGGCAGGGTGGACCCATGGGAGTGCTATCAGGACACCTGGCGGACAACCTGCAGTG
TGTTGGAGCACCATCGAGACCTGATGAAGGTAAGATGGGCTGTGGCTGAGG , 3
AGCCTCTCTTGTCCCGGCAGGGTGGAACCATGGGAGTGCTATCAGGACACCTGGCAGACAACCTGCAGTG
TGTTGGAGCACCATCGAGACCTGATGAAGGTAAGATGGGCTGTGGCTGAGG , 3
AGCCTCTCTTGTCCCGGCAGGGTGGACCCATGGGAGTGCTATCAGGACACCTGGCAGACAACCTGCAGTG
TGTTGGAGCACCATCGAAACCTGATGAAGGTAAGATGGGCTGTGGCTGAGG , 3
AGCCTCTCTTGTCCCGGCAGGGTGGACCCATGGGAGTGCTATCAGGACACCTGGCAGACAACCCGCAGTG
TGTTGGAGCACCATCGAGACCTGATGAAGGTAAGATGGGCTGTGGCTGAGG , 2
AGCCTCTCTTGTCCCGGCAGGGTGGACCCATGGGAGTGCTATCAGGACACCTGGCAGACAACCTGCAGCG
TGTTGGAGCACCATCGAGACCTGATGAAGGTAAGATGGGCTGTGGCTGAGG , 2
AGCCTCTCTTGTCCCGGCAGGGTGGACCCATGGGAGTGCTATCAGGACACCTGGCAGACAACCTGCATTG
TGTTGGAGCACCATCGAGACCTGATGAAGGTAAGATGGGCTGTGGCTGAGG , 2
AGCCTCTCTTGTCCCGGCAGGGTGGACCCATGGGAGTGCTATCAGGACACCTGGCAGACAACCTGCAGTG
TGTTGGAGCACCATCGAGTCCTGATGAAGGTAAGATGGGCTGTGGCTGAGG , 2

GEIC-Plate04-B07 TOTAL:2869 OrderedDict([('sp2', 1462), ('T95M', 1390), ('T95M only', 1), ('T95M Full ssODN', 1342), ('V98M', 0), ('V98M_only', 0), ('V98M Full ssODN', 0), ('Silent Block only', 0), ('Silent Block only Full ssODN', 0)])   [(0, 2865), (-1, 4)]
AGCCTCTCTTGTCCCGGCAGGGTGGACCCATGGGAGTGCTATCAGGACACCTGGCAGACAACCTGCAGTG
TGTTGGAGCACCATCGAGACCTGATGAAGGTAAGATGGGCTGTGGCTGAGG , 1425
AGCCTCTCTTGTCCCGGCAGGGTGGACCCATGGGAGTGCTATCAGGACACCTGGCAGACAATGTGCAGTG
TGTTGGAGCACCATCGAGACCTGATGAAGGTAAGATGGGCTGTGGCTGAGG , 1342

AGCCTCTCTTGTCCCGGCAGGGTGGACCCATGGGGGTGCTATCAGGACACCTGGCAGACAATGTGCAGTG
TGTTGGAGCACCATCGAGACCTGATGAAGGTAAGATGGGCTGTGGCTGAGG , 3
AGCCTCTCTTGTCCCGGCAGGGTGGACCCATGGGAGTGCTATCAGGACACCTGGCGGACAACCTGCAGTG
TGTTGGAGCACCATCGAGACCTGATGAAGGTAAGATGGGCTGTGGCTGAGG , 2
AGCCTCTCTTGTCCCGGCAGGGGGGACCCATGGGAGTGCTATCAGGACACCTGGCAGACAACCTGCAGTG
TGTTGGAGCACCATCGAGACCTGATGAAGGTAAGATGGGCTGTGGCTGAGG , 2
AGCCTCTCTTGTCCCGGCAGGGTGGACCCATGGGAGTGCTATCAGGACACCTGGCAGACAACCTGCAGTG
GGTTGGAGCACCATCGAGACCTGATGAAGGTAAGATGGGCTGTGGCTGAGG , 2
AGCCTCTCTTGTCCCGGCAGGGTGGACCCATGGGAGTGCTATCAGGACACCTGACAGACAATGTGCAGTG
TGTTGGAGCACCATCGAGACCTGATGAAGGTAAGATGGGCTGTGGCTGAGG , 2
AGCCTCTCTTGTCCCGGCAGGGGGGACCCATGGGAGTGCTATCAGGACACCTGGCAGACAATGTGCAGTG
TGTTGGAGCACCATCGAGACCTGATGAAGGTAAGATGGGCTGTGGCTGAGG , 2
AGCCTCTCTTGTCCCGGCAGGGTGGACCCTTGGGAGTGCTATCAGGACACCTGGCAGACAATGTGCAGTG
TGTTGGAGCACCATCGAGACCTGATGAAGGTAAGATGGGCTGTGGCTGAGG , 2
AGCCTCTCTTGTCCCGGCAGGGTGGACCCATGGGAGTGCTATCAGGACACCTGGCAGACAATGTGCAGCG
TGTTGGAGCACCATCGAGACCTGATGAAGGTAAGATGGGCTGTGGCTGAGG , 2
AGCCTCTCTTGTCCCGGCAGGGTGGACCCATGGGAGTGCTATCAGGACACCTGGCAGACAATGTGCAGTG
TGTTGGAGCACCATCGAGGCCTGATGAAGGTAAGATGGGCTGTGGCTGAGG , 2
AGCCTCTCTTGTCCCGGCAGGGTGGACCCATGGGAGTGCTAACAGGACACCTGGCAGACAACCTGCAGTG
TGTTGGAGCACCATCGAGACCTGATGAAGGTAAGATGGGCTGTGGCTGAGG , 2

GEIC-Plate04-B08 TOTAL:2838 OrderedDict([('sp2', 1472), ('T95M', 1344), ('T95M only', 0), ('T95M Full ssODN', 1293), ('V98M', 0), ('V98M_only', 0), ('V98M Full ssODN', 0), ('Silent Block only', 0), ('Silent Block only Full ssODN', 0)]) [(0, 2835), (-1, 3)]
AGCCTCTCTTGTCCCGGCAGGGTGGACCCATGGGAGTGCTATCAGGACACCTGGCAGACAACCTGCAGTG
TGTTGGAGCACCATCGAGACCTGATGAAGGTAAGATGGGCTGTGGCTGAGG , 1421
AGCCTCTCTTGTCCCGGCAGGGTGGACCCATGGGAGTGCTATCAGGACACCTGGCAGACAATGTGCAGTG
TGTTGGAGCACCATCGAGACCTGATGAAGGTAAGATGGGCTGTGGCTGAGG , 1293
AGCCTCTCTTGTCCCGGCAGGGTGGACCCATGGGAGTGCTATCAGGACACCTGGCAGACAATGTGCAGTG
GGTTGGAGCACCATCGAGACCTGATGAAGGTAAGATGGGCTGTGGCTGAGG , 3
AGCCTCTCTTGTCCCGGCAGGGTGGACCCATGGGAGTGCTATCAGGACACCTGGCAGACAACCTGCAGTG
CGTTGGAGCACCATCGAGACCTGATGAAGGTAAGATGGGCTGTGGCTGAGG , 3
AGCCTCTCTTGTCCCGGCAGGGGGGACCCATGGGAGTGCTATCAGGACACCTGGCAGACAATGTGCAGTG
TGTTGGAGCACCATCGAGACCTGATGAAGGTAAGATGGGCTGTGGCTGAGG , 3
AGCCTCTCTTGTCCCGGCAGGGTGGACCCATGGAAGTGCTATCAGGACACCTGGCAGACAACCTGCAGTG
TGTTGGAGCACCATCGAGACCTGATGAAGGTAAGATGGGCTGTGGCTGAGG , 3
AGCCTCTCTTGTCCCGGCAGGGAGGACCCATGGGAGTGCTATCAGGACACCTGGCAGACAATGTGCAGTG
TGTTGGAGCACCATCGAGACCTGATGAAGGTAAGATGGGCTGTGGCTGAGG , 3
AGCCTCTCTTGTCCCGGCAGGGTGGACCCATGGGAGTGCTATCAGGACGCCTGGCAGACAACCTGCAGTG
TGTTGGAGCACCATCGAGACCTGATGAAGGTAAGATGGGCTGTGGCTGAGG , 2
AGCCTCTCTTGTCCCGGCAGGGTGGACCCATGGGAGTGCTATCAGGACACCTGGCAGACAATGTGCAGTG
TGTTGGGGCACCATCGAGACCTGATGAAGGTAAGATGGGCTGTGGCTGAGG , 2
AGCCTCTCTTGCCCCGGCAGGGTGGACCCATGGGAGTGCTATCAGGACACCTGGCAGACAACCTGCAGTG
TGTTGGAGCACCATCGAGACCTGATGAAGGTAAGATGGGCTGTGGCTGAGG , 2
AGCCTCTCTTGTCCCGGCAGGGTGGACCCATGGGAGTGCTATCAGGACATCTGGCAGACAATGTGCAGTG
TGTTGGAGCACCATCGAGACCTGATGAAGGTAAGATGGGCTGTGGCTGAGG , 2
AGCCTCTCTTGTCCCGGCAGGGTGGACCCATGGGAGTGCTATCAGGACACCTGGCAGACAACCTGCAGTG
TGTTGGAGCACCATCGAGACCGATGAAGGTAAGATGGGCTGTGGCTGAGG , 1

GEIC-Plate04-C01 TOTAL:1462 OrderedDict([('sp2', 737), ('T95M', 0), ('T95M only', 0), ('T95M Full ssODN', 0), ('V98M', 709), ('V98M_only', 1), ('V98M Full ssODN', 684), ('Silent Block only', 0), ('Silent Block only Full ssODN', 0)])   [(0, 1462)]
AGCCTCTCTTGTCCCGGCAGGGTGGACCCATGGGAGTGCTATCAGGACACCTGGCAGACAACCTGCAGTG
TGTTGGAGCACCATCGAGACCTGATGAAGGTAAGATGGGCTGTGGCTGAGG , 712
AGCCTCTCTTGTCCCGGCAGGGTGGACCCATGGGAGTGCTATCAGGACACCTGGCAGACAACATGCAGTA
TGTTGGAGCACCATCGAGACCTGATGAAGGTAAGATGGGCTGTGGCTGAGG , 684
AGCCTCTCTTGTCCCGGCAGGGTGGACCCATGGGAGTGCTATCAGGACACCTGGCAGACAACCTGCAGTG
TGTTGGAGCGCCATCGAGACCTGATGAAGGTAAGATGGGCTGTGGCTGAGG , 3
AGCCTCTCTTGTCCCGGCAGGGTGGACCCATGGGAGTGCTATCAGGACACCTGGCAGACAGCATGCAGTA
TGTTGGAGCACCATCGAGACCTGATGAAGGTAAGATGGGCTGTGGCTGAGG , 2
AGCCTCTCTTGTCCCGGCAGGGTGGACCCATGGGAGTGCTATCAGGACACCTGGCAGACAACATGCAGTA
TGTTGGAGCACCATCGAGACCTGATGGAGGTAAGATGGGCTGTGGCTGAGG , 2
AGCCTCTCTTGTCCCGGCAGGGCGGACCCATGGGAGTGCTATCAGGACACCTGGCAGACAACCTGCAGTG
TGTTGGAGCACCATCGAGACCTGATGAAGGTAAGATGGGCTGTGGCTGAGG , 2
AGCCTCTCTTGTCCCGGCAGGGTGGACCCATGGGAGTGCTATCAGGACACCTGGCAGACAACATGCAGCA
TGTTGGAGCACCATCGAGACCTGATGAAGGTAAGATGGGCTGTGGCTGAGG , 1
AGCCTCTCTTGTCCCGGCAGGGCGGACCCATGGGAGTGCTATTAGGACACCTGGCAGACAACCTGCAGTG
TGTTGGAGCACCATCGAGACCTGATGAAGGTAAGATGGGCTGTGGCTGAGG , 1
AGCCTCTCTTGTCCCGGCAGGGTGGACCCATGGGAGTGCTATCAGGACACCTGGCAGACAACCTGCAGTG
TGTTGGAGCACCATCGAGACCTGATGAAGGTAAGACGGGCTGTGGCTGAGG , 1
AGCCTCTCTTGTCCCGGCAGGGTGGACCCATGGGAGTGCTATCAGGACACCTGGCAGACAACGTGCAGTA
TGTTGGAGCACCATCGAGACCTGATGAAGGTAAGATGGGCTGTGGCTGAGG , 1
AGCCTCTCTTGTCCCGGCAGGGTGGACCCATGGGAGTGCTATCAGGACACCTGGCAGACAACCTGCAGTG
TGTTGGAGCACCATCGAGACCTGTTGAAGGTAAGATGGGCTGTGGCTGAGG , 1
AGCCTCTCTTGTCCCGGCAGGGTGGACCCATGGGAGTGCTATCAGGACACCTGGCAGACAACCTGCAGTG
TGTTGGAGCACCATTGAGACCTGATGAAGGTAAGATGGGCTGTGGCTGAGG , 1

GEIC-Plate04-C02 TOTAL:3335 OrderedDict([('sp2', 3312), ('T95M', 0), ('T95M only', 1), ('T95M Full ssODN', 0), ('V98M', 0), ('V98M_only', 0), ('V98M Full ssODN', 0), ('Silent Block only', 0), ('Silent Block only Full ssODN', 0)])   [(0, 3334), (-1, 1)]
AGCCTCTCTTGTCCCGGCAGGGTGGACCCATGGGAGTGCTATCAGGACACCTGGCAGACAACCTGCAGTG
TGTTGGAGCACCATCGAGACCTGATGAAGGTAAGATGGGCTGTGGCTGAGG , 3191
AGCCTCTCTTGTCCCGGCAGGGTGGACCCATGGGAGTGCTATCAGGACACCTGGCAGACAACCTGCAGTG
TGTTGGAGCACCATCGAGGCCTGATGAAGGTAAGATGGGCTGTGGCTGAGG , 7
AGCCTCTCTTGTCCCGGCAGGGGGGACCCATGGGAGTGCTATCAGGACACCTGGCAGACAACCTGCAGTG
TGTTGGAGCACCATCGAGACCTGATGAAGGTAAGATGGGCTGTGGCTGAGG , 4
AGCCTCTCTTGTCCCGGCAGGGTGGACCCATGGGAGTGCTATCAGGACACCTGGCAGACAACCTGCAGTG
TGTTGGAGCACCATCGGGACCTGATGAAGGTAAGATGGGCTGTGGCTGAGG , 4
AGCCTCTCTTGTCCCGGCAGGGTGGACCCATGGGAGTGCCATCAGGACACCTGGCAGACAACCTGCAGTG
TGTTGGAGCACCATCGAGACCTGATGAAGGTAAGATGGGCTGTGGCTGAGG , 3
AGCCTCTCTTGTCCCGGCAGGGTGGACCCATGGGAGTGCTATCAGGACACCTGGCAGACAACCTGCAGCG
TGTTGGAGCACCATCGAGACCTGATGAAGGTAAGATGGGCTGTGGCTGAGG , 3
AGCCTCTCTTGTCCCGGCAGGGTGGACCCATGGGAGTGCTATCAGGACACCTGGCAGACAACCTGCAGTG
TGTTGGAGCACCATCGAGACCTGGTGAAGGTAAGATGGGCTGTGGCTGAGG , 3

AGCCTCTCTTGTCCCGGCAGGGTGGACCCATGGGAGTGCTATCAGGACACCTGGCAGACAACCTGCAGTG
TGTTGGAGCACCATCGAGACCTGATGGAGGTAAGATGGGCTGTGGCTGAGG , 3
AGCCTCTCTTGTCCCGGCAGGGTGGACCCATGGGAGTGCTATCAGGACACCTGGCAGACAACCTGTAGTG
TGTTGGAGCACCATCGAGACCTGATGAAGGTAAGATGGGCTGTGGCTGAGG , 2
AGCCTCTCTTGTCCCGGCAGGGTGGACCCATGGGAGTGCTATCGGGACACCTGGCAGACAACCTGCAGTG
TGTTGGAGCACCATCGAGACCTGATGAAGGTAAGATGGGCTGTGGCTGAGG , 2
AGCCTCTCTTGTCCCGGCAGGGTGGACCCATGGGAGTGCTATCAGGACACCTGGCAGACAACCTGCAGTG
TGTTGGAGCACCGTCGAGACCTGATGAAGGTAAGATGGGCTGTGGCTGAGG , 2
AGCCTCTCTTGTCCCGGCAGGGTGGACCCATGGGAGTGCTATCAGGACACCTGGCAGACAACTTGCAGTG
TGTTGGAGCACCATCGAGACCTGATGAAGGTAAGATGGGCTGTGGCTGAGG , 2

GEIC-Plate04-C03 TOTAL:2261 OrderedDict([('sp2', 2247), ('T95M', 0), ('T95M only', 1), ('T95M Full ssODN', 0), ('V98M', 0), ('V98M_only', 1), ('V98M Full ssODN', 0), ('Silent Block only', 0), ('Silent Block only Full ssODN', 0)])   [(0, 2258), (-1, 3)]
AGCCTCTCTTGTCCCGGCAGGGTGGACCCATGGGAGTGCTATCAGGACACCTGGCAGACAACCTGCAGTG
TGTTGGAGCACCATCGAGACCTGATGAAGGTAAGATGGGCTGTGGCTGAGG , 2154
AGCCTCTCTTGTCCCGGCAGGGTGGAACCATGGGAGTGCTATCAGGACACCTGGCAGACAACCTGCAGTG
TGTTGGAGCACCATCGAGACCTGATGAAGGTAAGATGGGCTGTGGCTGAGG , 6
AGCCTCTCTTGTCCCGGCAGGGTGGACCCATGGGAGTGCTATCAGGACACCTGGCAGACAACCTGCAGTG
GGTTGGAGCACCATCGAGACCTGATGAAGGTAAGATGGGCTGTGGCTGAGG , 4
AGCCTCTCTTGTCCCGGCAGGGCGGACCCATGGGAGTGCTATCAGGACACCTGGCAGACAACCTGCAGTG
TGTTGGAGCACCATCGAGACCTGATGAAGGTAAGATGGGCTGTGGCTGAGG , 4
AGCCTCTCTTGTCCCGGCAGGGTGGACCCATGGGAGTGCTATCAGGACACCTGGCAGACAACCTGCAGTG
TGTTGGAGCACCATCGAGGCCTGATGAAGGTAAGATGGGCTGTGGCTGAGG , 3
AGCCTCTCTTGTCCCGGCAGGGTGGACCCATGGGAGTGCTATCAGGACACCTGGCAGACAACCTGCAGCG
TGTTGGAGCACCATCGAGACCTGATGAAGGTAAGATGGGCTGTGGCTGAGG , 3
AGCCTCTCTTGTCCCGGCAGGGTGGACCCATGGGAGTGCTATCAGGACACCCGGCAGACAACCTGCAGTG
TGTTGGAGCACCATCGAGACCTGATGAAGGTAAGATGGGCTGTGGCTGAGG , 3
AGCCTCTCTTGTCCCGGCAGGGTGGACCCATGGGAGTGCTATCAGGACACCTGGCAGAAAACCTGCAGTG
TGTTGGAGCACCATCGAGACCTGATGAAGGTAAGATGGGCTGTGGCTGAGG , 2
AGCCTCTCTTGTCCCGGCAGGGTGGACCCATGGGAGTGCTATCAGGACACCTGGCAGACAACCTGCAGTG
TGTTGGAGCACCATCGAGACCTGTTGAAGGTAAGATGGGCTGTGGCTGAGG , 2
AGCCTCTCTTGTCCCGGCAGGGTGGACCCATGGGAGTGCTATCAGGACACCTGGCAGACAACCTGCAGTG
TGTTGGAGCACCATCGAGACCTGATGAAGGTAAGACGGGCTGTGGCTGAGG , 2
AGCCTCTCTTGTCACGGCAGGGTGGACCCATGGGAGTGCTATCAGGACACCTGGCAGACAACCTGCAGTG
TGTTGGAGCACCATCGAGACCTGATGAAGGTAAGATGGGCTGTGGCTGAGG , 2
AGCCTCTCTTGTCCCGGCAGGGTGGACCCATGGGAGTGCTATCAGGACACCTGGCAGACAACCTGCAGTG
TGTTGGAGCACCATCAAGACCTGATGAAGGTAAGATGGGCTGTGGCTGAGG , 2

GEIC-Plate04-C04 TOTAL:1765 OrderedDict([('sp2', 1744), ('T95M', 0), ('T95M only', 0), ('T95M Full ssODN', 0), ('V98M', 3), ('V98M_only', 1), ('V98M Full ssODN', 2), ('Silent Block only', 0), ('Silent Block only Full ssODN', 0)])   [(0, 1763), (-1, 2)]
AGCCTCTCTTGTCCCGGCAGGGTGGACCCATGGGAGTGCTATCAGGACACCTGGCAGACAACCTGCAGTG
TGTTGGAGCACCATCGAGACCTGATGAAGGTAAGATGGGCTGTGGCTGAGG , 1673
AGCCTCTCTTGTCCCGGCAGGGAGGACCCATGGGAGTGCTATCAGGACACCTGGCAGACAACCTGCAGTG
TGTTGGAGCACCATCGAGACCTGATGAAGGTAAGATGGGCTGTGGCTGAGG , 4

AGCCTCTCTTGTCCCGGCAGGGTGGACCCATGGGAGTGCTATCAGGACACCTGGCAGACAACCTGCAGTG
TGTTGGAGCACCATCGAGGCCTGATGAAGGTAAGATGGGCTGTGGCTGAGG , 4
AGCCTCTCTTGTCCCGGCAGGGTGGACCCATGGGAGTGCTATCAGGACACCTGGCAGACAACCTGCAGCG
TGTTGGAGCACCATCGAGACCTGATGAAGGTAAGATGGGCTGTGGCTGAGG , 4
AGCCTCTCTTGTCCCAGCAGGGTGGACCCATGGGAGTGCTATCAGGACACCTGGCAGACAACCTGCAGTG
TGTTGGAGCACCATCGAGACCTGATGAAGGTAAGATGGGCTGTGGCTGAGG , 3
AGCCTCTCTTGTCCCGGCAGGGTGGACCCATGGGAGTGCTATCAGGACACCTGGCAGACAACCTGCAGTG
GGTTGGAGCACCATCGAGACCTGATGAAGGTAAGATGGGCTGTGGCTGAGG , 3
AGCCTCTCTTGTCCCGGCAGGGGGGACCCATGGGAGTGCTATCAGGACACCTGGCAGACAACCTGCAGTG
TGTTGGAGCACCATCGAGACCTGATGAAGGTAAGATGGGCTGTGGCTGAGG , 3
AGCCTCTCTTGTCCCGGCAGGGTGGACCCATGGGAGTGCTATCAGGACACCTGGCAGACAACCTGCAGTG
TGTTGGAGCACCATCGAGACCTGATGAAGGTAAGATGGGTTGTGGCTGAGG , 2
AGCCTCTCTTGTCCCGGCAGGGTGGAACCATGGGAGTGCTATCAGGACACCTGGCAGACAACCTGCAGTG
TGTTGGAGCACCATCGAGACCTGATGAAGGTAAGATGGGCTGTGGCTGAGG , 2
AGCCTCTCTTGTCCCGGCAGGGTGGACCCATGGGAGTGCTATCAGGACACCTGGCAGACAACCTGCAGTG
TGTTGGAGCACCATCGAGTCCTGATGAAGGTAAGATGGGCTGTGGCTGAGG , 2
AGCCTCTCTTGTCCCGGCAGGGTGGACCCATGGGAGTGCTATCAGGACACCCGGCAGACAACCTGCAGTG
TGTTGGAGCACCATCGAGACCTGATGAAGGTAAGATGGGCTGTGGCTGAGG , 2
AGCCTCTCTTGTCCCGGCAGGGTGGACCCATGGGAGGGCTATCAGGACACCTGGCAGACAACCTGCAGTG
TGTTGGAGCACCATCGAGACCTGATGAAGGTAAGATGGGCTGTGGCTGAGG , 2

GEIC-Plate04-C05 TOTAL:2973 OrderedDict([('sp2', 2953), ('T95M', 0), ('T95M only', 0), ('T95M Full ssODN', 0), ('V98M', 2), ('V98M_only', 0), ('V98M Full ssODN', 2), ('Silent Block only', 0), ('Silent Block only Full ssODN', 0)])   [(0, 2971), (-1, 2)]
AGCCTCTCTTGTCCCGGCAGGGTGGACCCATGGGAGTGCTATCAGGACACCTGGCAGACAACCTGCAGTG
TGTTGGAGCACCATCGAGACCTGATGAAGGTAAGATGGGCTGTGGCTGAGG , 2848
AGCCTCTCTTGTCCCGGCAGGGGGGACCCATGGGAGTGCTATCAGGACACCTGGCAGACAACCTGCAGTG
TGTTGGAGCACCATCGAGACCTGATGAAGGTAAGATGGGCTGTGGCTGAGG , 9
AGCCTCTCTTGCCCCGGCAGGGTGGACCCATGGGAGTGCTATCAGGACACCTGGCAGACAACCTGCAGTG
TGTTGGAGCACCATCGAGACCTGATGAAGGTAAGATGGGCTGTGGCTGAGG , 6
AGCCTCTCTTGTCCCGACAGGGTGGACCCATGGGAGTGCTATCAGGACACCTGGCAGACAACCTGCAGTG
TGTTGGAGCACCATCGAGACCTGATGAAGGTAAGATGGGCTGTGGCTGAGG , 4
AGCCTCTCTTGTCCCGGCAGGGTGGACCCATGGGAGTGCTATCAGGACACCTGGCAGACAACCTGCAGTG
GGTTGGAGCACCATCGAGACCTGATGAAGGTAAGATGGGCTGTGGCTGAGG , 3
AGCCTCTCTTGTCCCGGCAGGGTGGACCCATGGGAGTGCTATCAGGAAACCTGGCAGACAACCTGCAGTG
TGTTGGAGCACCATCGAGACCTGATGAAGGTAAGATGGGCTGTGGCTGAGG , 3
AGCCTCTCTTGTCCCGGCAGGGTGGACCCATGGGAGTGCTGTCAGGACACCTGGCAGACAACCTGCAGTG
TGTTGGAGCACCATCGAGACCTGATGAAGGTAAGATGGGCTGTGGCTGAGG , 3
AGCCTCTCTTGTCCCGGCAGGGTGGACCCATGGGAGTGCTATCAGGACACCTGGCAGACAACCTGCAGTG
TGTTGGAGCACCATCGAGGCCTGATGAAGGTAAGATGGGCTGTGGCTGAGG , 2
AGCCTCTCTTGTCCCGGCAGGGTGGACCCATGGGAGTGCTATCAGGACACCTGGCCGACAACCTGCAGTG
TGTTGGAGCACCATCGAGACCTGATGAAGGTAAGATGGGCTGTGGCTGAGG , 2
AGCCTCTCTTGTCCCGGCAGGGTGGACCCATGGGAGTGCTATCAGGACACCTGGCAGACAACCTGCAGTG
TGTTGGAGCACCATCAAGACCTGATGAAGGTAAGATGGGCTGTGGCTGAGG , 2
AGCCTCTCTTGTCCCGGCAGGGTGGACCCATGGGAGTGCTATCAGGACACCTGGCAGACAACCTGCAGTG
TGTTGGAGCGCCATCGAGACCTGATGAAGGTAAGATGGGCTGTGGCTGAGG , 2
AGCCTCTCTTGTCCCGGCAGGGTGGACCCATGGGAGTGCTATCAGGACACCTGGCAGACAACATGCAGTA
TGTTGGAGCACCATCGAGACCTGATGAAGGTAAGATGGGCTGTGGCTGAGG , 2

GEIC-Plate04-C06 TOTAL:1497 OrderedDict([('sp2', 1485), ('T95M', 0), ('T95M only', 0), ('T95M Full ssODN', 0), ('V98M', 0), ('V98M_only', 0), ('V98M Full ssODN', 0), ('Silent Block only', 0), ('Silent Block only Full ssODN', 0)])   [(0, 1494), (-1, 3)]
AGCCTCTCTTGTCCCGGCAGGGTGGACCCATGGGAGTGCTATCAGGACACCTGGCAGACAACCTGCAGTG
TGTTGGAGCACCATCGAGACCTGATGAAGGTAAGATGGGCTGTGGCTGAGG , 1452
AGCCTCTCTTGTCCCGGCAGGGTGGACCCATGGGAGTGCTATCAGGACACCTGGCAGACAACCTTCAGTG
TGTTGGAGCACCATCGAGACCTGATGAAGGTAAGATGGGCTGTGGCTGAGG , 3
AGCCTCTCTTGTCCCGGCAGGGTGGACCCATGGGAGTGCTATCAGGACACCTGGCAGACAACCTGCAGCG
TGTTGGAGCACCATCGAGACCTGATGAAGGTAAGATGGGCTGTGGCTGAGG , 2
AGCCTCTCTTGTCCCGGCAGGGTGGACCCATGGGAGTGCTATCAGGACACCTGGCGGACAACCTGCAGTG
TGTTGGAGCACCATCGAGACCTGATGAAGGTAAGATGGGCTGTGGCTGAGG , 2
AGCCTCTCTTGTCCCGGCAGGGTGGACCCATGGGGGTGCTATCAGGACACCTGGCAGACAACCTGCAGTG
TGTTGGAGCACCATCGAGACCTGATGAAGGTAAGATGGGCTGTGGCTGAGG , 2
AGCCTCTCTTGTCCCGGCAGGGTGGACCCATGGGAGTGCTATCAGGACACCTGGCAGACAACCTGCAGTG
TGTTGGAGCACCATCGAGGCCTGATGAAGGTAAGATGGGCTGTGGCTGAGG , 2
AGCCTCTCTTGTCCCGGCAGGGTGGACCCATGGGAGTGCTATCAGGACACCTGGCAGACAACCTGCAGTG
TGCTGGAGCACCATCGAGACCTGATGAAGGTAAGATGGGCTGTGGCTGAGG , 2
AGCCTCTCTTGTCCCGGCAGGGTGGACCCATGGGAGTGCTATCAGGACACCTGGCAGACGACCTGCAGTG
TGTTGGAGCACCATCGAGACCTGATGAAGGTAAGATGGGCTGTGGCTGAGG , 1
AGCCTCTCTTGTTCCGGCAGGGTGGATCCAAGGGAGTGCTATCAGGACACCTGGCAGACAACCTGCAGCG
CGTTGGAGCACCATCGAGACGTAATGAAGGTAAGATGGGCTGTGGCTGAGG , 1
AGCCTCTCTTGTCCCGGCAGGGTGGACCCATGAGAGTGCTATCAGGACACCTGGCAGACAACCTGCAGTG
TGTTGGAGCACCATCGAGACCTGATGAAGGTAAGATGGGCTGTGGCTGAGG , 1
AGCCTCTCTTGTCCCGGCAGGGTGGACCCATGGGAATGCTATCAGGACACCTGGCAGACAACCTGCAGTG
TGTTGGAGCACCATCGAGACCTGATGAAGGTAAGATGGGCTGTGGCTGAGG , 1
AGCCTCTCTTGTCCCGGCAGGGTGGACCCATGGGAGTGCTATCAGGACACCTGGCAGACAACCTGCAGTG
TGTTGGAGCACCATCGAGACCTGATGAAGATAAGATGGGCTGTGGCTGAGG , 1

GEIC-Plate04-C07 TOTAL:3522 OrderedDict([('sp2', 1717), ('T95M', 1779), ('T95M only', 0), ('T95M Full ssODN', 1720), ('V98M', 3), ('V98M_only', 0), ('V98M Full ssODN', 3), ('Silent Block only', 0), ('Silent Block only Full ssODN', 0)])   [(0, 3521), (-1, 1)]
AGCCTCTCTTGTCCCGGCAGGGTGGACCCATGGGAGTGCTATCAGGACACCTGGCAGACAATGTGCAGTG
TGTTGGAGCACCATCGAGACCTGATGAAGGTAAGATGGGCTGTGGCTGAGG , 1720
AGCCTCTCTTGTCCCGGCAGGGTGGACCCATGGGAGTGCTATCAGGACACCTGGCAGACAACCTGCAGTG
TGTTGGAGCACCATCGAGACCTGATGAAGGTAAGATGGGCTGTGGCTGAGG , 1657
AGCCTCTCTTGTCCCGGCAGGGTGGACCCATGGGAGTGCTATCAGGACACCTGGCAGACAACCTGCAGTG
TGTTGGAGCACCATCGAGGCCTGATGAAGGTAAGATGGGCTGTGGCTGAGG , 5
AGCCTCTCTTGTCCCGGCAGGGAGGACCCATGGGAGTGCTATCAGGACACCTGGCAGACAATGTGCAGTG
TGTTGGAGCACCATCGAGACCTGATGAAGGTAAGATGGGCTGTGGCTGAGG , 4
AGCCTCTCTTGTCCCGGCAGGGTGGACCCATGGGAGTACTATCAGGACACCTGGCAGACAACCTGCAGTG
TGTTGGAGCACCATCGAGACCTGATGAAGGTAAGATGGGCTGTGGCTGAGG , 3
AGCCTCTCTTGTCCCGGCAGGGTGGACCCATGGGAGTGCTATCAGGACACCTGGCAGACAACATGCAGTA
TGTTGGAGCACCATCGAGACCTGATGAAGGTAAGATGGGCTGTGGCTGAGG , 3
AGCCTCTCTTGTCCCGGCAGGGTGGACCCATGGGAGTGCTATCAGGGCACCTGGCAGACAACCTGCAGTG
TGTTGGAGCACCATCGAGACCTGATGAAGGTAAGATGGGCTGTGGCTGAGG , 3

AGCCTCTCTTGTCCCGGCAGGGTGGACCCATGGGAGTGCTATCAGGACACCTGGCAGACAACCTGCAGTG
TGTTGGAGCGCCATCGAGACCTGATGAAGGTAAGATGGGCTGTGGCTGAGG , 3
AGCCTCTCTTGTCCCAGCAGGGTGGACCCATGGGAGTGCTATCAGGACACCTGGCAGACAACCTGCAGTG
TGTTGGAGCACCATCGAGACCTGATGAAGGTAAGATGGGCTGTGGCTGAGG , 3
AGCCTCTCTTGTCCCGGCAGGGTGGACCCATGGGAGTGCTATCAGGACACCTGGCAGACAATGTGCAGCG
TGTTGGAGCACCATCGAGACCTGATGAAGGTAAGATGGGCTGTGGCTGAGG , 3
AGCCTCTCTTGTCCCGGCAGGGTGGACCCATGGGAGTGCTATCAGGACACCTGGCAGACAATGTGCAGTG
TGTTGGAGCACCATAGAGACCTGATGAAGGTAAGATGGGCTGTGGCTGAGG , 3
AGCCTCTCTTGTCCCGGCAGGGTGGACCCATGGGAGTGCTATCAGGACACCTGGCAGACAACGTGCAGTG
TGTTGGAGCACCATCGAGACCTGATGAAGGTAAGATGGGCTGTGGCTGAGG , 2

GEIC-Plate04-C08 TOTAL:1869 OrderedDict([('sp2', 977), ('T95M', 1), ('T95M only', 0), ('T95M Full ssODN', 1), ('V98M', 875), ('V98M_only', 2), ('V98M Full ssODN', 833), ('Silent Block only', 0), ('Silent Block only Full ssODN', 0)])   [(0, 1867), (-1, 2)]
AGCCTCTCTTGTCCCGGCAGGGTGGACCCATGGGAGTGCTATCAGGACACCTGGCAGACAACCTGCAGTG
TGTTGGAGCACCATCGAGACCTGATGAAGGTAAGATGGGCTGTGGCTGAGG , 942
AGCCTCTCTTGTCCCGGCAGGGTGGACCCATGGGAGTGCTATCAGGACACCTGGCAGACAACATGCAGTA
TGTTGGAGCACCATCGAGACCTGATGAAGGTAAGATGGGCTGTGGCTGAGG , 833
AGCCTCTCTTGTCCCGGCAGGGTGGAACCATGGGAGTGCTATCAGGACACCTGGCAGACAACATGCAGTA
TGTTGGAGCACCATCGAGACCTGATGAAGGTAAGATGGGCTGTGGCTGAGG , 4
AGCCTCTCTTGTCCCGGCAGGGTGGACCCATGGGAGTGCTATCAGGACACCTGGCAGACAACATGCAGTG
TGTTGGAGCACCATCGAGACCTGATGAAGGTAAGATGGGCTGTGGCTGAGG , 3
AGCCTCTCTTGTCCCGGCAGGGTGGACCCATGGGAGTGCTATCAGGACACCTGGCAGACAACCTGCAGCG
TGTTGGAGCACCATCGAGACCTGATGAAGGTAAGATGGGCTGTGGCTGAGG , 2
AGCCTCTCTTGTCCCGGCAGGGTGGACCCATGGGAGTGCTATCAGGACACCTGGCAGACAACATGCAGTA
TGTTGGAGCACCATCGAGACCTGATGGAGGTAAGATGGGCTGTGGCTGAGG , 2
AGCCTCTCTTGTCCCGGCAGGGTGGACCCATGGGAGTGCTATCAGGACACCTGGCAGACAACATGCAGTA
TGTTGGAGCACCATCGAGGCCTGATGAAGGTAAGATGGGCTGTGGCTGAGG , 2
AGCCTCTCTTGTCCCGGCAGGGTGGACCCATGGGAGTGCTATCAGGACACCTGGCAGACAACCTGCAGTG
TGTTGGAGCACCATCGAGACCTGATGGAGGTAAGATGGGCTGTGGCTGAGG , 2
AGCCTCTCTTGTCCCGGCAGGGTGGACCCATGGGAGGGCTATCAGGACACCTGGCAGACAACATGCAGTA
TGTTGGAGCACCATCGAGACCTGATGAAGGTAAGATGGGCTGTGGCTGAGG , 2
AGCCTCTCTTGTCCCGGCAGGGTGGACCCATGGGAGTGCTATCAGGACACCTGGCAGACAACCTGCAGTA
TGTTGGAGCACCATCGAGACCTGATGAAGGTAAGATGGGCTGTGGCTGAGG , 2
AGCCTCTCTTGTCCCGGCAGGGAGGACCCATGGGAGTGCTATCAGGACACCTGGCAGACAACATGCAGTA
TGTTGGAGCACCATCGAGACCTGATGAAGGTAAGATGGGCTGTGGCTGAGG , 2
AGCCTCTCTTGTCCCGGCAGGGTGGACCCATGGGAGTGCTATCAGGACACCTGGCAGACAACATGCAGCA
TGTTGGAGCACCATCGAGACCTGATGAAGGTAAGATGGGCTGTGGCTGAGG , 1

GEIC-Plate04-D01 TOTAL:3456 OrderedDict([('sp2', 3438), ('T95M', 0), ('T95M only', 0), ('T95M Full ssODN', 0), ('V98M', 0), ('V98M_only', 0), ('V98M Full ssODN', 0), ('Silent Block only', 0), ('Silent Block only Full ssODN', 0)])   [(0, 3454), (-1, 2)]
AGCCTCTCTTGTCCCGGCAGGGTGGACCCATGGGAGTGCTATCAGGACACCTGGCAGACAACCTGCAGTG
TGTTGGAGCACCATCGAGACCTGATGAAGGTAAGATGGGCTGTGGCTGAGG , 3326
AGCCTCTCTTGTCCCGGCAGGGTGGACCCATGGGAGTGCTATCAGGACACCTGGCAGACAACCTGCAGTG
TGTTGGAGCACCATCGAGACCTGATGAAGGTAGGATGGGCTGTGGCTGAGG , 7

AGCCTCTCTTGTCCCGGCAGGGGGGACCCATGGGAGTGCTATCAGGACACCTGGCAGACAACCTGCAGTG
TGTTGGAGCACCATCGAGACCTGATGAAGGTAAGATGGGCTGTGGCTGAGG , 6
AGCCTCTCTTGTCCCGGCAGGGTGGACCCATGGGAGTGCTATCAGGACACCTGGCAGACAACCTGCAGTG
CGTTGGAGCACCATCGAGACCTGATGAAGGTAAGATGGGCTGTGGCTGAGG , 5
AGCCTCTCTTGTCCCGGCAGGGCGGACCCATGGGAGTGCTATCAGGACACCTGGCAGACAACCTGCAGTG
TGTTGGAGCACCATCGAGACCTGATGAAGGTAAGATGGGCTGTGGCTGAGG , 5
AGCCTCTCTTGTCCCGGCAGGGTGGACCCATGGGAGTGCTATCAGGACACCTGGCAGACAACCTGCAGTG
TGTTGGAGCACCATCGAGGCCTGATGAAGGTAAGATGGGCTGTGGCTGAGG , 3
AGCCTCTCTTGTCCCGGCAGGGAGGACCCATGGGAGTGCTATCAGGACACCTGGCAGACAACCTGCAGTG
TGTTGGAGCACCATCGAGACCTGATGAAGGTAAGATGGGCTGTGGCTGAGG , 3
AGCCTCTCTTGTCCCGGCAGGGTGGACCCATGGGAGTACTATCAGGACACCTGGCAGACAACCTGCAGTG
TGTTGGAGCACCATCGAGACCTGATGAAGGTAAGATGGGCTGTGGCTGAGG , 2
AGCCTCTCTTGTCCCGGCAGGGTGGACCCATGGGAGTGCTATCGGGACACCTGGCAGACAACCTGCAGTG
TGTTGGAGCACCATCGAGACCTGATGAAGGTAAGATGGGCTGTGGCTGAGG , 2
AGCCTCTCTTGTCCCGGCAGGGTGGACCCATGGGAGTGCTATCAGGACACCTGGCAGACAACCTGCAGTG
TGTTGGAGCACCATCGAGACCTGATGAAGGTGAGATGGGCTGTGGCTGAGG , 2
AGCCTCTCTTGTCCCGGCAGGGTGGACCCATGGGAGTGCTATCAGGACACCTGGCAGACAACCTGCAGTG
TGTTGGAGCACCATCGGGACCTGATGAAGGTAAGATGGGCTGTGGCTGAGG , 2
AGCCTCTCTTGTCCCGGCAGGATGGACCCATGGGAGTGCTATCAGGACACCTGGCAGACAACCTGCAGTG
TGTTGGAGCACCATCGAGACCTGATGAAGGTAAGATGGGCTGTGGCTGAGG , 2

GEIC-Plate04-D02 TOTAL:2487 OrderedDict([('sp2', 2469), ('T95M', 0), ('T95M only', 0), ('T95M Full ssODN', 0), ('V98M', 0), ('V98M_only', 1), ('V98M Full ssODN', 0), ('Silent Block only', 0), ('Silent Block only Full ssODN', 0)])   [(0, 2485), (-1, 2)]
AGCCTCTCTTGTCCCGGCAGGGTGGACCCATGGGAGTGCTATCAGGACACCTGGCAGACAACCTGCAGTG
TGTTGGAGCACCATCGAGACCTGATGAAGGTAAGATGGGCTGTGGCTGAGG , 2383
AGCCTCTCTTGTCCCGGCAGGGGGGACCCATGGGAGTGCTATCAGGACACCTGGCAGACAACCTGCAGTG
TGTTGGAGCACCATCGAGACCTGATGAAGGTAAGATGGGCTGTGGCTGAGG , 4
AGCCTCTCTTGTCCCGGCAGGGTGGACCCATGGGAGTGCTATCAGGACACCTGGCAGACAACCTGCAGTG
GGTTGGAGCACCATCGAGACCTGATGAAGGTAAGATGGGCTGTGGCTGAGG , 3
AGCCTCTCTTGTCCCGGCAGGGTGGACCCATGGGAGTGCTATCAGGACACCTGGCAGACAACCTGCAGTG
CGTTGGAGCACCATCGAGACCTGATGAAGGTAAGATGGGCTGTGGCTGAGG , 3
AGCCTCTCTTGTCCCGGCAGGGTGGACCCATGGGAGTGCTATCAGGACACCTGGCAGACAACCTGCAGTG
TGTTGGAGCACCATCGAGGCCTGATGAAGGTAAGATGGGCTGTGGCTGAGG , 2
AGCCTCTCTTGTCCCGGCAGGGTGGACCCATGGGAGTGCTATCAGAACACCTGGCAGACAACCTGCAGTG
TGTTGGAGCACCATCGAGACCTGATGAAGGTAAGATGGGCTGTGGCTGAGG , 2
AGCCTCTCTTGTCCCGGCAGGGAGGACCCATGGGAGTGCTATCAGGACACCTGGCAGACAACCTGCAGTG
TGTTGGAGCACCATCGAGACCTGATGAAGGTAAGATGGGCTGTGGCTGAGG , 2
AGCCTCTCTTGTCCCGGCAGGGTGGACCCATGGGAGTGCTATCAGGACACCTGGCAGACAACCTGCAGTG
TGTTGGAGCATCATCGAGACCTGATGAAGGTAAGATGGGCTGTGGCTGAGG , 2
AGCCTCTCTTGTCCCGGCAGGGTGGACCCATGGGAGTGCTATCAGGACACCTGGCAGACAACCTGCAGTG
TGTTGGAGCACCATCGAGACCTGATGAAGGTAAGATGGGCCGTGGCTGAGG , 2
AGCCTCTCTTGTCCCGGCAGGGTGGACCCATGGGAGTGCTATCAGGACACCTGGCAGACAACCTGCAGTG
TGTTGGAGCACCATCGAGACCCGATGAAGGTAAGATGGGCTGTGGCTGAGG , 2
AGCCTCTCTTGTCCCGGCAGGGTGGACCCATGGGAGTGCTATCAGGACACCTGGCAGACAGCCTGCAGTG
TGTTGGAGCACCATCGAGACCTGATGAAGGTAAGATGGGCTGTGGCTGAGG , 2
AGCCTCTCTTGTCCCGGCAGGGTGGACCCATGGGATTGCTATCAGGACACCTGGCAGACAACCTGCAGTG
TGTTGGAGCACCATCGAGACCTGATGAAGGTAAGATGGGCTGTGGCTGAGG , 2

GEIC-Plate04-D03 TOTAL:2542 OrderedDict([('sp2', 2526), ('T95M', 0), ('T95M only', 1), ('T95M Full ssODN', 0), ('V98M', 3), ('V98M_only', 0), ('V98M Full ssODN', 3), ('Silent Block only', 0), ('Silent Block only Full ssODN', 0)])   [(0, 2541), (-1, 1)]
AGCCTCTCTTGTCCCGGCAGGGTGGACCCATGGGAGTGCTATCAGGACACCTGGCAGACAACCTGCAGTG
TGTTGGAGCACCATCGAGACCTGATGAAGGTAAGATGGGCTGTGGCTGAGG , 2436
AGCCTCTCTTGTCCCGGCAGGGAGGACCCATGGGAGTGCTATCAGGACACCTGGCAGACAACCTGCAGTG
TGTTGGAGCACCATCGAGACCTGATGAAGGTAAGATGGGCTGTGGCTGAGG , 4
AGCCTCTCTTGTCCCGGCAGGGTGGACCCATGGGAGTGCTATCAGGACACCTGGCAGACAACCTGCAGTG
TGTTGGAGCACCATCGAGGCCTGATGAAGGTAAGATGGGCTGTGGCTGAGG , 4
AGCCTCTCTTGTCCCGGCAGGGTGGACCCATGGGAGTGCTATCAGGACACCTGGCAGACAACCTGCAGTG
TGTTGGGGCACCATCGAGACCTGATGAAGGTAAGATGGGCTGTGGCTGAGG , 3
AGCCTCTCTTGTCCCGGCAGGGTGGACCCATGGGAGTGCTATCGGGACACCTGGCAGACAACCTGCAGTG
TGTTGGAGCACCATCGAGACCTGATGAAGGTAAGATGGGCTGTGGCTGAGG , 3
AGCCTCTCTTGTCCCGGCAGGGTGGACCCATGGGAGTGCTATCAGGACACCTGGCAGACAACCTGCAGTG
GGTTGGAGCACCATCGAGACCTGATGAAGGTAAGATGGGCTGTGGCTGAGG , 3
AGCCTCTCTTGTCCCGGCAGGGTGGACCCATGGGAGTGCTATCAGGACACCTGGCAGACAACCTGCAGTG
TGTTGGAGCACCATCGAGACCTGATGAAGGTAAGGTGGGCTGTGGCTGAGG , 3
AGCCTCTCTTGTCCCGACAGGGTGGACCCATGGGAGTGCTATCAGGACACCTGGCAGACAACCTGCAGTG
TGTTGGAGCACCATCGAGACCTGATGAAGGTAAGATGGGCTGTGGCTGAGG , 3
AGCCTCTCTTGTCCCGGCAGGGTGGACCCATGGGAGTGCTATCAGGACACCTGGCAGACAACATGCAGTA
TGTTGGAGCACCATCGAGACCTGATGAAGGTAAGATGGGCTGTGGCTGAGG , 3
AGCCTCTCTTGTCCCGGCAGGGTGGACCCATGGGAGTGCTATCAGGACACCTGGCAGACAACCTGCAGTG
TGTTGGAGAACCATCGAGACCTGATGAAGGTAAGATGGGCTGTGGCTGAGG , 2
AGCCTCTCTTGTCCCGGCAGGGTGGACCCATGGCAGTGCTATCAGGACACCTGGCAGACAACCTGCAGTG
TGTTGGAGCACCATCGAGACCTGATGAAGGTAAGATGGGCTGTGGCTGAGG , 2
AGCCTCTCTTGTCCCGGCAGGGTGGACCCATGGGAGTGCTATCAGGACACCTGGCAGACAACCTGCAGTG
TGTTGGAGCCCCATCGAGACCTGATGAAGGTAAGATGGGCTGTGGCTGAGG , 2

GEIC-Plate04-D04 TOTAL:2217 OrderedDict([('sp2', 2188), ('T95M', 0), ('T95M only', 1), ('T95M Full ssODN', 0), ('V98M', 8), ('V98M_only', 0), ('V98M Full ssODN', 8), ('Silent Block only', 0), ('Silent Block only Full ssODN', 0)])   [(0, 2217)]
AGCCTCTCTTGTCCCGGCAGGGTGGACCCATGGGAGTGCTATCAGGACACCTGGCAGACAACCTGCAGTG
TGTTGGAGCACCATCGAGACCTGATGAAGGTAAGATGGGCTGTGGCTGAGG , 2099
AGCCTCTCTTGTCCCGGCAGGGTGGACCCATGGGAGTGCTATCAGGACACCTGGCAGACAACATGCAGTA
TGTTGGAGCACCATCGAGACCTGATGAAGGTAAGATGGGCTGTGGCTGAGG , 8
AGCCTCTCTTGTCCCGGCAGGGGGGACCCATGGGAGTGCTATCAGGACACCTGGCAGACAACCTGCAGTG
TGTTGGAGCACCATCGAGACCTGATGAAGGTAAGATGGGCTGTGGCTGAGG , 5
AGCCTCTCTTGTCCCGGCAGGGTGGACCCATGGGAGTGCTATCAGGACACCTGGCAGACAACCTGCAGTG
TGTTGGAGCACCATCGAGGCCTGATGAAGGTAAGATGGGCTGTGGCTGAGG , 5
AGCCTCTCTTGTCCCGGCAGGGTGGACCCATGGGAGTGCTATCAGGACACCTGGCAGACAACCTGCAGTG
CGTTGGAGCACCATCGAGACCTGATGAAGGTAAGATGGGCTGTGGCTGAGG , 4
AGCCTCTCTTGTCCCGGCAGGGTGGACCCATGGGAGTGCTATCAGGACACCTGGCAGACAACCTGCAGTG
GGTTGGAGCACCATCGAGACCTGATGAAGGTAAGATGGGCTGTGGCTGAGG , 4
AGCCTCTCTTGTCCCGGCAGGGTGGACCCATGGGAGTGCTATCAGGACACCTGGCAGACAACCTGCAGCG
TGTTGGAGCACCATCGAGACCTGATGAAGGTAAGATGGGCTGTGGCTGAGG , 3

AGCCTCTCTTGTCCCGGCAGGGTGGACCCATGGGAGTGCTATCAGGACACCTGGCAGACAACCTGCAGTG
TGTTGGAGCACCTTCGAGACCTGATGAAGGTAAGATGGGCTGTGGCTGAGG , 3
AGCCTCTCTTGTCCCGGCAGGGTGGACCCATGGGAGTGCTATCAGGGCACCTGGCAGACAACCTGCAGTG
TGTTGGAGCACCATCGAGACCTGATGAAGGTAAGATGGGCTGTGGCTGAGG , 3
AGCCTCTCTTGTCCCGGCAGGGCGGACCCATGGGAGTGCTATCAGGACACCTGGCAGACAACCTGCAGTG
TGTTGGAGCACCATCGAGACCTGATGAAGGTAAGATGGGCTGTGGCTGAGG , 3
AGCCTCTCTTGTCCCGGCAGGGTGGACCCATGGGAGTGCTATCAGGACACCTGGCAGACGACCTGCAGTG
TGTTGGAGCACCATCGAGACCTGATGAAGGTAAGATGGGCTGTGGCTGAGG , 2
AGCCTCTCTTGTCCCGGCAGGGTGTACCCATGGGAGTGCTATCAGGACACCTGGCAGACAACCTGCAGTG
TGTTGGAGCACCATCGAGACCTGATGAAGGTAAGATGGGCTGTGGCTGAGG , 2

GEIC-Plate04-D05 TOTAL:2140 OrderedDict([('sp2', 2113), ('T95M', 0), ('T95M only', 0), ('T95M Full ssODN', 0), ('V98M', 6), ('V98M_only', 1), ('V98M Full ssODN', 5), ('Silent Block only', 0), ('Silent Block only Full ssODN', 0)])   [(0, 2140)]
AGCCTCTCTTGTCCCGGCAGGGTGGACCCATGGGAGTGCTATCAGGACACCTGGCAGACAACCTGCAGTG
TGTTGGAGCACCATCGAGACCTGATGAAGGTAAGATGGGCTGTGGCTGAGG , 2030
AGCCTCTCTTGTCCCGGCAGGGTGGACCCATGGGAGTGCTATCAGGACACCTGGCAGACAACCTGCAGCG
TGTTGGAGCACCATCGAGACCTGATGAAGGTAAGATGGGCTGTGGCTGAGG , 6
AGCCTCTCTTGTCCCGGCAGGGGGGACCCATGGGAGTGCTATCAGGACACCTGGCAGACAACCTGCAGTG
TGTTGGAGCACCATCGAGACCTGATGAAGGTAAGATGGGCTGTGGCTGAGG , 5
AGCCTCTCTTGTCCCGGCAGGGTGGACCCATGGGAGTGCTATCAGGACACCTGGCAGACAACATGCAGTA
TGTTGGAGCACCATCGAGACCTGATGAAGGTAAGATGGGCTGTGGCTGAGG , 5
AGCCTCTCTTGTCCCGGCAGGGTGGACCCATGGGAGTGCTATCAGGGCACCTGGCAGACAACCTGCAGTG
TGTTGGAGCACCATCGAGACCTGATGAAGGTAAGATGGGCTGTGGCTGAGG , 3
AGCCTCTCTTGTCCCGGCAGGGAGGACCCATGGGAGTGCTATCAGGACACCTGGCAGACAACCTGCAGTG
TGTTGGAGCACCATCGAGACCTGATGAAGGTAAGATGGGCTGTGGCTGAGG , 3
AGCCTCTCTTGTCCCGGCAGGGTGGACCCGTGGGAGTGCTATCAGGACACCTGGCAGACAACCTGCAGTG
TGTTGGAGCACCATCGAGACCTGATGAAGGTAAGATGGGCTGTGGCTGAGG , 3
AGCCTCTCTTGTCCCGGCAGGGTGGACCCATGGGAGTGCTATCAGGACACCTGGCAGACAACCTGCAGTG
GGTTGGAGCACCATCGAGACCTGATGAAGGTAAGATGGGCTGTGGCTGAGG , 3
AGCCTCTCTTGTCCCGGCAGGGTGGACCCATGGGAGTGCTATCAGGACACCTGGCAGGCAACCTGCAGTG
TGTTGGAGCACCATCGAGACCTGATGAAGGTAAGATGGGCTGTGGCTGAGG , 3
AGCCTCTCTTGTCCTGGCAGGGTGGACCCATGGGAGTGCTATCAGGACACCTGGCAGACAACCTGCAGTG
TGTTGGAGCACCATCGAGACCTGATGAAGGTAAGATGGGCTGTGGCTGAGG , 3
AGCCTCTCTTGTCCCGGCAGGGTGGACCCATGGGAGTGCTATCAGGACACCTGGCAGACGACCTGCAGTG
TGTTGGAGCACCATCGAGACCTGATGAAGGTAAGATGGGCTGTGGCTGAGG , 2
AGCCTCTCTTGTCCCGGCAGGGTGGACCCATGGGAGTGCTATCAGGACACCTGGCAGACAACCTGCAGTG
TGTTGGAGCACCATCGGGACCTGATGAAGGTAAGATGGGCTGTGGCTGAGG , 2

GEIC-Plate04-D06 TOTAL:3292 OrderedDict([('sp2', 3269), ('T95M', 0), ('T95M only', 0), ('T95M Full ssODN', 0), ('V98M', 3), ('V98M_only', 0), ('V98M Full ssODN', 3), ('Silent Block only', 0), ('Silent Block only Full ssODN', 0)])   [(0, 3290), (-1, 2)]
AGCCTCTCTTGTCCCGGCAGGGTGGACCCATGGGAGTGCTATCAGGACACCTGGCAGACAACCTGCAGTG
TGTTGGAGCACCATCGAGACCTGATGAAGGTAAGATGGGCTGTGGCTGAGG , 3167
AGCCTCTCTTGTCCCGGCAGGGGGGACCCATGGGAGTGCTATCAGGACACCTGGCAGACAACCTGCAGTG
TGTTGGAGCACCATCGAGACCTGATGAAGGTAAGATGGGCTGTGGCTGAGG , 9

AGCCTCTCTTGTCCCGGCAGGGTGGGCCCATGGGAGTGCTATCAGGACACCTGGCAGACAACCTGCAGTG
TGTTGGAGCACCATCGAGACCTGATGAAGGTAAGATGGGCTGTGGCTGAGG , 4
AGCCTCTCTTGTCCCGGCAGGGTGGACCCATGGGAGTGCTATCAGGACACCTGGCAGACAACCTGCAGTG
GGTTGGAGCACCATCGAGACCTGATGAAGGTAAGATGGGCTGTGGCTGAGG , 4
AGCCTCTCTTGTCCCGGCAGGGTGGACCCATGGGAGTGCTATCAGGACACCTGGTAGACAACCTGCAGTG
TGTTGGAGCACCATCGAGACCTGATGAAGGTAAGATGGGCTGTGGCTGAGG , 3
AGCCTCTCTTGTCCCGGCAGGGTGGACCCATGGGAGTGCTATCAGGACACCTGGCAGACAACATGCAGTA
TGTTGGAGCACCATCGAGACCTGATGAAGGTAAGATGGGCTGTGGCTGAGG , 3
AGCCTCTCTTGTCCCGGCAGGGTGGACCCATGGGAGTGCTATCAGGACACCTGGCAGACAACCTGCAGTG
TGTTGGAGCACCATCGAGGCCTGATGAAGGTAAGATGGGCTGTGGCTGAGG , 3
AGCCTCTCTTGTCCCGGCAGGGTGGACCCATGGGAGTGCTATCAGGACACCTGGCAGACAACCTGCAGTG
TGTTGGAGCACCATCGAGACCTGATGAAGGTAAGATGGGCGGTGGCTGAGG , 3
AGCCTCTCTTGTCCCGGCAGGGTGGACCCATGGGAGTGCTATCAGGACACCTGGCAGACAACCTGCAGTG
CGTTGGAGCACCATCGAGACCTGATGAAGGTAAGATGGGCTGTGGCTGAGG , 3
AGCCTCTCTTGTCCCGGCAGGGTGGACCCATGGGAGGGCTATCAGGACACCTGGCAGACAACCTGCAGTG
TGTTGGAGCACCATCGAGACCTGATGAAGGTAAGATGGGCTGTGGCTGAGG , 3
AGCCTCTCTTGTCCCGGCAGGGTGGACCCATGGGAGTGCTATCAGGACACCCGGCAGACAACCTGCAGTG
TGTTGGAGCACCATCGAGACCTGATGAAGGTAAGATGGGCTGTGGCTGAGG , 3
AGCCTCTCTTGTGCCGGCAGGGTGGACCCATGGGAGTGCTATCAGGACACCTGGCAGACAACCTGCAGTG
TGTTGGAGCACCATCGAGACCTGATGAAGGTAAGATGGGCTGTGGCTGAGG , 3

GEIC-Plate04-D07 TOTAL:3915 OrderedDict([('sp2', 1927), ('T95M', 0),
('T95M only', 0), ('T95M Full ssODN', 0), ('V98M', 1951),
('V98M_only', 4), ('V98M Full ssODN', 1887), ('Silent Block only', 0),
('Silent Block only Full ssODN', 0)])   [(0, 3913), (-1, 2)]
AGCCTCTCTTGTCCCGGCAGGGTGGACCCATGGGAGTGCTATCAGGACACCTGGCAGACAACATGCAGTA
TGTTGGAGCACCATCGAGACCTGATGAAGGTAAGATGGGCTGTGGCTGAGG , 1887
AGCCTCTCTTGTCCCGGCAGGGTGGACCCATGGGAGTGCTATCAGGACACCTGGCAGACAACCTGCAGTG
TGTTGGAGCACCATCGAGACCTGATGAAGGTAAGATGGGCTGTGGCTGAGG , 1863
AGCCTCTCTTGTCCCGGCAGGGGGGACCCATGGGAGTGCTATCAGGACACCTGGCAGACAACCTGCAGTG
TGTTGGAGCACCATCGAGACCTGATGAAGGTAAGATGGGCTGTGGCTGAGG , 6
AGCCTCTCTTGTCCCGGCAGGGTGGACCCATGGGAGTGCTATCAGGACACCTGGCAGACAACATGCAGTG
TGTTGGAGCACCATCGAGACCTGATGAAGGTAAGATGGGCTGTGGCTGAGG , 5
AGCCTCTCTTGTCCCGGCAGGGTGGACCCATGGGAGTGCTATCAGGACACCTGGCAGACAACCTGCAGTA
TGTTGGAGCACCATCGAGACCTGATGAAGGTAAGATGGGCTGTGGCTGAGG , 4
AGCCTCTCTTGTCCCGGCAGGGCGGACCCATGGGAGTGCTATCAGGACACCTGGCAGACAACATGCAGTA
TGTTGGAGCACCATCGAGACCTGATGAAGGTAAGATGGGCTGTGGCTGAGG , 3
AGCCTCTCTTGTCCCGGCAGGGTGGACCCATGGGAGTGCTATCAGGACACCTGGGAGACAACATGCAGTA
TGTTGGAGCACCATCGAGACCTGATGAAGGTAAGATGGGCTGTGGCTGAGG , 3
AGCCTCTCTTGTCCCGGCAGGGGGGACCCATGGGAGTGCTATCAGGACACCTGGCAGACAACATGCAGTA
TGTTGGAGCACCATCGAGACCTGATGAAGGTAAGATGGGCTGTGGCTGAGG , 3
AGCCTCTCTTGTCCCGGCAGGGTGGACCCATGGGAGTGCTATCAGGACACCTGGCAGACAACCTTCAGTG
TGTTGGAGCACCATCGAGACCTGATGAAGGTAAGATGGGCTGTGGCTGAGG , 2
AGCCTCTCTTGTCCCGGCAGGGTGGACCCATGGGAGTGCTATCAGGACACCTGGCAGACAACCTGCAGTG
TGTTGGAGCACCATCGAGACCTGATGAAGGTAAGATGGACTGTGGCTGAGG , 2
AGCCTCTCTTGTCCCGGCAGGGTGGACCCATGGGAGTGCTATCAGGACACCTGGCAGACAACCTGCAGTG
TGTTGGAGCACCATCGAGACCTGATGATGGTAAGATGGGCTGTGGCTGAGG , 2
AGCCTCTCTTGTCCCGGCAGGGTGGACCCATGGGAGTGCTATCAGGACACCTGGCAGACAACCTGCAGCG
TGTTGGAGCACCATCGAGACCTGATGAAGGTAAGATGGGCTGTGGCTGAGG , 2

GEIC-Plate04-D08 TOTAL:1985 OrderedDict([('sp2', 1052), ('T95M', 918), ('T95M only', 1), ('T95M Full ssODN', 889), ('V98M', 0), ('V98M_only', 0), ('V98M Full ssODN', 0), ('Silent Block only', 0), ('Silent Block only Full ssODN', 0)])   [(0, 1985)]
AGCCTCTCTTGTCCCGGCAGGGTGGACCCATGGGAGTGCTATCAGGACACCTGGCAGACAACCTGCAGTG
TGTTGGAGCACCATCGAGACCTGATGAAGGTAAGATGGGCTGTGGCTGAGG , 1013
AGCCTCTCTTGTCCCGGCAGGGTGGACCCATGGGAGTGCTATCAGGACACCTGGCAGACAATGTGCAGTG
TGTTGGAGCACCATCGAGACCTGATGAAGGTAAGATGGGCTGTGGCTGAGG , 889
AGCCTCTCTTGTCCCGGCAGGGTGGACCCATGGGAGTGCTATCAGGACACCTGGCAGACAATGTGCAGTG
CGTTGGAGCACCATCGAGACCTGATGAAGGTAAGATGGGCTGTGGCTGAGG , 2
AGCCTCTCTTGTCCCGGCAGGGTGGACCCATGGGAGTGCTATCAGGACACCTGGCAGACAACCTGCAGTG
TGTTGGAGCACCATCGAGACCTGACGAAGGTAAGATGGGCTGTGGCTGAGG , 2
AGCCTCTCTTGTCCCGGCAGGGTGGACCCATGGGAGTGCTATCAGGACACCTGGCAGACAACCTGCAGTG
TGTTGGAGCACCATCGAGACCTGATGAAGGTAAGATGGGCGGTGGCTGAGG , 2
AGCCTCTCTTGTCCCGGCAGGGTGGACCCATGGGAGTGCTATCAGGACACCTGGCAGACAATGTGCAGCG
TGTTGGAGCACCATCGAGACCTGATGAAGGTAAGATGGGCTGTGGCTGAGG , 2
AGCCTCTCTTGTCCCGGCAGGGTGGACCCATGGGAGTGCTATCAGGACACCTGGCAGACAACCTGCAGTG
TGTTGGAGCACCATCGAGACCTGATGAAGGTAGGATGGGCTGTGGCTGAGG , 2
AGCCTCTCTTGTCCCGGCAGGGTGGACCCATGGGAGTGCTATCAGGACACCTGGCAGACAATGTGCAGTG
GGTTGGAGCACCATCGAGACCTGATGAAGGTAAGATGGGCTGTGGCTGAGG , 2
AGCCTCTCTTGTCCCGGCAGGGTGGACCCATGGGAGTGCTATCAGGACACCAGGCAGACAATGTGCAGTG
TGTTGGAGCACCATCGAGACCTGATGAAGGTAAGATGGGCTGTGGCTGAGG , 1
AGCCTCTCTTGTCCCGGCAGGGTGGACCCATGAGAGTGCTATCAGGACACCTGGCAGACAATGTGCAGTG
TGTTGGAGCACCATCGAGACCTGATGAAGGTAAGATGGGCTGTGGCTGAGG , 1
AGCCTCTCTTGTCCCGGCAGGGTGGACCCATGGGCGTGCTATCAGGACACCTGGCAGACAATGTGCAGTG
TGTTGGAGCACCATCGAGACCTGATGAAGGTAAGATGGGCTGTGGCTGAGG , 1
AGCCTCTCTTGTCCCGGCAGGGTGGACCCATGGGAGTGCTATCAGGACACCTGGCAGACAACGTGCAGTG
TGTTGGAGCACCATCGAGACCTGATGAAGGTAAGATGGGCTGTGGCTGAGG , 1

GEIC-Plate04-E01 TOTAL:2213 OrderedDict([('sp2', 2201), ('T95M', 0), ('T95M only', 1), ('T95M Full ssODN', 0), ('V98M', 0), ('V98M_only', 1), ('V98M Full ssODN', 0), ('Silent Block only', 0), ('Silent Block only Full ssODN', 0)])   [(0, 2213)]
AGCCTCTCTTGTCCCGGCAGGGTGGACCCATGGGAGTGCTATCAGGACACCTGGCAGACAACCTGCAGTG
TGTTGGAGCACCATCGAGACCTGATGAAGGTAAGATGGGCTGTGGCTGAGG , 2127
AGCCTCTCTTGTCCCGGCAGGGTGGACCCATGGGAGTGCTATCAGGACACCTGGCAGACAACCTGCAGTG
TGTTGGAGCACCATCGAGACCTGATGAAGGTAAGATGGGCTTTGGCTGAGG , 3
AGCCTCTCTTGTCCCGGCAGGGTGGACCCATGGGAGTGCTATCAGGACACCTGGCAGACAACCTGCAGTG
TGTTGGAGCACCATCGAGGCCTGATGAAGGTAAGATGGGCTGTGGCTGAGG , 3
AGCCTCTCTTGTCCCGGCAGGGTGGACCCATGGGAGTGCTATCAGGACACCTGGCAGACAACCTGCAGTG
TGTTGGAGCACCATCGAGACCTGATGGAGGTAAGATGGGCTGTGGCTGAGG , 2
AGCCTCTCTTGTCCCGGCAGGGTGGACCCATGGGAGTGCTATCAGGACACCTGGCAGACAACCCGCAGTG
TGTTGGAGCACCATCGAGACCTGATGAAGGTAAGATGGGCTGTGGCTGAGG , 2
AGCCTCTCTTGTCCCGGCAGGGTGGACCCATGGGAGTGCTATCAGGACACCTGGCAGACAGCCTGCAGTG
TGTTGGAGCACCATCGAGACCTGATGAAGGTAAGATGGGCTGTGGCTGAGG , 2
AGCCTCTCTTGTCCCGGCAGGGTGGACCCATGGGAGTGCTATCAGGACACCTGGCGGACAACCTGCAGTG
TGTTGGAGCACCATCGAGACCTGATGAAGGTAAGATGGGCTGTGGCTGAGG , 2

AGCCTCTCTTGTCCCGGCAGGGTGGACCCATGGGAGTGCTATCAGGACACCTGGCAGACAACCTGCAGTG
TGTTGGAGCACCATCGGGACCTGATGAAGGTAAGATGGGCTGTGGCTGAGG , 2
AGCCTCTCTTGTCCCGGCAGGGTGGACCCATGGGAGTGCTATCAGGACACCTGGCAGACAACCTGCAGTG
TGTTGGAGCACCATCGAGACCTGATGAAGGTAAGATGGGCTATGGCTGAGG , 2
AGCCTCTCTTGTCCCGGCAGGGTGGACCCATGGGAGTGCTATCAGGGCACCTGGCAGACAACCTGCAGTG
TGTTGGAGCACCATCGAGACCTGATGAAGGTAAGATGGGCTGTGGCTGAGG , 2
AGCCTCTCTTATCCCGGCAGGGTGGACCCATGGGAGTGCTATCAGGACACCTGGCAGACAACCTGCAGTG
TGTTGGAGCACCATCGAGACCTGATGAAGGTAAGATGGGCTGTGGCTGAGG , 2
AGCCTCTCTTGTCCCGGCAGGGTGGACCCATGGGAGTACTATCAGGACACCTGGCAGACAACCTGCAGTG
TGTTGGAGCACCATCGAGACCTGATGAAGGTAAGATGGGCTGTGGCTGAGG , 2

GEIC-Plate04-E02 TOTAL:2119 OrderedDict([('sp2', 2107), ('T95M', 0), ('T95M only', 0), ('T95M Full ssODN', 0), ('V98M', 4), ('V98M_only', 0), ('V98M Full ssODN', 3), ('Silent Block only', 0), ('Silent Block only Full ssODN', 0)])   [(0, 2118), (-1, 1)]
AGCCTCTCTTGTCCCGGCAGGGTGGACCCATGGGAGTGCTATCAGGACACCTGGCAGACAACCTGCAGTG
TGTTGGAGCACCATCGAGACCTGATGAAGGTAAGATGGGCTGTGGCTGAGG , 2000
AGCCTCTCTTGTCCCGGCAGGGTGGACCCATGGGAGTGCTATCAGGACACCTGGCAGACAACCTGCAGTG
TGTTGGAGCACCATCGAGACCTGATGGAGGTAAGATGGGCTGTGGCTGAGG , 5
AGCCTCTCTTGTCCCGGCAGGGGGGACCCATGGGAGTGCTATCAGGACACCTGGCAGACAACCTGCAGTG
TGTTGGAGCACCATCGAGACCTGATGAAGGTAAGATGGGCTGTGGCTGAGG , 4
AGCCTCTCTTGTCCCGGCAGGGTGGACCCATGGGAGTGCTATCAGGACACCTGGCAGACAACCTGCAGTG
TGTTGGAGCGCCATCGAGACCTGATGAAGGTAAGATGGGCTGTGGCTGAGG , 4
AGCCTCTCTTGTCCCGGCAGGGTGGACCCATGGGAGTGCTATCAGGACACCTGGCAGACAACATGCAGTA
TGTTGGAGCACCATCGAGACCTGATGAAGGTAAGATGGGCTGTGGCTGAGG , 3
AGCCTCTCTTGTCCCGGCAGGGTGGACCCATGGGAGGGCTATCAGGACACCTGGCAGACAACCTGCAGTG
TGTTGGAGCACCATCGAGACCTGATGAAGGTAAGATGGGCTGTGGCTGAGG , 3
AGCCTCTCTTGTCCCGGCAGGGTGGACCCATGGGAGTGCTATCAGGACACCTGGCAGACAACGTGCAGTG
TGTTGGAGCACCATCGAGACCTGATGAAGGTAAGATGGGCTGTGGCTGAGG , 2
AGCCTCTCTTGTCCCGGCAGGGTGGACCCATGGGAGTGCTATCAGGACACCTGGCGGACAACCTGCAGTG
TGTTGGAGCACCATCGAGACCTGATGAAGGTAAGATGGGCTGTGGCTGAGG , 2
AGCCTCTCTTGTCCCGGCAGGGTGGACCCATGGGAGTGCTATCAGGACTCCTGGCAGACAACCTGCAGTG
TGTTGGAGCACCATCGAGACCTGATGAAGGTAAGATGGGCTGTGGCTGAGG , 2
AGCCTCTCTTGTCCCGGCAGGGTGGACCCATGGGAGTGCTATCAGGGCACCTGGCAGACAACCTGCAGTG
TGTTGGAGCACCATCGAGACCTGATGAAGGTAAGATGGGCTGTGGCTGAGG , 2
AGCCTCTCTTGTCCCGGCAGGGTGGACCCATGGGAGTGCTATCAGGACACCTGGCAGACAACCTGCAGTG
TGTTGGAGCACCATCGAGGCCTGATGAAGGTAAGATGGGCTGTGGCTGAGG , 2
AGCCTCTCTTGTCCCGGCAGGGTGGACCCATGGGAGTGCTATCAGGACACCTGGCAGACAACCTGCAGTG
TGCTGGAGCACCATCGAGACCTGATGAAGGTAAGATGGGCTGTGGCTGAGG , 2

GEIC-Plate04-E03 TOTAL:2693 OrderedDict([('sp2', 2672), ('T95M', 0), ('T95M only', 0), ('T95M Full ssODN', 0), ('V98M', 1), ('V98M_only', 1), ('V98M Full ssODN', 1), ('Silent Block only', 0), ('Silent Block only Full ssODN', 0)])   [(0, 2689), (-1, 4)]
AGCCTCTCTTGTCCCGGCAGGGTGGACCCATGGGAGTGCTATCAGGACACCTGGCAGACAACCTGCAGTG
TGTTGGAGCACCATCGAGACCTGATGAAGGTAAGATGGGCTGTGGCTGAGG , 2563
AGCCTCTCTTGTCCCGGCAGGGAGGACCCATGGGAGTGCTATCAGGACACCTGGCAGACAACCTGCAGTG
TGTTGGAGCACCATCGAGACCTGATGAAGGTAAGATGGGCTGTGGCTGAGG , 5

AGCCTCTCTTGTCCCGGCAGGGGGGACCCATGGGAGTGCTATCAGGACACCTGGCAGACAACCTGCAGTG
TGTTGGAGCACCATCGAGACCTGATGAAGGTAAGATGGGCTGTGGCTGAGG , 4
AGCCTCTCTTGTCCCGGCAGGGTGGACCCATGGGAGTGCTATCAGGACACCTGGCAGACAACCTGCAGTG
CGTTGGAGCACCATCGAGACCTGATGAAGGTAAGATGGGCTGTGGCTGAGG , 4
AGCCTCTCTTGTCCCGGCAGGGTGGACCCATGGGAGGGCTATCAGGACACCTGGCAGACAACCTGCAGTG
TGTTGGAGCACCATCGAGACCTGATGAAGGTAAGATGGGCTGTGGCTGAGG , 4
AGCCTCTCTTGTCCCGGCGGGGTGGACCCATGGGAGTGCTATCAGGACACCTGGCAGACAACCTGCAGTG
TGTTGGAGCACCATCGAGACCTGATGAAGGTAAGATGGGCTGTGGCTGAGG , 3
AGCCTCTCTTGTCCCGGCAGGGTGGACACATGGGAGTGCTATCAGGACACCTGGCAGACAACCTGCAGTG
TGTTGGAGCACCATCGAGACCTGATGAAGGTAAGATGGGCTGTGGCTGAGG , 3
AGCCTCTCTTGTCCCGGCAGGGCGGACCCATGGGAGTGCTATCAGGACACCTGGCAGACAACCTGCAGTG
TGTTGGAGCACCATCGAGACCTGATGAAGGTAAGATGGGCTGTGGCTGAGG , 3
AGCCTCTCTTGTCCCGGCAGGGTGGACCCATGGGAGTGCTATCAGGACACCTGGCAGACAACGTGCAGTG
TGTTGGAGCACCATCGAGACCTGATGAAGGTAAGATGGGCTGTGGCTGAGG , 2
AGCCTCTCTTGTCCCGGCAGGGTGGACCCATGGGAGTGCTATCAGGACACCTGGCAGACAACCTGCAGTG
TGTTGGAGCACCATCGAGACCTGATGCAGGTAAGATGGGCTGTGGCTGAGG , 2
AGCCTCTCTTGTCCCGGCAGGGTGGACCCATGGGAGTGCTATCAGGACACCTGGCAGACAACCTGCAGTG
TGTTGGAGCACCATCGAGACCTGATGATGGTAAGATGGGCTGTGGCTGAGG , 2
AGCCTCTCTTGTCCCGGCAAGGTGGACCCATGGGAGTGCTATCAGGACACCTGGCAGACAACCTGCAGTG
TGTTGGAGCACCATCGAGACCTGATGAAGGTAAGATGGGCTGTGGCTGAGG , 2

GEIC-Plate04-E04 TOTAL:3283 OrderedDict([('sp2', 3265), ('T95M', 0), ('T95M only', 0), ('T95M Full ssODN', 0), ('V98M', 0), ('V98M_only', 0), ('V98M Full ssODN', 0), ('Silent Block only', 0), ('Silent Block only Full ssODN', 0)]) [(0, 3282), (-1, 1)]
AGCCTCTCTTGTCCCGGCAGGGTGGACCCATGGGAGTGCTATCAGGACACCTGGCAGACAACCTGCAGTG
TGTTGGAGCACCATCGAGACCTGATGAAGGTAAGATGGGCTGTGGCTGAGG , 3175
AGCCTCTCTTGTCCCGGCAGGGTGGACCCATGGGAGTGCTATCAGGACACCTGGCAGACAACCTGCAGTG
TGTTGGAGCACCATCGAGGCCTGATGAAGGTAAGATGGGCTGTGGCTGAGG , 5
AGCCTCTCTTGTCCCGGCAGGGCGGACCCATGGGAGTGCTATCAGGACACCTGGCAGACAACCTGCAGTG
TGTTGGAGCACCATCGAGACCTGATGAAGGTAAGATGGGCTGTGGCTGAGG , 5
AGCCTCTCTTGTCCCGGCAGGGTGGACCCATGGGAGTGCTATCAGGACACCTGGCAGACAACCTGCAGTG
GGTTGGAGCACCATCGAGACCTGATGAAGGTAAGATGGGCTGTGGCTGAGG , 4
AGCCTCTCTTGTCCCGGCAGGGTGGACCCATGGGAGTGCTATCAGGACACCTGGCAGACAACCTGCAGTG
TGTTGGAGAACCATCGAGACCTGATGAAGGTAAGATGGGCTGTGGCTGAGG , 3
AGCCTCTCTTGTCCCGGCAGGGAGGACCCATGGGAGTGCTATCAGGACACCTGGCAGACAACCTGCAGTG
TGTTGGAGCACCATCGAGACCTGATGAAGGTAAGATGGGCTGTGGCTGAGG , 3
AGCCTCTCTTGTCCCGGCAGGGTGGACCCATGGGAGTGCTATCAGGACACCTGGCAGACAACCTGCGGTG
TGTTGGAGCACCATCGAGACCTGATGAAGGTAAGATGGGCTGTGGCTGAGG , 3
AGCCTCTCTTGTCCCGGCAGGGTGGACCCATGGGAGTGCTATCAGGACACCTGGCAGACAGCCTGCAGTG
TGTTGGAGCACCATCGAGACCTGATGAAGGTAAGATGGGCTGTGGCTGAGG , 2
AGCCTCTCTTGTCCCGGCAGGGTGGACCCATGGGAGTGCTATCAGGACACCTGGCAGACAACCTGCAGTG
TGTTGGAGCACCATCGAGACCTGATGAAGGTAAGATGGGCGGTGGCTGAGG , 2
AGCCTCTCTTGTCCCGGCAGGGTGGACCCATGGGAGTGCTATCAGGACACCTGGCAGACAACCTGCAGTG
TGTTGGAGCACCATCGAGACCTGATGATGGTAAGATGGGCTGTGGCTGAGG , 2
AGCCTCTCTTGTCCCGGCAGGGTGGCCCCATGGGAGTGCTATCAGGACCCCTGGCAGACAACCTGCAGTG
TGTTGGAGCACCATCGAGACCTGATGAAGGTAAGATGGGCTGTGGCTGAGG , 2
AGCCTCTCTTGTCCCGGCAGGGTGGACCCATGGGAGTGCTATCAGGACACCTGGCAGACAACCCGCAGTG
TGTTGGAGCACCATCGAGACCTGATGAAGGTAAGATGGGCTGTGGCTGAGG , 2

GEIC-Plate04-E05 TOTAL:3250 OrderedDict([('sp2', 3230), ('T95M', 0), ('T95M only', 0), ('T95M Full ssODN', 0), ('V98M', 0), ('V98M_only', 0), ('V98M Full ssODN', 0), ('Silent Block only', 0), ('Silent Block only Full ssODN', 0)])   [(0, 3246), (-1, 4)]
AGCCTCTCTTGTCCCGGCAGGGTGGACCCATGGGAGTGCTATCAGGACACCTGGCAGACAACCTGCAGTG
TGTTGGAGCACCATCGAGACCTGATGAAGGTAAGATGGGCTGTGGCTGAGG , 3122
AGCCTCTCTTGTCCCGGCAGGGTGGACCCATGGGAGTGCTATCAGGACACCTGGCAGACAACCTGCAGTG
TGTTGGAGCACCATCGGGACCTGATGAAGGTAAGATGGGCTGTGGCTGAGG , 4
AGCCTCTCTTGTCCCGGCAGGGTGGACCCATGGGAGTGCTATCAGGACACCTGGCAGACAACCTGCAGCG
TGTTGGAGCACCATCGAGACCTGATGAAGGTAAGATGGGCTGTGGCTGAGG , 4
AGCCTCTCTTGTCCCGGCAGGGTGGACCCATGGGAGCGCTATCAGGACACCTGGCAGACAACCTGCAGTG
TGTTGGAGCACCATCGAGACCTGATGAAGGTAAGATGGGCTGTGGCTGAGG , 4
AGCCTCTCTTGTCCCGGCAGGGTGGACCCATGGGAGGGCTATCAGGACACCTGGCAGACAACCTGCAGTG
TGTTGGAGCACCATCGAGACCTGATGAAGGTAAGATGGGCTGTGGCTGAGG , 4
AGCCTCTCTTGTCCCGGCAGGGGGGACCCATGGGAGTGCTATCAGGACACCTGGCAGACAACCTGCAGTG
TGTTGGAGCACCATCGAGACCTGATGAAGGTAAGATGGGCTGTGGCTGAGG , 3
AGCCTCTCTTGTCCCGGCAGGGTGGACCCATGGGAGTGCTATCAGGACACCTGGCAGACAACCTGCAGTG
TGTTGGAGCGCCATCGAGACCTGATGAAGGTAAGATGGGCTGTGGCTGAGG , 3
AGCCTCTCTTGTCCCGGCAGGGTGGACCCATGGGAGTGCTATCAGGACACCTGGCAGGCAACCTGCAGTG
TGTTGGAGCACCATCGAGACCTGATGAAGGTAAGATGGGCTGTGGCTGAGG , 3
AGCCTCTCTTGTCCCGGCAGGGTGGACCCATGGGAGTGCTATCAGGACACCTGGCAGACAACCTGCAGTG
TGTTGGAGCACCATCGAGACCTGATGGAGGTAAGATGGGCTGTGGCTGAGG , 3
AGCCTCTCTTGTCCCGGCAGGGTGGACCCGTGGGAGTGCTATCAGGACACCTGGCAGACAACCTGCAGTG
TGTTGGAGCACCATCGAGACCTGATGAAGGTAAGATGGGCTGTGGCTGAGG , 3
AGCCTCTCTTGTCCCGGCAGGGTGGACCCATGGGAGTGCTATCAGGACACCTGGCAGACAACCTGCAGTG
TGTTGGAGCACCATCGAGGCCTGATGAAGGTAAGATGGGCTGTGGCTGAGG , 2
AGCCTCTCTTGTCCCGGCAGGGTGGACCCATGGGAGTGCTATCAGGACACCTGGCAGACAACCTGCAGTG
TGTTGGAGCACCATCGAGACCTGATGAGGGTAAGATGGGCTGTGGCTGAGG , 2

GEIC-Plate04-E06 TOTAL:3580 OrderedDict([('sp2', 3558), ('T95M', 0), ('T95M only', 0), ('T95M Full ssODN', 0), ('V98M', 0), ('V98M_only', 0), ('V98M Full ssODN', 0), ('Silent Block only', 0), ('Silent Block only Full ssODN', 0)])   [(0, 3578), (-1, 2)]
AGCCTCTCTTGTCCCGGCAGGGTGGACCCATGGGAGTGCTATCAGGACACCTGGCAGACAACCTGCAGTG
TGTTGGAGCACCATCGAGACCTGATGAAGGTAAGATGGGCTGTGGCTGAGG , 3430
AGCCTCTCTTGTCCCGGCAGGGGGGACCCATGGGAGTGCTATCAGGACACCTGGCAGACAACCTGCAGTG
TGTTGGAGCACCATCGAGACCTGATGAAGGTAAGATGGGCTGTGGCTGAGG , 7
AGCCTCTCTTGTCCCGGCAGGGTGGACCCATGGGAGTGCTATCAGGACACCTGGCAGACAACCTGCAGTG
TGTTGGAGCACCATCGAGGCCTGATGAAGGTAAGATGGGCTGTGGCTGAGG , 5
AGCCTCTCTTGTCCCGGCAGGGTGGACCCATGGGAGTGCTATCGGGACACCTGGCAGACAACCTGCAGTG
TGTTGGAGCACCATCGAGACCTGATGAAGGTAAGATGGGCTGTGGCTGAGG , 4
AGCCTCTCTTGTCCCGGCAGGGTGGACCCATGGGAGTGCTATCAGGACACCTGGCAGACAACCTGCAGTG
GGTTGGAGCACCATCGAGACCTGATGAAGGTAAGATGGGCTGTGGCTGAGG , 4
AGCCTCTCTTGTCCCGGCAGGGCGGACCCATGGGAGTGCTATCAGGACACCTGGCAGACAACCTGCAGTG
TGTTGGAGCACCATCGAGACCTGATGAAGGTAAGATGGGCTGTGGCTGAGG , 4
AGCCTCTCTTGTCCCGGCAGGGTGGACCCATGGGAGTGCTATCAGGACACCTGGCAGACAACCTGCAGTG
TGTTGGAGCACCATCGAGACCTGATGAGGGTAAGATGGGCTGTGGCTGAGG , 3

AGCCTCTCTTGTCCCAGCAGGGTGGACCCATGGGAGTGCTATCAGGACACCTGGCAGACAACCTGCAGTG
TGTTGGAGCACCATCGAGACCTGATGAAGGTAAGATGGGCTGTGGCTGAGG , 3
AGCCTCTCTTGTCCCGGCAGGGTGGACCCATGAGAGTGCTATCAGGACACCTGGCAGACAACCTGCAGTG
TGTTGGAGCACCATCGAGACCTGATGAAGGTAAGATGGGCTGTGGCTGAGG , 3
AGCCTCTCTTGTCCCGGCAGGGTGGACCCATGGGAGTGCTATCAGGACACCTGGCAGACAACCTGCGGTG
TGTTGGAGCACCATCGAGACCTGATGAAGGTAAGATGGGCTGTGGCTGAGG , 3
AGCCTCTCTTGTCCCGGCAGGGTGGACCCATGGGAGTACTATCAGGACACCTGGCAGACAACCTGCAGTG
TGTTGGAGCACCATCGAGACCTGATGAAGGTAAGATGGGCTGTGGCTGAGG , 2
AGCCTCTCTTGTCCCGGCAGGGTGGACCCATGGGAGTGCTATCAGGACACCTGGCGGACAACCTGCAGTG
TGTTGGAGCACCATCGAGACCTGATGAAGGTAAGATGGGCTGTGGCTGAGG , 2

GEIC-Plate04-E07 TOTAL:3700 OrderedDict([('sp2', 2119), ('T95M', 1), ('T95M only', 0), ('T95M Full ssODN', 1), ('V98M', 1538), ('V98M_only', 11), ('V98M Full ssODN', 1486), ('Silent Block only', 0), ('Silent Block only Full ssODN', 0)])   [(0, 3698), (-1, 2)]
AGCCTCTCTTGTCCCGGCAGGGTGGACCCATGGGAGTGCTATCAGGACACCTGGCAGACAACCTGCAGTG
TGTTGGAGCACCATCGAGACCTGATGAAGGTAAGATGGGCTGTGGCTGAGG , 2040
AGCCTCTCTTGTCCCGGCAGGGTGGACCCATGGGAGTGCTATCAGGACACCTGGCAGACAACATGCAGTA
TGTTGGAGCACCATCGAGACCTGATGAAGGTAAGATGGGCTGTGGCTGAGG , 1486
AGCCTCTCTTGTCCCGGCAGGGTGGACCCATGGGAGTGCTATCAGGACACCTGGCAGACAACCTGCAGTA
TGTTGGAGCACCATCGAGACCTGATGAAGGTAAGATGGGCTGTGGCTGAGG , 11
AGCCTCTCTTGTCCCGGCAGGGTGGACCCATGGGAGTGCTATCAGGACACCTGGCAGACAACATGCAGTG
TGTTGGAGCACCATCGAGACCTGATGAAGGTAAGATGGGCTGTGGCTGAGG , 7
AGCCTCTCTTGTCCCGGCAGGGTGGACCCATGGGAGTGCTATCAGGACACCTGGCAGACAACCTGCAGTG
TGTTGGAGCACCATCGGGACCTGATGAAGGTAAGATGGGCTGTGGCTGAGG , 4
AGCCTCTCTTGTCCCGGCAGGGTGGAACCATGGGAGTGCTATCAGGACACCTGGCAGACAACCTGCAGTG
TGTTGGAGCACCATCGAGACCTGATGAAGGTAAGATGGGCTGTGGCTGAGG , 3
AGCCTCTCTTGTCCCGGCAGGGTGGACCCATGGGGGTGCTATCAGGACACCTGGCAGACAACATGCAGTA
TGTTGGAGCACCATCGAGACCTGATGAAGGTAAGATGGGCTGTGGCTGAGG , 3
AGCCTCTCTTGTCCCGGCAGGGTGGACCCATGGGAGTGCTATCAGGACACCTGGCAGACAACCTGCAGTG
GGTTGGAGCACCATCGAGACCTGATGAAGGTAAGATGGGCTGTGGCTGAGG , 3
AGCCTCTCTTGTCCCGGCAGGGTGGACCCATGGGAGTGCTATCAGGACACCTGGCAGACAACCTGCAGTG
TGTTGGAACACCATCGAGACCTGATGAAGGTAAGATGGGCTGTGGCTGAGG , 3
AGCCTCTCTTGTCCCGGCAGGGCGGACCCATGGGAGTGCTATCAGGACACCTGGCAGACAACCTGCAGTG
TGTTGGAGCACCATCGAGACCTGATGAAGGTAAGATGGGCTGTGGCTGAGG , 3
AGCCTCTCTTGTCCCGGCAGGGTGGACCCATGGGAGTGCTATCAGGACACCTGGCAGACAACCTTCAGTG
TGTTGGAGCACCATCGAGACCTGATGAAGGTAAGATGGGCTGTGGCTGAGG , 2
AGCCTCTCTTGTCCCGGCAGGGCGGACCCATGGGAGTGCTATCAGGACACCTGGCAGACAACATGCAGTA
TGTTGGAGCACCATCGAGACCTGATGAAGGTAAGATGGGCTGTGGCTGAGG , 2

GEIC-Plate04-E08 TOTAL:3039 OrderedDict([('sp2', 1541), ('T95M', 1), ('T95M only', 0), ('T95M Full ssODN', 1), ('V98M', 1478), ('V98M_only', 4), ('V98M Full ssODN', 1421), ('Silent Block only', 0), ('Silent Block only Full ssODN', 0)])   [(0, 3038), (-1, 1)]
AGCCTCTCTTGTCCCGGCAGGGTGGACCCATGGGAGTGCTATCAGGACACCTGGCAGACAACCTGCAGTG
TGTTGGAGCACCATCGAGACCTGATGAAGGTAAGATGGGCTGTGGCTGAGG , 1497
AGCCTCTCTTGTCCCGGCAGGGTGGACCCATGGGAGTGCTATCAGGACACCTGGCAGACAACATGCAGTA
TGTTGGAGCACCATCGAGACCTGATGAAGGTAAGATGGGCTGTGGCTGAGG , 1421

AGCCTCTCTTGTCCCGGCAGGGCGGACCCATGGGAGTGCTATCAGGACACCTGGCAGACAACATGCAGTA
TGTTGGAGCACCATCGAGACCTGATGAAGGTAAGATGGGCTGTGGCTGAGG , 7
AGCCTCTCTTGTCCCGGCAGGGTGGACCCATGGGAGTGCTATCAGGACACCTGGCAGACAACATGCAGTG
TGTTGGAGCACCATCGAGACCTGATGAAGGTAAGATGGGCTGTGGCTGAGG , 6
AGCCTCTCTTGTCCCGGCAGGGTGGACCCATGGGAGTGCTATCAGGACACCTGGCAGACAACCTGCAGTA
TGTTGGAGCACCATCGAGACCTGATGAAGGTAAGATGGGCTGTGGCTGAGG , 4
AGCCTCTCTTGTCCCGGCAGGGGGGACCCATGGGAGTGCTATCAGGACACCTGGCAGACAACCTGCAGTG
TGTTGGAGCACCATCGAGACCTGATGAAGGTAAGATGGGCTGTGGCTGAGG , 3
AGCCTCTCTTGTCCCGGCAGGGTGGACCCATGGGAGTGCTATCAGGACACCTGGCAGACAACATGCAGTA
TGTTAGAGCACCATCGAGACCTGATGAAGGTAAGATGGGCTGTGGCTGAGG , 2
AGCCTCTCTTGTCCCGGCAGGGTGGACCCATGGGAGTGGTATCAGGACACCTGGCAGACAACCTGCAGTG
TGTTGGAGCACCATCGAGACCTGATGAAGGTAAGATGGGCTGTGGCTGAGG , 2
AGCCTCTCTTGTCCCGGCAGGGTGGACCCATGGGAGTGCTATCAGGACACCTGGCAGACAACATGCAGTA
TGTTGGAGCACCATCGAGACCTGATGAGGGTAAGATGGGCTGTGGCTGAGG , 2
AGCCTCTCTTGTCCCGGCAGGGTGGGCCCATGGGAGTGCTATCAGGACACCTGGCAGACAACCTGCAGTG
TGTTGGAGCACCATCGAGACCTGATGAAGGTAAGATGGGCTGTGGCTGAGG , 2
AGCCTCTCTTGTCCCGGCAGGGTGGACCCATGGGAGTGCTATCAGGGCACCTGGCAGACAACCTGCAGTG
TGTTGGAGCACCATCGAGACCTGATGAAGGTAAGATGGGCTGTGGCTGAGG , 2
AGCCTCTCTTGTCCCGGCAGGGAGGACCCATGGGAGTGCTATCAGGACACCTGGCAGACAACCTGCAGTG
TGTTGGAGCACCATCGAGACCTGATGAAGGTAAGATGGGCTGTGGCTGAGG , 2

GEIC-Plate04-F01 TOTAL:3109 OrderedDict([('sp2', 3083), ('T95M', 1), ('T95M only', 0), ('T95M Full ssODN', 0), ('V98M', 9), ('V98M_only', 0), ('V98M Full ssODN', 8), ('Silent Block only', 0), ('Silent Block only Full ssODN', 0)])   [(0, 3108), (-1, 1)]
AGCCTCTCTTGTCCCGGCAGGGTGGACCCATGGGAGTGCTATCAGGACACCTGGCAGACAACCTGCAGTG
TGTTGGAGCACCATCGAGACCTGATGAAGGTAAGATGGGCTGTGGCTGAGG , 2972
AGCCTCTCTTGTCCCGGCAGGGTGGACCCATGGGAGTGCTATCAGGACACCTGGCAGACAACATGCAGTA
TGTTGGAGCACCATCGAGACCTGATGAAGGTAAGATGGGCTGTGGCTGAGG , 8
AGCCTCTCTTGTCCCGGCAGGGAGGACCCATGGGAGTGCTATCAGGACACCTGGCAGACAACCTGCAGTG
TGTTGGAGCACCATCGAGACCTGATGAAGGTAAGATGGGCTGTGGCTGAGG , 5
AGCCTCTCTTGTCCCGGCAGGGTGGACCCATGGGAGTGCTATCAGGACACCTGGCGGACAACCTGCAGTG
TGTTGGAGCACCATCGAGACCTGATGAAGGTAAGATGGGCTGTGGCTGAGG , 4
AGCCTCTCTTGTCCCGGCAGGGTGGACCCATGGGAGTGCTATCAGGACACCTGGCAGACAACCTGCAGTG
TGTTGGAGCACCATCGAGACCTGATGAGGGTAAGATGGGCTGTGGCTGAGG , 4
AGCCTCTCTTGTCCCGGCAGGGTGGACCCATGGGAGTGCTATCAGGACACCTGGCAGACAACCTGCAGTG
TGTTGGAGCACCATCAAGACCTGATGAAGGTAAGATGGGCTGTGGCTGAGG , 3
AGCCTCTCTTGTCCCGGCAGGGGGGACCCATGGGAGTGCTATCAGGACACCTGGCAGACAACCTGCAGTG
TGTTGGAGCACCATCGAGACCTGATGAAGGTAAGATGGGCTGTGGCTGAGG , 3
AGCCTCTCTTGTCCCGGCAGGGTGGACCCATGGGAGTGCTATCAGGACACCTGGCAGACAACCCGCAGTG
TGTTGGAGCACCATCGAGACCTGATGAAGGTAAGATGGGCTGTGGCTGAGG , 3
AGCCTCTCTTGTCCCGGCAGGGTGGACCCATGGGAGTGCTATCAGGACACCTGGCAGACAACCTGCAGTG
CGTTGGAGCACCATCGAGACCTGATGAAGGTAAGATGGGCTGTGGCTGAGG , 3
AGCCTCTCTTGTCCCGGCAGGGTGGACCCATGGGAGTGCTATCAGGACACCTGGCAGACAACCTGCAGTG
TGTTGGAGCTCCATCGAGACCTGATGAAGGTAAGATGGGCTGTGGCTGAGG , 3
AGCCTCTCTTGCCCCGGCAGGGTGGACCCATGGGAGTGCTATCAGGACACCTGGCAGACAACCTGCAGTG
TGTTGGAGCACCATCGAGACCTGATGAAGGTAAGATGGGCTGTGGCTGAGG , 3
AGCCTCTCTTGTCCCGGCAGGGTGGACCCATGGGAGTGCTATCAGGACACCTGGCAGACAACCTGCAGTG
TGTTGGAGCACCATCGAGACCTGACGAAGGTAAGATGGGCTGTGGCTGAGG , 2

GEIC-Plate04-F02 TOTAL:3100 OrderedDict([('sp2', 3084), ('T95M', 0), ('T95M only', 0), ('T95M Full ssODN', 0), ('V98M', 1), ('V98M_only', 0), ('V98M Full ssODN', 1), ('Silent Block only', 0), ('Silent Block only Full ssODN', 0)]) [(0, 3096), (-1, 4)]
AGCCTCTCTTGTCCCGGCAGGGTGGACCCATGGGAGTGCTATCAGGACACCTGGCAGACAACCTGCAGTG
TGTTGGAGCACCATCGAGACCTGATGAAGGTAAGATGGGCTGTGGCTGAGG , 2998
AGCCTCTCTTGTCCCGGCAGGGTGGACCCATGGGAGTGCTATCAGGACACCTGGCAGACAGCCTGCAGTG
TGTTGGAGCACCATCGAGACCTGATGAAGGTAAGATGGGCTGTGGCTGAGG , 5
AGCCTCTCTTGTCCCGGCAGGGTGGACCCATGGGAGTGCTATCAGGACACCTGGCAGACAACCTGCAGTG
TGTTGGAGCACCATCGGGACCTGATGAAGGTAAGATGGGCTGTGGCTGAGG , 4
AGCCTCTCTTGTCCCGGCAGGGCGGACCCATGGGAGTGCTATCAGGACACCTGGCAGACAACCTGCAGTG
TGTTGGAGCACCATCGAGACCTGATGAAGGTAAGATGGGCTGTGGCTGAGG , 4
AGCCTCTCTTGTCCCGGCAGGGTGGACCCATGGGAGTGCTATCAGGACACCTGGCAGACAACCTGCAGTG
TGTTGGAGCACCATCGAGACCTGATGAAGGTAAGGTGGGCTGTGGCTGAGG , 3
AGCCTCTCTTGTCCCGGCAGGGTGGACCCATGGGAGTGCTATCAGGACACCTGGCAGACAACCTGCAGTG
TGTTGGAGCACCATCGAGACCTGAAGAAGGTAAGATGGGCTGTGGCTGAGG , 2
AGCCTCTCTTGTCACGGCAGGGTGGACCCATGGGAGTGCTATCAGGACACCTGGCAGACAACCTGCAGTG
TGTTGGAGCACCATCGAGACCTGATGAAGGTAAGATGGGCTGTGGCTGAGG , 2
AGCCTCTCTTGTCCCGGCAGGGTGGACTCATGGGAGTGCTATCAGGACACCTGGCAGACAACCTGCAGTG
TGTTGGAGCACCATCGAGACCTGATGAAGGTAAGATGGGCTGTGGCTGAGG , 2
AGCCTCTCTTGTCCCGGCAGGGTGGACCCATGGGAGTGCTATCAGGACACCTGGCAGACAACCTGCAGTG
TGTTGGAGCACCATCGAGACCTGATTAAGGTAAGATGGGCTGTGGCTGAGG , 2
AGCCTCTCTTATCCCGGCAGGGTGGACCCATGGGAGTGCTATCAGGACACCTGGCAGACAACCTGCAGTG
TGTTGGAGCACCATCGAGACCTGATGAAGGTAAGATGGGCTGTGGCTGAGG , 2
AGCCTCTCTTGTCCCGGCAGGGTGGACCCATGGGAGGGCTATCAGGACACCTGGCAGACAACCTGCAGTG
TGTTGGAGCACCATCGAGACCTGATGAAGGTAAGATGGGCTGTGGCTGAGG , 2
AGCCTCTCTTGTCCCGGCAGGGTGGACCCATGGGAGTGCTATCAGGACACCTGGCAGACAACCTGCAGTG
TGTTGGAGTACCATCGAGACCTGATGAAGGTAAGATGGGCTGTGGCTGAGG , 2

GEIC-Plate04-F03 TOTAL:2626 OrderedDict([('sp2', 2606), ('T95M', 0), ('T95M only', 2), ('T95M Full ssODN', 0), ('V98M', 3), ('V98M_only', 0), ('V98M Full ssODN', 3), ('Silent Block only', 0), ('Silent Block only Full ssODN', 0)]) [(0, 2626)]
AGCCTCTCTTGTCCCGGCAGGGTGGACCCATGGGAGTGCTATCAGGACACCTGGCAGACAACCTGCAGTG
TGTTGGAGCACCATCGAGACCTGATGAAGGTAAGATGGGCTGTGGCTGAGG , 2515
AGCCTCTCTTGTCCCGGCAGGGGGGACCCATGGGAGTGCTATCAGGACACCTGGCAGACAACCTGCAGTG
TGTTGGAGCACCATCGAGACCTGATGAAGGTAAGATGGGCTGTGGCTGAGG , 5
AGCCTCTCTTGTCCCGGCAGGGTGGACCCATGGGAGTGCTATCAGGACACCTGGCAGACAACCTGCAGCG
TGTTGGAGCACCATCGAGACCTGATGAAGGTAAGATGGGCTGTGGCTGAGG , 3
AGCCTCTCTTGTCCCGGCAGGGTGGGCCCATGGGAGTGCTATCAGGACACCTGGCAGACAACCTGCAGTG
TGTTGGAGCACCATCGAGACCTGATGAAGGTAAGATGGGCTGTGGCTGAGG , 3
AGCCTCTCTTGTCCCGGCAGGGTGGACCCATGGGAGTGCTATCAGGACACCTGGCAGACAACCTGCAGTG
TGTTGGAGCGCCATCGAGACCTGATGAAGGTAAGATGGGCTGTGGCTGAGG , 3
AGCCTCTCTTGTCCCGGCAGGGTGGACCCATGGGAGTGCTATCAGGACACCTGGCAGACAACCTGCAGTG
TGTTGGAGCACCATCGAGACCTGATGAAGGTAAAATGGGCTGTGGCTGAGG , 3
AGCCTCTCTTGTCCCGGCAGGGTGGACCCATGGGAGTGCTATCAGGACACCTGGCAGACAACATGCAGTA
TGTTGGAGCACCATCGAGACCTGATGAAGGTAAGATGGGCTGTGGCTGAGG , 3

AGCCTCTCTTGTCCCGGCAGGGTGGACCCATGGGAGTGCTATCAGGACACCTGGCAGACAACCTGCAGTG
TGCTGGAGCACCATCGAGACCTGATGAAGGTAAGATGGGCTGTGGCTGAGG , 3
AGCCTCTCTTGTCCCGGCAGGGTGGACCCATGGGAGTGCTATCAGAACACCTGGCAGACAACCTGCAGTG
TGTTGGAGCACCATCGAGACCTGATGAAGGTAAGATGGGCTGTGGCTGAGG , 2
AGCCTCTCTTGTCCCGGCAGGGTGGACCCATGGGAGTGCTATCAGGACACCTGGCGGACAACCTGCAGTG
TGTTGGAGCACCATCGAGACCTGATGAAGGTAAGATGGGCTGTGGCTGAGG , 2
AGCCTCTCTTGTCCCGGCAGGGTGGACCCATGGGAGTGCTATCAGGACACCTAGCAGACAACCTGCAGTG
TGTTGGAGCACCATCGAGACCTGATGAAGGTAAGATGGGCTGTGGCTGAGG , 2
AGCCTCTCTTGTCCCGGCAGGGTGGACCCATGGGAGTGCTATCAGGACACCTGGCAGACAACCTGCAGTG
TGTTGGAGCACCATCGAGACCTGATGAAGGCAAGATGGGCTGTGGCTGAGG , 2

GEIC-Plate04-F04 TOTAL:2236 OrderedDict([('sp2', 2219), ('T95M', 0), ('T95M only', 0), ('T95M Full ssODN', 0), ('V98M', 1), ('V98M_only', 0), ('V98M Full ssODN', 1), ('Silent Block only', 0), ('Silent Block only Full ssODN', 0)])   [(0, 2234), (-1, 1), (-2, 1)]
AGCCTCTCTTGTCCCGGCAGGGTGGACCCATGGGAGTGCTATCAGGACACCTGGCAGACAACCTGCAGTG
TGTTGGAGCACCATCGAGACCTGATGAAGGTAAGATGGGCTGTGGCTGAGG , 2134
AGCCTCTCTTGTCCCGGCAGGGTGGACCCATGGGAGTGCTATCAGGACACCTGGCAGACAACCTGCAGTG
GGTTGGAGCACCATCGAGACCTGATGAAGGTAAGATGGGCTGTGGCTGAGG , 5
AGCCTCTCTTGTCCCGGCAGGGTGGACCCATGGGAGTGCTATCAGGGCACCTGGCAGACAACCTGCAGTG
TGTTGGAGCACCATCGAGACCTGATGAAGGTAAGATGGGCTGTGGCTGAGG , 3
AGCCTCTCTTGTCCCGGCAGGGTGGACCCATGGGAGTGCTATCATGACACCTGGCAGACAACCTGCAGTG
TGTTGGAGCACCATCGAGACCTGATGAAGGTAAGATGGGCTGTGGCTGAGG , 3
AGCCTCTCTTGTCCCGGCAGGGTGGACCCATGGGAGTGCTATCAGGACACCTGGCAGAAAACCTGCAGTG
TGTTGGAGCACCATCGAGACCTGATGAAGGTAAGATGGGCTGTGGCTGAGG , 2
AGCCTCTCTTGTCCCGGCAGGGTGGACCCATGGGAGTGCTATCAGGACACCTGGCAGACAACCTGCAGTG
TGTTGGAGCACCATCGAGACCTGATGAAGGTAAGACGGGCTGTGGCTGAGG , 2
AGCCTCTCTTGTCCCGGCAGGGTGGACCCATGGGAGTGCTAACAGGACACCTGGCAGACAACCTGCAGTG
TGTTGGAGCACCATCGAGACCTGATGAAGGTAAGATGGGCTGTGGCTGAGG , 2
AGCCTCTCTTGTCCCGGCAGGGTGGACCCATGGGAGTGCTATCAGGACACCTGACAGACAACCTGCAGTG
TGTTGGAGCACCATCGAGACCTGATGAAGGTAAGATGGGCTGTGGCTGAGG , 2
AGCCTCTCTTGTCCCGGCAGGGTGAACCCATGGGAGTGCTATCAGGACACCTGGCAGACAACCTGCAGTG
TGTTGGAGCACCATCGAGACCTGATGAAGGTAAGATGGGCTGTGGCTGAGG , 2
AGCCTCTCTTGTCCCGGCAGGGTGGACCCATGGGAGTGCTATCAGGACACCTGGCAGACAACCTGCAGTG
TGTTGGAGCGCCATCGAGACCTGATGAAGGTAAGATGGGCTGTGGCTGAGG , 2
AGCCTCTCTTGTCCCGGCAGGGTGTACCCATGGGAGTGCTATCAGGACACCTGGCAGACAACCTGCAGTG
TGTTGGAGCACCATCGAGACCTGATGAAGGTAAGATGGGCTGTGGCTGAGG , 2
AGCCTCTCTTGCCCCGGCAGGGTGGACCCATGGGAGTGCTATCAGGACACCTGGCAGACAACCTGCAGTG
TGTTGGAGCACCATCGAGACCTGATGAAGGTAAGATGGGCTGTGGCTGAGG , 2

GEIC-Plate04-F05 TOTAL:3432 OrderedDict([('sp2', 3402), ('T95M', 0), ('T95M only', 1), ('T95M Full ssODN', 0), ('V98M', 0), ('V98M_only', 0), ('V98M Full ssODN', 0), ('Silent Block only', 0), ('Silent Block only Full ssODN', 0)])   [(0, 3430), (-1, 2)]
AGCCTCTCTTGTCCCGGCAGGGTGGACCCATGGGAGTGCTATCAGGACACCTGGCAGACAACCTGCAGTG
TGTTGGAGCACCATCGAGACCTGATGAAGGTAAGATGGGCTGTGGCTGAGG , 3274
AGCCTCTCTTGTCCCGGCAGGGGGGACCCATGGGAGTGCTATCAGGACACCTGGCAGACAACCTGCAGTG
TGTTGGAGCACCATCGAGACCTGATGAAGGTAAGATGGGCTGTGGCTGAGG , 6

AGCCTCTCTTGTCCCGGCAGGGTGGACCCATGGGAGTGCTATCAGGACACCTGGCAGACAACCTGCAGTG
TGTTGGAGCACCATCGAGGCCTGATGAAGGTAAGATGGGCTGTGGCTGAGG , 5
AGCCTCTCTTGTCCCGGCAGGGTGGACCCATGGGAGTGCTATCAGGACACCTGGCAGACAACCTGCAGTG
TGTTGGAGCACCATCGAGACCTGATGAAGGTAAGATGGGCCGTGGCTGAGG , 4
AGCCTCTCTTGTCCCGGCAGGGTGGACCCATGGGAGTGCTATCAGGACACCTGGCAGACAACCTGCAGTG
TGTTAGAGCACCATCGAGACCTGATGAAGGTAAGATGGGCTGTGGCTGAGG , 3
AGCCTCTCTTGTCCCGGCAGGGTGGACCCATGGGAGTGCTATCAGGACACCTGGCAGACAACCTGCAGCG
TGTTGGAGCACCATCGAGACCTGATGAAGGTAAGATGGGCTGTGGCTGAGG , 3
AGCCTCTCTTGTCCCGGCAGGGTGGACCCATGGGAGTGCTATCAGGACACCTGGCAGACAACCTGCAGTG
TGTTGGAGCACCATCGGGACCTGATGAAGGTAAGATGGGCTGTGGCTGAGG , 3
AGCCTCTCTTGTCCCGGCAGGGTGGACCCATGGGAGTGCTATCAGGACACCTGGCAGACAACCTGCAGTG
CGTTGGAGCACCATCGAGACCTGATGAAGGTAAGATGGGCTGTGGCTGAGG , 3
AGCCTCTCTTGTCCCGGCAGGGTGGACCCATGGGAGTGCTATCAGGACACCTGGCAGACAACCTGCAGTG
TGTTGGAGCACCATCGAGACCTGATGAGGGTAAGATGGGCTGTGGCTGAGG , 3
AGCCTCTCTTGTCCCGGCAGGGTGAACCCATGGGAGTGCTATCAGGACACCTGGCAGACAACCTGCAGTG
TGTTGGAGCACCATCGAGACCTGATGAAGGTAAGATGGGCTGTGGCTGAGG , 3
AGCCTCTCTTGTCCCGGCAGGGCGGACCCATGGGAGTGCTATCAGGACACCTGGCAGACAACCTGCAGTG
TGTTGGAGCACCATCGAGACCTGATGAAGGTAAGATGGGCTGTGGCTGAGG , 3
AGCCTCTCTTGTCCCGGCAGGGTGGACCCATGGGAGTGCTATCAGGACACCTGGCGGACAACCTGCAGTG
TGTTGGAGCACCATCGAGACCTGATGAAGGTAAGATGGGCTGTGGCTGAGG , 2

GEIC-Plate04-F06 TOTAL:3112 OrderedDict([('sp2', 3099), ('T95M', 0), ('T95M only', 0), ('T95M Full ssODN', 0), ('V98M', 0), ('V98M_only', 1), ('V98M Full ssODN', 0), ('Silent Block only', 0), ('Silent Block only Full ssODN', 0)])   [(0, 3111), (-1, 1)]
AGCCTCTCTTGTCCCGGCAGGGTGGACCCATGGGAGTGCTATCAGGACACCTGGCAGACAACCTGCAGTG
TGTTGGAGCACCATCGAGACCTGATGAAGGTAAGATGGGCTGTGGCTGAGG , 2968
AGCCTCTCTTGTCCCGGCAGGGGGGACCCATGGGAGTGCTATCAGGACACCTGGCAGACAACCTGCAGTG
TGTTGGAGCACCATCGAGACCTGATGAAGGTAAGATGGGCTGTGGCTGAGG , 7
AGCCTCTCTTGTCCCGGCAGGGTGGACCCATGGGAGTGCTATCGGGACACCTGGCAGACAACCTGCAGTG
TGTTGGAGCACCATCGAGACCTGATGAAGGTAAGATGGGCTGTGGCTGAGG , 6
AGCCTCTCTTGTCCCGGCAGGGTGGACCCATGGGAGTGCTATCAGGACACCTGGCAGACAACCTGCAGTG
TGTTGGAGCACCATCGAGACCTGATGAGGGTAAGATGGGCTGTGGCTGAGG , 5
AGCCTCTCTTGTCCCGGCAGGGTGGACCCATGGGAGTGCTATCAGGACACCTGGCGGACAACCTGCAGTG
TGTTGGAGCACCATCGAGACCTGATGAAGGTAAGATGGGCTGTGGCTGAGG , 4
AGCCTCTCTTGTCCCGGCAGGGTGGACCCATGGGAGTGCTATCAGGACACCTAGCAGACAACCTGCAGTG
TGTTGGAGCACCATCGAGACCTGATGAAGGTAAGATGGGCTGTGGCTGAGG , 4
AGCCTCTCTTGTCCCGGCAGGGTGGACCCATGGGAGTGCTATCAGGACACCTGGCAGACAACCTGCAGTG
TGTTGGAGCACCATCGAGACCTGATGGAGGTAAGATGGGCTGTGGCTGAGG , 4
AGCCTCTCTTGTCCCGGCAGGGTGGACCCATGGGAGTGCTATCAGGACACCTGGCAGACAACCTGCAGTG
TGTTGGAGCACCATCGGGACCTGATGAAGGTAAGATGGGCTGTGGCTGAGG , 3
AGCCTCTCTTGTCCCGGCAGGGTGGACCCATGGGAGTGCTATCAGGACACCTGGCAGACAACCTGCAGTG
TGTTGGAGCACCATCGAGGCCTGATGAAGGTAAGATGGGCTGTGGCTGAGG , 3
AGCCTCTCTTGTCCCGGCAGGGTGGAACCATGGGAGTGCTATCAGGACACCTGGCAGACAACCTGCAGTG
TGTTGGAGCACCATCGAGACCTGATGAAGGTAAGATGGGCTGTGGCTGAGG , 3
AGCCTCTCTTGTCCCGGCAGGGTGGACCCATGGGAGTGCTATCAGGACACCCGGCAGACAACCTGCAGTG
TGTTGGAGCACCATCGAGACCTGATGAAGGTAAGATGGGCTGTGGCTGAGG , 3
AGCCTCTCTTGTCCCGGCAGGGTGGACCCATGGGAGTGCTATCAGGACACCTGGCAGACAACCTGCAGTG
CGTTGGAGCACCATCGAGACCTGATGAAGGTAAGATGGGCTGTGGCTGAGG , 3

GEIC-Plate04-F07 TOTAL:3016 OrderedDict([('sp2', 1455), ('T95M', 0), ('T95M only', 0), ('T95M Full ssODN', 0), ('V98M', 1538), ('V98M_only', 1), ('V98M Full ssODN', 1469), ('Silent Block only', 0), ('Silent Block only Full ssODN', 0)])   [(0, 3012), (-1, 3), (1, 1)]
AGCCTCTCTTGTCCCGGCAGGGTGGACCCATGGGAGTGCTATCAGGACACCTGGCAGACAACATGCAGTATGTTGGAGCACCATCGAGACCTGATGAAGGTAAGATGGGCTGTGGCTGAGG , 1469
AGCCTCTCTTGTCCCGGCAGGGTGGACCCATGGGAGTGCTATCAGGACACCTGGCAGACAACCTGCAGTGTGTTGGAGCACCATCGAGACCTGATGAAGGTAAGATGGGCTGTGGCTGAGG , 1389
AGCCTCTCTTGTCCCGGCAGGGTGGACCCATGGGAGTGCTATCAGGACACCTGGCAGACAACATGCAGTATGTTGGAGCACCATCGGGACCTGATGAAGGTAAGATGGGCTGTGGCTGAGG , 4
AGCCTCTCTTGTCCCGACAGGGTGGACCCATGGGAGTGCTATCAGGACACCTGGCAGACAACCTGCAGTGTGTTGGAGCACCATCGAGACCTGATGAAGGTAAGATGGGCTGTGGCTGAGG , 4
AGCCTCTCTTGTCCCGGCAGGGTGGACCCATGGGAGGGCTATCAGGACACCTGGCAGACAACATGCAGTATGTTGGAGCACCATCGAGACCTGATGAAGGTAAGATGGGCTGTGGCTGAGG , 4
AGCCTCTCTTGTCCCGGCAGGGTGGACCCATGGGAGTGCTATCAGGACACCTGGCAGACAACCTGCAGTGTGTTGGAGCACCATCGAGACCTGATGAAGGTAGGATGGGCTGTGGCTGAGG , 4
AGCCTCTCTTGTCCCGGCAGGGTGGACCCATGGGAGTGCTATCAGGACACCTGGCAGACAGCATGCAGTATGTTGGAGCACCATCGAGACCTGATGAAGGTAAGATGGGCTGTGGCTGAGG , 3
AGCCTCTCTTGTCCCGGCAGGGTGGACCCATGGGAGTGCTATCAGGACACCTGGCAGACAACATGCAGTGTGTTGGAGCACCATCGAGACCTGATGAAGGTAAGATGGGCTGTGGCTGAGG , 3
AGCCTCTCTTGTCCCGGCAGGGTGGACCCATGGGAGTGCTATCAGGACACCTGGCAGGCAACCTGCAGTGTGTTGGAGCACCATCGAGACCTGATGAAGGTAAGATGGGCTGTGGCTGAGG , 3
AGCCTCTCTTGTCCCGGCAGGGTGGACCCATGGGAGTGCTATCAGGACACCTGGCAGACAACATGCAGTATGTCGGAGCACCATCGAGACCTGATGAAGGTAAGATGGGCTGTGGCTGAGG , 2
AGCCTCTCTTGTCCCGGCAGGGTGGACCCATAGGAGTGCTATCAGGACACCTGGCAGACAACATGCAGTATGTTGGAGCACCATCGAGACCTGATGAAGGTAAGATGGGCTGTGGCTGAGG , 2
AGCCTCTCTTGTCCCGGCAGGGTGGACCCATGGGAGTGCTATCAGGACACCTGGCAGACAACATGCAGCATGTTGGAGCACCATCGAGACCTGATGAAGGTAAGATGGGCTGTGGCTGAGG , 2

GEIC-Plate04-F08 TOTAL:3062 OrderedDict([('sp2', 1566), ('T95M', 1466), ('T95M only', 0), ('T95M Full ssODN', 1423), ('V98M', 4), ('V98M_only', 0), ('V98M Full ssODN', 4), ('Silent Block only', 0), ('Silent Block only Full ssODN', 0)])   [(0, 3059), (-1, 3)]
AGCCTCTCTTGTCCCGGCAGGGTGGACCCATGGGAGTGCTATCAGGACACCTGGCAGACAACCTGCAGTGTGTTGGAGCACCATCGAGACCTGATGAAGGTAAGATGGGCTGTGGCTGAGG , 1506
AGCCTCTCTTGTCCCGGCAGGGTGGACCCATGGGAGTGCTATCAGGACACCTGGCAGACAATGTGCAGTGTGTTGGAGCACCATCGAGACCTGATGAAGGTAAGATGGGCTGTGGCTGAGG , 1423
AGCCTCTCTTGTCCCGGCAGGGGGGACCCATGGGAGTGCTATCAGGACACCTGGCAGACAACCTGCAGTGTGTTGGAGCACCATCGAGACCTGATGAAGGTAAGATGGGCTGTGGCTGAGG , 4
AGCCTCTCTTGTCCCGGCAGGGTGGACCCATGGGAGTGCTATCAGGACACCTGGCAGACAACATGCAGTATGTTGGAGCACCATCGAGACCTGATGAAGGTAAGATGGGCTGTGGCTGAGG , 4
AGCCTCTCTTGTCCCGGCAGGGTGGACCCATGGGAGTGCTATCAGGACGCCTGGCAGACAACCTGCAGTGTGTTGGAGCACCATCGAGACCTGATGAAGGTAAGATGGGCTGTGGCTGAGG , 4
AGCCTCTCTTGTCCCGGCAGGGTGGACCCATGGGAGTGCCATCAGGACACCTGGCAGACAACCTGCAGTGTGTTGGAGCACCATCGAGACCTGATGAAGGTAAGATGGGCTGTGGCTGAGG , 3
AGCCTCTCTTGTCCCGGCAGGGTGGACCCATGGGAGTGCTATCAGGACACCTGGCAGACAACCTGCAGTG

TGTTGGAGCACCATCGAGGCCTGATGAAGGTAAGATGGGCTGTGGCTGAGG , 3
AGCCTCTCTTGTCCCGGCAGGGTGGACCCATGGGAGTGCTATCAGGACACCTGGCAGACAACCTGCAGCG
TGTTGGAGCACCATCGAGACCTGATGAAGGTAAGATGGGCTGTGGCTGAGG , 3
AGCCTCTCTTGTCCCGGCAGGGAGGACCCATGGGAGTGCTATCAGGACACCTGGCAGACAACCTGCAGTG
TGTTGGAGCACCATCGAGACCTGATGAAGGTAAGATGGGCTGTGGCTGAGG , 3
AGCCTCTCTTGTCCCGGCAGGGTGGACCCATGGGAGTGCTATCAGGACACCTGGCAAACAATGTGCAGTG
TGTTGGAGCACCATCGAGACCTGATGAAGGTAAGATGGGCTGTGGCTGAGG , 2
AGCCTCTCTTGTCCCGGCAGGGTGGGCCCATGGGAGTGCTATCAGGACACCTGGCAGACAATGTGCAGTG
TGTTGGAGCACCATCGAGACCTGATGAAGGTAAGATGGGCTGTGGCTGAGG , 2
AGCCTCTCTTGCCCCGGCAGGGTGGACCCATGGGAGTGCTATCAGGACACCTGGCAGACAATGTGCAGTG
TGTTGGAGCACCATCGAGACCTGATGAAGGTAAGATGGGCTGTGGCTGAGG , 2

GEIC-Plate04-G01 TOTAL:3243 OrderedDict([('sp2', 3228), ('T95M', 0), ('T95M only', 0), ('T95M Full ssODN', 0), ('V98M', 0), ('V98M_only', 0), ('V98M Full ssODN', 0), ('Silent Block only', 0), ('Silent Block only Full ssODN', 0)])   [(0, 3238), (-1, 5)]
AGCCTCTCTTGTCCCGGCAGGGTGGACCCATGGGAGTGCTATCAGGACACCTGGCAGACAACCTGCAGTG
TGTTGGAGCACCATCGAGACCTGATGAAGGTAAGATGGGCTGTGGCTGAGG , 3101
AGCCTCTCTTGTCCCGGCAGGGGGGACCCATGGGAGTGCTATCAGGACACCTGGCAGACAACCTGCAGTG
TGTTGGAGCACCATCGAGACCTGATGAAGGTAAGATGGGCTGTGGCTGAGG , 4
AGCCTCTCTTGTCCCGGCAGGGTGGACCCATGGGAGTGCTATCAGGACACCTGGCAGACAACCTGCAGTG
TGTTGGAGCACCATCGGGACCTGATGAAGGTAAGATGGGCTGTGGCTGAGG , 4
AGCCTCTCTTGTCCCGGCAGGGTGGACCCATGGGAGTGCTATCAGGACACCTGGCAGACAACCTGCAGTG
TGTTGGAGCACCATCGAGACCTTATGAAGGTAAGATGGGCTGTGGCTGAGG , 4
AGCCTCTCTTGTCCCGGCAGGGTGGACCCATGGGAGTACTATCAGGACACCTGGCAGACAACCTGCAGTG
TGTTGGAGCACCATCGAGACCTGATGAAGGTAAGATGGGCTGTGGCTGAGG , 3
AGCCTCTCTTGTCCCGGCAGGGAGGACCCATGGGAGTGCTATCAGGACACCTGGCAGACAACCTGCAGTG
TGTTGGAGCACCATCGAGACCTGATGAAGGTAAGATGGGCTGTGGCTGAGG , 3
AGCCTCTCTTGTCCCGGCAGGGTGGACCCATGGGAGTGCTATCAGGACACCTGGCAGACAACCTGCAGTG
GGTTGGAGCACCATCGAGACCTGATGAAGGTAAGATGGGCTGTGGCTGAGG , 3
AGCCTCTCTTGTCACGGCAGGGTGGACCCATGGGAGTGCTATCAGGACACCTGGCAGACAACCTGCAGTG
TGTTGGAGCACCATCGAGACCTGATGAAGGTAAGATGGGCTGTGGCTGAGG , 3
AGCCTCTCTTGTCCCGGCAGGGTGGACCCATGGGAGTGCTATCAGGGCACCTGGCAGACAACCTGCAGTG
TGTTGGAGCACCATCGAGACCTGATGAAGGTAAGATGGGCTGTGGCTGAGG , 2
AGCCTCTCTTGTCCCGGCAGGGTGGACCCATGGGAGTGCTATCAGGACACCTGGCAGACAACCTGTAGTG
TGTTGGAGCACCATCGAGACCTGATGAAGGTAAGATGGGCTGTGGCTGAGG , 2
AGCCTCTCTTGTCCTGGCAGGGTGGACCCATGGGAGTGCTATCAGGACACCTGGCAGACAACCTGCAGTG
TGTTGGAGCACCATCGAGACCTGATGAAGGTAAGATGGGCTGTGGCTGAGG , 2
AGCCTCTCTTGTCCCGGCAGGGTGGACCCATGGAGTGCTATCAGGACACCTGGCAGACAACCTGCAGTGT
GTTGGAGCACCATCGAGACCTGATGAAGGTAAGATGGGCTGTGGCTGAGG , 2

GEIC-Plate04-G02 TOTAL:2811 OrderedDict([('sp2', 2795), ('T95M', 0), ('T95M only', 1), ('T95M Full ssODN', 0), ('V98M', 0), ('V98M_only', 0), ('V98M Full ssODN', 0), ('Silent Block only', 0), ('Silent Block only Full ssODN', 0)])   [(0, 2809), (-1, 2)]
AGCCTCTCTTGTCCCGGCAGGGTGGACCCATGGGAGTGCTATCAGGACACCTGGCAGACAACCTGCAGTG
TGTTGGAGCACCATCGAGACCTGATGAAGGTAAGATGGGCTGTGGCTGAGG , 2713
AGCCTCTCTTGTCCCGGCAGGGAGGACCCATGGGAGTGCTATCAGGACACCTGGCAGACAACCTGCAGTG

TGTTGGAGCACCATCGAGACCTGATGAAGGTAAGATGGGCTGTGGCTGAGG , 4
AGCCTCTCTTGTCCCGGCAGGGTGGACCCATGGGAGTGCTATCAGGACACCTGGCAGACAACCTGCAGTG
GGTTGGAGCACCATCGAGACCTGATGAAGGTAAGATGGGCTGTGGCTGAGG , 4
AGCCTCTCTTGTCCCGGCAGGGTGGACCCATGGGAGTGCTATCAGGACACCTGGCAGACAACCCGCAGTG
TGTTGGAGCACCATCGAGACCTGATGAAGGTAAGATGGGCTGTGGCTGAGG , 3
AGCCTCTCTTGTCCCGGCAGGGTGGACCCATGGGAGTGCTATCAGGACACCTGGCAGACAACCTGCAGTG
TGTTGGAGCACCATCGAGACCTGATGAAGGCAAGATGGGCTGTGGCTGAGG , 3
AGCCTCTCTTGTCCCGGCAGGGGGGACCCATGGGAGTGCTATCAGGACACCTGGCAGACAACCTGCAGTG
TGTTGGAGCACCATCGAGACCTGATGAAGGTAAGATGGGCTGTGGCTGAGG , 3
AGCCTCTCTTGTCCCGGCAGGGTGGACCCATGGGGGTGCTATCAGGACACCTGGCAGACAACCTGCAGTG
TGTTGGAGCACCATCGAGACCTGATGAAGGTAAGATGGGCTGTGGCTGAGG , 3
AGCCTCTCTTGTCCCGGCAGGGCGGACCCATGGGAGTGCTATCAGGACACCTGGCAGACAACCTGCAGTG
TGTTGGAGCACCATCGAGACCTGATGAAGGTAAGATGGGCTGTGGCTGAGG , 3
AGCCTCTCTTGTCCCGGCAGGGTGGACCCATGGGAGTGCTATCAGGACACCTGGCAGACGACCTGCAGTG
TGTTGGAGCACCATCGAGACCTGATGAAGGTAAGATGGGCTGTGGCTGAGG , 2
AGCCTCTCTTGTCCCGGCAGGGTGGACCCATGGGAGTGCTATCAGGACACCTGGCAGACAACCTGCAGTG
TGTTGGAGCACCATCGAGGCCTGATGAAGGTAAGATGGGCTGTGGCTGAGG , 2
AGCCTCTCTTGTCCCGGCAGGGTGGACCCATGGGAGTGCTATCAGGACACCTGGCAGGCAACCTGCAGTG
TGTTGGAGCACCATCGAGACCTGATGAAGGTAAGATGGGCTGTGGCTGAGG , 2
AGCCTCTCTTGTCCCGGCAGGGTGGACCCATGGGAGTGCTATCAGGACACCTGACAGACAACCTGCAGTG
TGTTGGAGCACCATCGAGACCTGATGAAGGTAAGATGGGCTGTGGCTGAGG , 2

GEIC-Plate04-G03 TOTAL:2414 OrderedDict([('sp2', 2392), ('T95M', 0), ('T95M only', 0), ('T95M Full ssODN', 0), ('V98M', 0), ('V98M_only', 0), ('V98M Full ssODN', 0), ('Silent Block only', 0), ('Silent Block only Full ssODN', 0)])   [(0, 2413), (-1, 1)]
AGCCTCTCTTGTCCCGGCAGGGTGGACCCATGGGAGTGCTATCAGGACACCTGGCAGACAACCTGCAGTG
TGTTGGAGCACCATCGAGACCTGATGAAGGTAAGATGGGCTGTGGCTGAGG , 2314
AGCCTCTCTTGTCCCGGCAGGGTGGACCCATGGGAGTGCTATCAGGACACCTGGCAGACAACCTGCAGTT
TGTTGGAGCACCATCGAGACCTGATGAAGGTAAGATGGGCTGTGGCTGAGG , 5
AGCCTCTCTTGTCCCGGCAGGGTGGACCCATGGGAGTGCTATCAGGACACCTGGCAGACAACCTGCAGTG
TGTTGGAGCACCATCGAGACCTGATGAAGGTGAGATGGGCTGTGGCTGAGG , 4
AGCCTCTCTTGTCCCGGCAGGGCGGACCCATGGGAGTGCTATCAGGACACCTGGCAGACAACCTGCAGTG
TGTTGGAGCACCATCGAGACCTGATGAAGGTAAGATGGGCTGTGGCTGAGG , 4
AGCCTCTCTTGTCCCGGCAGGGTGGGCCCATGGGAGTGCTATCAGGACACCTGGCAGACAACCTGCAGTG
TGTTGGAGCACCATCGAGACCTGATGAAGGTAAGATGGGCTGTGGCTGAGG , 3
AGCCTCTCTTGTCCCGGCAGGGTGGACCCATGGGAGTGCTATCAGGACACCTGGCAGACAACCTGCAGTG
TGTTGGAGCACCATCGGGACCTGATGAAGGTAAGATGGGCTGTGGCTGAGG , 3
AGCCTCTCTTGTCCCGGCAGGGTGGACCCATGGGAGTGCTATCAGGACACCTGGCAGACAACCTGCAGTG
GGTTGGAGCACCATCGAGACCTGATGAAGGTAAGATGGGCTGTGGCTGAGG , 3
AGCCTCTCTTGTCCCGGCAGGGTGGACCCATGGGAGTGCTATCAGGACACCTGGCAGACAACCTGCAGTG
TGTTGGAGCACCATCGAGGCCTGATGAAGGTAAGATGGGCTGTGGCTGAGG , 3
AGCCTCTCTTGTCCCGGCAGGGTGGACCCATGGGAGTGCTAACAGGACACCTGGCAGACAACCTGCAGTG
TGTTGGAGCACCATCGAGACCTGATGAAGGTAAGATGGGCTGTGGCTGAGG , 2
AGCCTCTCTTGTCCCGGCAGGGTGGACCCATGGGAGTGCTATCAGGACACCTGGCAGACAACCTGCAGTG
TGTTGGAGCACCATCGAGACCTGATGAAGGTAAGATGAGCTGTGGCTGAGG , 2
AGCCTCTCTTGTCCCGGCAGGGTGGACCCATGGGAGTGCTATCAGGACACCTGGCAGACAACCTGCAGCG
TGTTGGAGCACCATCGAGACCTGATGAAGGTAAGATGGGCTGTGGCTGAGG , 2
AGCCTCTCTTGTCCCGGCAGGGTGGACCCATGGGAGTGCTATCAGGACACCTGGCAGGCAACCTGCAGTG

TGTTGGAGCACCATCGAGACCTGATGAAGGTAAGATGGGCTGTGGCTGAGG , 2

GEIC-Plate04-G04 TOTAL:3438 OrderedDict([('sp2', 3418), ('T95M', 0), ('T95M only', 1), ('T95M Full ssODN', 0), ('V98M', 1), ('V98M_only', 0), ('V98M Full ssODN', 1), ('Silent Block only', 0), ('Silent Block only Full ssODN', 0)]) [(0, 3438)]
AGCCTCTCTTGTCCCGGCAGGGTGGACCCATGGGAGTGCTATCAGGACACCTGGCAGACAACCTGCAGTG
TGTTGGAGCACCATCGAGACCTGATGAAGGTAAGATGGGCTGTGGCTGAGG , 3308
AGCCTCTCTTGTCCCGGCAGGGGGGACCCATGGGAGTGCTATCAGGACACCTGGCAGACAACCTGCAGTG
TGTTGGAGCACCATCGAGACCTGATGAAGGTAAGATGGGCTGTGGCTGAGG , 7
AGCCTCTCTTGTCCCGGCAGGGAGGACCCATGGGAGTGCTATCAGGACACCTGGCAGACAACCTGCAGTG
TGTTGGAGCACCATCGAGACCTGATGAAGGTAAGATGGGCTGTGGCTGAGG , 4
AGCCTCTCTTGTCCCGGCAGGGTGGACCCATGGGAGTGCTATCAGGACACCTGGCAGACAACCTGCAGTG
TGTTGGAGCACCATCGAGGCCTGATGAAGGTAAGATGGGCTGTGGCTGAGG , 4
AGCCTCTCTTGTCCCGGCAGGGCGGACCCATGGGAGTGCTATCAGGACACCTGGCAGACAACCTGCAGTG
TGTTGGAGCACCATCGAGACCTGATGAAGGTAAGATGGGCTGTGGCTGAGG , 3
AGCCTCTCTTGTCCCGGCAGGGTGGACCCATGGGAGTGCTATCAGGACACCTGGCGGACAACCTGCAGTG
TGTTGGAGCACCATCGAGACCTGATGAAGGTAAGATGGGCTGTGGCTGAGG , 2
AGCCTCTCTTGTCCCGGCAGGGTGGACCCATGGGAGTGCTATCAGGACACCTAGCAGACAACCTGCAGTG
TGTTGGAGCACCATCGAGACCTGATGAAGGTAAGATGGGCTGTGGCTGAGG , 2
AGCCTCTCTTGTCCCGGCAGGGTGGACCCATGGGAGTGCTATCAGGACACCTGGCAGACAACCTGCAGTG
TGTTGGAGCACCATCGAGACCTGATAAAGGTAAGATGGGCTGTGGCTGAGG , 2
AGCCTCTCTTGTCCCGGCAAGGTGGACCCATGGGAGTGCTATCAGGACACCTGGCAGACAACCTGCAGTG
TGTTGGAGCACCATCGAGACCTGATGAAGGTAAGATGGGCTGTGGCTGAGG , 2
AGCCTCTCTTGTCCCGACAGGGTGGACCCATGGGAGTGCTATCAGGACACCTGGCAGACAACCTGCAGTG
TGTTGGAGCACCATCGAGACCTGATGAAGGTAAGATGGGCTGTGGCTGAGG , 2
AGCCTCTCTTGTCCCGGCAGGGTGGACCCATGGGAGTGCTATCAGGACACCTGGCAGACAACCTGCAGTG
TGCTGGAGCACCATCGAGACCTGATGAAGGTAAGATGGGCTGTGGCTGAGG , 2
AGCCTCTCTTGTCCCGGCAGGGTGGACCCATGGGAGTGCTATCAGGACACCTGGCAGACAACCTGCAGCG
TGTTGGAGCACCATCGAGACCTGATGAAGGTAAGATGGGCTGTGGCTGAGG , 2

GEIC-Plate04-G05 TOTAL:3416 OrderedDict([('sp2', 3383), ('T95M', 0), ('T95M only', 1), ('T95M Full ssODN', 0), ('V98M', 0), ('V98M_only', 0), ('V98M Full ssODN', 0), ('Silent Block only', 0), ('Silent Block only Full ssODN', 0)]) [(0, 3415), (-1, 1)]
AGCCTCTCTTGTCCCGGCAGGGTGGACCCATGGGAGTGCTATCAGGACACCTGGCAGACAACCTGCAGTG
TGTTGGAGCACCATCGAGACCTGATGAAGGTAAGATGGGCTGTGGCTGAGG , 3238
AGCCTCTCTTGTCCCGGCAGGGGGGACCCATGGGAGTGCTATCAGGACACCTGGCAGACAACCTGCAGTG
TGTTGGAGCACCATCGAGACCTGATGAAGGTAAGATGGGCTGTGGCTGAGG , 5
AGCCTCTCTTGTCCCGGCAGGGTGGACCCATGGGAGTGCTATCAGGACACCTGGCAGACAACCTGCAGTG
CGTTGGAGCACCATCGAGACCTGATGAAGGTAAGATGGGCTGTGGCTGAGG , 5
AGCCTCTCTTGTCCCGGCAGGGCGGACCCATGGGAGTGCTATCAGGACACCTGGCAGACAACCTGCAGTG
TGTTGGAGCACCATCGAGACCTGATGAAGGTAAGATGGGCTGTGGCTGAGG , 5
AGCCTCTCTTGTCCCGGCAGGGTGGACCCATGGGAGTGCTATCAGGACACCTGGCAGACAACCTGCAGTG
TGTTGGAGCACCATCGAGTCCTGATGAAGGTAAGATGGGCTGTGGCTGAGG , 4
AGCCTCTCTTGTCCCGGCAGGGTGGACCCATGGGAGTGCTATCAGGACACCTGGCGGACAACCTGCAGTG
TGTTGGAGCACCATCGAGACCTGATGAAGGTAAGATGGGCTGTGGCTGAGG , 3
AGCCTCTCTTGTCCTGGCAGGGTGGACCCATGGGAGTGCTATCAGGACACCTGGCAGACAACCTGCAGTG

TGTTGGAGCACCATCGAGACCTGATGAAGGTAAGATGGGCTGTGGCTGAGG , 3
AGCCTCTCTTGTCCCGACAGGGTGGACCCATGGGAGTGCTATCAGGACACCTGGCAGACAACCTGCAGTG
TGTTGGAGCACCATCGAGACCTGATGAAGGTAAGATGGGCTGTGGCTGAGG , 3
AGCCTCTCTTGTCCCGGCAGGGTGGACCCATGGGAGTGCTATCAGGACACCTGGCAGACAACCTGCAGTG
TGTTGGAGCACCATCGAGACCTGATGAAGGTGAGATGGGCTGTGGCTGAGG , 3
AGCCTCTCTTGTCCCGGCAGGGTGGACCCATGGGAGTGCTATCAGGACACCTGGCAGACAACCTGCAGTG
GGTTGGAGCACCATCGAGACCTGATGAAGGTAAGATGGGCTGTGGCTGAGG , 3
AGCCTCTCTTGTCCCGGCAGGGTGGACCCATGGGAGTGCTATCAGGACTCCTGGCAGACAACCTGCAGTG
TGTTGGAGCACCATCGAGACCTGATGAAGGTAAGATGGGCTGTGGCTGAGG , 2
AGCCTCTCTTGTCCCGGCAGGGTGGACCCATGGGAGTGCTATCAGGACACCTAGCAGACAACCTGCAGTG
TGTTGGAGCACCATCGAGACCTGATGAAGGTAAGATGGGCTGTGGCTGAGG , 2

GEIC-Plate04-G06 TOTAL:3455 OrderedDict([('sp2', 3431), ('T95M', 0), ('T95M only', 1), ('T95M Full ssODN', 0), ('V98M', 2), ('V98M_only', 0), ('V98M Full ssODN', 2), ('Silent Block only', 0), ('Silent Block only Full ssODN', 0)])   [(0, 3452), (-1, 3)]
AGCCTCTCTTGTCCCGGCAGGGTGGACCCATGGGAGTGCTATCAGGACACCTGGCAGACAACCTGCAGTG
TGTTGGAGCACCATCGAGACCTGATGAAGGTAAGATGGGCTGTGGCTGAGG , 3304
AGCCTCTCTTGTCCCGGCAGGGTGGACCCATGGGAGTGCTATCAGGACACCTGGCAGACAACCTGCAGTG
TGTTGGAGCACCATCGAGGCCTGATGAAGGTAAGATGGGCTGTGGCTGAGG , 6
AGCCTCTCTTGTCCCGGCAGGGTGGACCCATGGGAGTGCTATCAGGACACCTGGCAGACAACCTGCAGTG
GGTTGGAGCACCATCGAGACCTGATGAAGGTAAGATGGGCTGTGGCTGAGG , 4
AGCCTCTCTTGTCCCGGCAGGGTGGACCCATGGGAGTGCTATCAGGACGCCTGGCAGACAACCTGCAGTG
TGTTGGAGCACCATCGAGACCTGATGAAGGTAAGATGGGCTGTGGCTGAGG , 4
AGCCTCTCTTGTCCCGGCAGGGTGGACCCATGGGAGTGCTATCAGGACACCTGGCAGACAACCTGCAGTG
TGTTGGAGCACCATCGAGACCTGATGAAGGCAAGATGGGCTGTGGCTGAGG , 4
AGCCTCTCTTGTCCCGGCAGGGCGGACCCATGGGAGTGCTATCAGGACACCTGGCAGACAACCTGCAGTG
TGTTGGAGCACCATCGAGACCTGATGAAGGTAAGATGGGCTGTGGCTGAGG , 4
AGCCTCTCTTGTCCCGGCAGGGTGGACCCATGGGAGTGCTATCAGGACACCTGGCAGACAACCTGCAGTG
TGTTGGAGCACCATCGAGACCTGATGAAAGTAAGATGGGCTGTGGCTGAGG , 3
AGCCTCTCTTGTCCCGGCAGGGTGGACCCATGGGAGTGCTATCAGGACACCTGGCAGACAACCTGCAGCG
TGTTGGAGCACCATCGAGACCTGATGAAGGTAAGATGGGCTGTGGCTGAGG , 3
AGCCTCTCTTGTCCCGGCAGGGTGGACCCATGGGAGTGCTATCAGGACACCTGGCAGACAACCTGCAGTG
CGTTGGAGCACCATCGAGACCTGATGAAGGTAAGATGGGCTGTGGCTGAGG , 3
AGCCTCTCTTGTCCCGGCAGGGTGGACCCATGGGAGGGCTATCAGGACACCTGGCAGACAACCTGCAGTG
TGTTGGAGCACCATCGAGACCTGATGAAGGTAAGATGGGCTGTGGCTGAGG , 3
AGCCTCTCTTGTCCCGGCAGGGTGGACCCATGGGAGTGCTATCAGGACACCTGGCAGACAACCTGCGGTG
TGTTGGAGCACCATCGAGACCTGATGAAGGTAAGATGGGCTGTGGCTGAGG , 3
AGCCTCTCTTGCCCCGGCAGGGTGGACCCATGGGAGTGCTATCAGGACACCTGGCAGACAACCTGCAGTG
TGTTGGAGCACCATCGAGACCTGATGAAGGTAAGATGGGCTGTGGCTGAGG , 3

GEIC-Plate04-G07 TOTAL:2886 OrderedDict([('sp2', 1404), ('T95M', 0), ('T95M only', 0), ('T95M Full ssODN', 0), ('V98M', 1451), ('V98M_only', 4), ('V98M Full ssODN', 1395), ('Silent Block only', 0), ('Silent Block only Full ssODN', 0)])   [(0, 2884), (-1, 2)]
AGCCTCTCTTGTCCCGGCAGGGTGGACCCATGGGAGTGCTATCAGGACACCTGGCAGACAACATGCAGTA
TGTTGGAGCACCATCGAGACCTGATGAAGGTAAGATGGGCTGTGGCTGAGG , 1395
AGCCTCTCTTGTCCCGGCAGGGTGGACCCATGGGAGTGCTATCAGGACACCTGGCAGACAACCTGCAGTG

TGTTGGAGCACCATCGAGACCTGATGAAGGTAAGATGGGCTGTGGCTGAGG , 1342
AGCCTCTCTTGTCCCGGCAGGGGGGACCCATGGGAGTGCTATCAGGACACCTGGCAGACAACCTGCAGTG
TGTTGGAGCACCATCGAGACCTGATGAAGGTAAGATGGGCTGTGGCTGAGG , 10
AGCCTCTCTTGTCCCGGCAGGGTGGACCCATGGGAGTGCTATCAGGACACCTGGCAGACAACATGCAGTG
TGTTGGAGCACCATCGAGACCTGATGAAGGTAAGATGGGCTGTGGCTGAGG , 5
AGCCTCTCTTGTCCCGGCAGGGTGGACCCATGGGAGTGCTATCAGGACACCTGGCAGACAACCTGCAGTG
CGTTGGAGCACCATCGAGACCTGATGAAGGTAAGATGGGCTGTGGCTGAGG , 4
AGCCTCTCTTGTCCCGGCAGGGTGGACCCATGGGAGTGCTATCAGGACACCTGGCAGACAACCTGCAGTA
TGTTGGAGCACCATCGAGACCTGATGAAGGTAAGATGGGCTGTGGCTGAGG , 4
AGCCTCTCTTGTCCCGGCAGGGTGGACCCATGGGAGGGCTATCAGGACACCTGGCAGACAACCTGCAGTG
TGTTGGAGCACCATCGAGACCTGATGAAGGTAAGATGGGCTGTGGCTGAGG , 4
AGCCTCTCTTGTCCCGGCAGGGTGGACCCATGGGAGTGCTATCAGGACACCTGGCAGACAACCTGCAGTG
TGTTGGAGCACCATCGAGGCCTGATGAAGGTAAGATGGGCTGTGGCTGAGG , 3
AGCCTCTCTTGTCCCGGCAGGGTGGACCTATGGGAGTGCTATCAGGACACCTGGCAGACAACCTGCAGTG
TGTTGGAGCACCATCGAGACCTGATGAAGGTAAGATGGGCTGTGGCTGAGG , 2
AGCCTCTCTTGTCCCGGCAGGGTGGACCCATGGGAGTGCTATCAGGGCACCTGGCAGACAACCTGCAGTG
TGTTGGAGCACCATCGAGACCTGATGAAGGTAAGATGGGCTGTGGCTGAGG , 2
AGCCTCTCTTGTCCCGGCAGGGTGGACCCATGGGAGTGCTATCAGGACACCTGGCAGACAACATGCAGTA
TGATGGAGCACCATCGAGACCTGATGAAGGTAAGATGGGCTGTGGCTGAGG , 2
AGCCTCTCTTGTCCCGGCAGGGTGGACCCATGGGAGTGCTATCAGGACACCTGACAGACAACATGCAGTA
TGTTGGAGCACCATCGAGACCTGATGAAGGTAAGATGGGCTGTGGCTGAGG , 2

GEIC-Plate04-G08 TOTAL:1889 OrderedDict([('sp2', 922), ('T95M', 945), ('T95M only', 0), ('T95M Full ssODN', 910), ('V98M', 2), ('V98M_only', 0), ('V98M Full ssODN', 2), ('Silent Block only', 0), ('Silent Block only Full ssODN', 0)])   [(0, 1888), (-1, 1)]
AGCCTCTCTTGTCCCGGCAGGGTGGACCCATGGGAGTGCTATCAGGACACCTGGCAGACAATGTGCAGTG
TGTTGGAGCACCATCGAGACCTGATGAAGGTAAGATGGGCTGTGGCTGAGG , 910
AGCCTCTCTTGTCCCGGCAGGGTGGACCCATGGGAGTGCTATCAGGACACCTGGCAGACAACCTGCAGTG
TGTTGGAGCACCATCGAGACCTGATGAAGGTAAGATGGGCTGTGGCTGAGG , 890
AGCCTCTCTTGTCCCGGCAGGGTGGACCCATGGGAGTGCTATCAGGACACCTGGCAGACAATGTGCAGTG
TGTTGGAGCGCCATCGAGACCTGATGAAGGTAAGATGGGCTGTGGCTGAGG , 3
AGCCTCTCTTGTCCCGGCAGGGTGGACCCATGGGAGTGCTATCAGGACACCTGGCAGACAACCTGCAGTG
GGTTGGAGCACCATCGAGACCTGATGAAGGTAAGATGGGCTGTGGCTGAGG , 3
AGCCTCTCTTGTCCCGGCAGGGAGGACCCATGGGAGTGCTATCAGGACACCTGGCAGACAATGTGCAGTG
TGTTGGAGCACCATCGAGACCTGATGAAGGTAAGATGGGCTGTGGCTGAGG , 2
AGCCTCTCTTGTCCCGGCAGGGTGGACCCATGGGAGTGCTATCAGGACACCTGGCAGACAATGTGCGGTG
TGTTGGAGCACCATCGAGACCTGATGAAGGTAAGATGGGCTGTGGCTGAGG , 2
AGCCTCTCTTGCCCCGGCAGGGTGGACCCATGGGAGTGCTATCAGGACACCTGGCAGACAATGTGCAGTG
TGTTGGAGCACCATCGAGACCTGATGAAGGTAAGATGGGCTGTGGCTGAGG , 2
AGCCTCTCTTGTCTCGGCAGGGTGGACCCATGGGAGTGCTATCAGGACACCTGGCAGACAATGTGCAGTG
TGTTGGAGCACCATCGAGACCTGATGAAGGTAAGATGGGCTGTGGCTGAGG , 2
AGCCTCTCTTGTCCCGGCAGGGGGGACCCATGGGAGTGCTATCAGGACACCTGGCAGACAACCTGCAGTG
TGTTGGAGCACCATCGAGACCTGATGAAGGTAAGATGGGCTGTGGCTGAGG , 2
AGCCTCTCTTGTCCCGGCAGGGTGGACCCATGGGAGTGCTATCAGGACACCTGGCAGACAACATGCAGTA
TGTTGGAGCACCATCGAGACCTGATGAAGGTAAGATGGGCTGTGGCTGAGG , 2
AGCCTCTCTTGTCCCGGCAGGGTGGACCCATGGGAGTGCTATCAGGACACCTGGCAGATAATGTGCAGTG
TGTTGGAGCACCATCGAGACCTGATGAAGGTAAGATGGGCTGTGGCTGAGG , 2
AGCCTCTCTTGTCCCGGCAGGGTGGACCCATGGGAGTGCTATCAGGATACCTGGCAGACAATGTGCAGTG

TGTTGGAGCACCATCGAGACCTGATGAAGGTAAGATGGGCTGTGGCTGAGG , 2

GEIC-Plate04-H01 TOTAL:2764 OrderedDict([('sp2', 2750), ('T95M', 0), ('T95M only', 0), ('T95M Full ssODN', 0), ('V98M', 0), ('V98M_only', 0), ('V98M Full ssODN', 0), ('Silent Block only', 0), ('Silent Block only Full ssODN', 0)])   [(0, 2762), (-1, 2)]
AGCCTCTCTTGTCCCGGCAGGGTGGACCCATGGGAGTGCTATCAGGACACCTGGCAGACAACCTGCAGTG
TGTTGGAGCACCATCGAGACCTGATGAAGGTAAGATGGGCTGTGGCTGAGG , 2653
AGCCTCTCTTGTCCCGGCAGGGTGGACCCATGGGAGTGCTATCAGGACACCTGGCAGACAACCTGCAGTG
TGTTGGAGCACCATCGAGACCTGGTGAAGGTAAGATGGGCTGTGGCTGAGG , 5
AGCCTCTCTTGTCCCGGCAGGGTGGACCCATGGGAGTGCTATCAGGACACCTGGCAGACAACCTGCAGTG
TGTTGGAGCACCATCGAGGCCTGATGAAGGTAAGATGGGCTGTGGCTGAGG , 5
AGCCTCTCTTGTCCTGGCAGGGTGGACCCATGGGAGTGCTATCAGGACACCTGGCAGACAACCTGCAGTG
TGTTGGAGCACCATCGAGACCTGATGAAGGTAAGATGGGCTGTGGCTGAGG , 4
AGCCTCTCTTGTCCCGGCAGGGTGGACCCATGGGAGTGCTATCAGGACACCTGGCAGACAACCTGCAGCG
TGTTGGAGCACCATCGAGACCTGATGAAGGTAAGATGGGCTGTGGCTGAGG , 3
AGCCTCTCTTGTCCCGGCAGGGTGGACCCATGGGAGTGCTATCAGGACACCTGGCAGACAACCTGCAGTG
TGTTGGAGCGCCATCGAGACCTGATGAAGGTAAGATGGGCTGTGGCTGAGG , 3
AGCCTCTCTTGTCCCGGCAGGGAGGACCCATGGGAGTGCTATCAGGACACCTGGCAGACAACCTGCAGTG
TGTTGGAGCACCATCGAGACCTGATGAAGGTAAGATGGGCTGTGGCTGAGG , 3
AGCCTCTCTTGTCCCGGCAGGGCGGACCCATGGGAGTGCTATCAGGACACCTGGCAGACAACCTGCAGTG
TGTTGGAGCACCATCGAGACCTGATGAAGGTAAGATGGGCTGTGGCTGAGG , 3
AGCCTCTCTTGTCCCGGCAGGGGGGACCCATGGGAGTGCTATCAGGACACCTGGCAGACAACCTGCAGTG
TGTTGGAGCACCATCGAGACCTGATGAAGGTAAGATGGGCTGTGGCTGAGG , 3
AGCCTCTCTTGTCCCGGCAGGGTGGACCCATGGGAGTGCTATCAGGACACCTGGCAGACAACCTGCAGTG
CGTTGGAGCACCATCGAGACCTGATGAAGGTAAGATGGGCTGTGGCTGAGG , 3
AGCCTCTCTTGTCCCGGCAGGGTGGACCCATGGGAGTGCTATCAGGACACCTGGCAGACAACCTGCAGTG
TGTTGGAGCACCATCGAGACCTGTTGAAGGTAAGATGGGCTGTGGCTGAGG , 2
AGCCTCTCTTGTCCCGGCAGGGTGGACCCACGGGAGTGCTATCAGGACACCTGGCAGACAACCTGCAGTG
TGTTGGAGCACCATCGAGACCTGATGAAGGTAAGATGGGCTGTGGCTGAGG , 2

GEIC-Plate04-H02 TOTAL:2002 OrderedDict([('sp2', 1976), ('T95M', 0), ('T95M only', 0), ('T95M Full ssODN', 0), ('V98M', 15), ('V98M_only', 1), ('V98M Full ssODN', 15), ('Silent Block only', 0), ('Silent Block only Full ssODN', 0)])   [(0, 2002)]
AGCCTCTCTTGTCCCGGCAGGGTGGACCCATGGGAGTGCTATCAGGACACCTGGCAGACAACCTGCAGTG
TGTTGGAGCACCATCGAGACCTGATGAAGGTAAGATGGGCTGTGGCTGAGG , 1900
AGCCTCTCTTGTCCCGGCAGGGTGGACCCATGGGAGTGCTATCAGGACACCTGGCAGACAACATGCAGTA
TGTTGGAGCACCATCGAGACCTGATGAAGGTAAGATGGGCTGTGGCTGAGG , 15
AGCCTCTCTTGTCCCGGCAGGGTGGACCCATGGGAGGGCTATCAGGACACCTGGCAGACAACCTGCAGTG
TGTTGGAGCACCATCGAGACCTGATGAAGGTAAGATGGGCTGTGGCTGAGG , 4
AGCCTCTCTTGTCCCGGCAGGGTGGACCCATGGGAGTGCTATCAGGACACCTGGCAGACAACCTGCAGTG
TGTTGGAGCACCATCGAGTCCTGATGAAGGTAAGATGGGCTGTGGCTGAGG , 3
AGCCTCTCTTGTCCCGGCAGGGTGGACCCATGGGAGTGCTATCAGGACACCCGGCAGACAACCTGCAGTG
TGTTGGAGCACCATCGAGACCTGATGAAGGTAAGATGGGCTGTGGCTGAGG , 3
AGCCTCTCTTGTCCCGGCAGGGCGGACCCATGGGAGTGCTATCAGGACACCTGGCAGACAACCTGCAGTG
TGTTGGAGCACCATCGAGACCTGATGAAGGTAAGATGGGCTGTGGCTGAGG , 3
AGCCTCTCTTGTCCCGGCAGGGTGGACCCATGGGAGTGCTATCAGGACACCTGGCAGACAACCCGCAGTG

TGTTGGAGCACCATCGAGACCTGATGAAGGTAAGATGGGCTGTGGCTGAGG , 2
AGCCTCTCTTGTCCCGGCAGGGTGGACCCATGGGCGTGCTATCAGGACACCTGGCAGACAACCTGCAGTG
TGTTGGAGCACCATCGAGACCTGATGAAGGTAAGATGGGCTGTGGCTGAGG , 2
AGCCTCTCTTGTCCCGGCAGGGTGGACCCATGGGAGTGCTATCAGGACACCTGGCAGACAACCTGCAGTG
TGTTGGAGCACCATCGGGACCTGATGAAGGTAAGATGGGCTGTGGCTGAGG , 2
AGCCTCTCTTGTCCCGGCAGGGTGGACCCATGGGAGTGCTATCAGGTCACCTGGCAGACAACCTGCAGTG
TGTTGGAGCACCATCGAGACCTGATGAAGGTAAGATGGGCTGTGGCTGAGG , 2
AGCCTCTCTTGTCCCGGCAGGGTGGACCCATGGGAGTACTATCAGGACACCTGGCAGACAACCTGCAGTG
TGTTGGAGCACCATCGAGACCTGATGAAGGTAAGATGGGCTGTGGCTGAGG , 2
AGCCTCTCTTGTCCCGGCAGGGTGGATCCATGGGAGTGCTATCAGGACACCTGGCAGACAACCTGCAGTG
TGTTGGAGCACCATCGAGACCTGATGAAGGTAAGATGGGCTGTGGCTGAGG , 2

GEIC-Plate04-H03 TOTAL:3305 OrderedDict([('sp2', 3284), ('T95M', 0), ('T95M only', 0), ('T95M Full ssODN', 0), ('V98M', 0), ('V98M_only', 0), ('V98M Full ssODN', 0), ('Silent Block only', 0), ('Silent Block only Full ssODN', 0)])   [(0, 3304), (-1, 1)]
AGCCTCTCTTGTCCCGGCAGGGTGGACCCATGGGAGTGCTATCAGGACACCTGGCAGACAACCTGCAGTG
TGTTGGAGCACCATCGAGACCTGATGAAGGTAAGATGGGCTGTGGCTGAGG , 3167
AGCCTCTCTTGTCCCGGCAGGGTGGACCCATGGGAGTGCTATCAGGACACCTGGCAGACAACCTGCAGTG
GGTTGGAGCACCATCGAGACCTGATGAAGGTAAGATGGGCTGTGGCTGAGG , 4
AGCCTCTCTTGTCCCGGCAGGGTGGACCCATGGGAGTGCTATCAGGACACCCGGCAGACAACCTGCAGTG
TGTTGGAGCACCATCGAGACCTGATGAAGGTAAGATGGGCTGTGGCTGAGG , 4
AGCCTCTCTTGTCCCGGCAGGGTGGACCCATGGGAGTGCTATCAGGACACCTGGCAGACAACCTGCGGTG
TGTTGGAGCACCATCGAGACCTGATGAAGGTAAGATGGGCTGTGGCTGAGG , 4
AGCCTCTCTTGTCCCGGCAGGGGGGACCCATGGGAGTGCTATCAGGACACCTGGCAGACAACCTGCAGTG
TGTTGGAGCACCATCGAGACCTGATGAAGGTAAGATGGGCTGTGGCTGAGG , 3
AGCCTCTCTTGTCCCGGCAGGGTGGACCCATGGGAGTGCTATCAGGACACCTGACAGACAACCTGCAGTG
TGTTGGAGCACCATCGAGACCTGATGAAGGTAAGATGGGCTGTGGCTGAGG , 3
AGCCTCTCTTGTCCCGGCAGGGTGGACCCATGGGGGTGCTATCAGGACACCTGGCAGACAACCTGCAGTG
TGTTGGAGCACCATCGAGACCTGATGAAGGTAAGATGGGCTGTGGCTGAGG , 3
AGCCTCTCTTGTCCCAGCAGGGTGGACCCATGGGAGTGCTATCAGGACACCTGGCAGACAACCTGCAGTG
TGTTGGAGCACCATCGAGACCTGATGAAGGTAAGATGGGCTGTGGCTGAGG , 3
AGCCTCTCTTGTCCCGGCAGGGTGGACCCATGGGAGTGCTATCAGGACACCTGGCAGACAACCTGCAGTG
TGTTGGAGCACCATCGAGACCTGATGACGGTAAGATGGGCTGTGGCTGAGG , 3
AGCCTCTCTTGTCCCGGCAGGGTGGACCCATGGGAGGGCTATCAGGACACCTGGCAGACAACCTGCAGTG
TGTTGGAGCACCATCGAGACCTGATGAAGGTAAGATGGGCTGTGGCTGAGG , 3
AGCCTCTCTTGTCCCGGCAGGGTAGACCCATGGGAGTGCTATCAGGACACCTGGCAGACAACCTGCAGTG
TGTTGGAGCACCATCGAGACCTGATGAAGGTAAGATGGGCTGTGGCTGAGG , 3
AGCCTCTCTTGTCCCGGCAGGGTGGACCCATGGGAGTGCTATCAGGACACCTGGCTGACAACCTGCAGTG
TGTTGGAGCACCATCGAGACCTGATGAAGGTAAGATGGGCTGTGGCTGAGG , 2

GEIC-Plate04-H04 TOTAL:2317 OrderedDict([('sp2', 2289), ('T95M', 0), ('T95M only', 0), ('T95M Full ssODN', 0), ('V98M', 13), ('V98M_only', 0), ('V98M Full ssODN', 12), ('Silent Block only', 0), ('Silent Block only Full ssODN', 0)])   [(0, 2313), (-1, 4)]
AGCCTCTCTTGTCCCGGCAGGGTGGACCCATGGGAGTGCTATCAGGACACCTGGCAGACAACCTGCAGTG
TGTTGGAGCACCATCGAGACCTGATGAAGGTAAGATGGGCTGTGGCTGAGG , 2200
AGCCTCTCTTGTCCCGGCAGGGTGGACCCATGGGAGTGCTATCAGGACACCTGGCAGACAACATGCAGTA

TGTTGGAGCACCATCGAGACCTGATGAAGGTAAGATGGGCTGTGGCTGAGG , 12
AGCCTCTCTTGTCCCGGCAGGGTGGACCCATGGGAGTGCTATCGGGACACCTGGCAGACAACCTGCAGTG
TGTTGGAGCACCATCGAGACCTGATGAAGGTAAGATGGGCTGTGGCTGAGG , 4
AGCCTCTCTTGTCCCGGCAGGGGGGACCCATGGGAGTGCTATCAGGACACCTGGCAGACAACCTGCAGTG
TGTTGGAGCACCATCGAGACCTGATGAAGGTAAGATGGGCTGTGGCTGAGG , 4
AGCCTCTCTTGTCCCGGCAGGGTGGACCCATGGGAGTGCTATCAGGACACCTGGCGGACAACCTGCAGTG
TGTTGGAGCACCATCGAGACCTGATGAAGGTAAGATGGGCTGTGGCTGAGG , 3
AGCCTCTCTTGTCCCGGCAGGGTGGACCCATGGGAGGGCTATCAGGACACCTGGCAGACAACCTGCAGTG
TGTTGGAGCACCATCGAGACCTGATGAAGGTAAGATGGGCTGTGGCTGAGG , 3
AGCCTCTCTTGTCCCGGCAGGGTGGACCCATGGGAGTGCTATCAGGACACCCGGCAGACAACCTGCAGTG
TGTTGGAGCACCATCGAGACCTGATGAAGGTAAGATGGGCTGTGGCTGAGG , 3
AGCCTCTCTTGTCCCGGCAGGGTGGACCCATGGGAGTGCTATCAGGACACCTGGCAGACAACCTGCAGTG
TGTTGGAGCGCCATCGAGACCTGATGAAGGTAAGATGGGCTGTGGCTGAGG , 3
AGCCTCTCTTGTCCCGGCAGGGCGGACCCATGGGAGTGCTATCAGGACACCTGGCAGACAACCTGCAGTG
TGTTGGAGCACCATCGAGACCTGATGAAGGTAAGATGGGCTGTGGCTGAGG , 3
AGCCTCTCTTGTCCCGGCAGGGTGGACCCATGGGAGTGCTATCAGGACACCTGGCAGACAACCTGCAGTG
TGTTGGAGCACAATCGAGACCTGATGAAGGTAAGATGGGCTGTGGCTGAGG , 3
AGCCTCTCTTGTCCCGGCAGGGTGGACCCATGGGAGTGCTATCAGGACACCTGGCAGACAACCTGCAGTG
TGTTGGAGCACCATCGAGGCCTGATGAAGGTAAGATGGGCTGTGGCTGAGG , 2
AGCCTCTCTTGTCCCGGCAGGGTGGACCCATGGGATTGCTATCAGGACACCTGGCAGACAACCTGCAGTG
TGTTGGAGCACCATCGAGACCTGATGAAGGTAAGATGGGCTGTGGCTGAGG , 2

GEIC-Plate04-H05 TOTAL:2591 OrderedDict([('sp2', 2572), ('T95M', 0), ('T95M only', 1), ('T95M Full ssODN', 0), ('V98M', 3), ('V98M_only', 0), ('V98M Full ssODN', 3), ('Silent Block only', 0), ('Silent Block only Full ssODN', 0)])   [(0, 2588), (-1, 3)]
AGCCTCTCTTGTCCCGGCAGGGTGGACCCATGGGAGTGCTATCAGGACACCTGGCAGACAACCTGCAGTG
TGTTGGAGCACCATCGAGACCTGATGAAGGTAAGATGGGCTGTGGCTGAGG , 2503
AGCCTCTCTTGTCCCGGCAGGGTGGACCCATGGGAGTGCTATCAGGGCACCTGGCAGACAACCTGCAGTG
TGTTGGAGCACCATCGAGACCTGATGAAGGTAAGATGGGCTGTGGCTGAGG , 4
AGCCTCTCTTGTCCCGGCAGGGTGGACCCATGGGAGTGCTATCAGGACACCTGGCAGACAACCTGCAGTG
GGTTGGAGCACCATCGAGACCTGATGAAGGTAAGATGGGCTGTGGCTGAGG , 4
AGCCTCTCTTGTCCCGGCAGGGTGGACCCATGGGAGTGCTATCAGGACACCCGGCAGACAACCTGCAGTG
TGTTGGAGCACCATCGAGACCTGATGAAGGTAAGATGGGCTGTGGCTGAGG , 4
AGCCTCTCTTGTCCCGGCAGGGTGGACCCATGGGAGTGCTATCAGGACACCTGGCAGACAACCTGCAGTG
TGTTGGAGCACCATCGAGACCTGATGAAGGTAAGATGGGCGGTGGCTGAGG , 3
AGCCTCTCTTGTCCCGGCAGGGTGGACCCATGGGAGTGCTATCAGGACACCTGGCAGACAACCTGCAGTG
TGTTGGAGCACCATCGAGACCTGATGAAGGTAAGGTGGGCTGTGGCTGAGG , 3
AGCCTCTCTTGTCCCGGCAGGGTGGACCCATGGGAGTGCTATCAGGACACCTGGCAGACAACATGCAGTA
TGTTGGAGCACCATCGAGACCTGATGAAGGTAAGATGGGCTGTGGCTGAGG , 3
AGCCTCTCTTGTCCCGGCAGGGTGGACCCATGGGAGTGCTATCAGGACACCTGGCAGACAACCTGCAGTG
TGTTGGAGCACCATCGAGGCCTGATGAAGGTAAGATGGGCTGTGGCTGAGG , 3
AGCCTCTCTTGTCCCGGCAGGGTGGACCCATGGGAGTGCTATCAGGACACCTGGCAGACAACCTGCAGTG
TGTTGGAGCACCATCGAGAACTGATGAAGGTAAGATGGGCTGTGGCTGAGG , 2
AGCCTCTCTTGTCCCGGCAGGGTGGACCCATGGGAGTGCTATCAGGACACCTGGCAGACAACCTGCAGTG
TGTTGGAGCACCATCGAGACCTGATGAAGTTAAGATGGGCTGTGGCTGAGG , 2
AGCCTCTCTTGTCCCGGCAGGGTGGACCCATGGGAGTGCTATCAGGACACCTGGCAGACAACCTGCAGTG
TGTTGGAGCACCATCGAGACCTGACGAAGGTAAGATGGGCTGTGGCTGAGG , 2
AGCCTCTCTTGTCCCGGCAGGGTGGGCCCATGGGAGTGCTATCAGGACACCTGGCAGACAACCTGCAGTG

TGTTGGAGCACCATCGAGACCTGATGAAGGTAAGATGGGCTGTGGCTGAGG , 2

GEIC-Plate04-H06 TOTAL:1653 OrderedDict([('sp2', 1635), ('T95M', 0), ('T95M only', 0), ('T95M Full ssODN', 0), ('V98M', 5), ('V98M_only', 0), ('V98M Full ssODN', 5), ('Silent Block only', 0), ('Silent Block only Full ssODN', 0)]) [(0, 1652), (-1, 1)]
AGCCTCTCTTGTCCCGGCAGGGTGGACCCATGGGAGTGCTATCAGGACACCTGGCAGACAACCTGCAGTG
TGTTGGAGCACCATCGAGACCTGATGAAGGTAAGATGGGCTGTGGCTGAGG , 1577
AGCCTCTCTTGTCCCGGCAGGGCGGACCCATGGGAGTGCTATCAGGACACCTGGCAGACAACCTGCAGTG
TGTTGGAGCACCATCGAGACCTGATGAAGGTAAGATGGGCTGTGGCTGAGG , 6
AGCCTCTCTTGTCCCGGCAGGGTGGACCCATGGGAGTGCTATCAGGACACCTGGCAGACAACATGCAGTA
TGTTGGAGCACCATCGAGACCTGATGAAGGTAAGATGGGCTGTGGCTGAGG , 5
AGCCTCTCTTGTCCCGGCAGGGTGGACCCATGGGAGTGCTATCAGGACACCTGGCAGACAACCCGCAGTG
TGTTGGAGCACCATCGAGACCTGATGAAGGTAAGATGGGCTGTGGCTGAGG , 3
AGCCTCTCTTGTCCCGGCAGGGGGGACCCATGGGAGTGCTATCAGGACACCTGGCAGACAACCTGCAGTG
TGTTGGAGCACCATCGAGACCTGATGAAGGTAAGATGGGCTGTGGCTGAGG , 3
AGCCTCTCTTGTCCCGGCAGGGTGGACCCATGGGAGTGCTATCAGGACACCTGGCAGACAACCTGCAGTG
TGTTGGAGCACCATCGAGGCCTGATGAAGGTAAGATGGGCTGTGGCTGAGG , 2
AGCCTCTCTTGTCCCGGCAGGGTGGACCCATGGGAGTGCTATCAGGACACCTGGCGGACAACCTGCAGTG
TGTTGGAGCACCATCGAGACCTGATGAAGGTAAGATGGGCTGTGGCTGAGG , 2
AGCCTCTCTTGTCCCGGCAGGGTGGACCCATGGGAGTGCTATCAGGACACCTGGCAGACAACCTGCAGCG
TGTTGGAGCACCATCGAGACCTGATGAAGGTAAGATGGGCGGTGGCTGAGG , 2
AGCCTCTCTTGTCCCGGCAGGGTGGACCCATGGGAGTGCTATCAGGACACCTGGCAGACAACCTGCAGTG
TGTTGAAGCACCATCGAGACCTGATGAAGGTAAGATGGGCTGTGGCTGAGG , 2
AGCCTCTCTTGTCCCGGCAGGGTGGACCCATGGGAGTGCTATCAGGACACCTGGCAGACAACCTTCAGTG
TGTTGGAGCACCATCGAGACCTGATGAAGGTAAGATGGGCTGTGGCTGAGG , 2
AGCCTCTCTTGTCCCGGCAGGGTGGACCCATGGGAGTGCTATCAGGACACCTGGCAGACAACCTGCAGTG
TGTTGGAGCACCATCGAGACCTGATGAAGGTAAGATGGACTGTGGCTGAGG , 2
AGCCTCTCTTGTCCCGGCGGGGTGGACCCATGGGAGTGCTATCAGGACACCTGGCAGACAACCTGCAGTG
TGTTGGAGCACCATCGAGACCTGATGAAGGTAAGATGGGCTGTGGCTGAGG , 1

GEIC-Plate04-H07 TOTAL:661 OrderedDict([('sp2', 309), ('T95M', 0), ('T95M only', 1), ('T95M Full ssODN', 0), ('V98M', 342), ('V98M_only', 0), ('V98M Full ssODN', 321), ('Silent Block only', 0), ('Silent Block only Full ssODN', 0)]) [(0, 660), (-1, 1)]
AGCCTCTCTTGTCCCGGCAGGGTGGACCCATGGGAGTGCTATCAGGACACCTGGCAGACAACATGCAGTA
TGTTGGAGCACCATCGAGACCTGATGAAGGTAAGATGGGCTGTGGCTGAGG , 321
AGCCTCTCTTGTCCCGGCAGGGTGGACCCATGGGAGTGCTATCAGGACACCTGGCAGACAACCTGCAGTG
TGTTGGAGCACCATCGAGACCTGATGAAGGTAAGATGGGCTGTGGCTGAGG , 286
AGCCTCTCTTGTCCCGGCAGGGTGGACCCATGGGAGTGCTATCAGGACACCTGGCAGACAGCATGCAGTA
TGTTGGAGCACCATCGAGACCTGATGAAGGTAAGATGGGCTGTGGCTGAGG , 2
AGCCTCTCTTGTCCCGGCAGGGTGGACCCATGGGAGTGCTATCAGGACACCTGGCAGACAACATGCAGTA
TGCTGGAGCACCATCGAGACCTGATGAAGGTAAGATGGGCTGTGGCTGAGG , 2
AGCCTCTCTTGTCCCGGCAGGGTGGACCCATGGGAGTGCTATCAGGACACCTGGCAGACAACATGCGGTA
TGTTGGAGCACCATCGAGACCTGATGAAGGTAAGATGGGCTGTGGCTGAGG , 2
AGCCTCTCTTGACCCGGCAGGGTGGACCCATGGGAGTGCTATCAGGACACCTGGCAGACAACATGCAGTA
TGTTGGAGCACCATCGAGACCTGATGAAGGTAAGATGGGCTGTGGCTGAGG , 2
AGCCTCTCTTGTCCCGGCAGGGTGGACCCATGGGAGTGCTATCAGGACACCTGGCAGACAACATGCAGCA

TGTTGGAGCACCATCGAGACCTGATGAAGGTAAGATGGGCTGTGGCTGAGG , 1
AGCCTCTCTTGTCCCGGCAGGGTGGACCCATGGGAGTGCTATCAGGACACCTGGCAGACAACGTGCAGTA
TGTTGGAGCACCATCGAGACCTGATGAAGGTAAGATGGGCTGTGGCTGAGG , 1
AGCCTCTCTTGTCCCGGCAGGGTGGACCCATGGGAGTGCTATCAGGACACCTGGCAGACAACATGCAGTA
TGTTGGAGCACCATCGAGACCTGATGAAGGTAAGATGGGCTATGGCTGAGG , 1
AGCCTCTCTTGTCCCGGCAGGGTGGACCCACGGGAGTGCTATCAGGACACCTGGCAGACAACCTGCAGTG
TGTTGGAGCACCATCGAGACCTGATGAAGGTAAGATGGGCTGTGGCTGAGG , 1
AGCCTCTCTTGTCCCGGCAGGGTGGACCCATGGGAGCGCTATCAGGACACCTGGCAGACAACCTGCAGTG
TGTTGGAGCACCATCGAGACCTGATGAAGGTAAGATGGGCTGTGGCTGAGG , 1
AGCCTCTCTTGTCCCGGCAGGGTGGACCATGGGAGTGCTATCAGGACACCTGGCAGACAACCTGCAGTGT
GTTGGAGCACCATCGAGACCTGATGAAGGTAAGATGGGCTGTGGCTGAGG , 1
